# Global Epidemiology of Amputation‐Related Disability From 1990 to 2021: Geographic Variation and Projections to 2050

**DOI:** 10.1002/jcsm.70356

**Published:** 2026-08-07

**Authors:** Xuhui Cuan, Jianguang Zhang, Zhuowen Lu, Fengyang Cui, Chenxi Zhao, Wenzhao Lu, Mengliang Luo, Song Xue, Chao Xie, Lei Jin, Meiling Shi, Jian Zhang, Meisong Zhu, Rongdong Liao, Xuanxuan Huang, Sikuan Zheng, Jianye Tan

**Affiliations:** ^1^ Department of Orthopedics The Second Affiliated Hospital, Jiangxi Medical College, Nanchang University Jiangxi China; ^2^ Department of Orthopedics Affiliated Hospital of North Sichuan Medical College Nanchong China; ^3^ Queen Mary College, Jiangxi Medical College, Nanchang University Nanchang Jiangxi China; ^4^ Department of Joint and Orthopedics Zhujiang Hospital, Southern Medical University Guangzhou China; ^5^ Department of Sports Medicine and Rehabilitation Peking University Shenzhen Hospital Shenzhen China; ^6^ The National Key Clinical Specialty, Engineering Research Center of Diagnostic and Therapeutic Technology and Devices for Cerebrovascular Diseases Zhujiang Hospital, Southern Medical University Guangzhou China; ^7^ Department of Anesthesiology The First Affiliated Hospital of USTC, Division of Life Sciences and Medicine, University of Science and Technology of China Hefei China; ^8^ Institute of Molecular Physiology Shenzhen Bay Laboratory Shenzhen China

**Keywords:** ageing, amputation, disability, Global Burden of Disease, physical function

## Abstract

**Background:**

Amputation is a critical event leading to the permanent loss of limb function and long‐term functional impairment, posing a substantial burden on global healthcare systems. However, the global burden, demographic patterns and future trajectory of amputation‐related disability have not been comprehensively quantified.

**Methods:**

We analysed Global Burden of Disease (GBD) 2021 data to assess the prevalence and years lived with disability (YLDs) for seven amputation subtypes (fingers, thumbs, toes, unilateral and bilateral upper and lower limbs) from 1990 to 2021. Projections to 2050 were made using the Bayesian age–period–cohort (BAPC) modelling.

**Results:**

In 2021, the global total number of amputation cases reached approximately 445 million (95% uncertainty intervals [UI]: 409–486 million), with an age‐standardized rate (ASR) of 5330 (95% UI: 4890–5820) cases per 100 000 population. From 1990 to 2021, the overall global burden of amputation demonstrated a declining trend in ASR, although the absolute number of cases increased substantially. Among all amputation subtypes, finger amputation (AMP‐F) had the highest prevalence, accounting for 50.3% of all cases, followed by thumb amputation (AMP‐Th) at 22.4% and toe amputation (AMP‐Toe) at 19.0%; these three subtypes alone accounted for over 91% of all cases. Regarding YLDs, unilateral lower limb amputation (AMP‐LL1) contributed most significantly (20.8%), followed by bilateral upper limb amputation (AMP‐UL2) at 18.9%, despite their relatively lower prevalence. In terms of the YLD burden, the age distribution exhibited a spindle‐shaped pattern, peaking in the 30–59 age group overall, with notable subtype‐specific variations (e.g., AMP‐LL1 peaking at ≥ 95 years and bilateral lower limb amputation [AMP‐LL2] prominent in middle‐aged groups, particularly 50–64 years in certain regions). Additionally, the etiological profile shifted markedly, with conflict and terrorism rising sharply from the 8th to the 4th leading driver of amputation burden globally, exerting a particularly strong impact in North Africa and the Middle East. Projections indicate substantial future increases in both prevalence and YLDs associated with AMP‐LL1, necessitating heightened vigilance.

**Conclusion:**

Although the age‐standardized prevalence of amputation slightly declined globally from 1990 to 2021, the absolute number of cases increased significantly, exhibiting pronounced demographic and geographic disparities. To mitigate this escalating burden, tailored strategies integrating prevention and long‐term rehabilitation planning are urgently needed.

**Abbreviations**
AMP‐F: Amputation of fingers (excluding thumb)
AMP‐LL1: Amputation of lower limb, unilateral
AMP‐LL2: Amputation of lower limbs, bilateral
AMP‐Th: Amputation of thumb
AMP‐Toe: Amputation of toe/toes
AMP‐UL1: Amputation of upper limb, unilateral
AMP‐UL2: Amputation of upper limbs, bilateral
ASR: Age‐standardized rate
BAPC: Bayesian age–period–cohort
DFU: Diabetic foot ulcers
EAPC: Estimated annual percentage change
GBD: Global Burden of Disease
IDF: International Diabetes Federation
INLA: Integrated nested Laplace approximation
LBMD: Low bone mineral density
MR‐BRT: Meta‐regression‐Bayesian regularization trimming
PAD: Peripheral artery disease
RW2: Second‐order random walk
SDI: Socio‐demographic index
UI: Uncertainty intervals
YLDs: Years lived with disability

## 1 Introduction

Amputation is a surgical procedure involving the partial or complete removal of a limb that has lost its viability, physiological function, or poses a severe threat to life [1, 2, 3]. Although often life‐saving, amputation results in irreversible functional loss and long‐term disability, frequently leading to impaired mobility, reduced physical capacity and prolonged dependence on rehabilitation. These consequences are particularly pronounced among older adults and individuals with chronic diseases such as diabetes, making amputation an important contributor to the global disability burden.

Existing studies have mainly focused on specific etiologies or selected populations, such as diabetic foot disease, traumatic upper limb injury or tumour‐related amputation [4, 5]. While these studies have improved understanding of local risk factors and clinical outcomes, three important gaps remain. First, comprehensive cross‐national epidemiological assessments are limited, restricting comparisons across regions with different socioeconomic conditions. Second, most studies focus on individual amputation types or etiologies, with few providing systematic comparisons across anatomical sites or unilateral and bilateral amputations. Third, evidence on the long‐term disability burden and temporal trends of amputation remains insufficient, limiting evaluation of changes associated with advances in healthcare and public health interventions.

**FIGURE 1:**
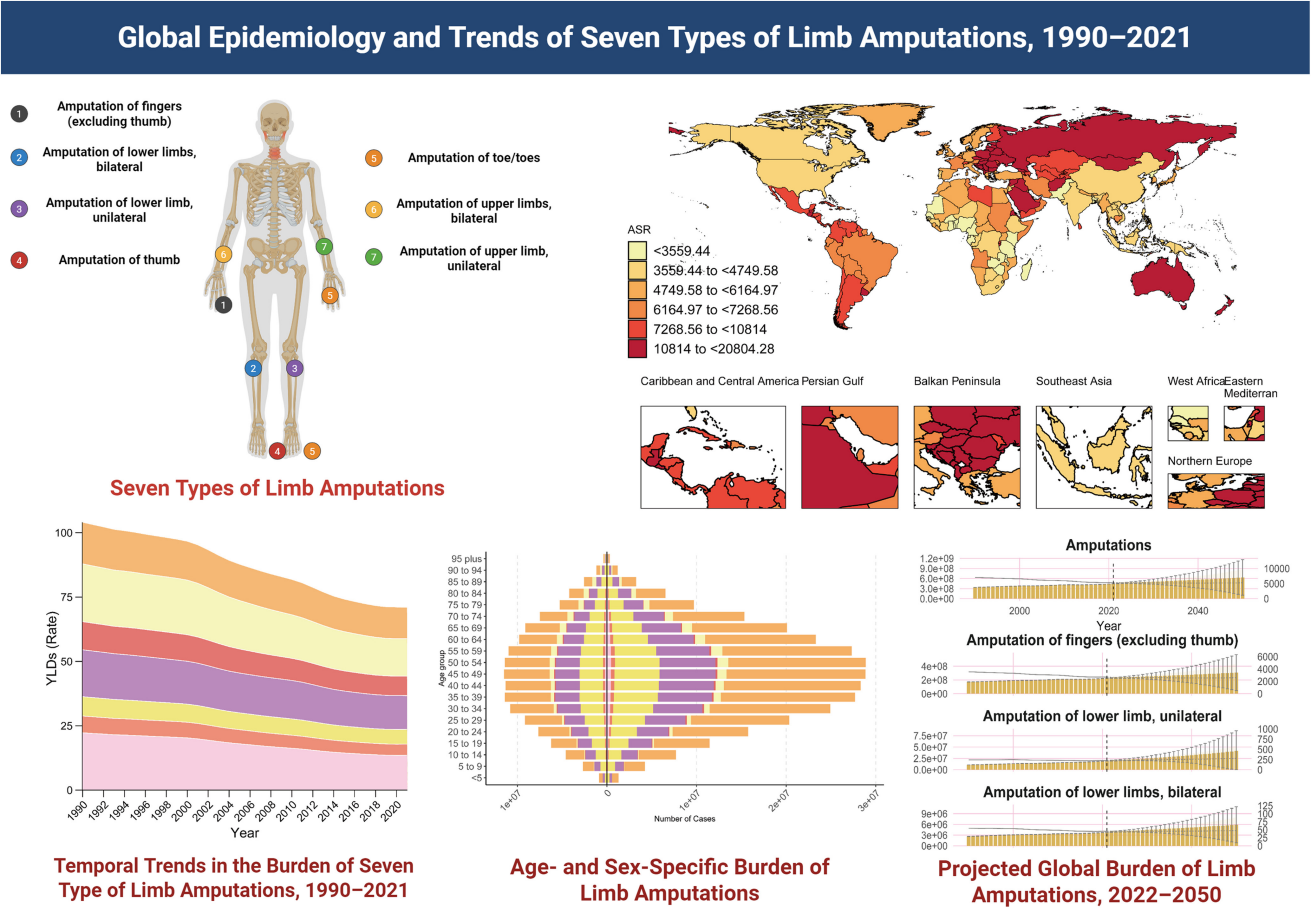
Overview of global epidemiology and trends of seven types of limb amputations, 1990–2021. Schematic representation of seven subtypes of limb amputations.

Using data from the Global Burden of Disease (GBD) 2021 study, we conducted a systematic assessment of seven amputation subtypes, including amputations of fingers, thumbs, toes and unilateral and bilateral amputations of the upper and lower limbs. We analysed trends in prevalence and years lived with disability (YLDs) by age, sex and region from 1990 to 2021 (Figure 1). By characterizing the long‐term and heterogeneous burden of amputation‐related disability across populations, this study aims to provide evidence to inform prevention strategies, rehabilitation planning and resource allocation at both global and regional levels, with particular relevance to ageing populations and the growing burden of chronic disease.

## 2 Methods

### 2.1 Study Population and Data Collection

This secondary analysis used GBD 2021 data to assess the epidemiological characteristics of overall amputation and seven subtypes across all age groups. The dataset covers 204 countries and territories grouped into 21 regions and provides estimates for 369 diseases, injuries and impairments, as well as 88 risk factors. Within the GBD framework, amputation encompasses seven subtypes (Table S1).

Key metrics analysed include prevalence, YLDs and their estimated annual percentage change (EAPC). Data were processed using meta‐regression‐Bayesian regularized trimmed (MR‐BRT) and DisMod‐MR 2.1 models to adjust for differences among data sources and improve cross‐study comparability [6, 7, 8].

To facilitate population comparisons, age‐standardized rate (ASR) was computed for each amputation subtype. The ASR calculations utilized direct age standardization, with the GBD 2021 standard population serving as the weighting factor, and followed the formula outlined below:

$$\frac{\sum_{i=1}^{N}a_{i}W_{i}}{\sum_{i=1}^{N}W_{i}}$$

In this context, a_i_ signifies the particular rate pertaining to the ith age group, W_i_ stands for the standard population weight allocated to that respective age group and N indicates the overall count of age groups. The calculation of statistical estimations was performed utilizing the ‘age.adjust.direct’ function available in the ‘epitools’ package within the R software environment, with the threshold for statistical significance established at *p* < 0.05.

### 2.2 Trend Forecasting Based on the Bayesian Age–Period–Cohort (BAPC) Model

The BAPC model was used to project global amputation prevalence and burden to 2050, with attention to subtype differences by laterality and limb site. The model was fitted using the BAPC package in R based on GBD 2021 data from 1990 to 2021. Bayesian inference was performed using the integrated nested Laplace approximation (INLA), with the second‐order random walk (RW2) priors and penalized complexity (PC) hyperpriors specified for the age, period and cohort effects to ensure smooth estimation. Future trends were estimated by extrapolating period and cohort effects. To evaluate the predictive performance of the model, we additionally performed a temporal validation analysis. For temporal validation, the model was refitted using 1990–2015 data to predict the 2016–2021 burden. Predictive performance was assessed by comparing predicted and observed GBD estimates, calculating mean absolute percentage error (MAPE) and examining whether observed values fell within the predicted 95% credible intervals.

## 3 Results

### 3.1 Global, Regional and National Burden of Overall Amputations

In 2021, global amputations totalled approximately 445 million cases (95% uncertainty interval [UI]: 409–486 million), corresponding to an ASR prevalence of 5330 (95% UI: 4890–5820) cases per 100 000 population. Concurrently, the total YLDs reached 5.94 million cases, with an ASR YLDs of 71.1 (Table S2). Among the 21 GBD regions, Central Europe, Australasia and Eastern Europe had the highest prevalence ASRs at 16 100, 13 100 and 12 600, respectively. Concurrently, from the perspective of ASR YLDs, Central Europe (145), Eastern Europe (131) and North Africa and Middle East (120) ranked foremost (Table S2). Nationally, Afghanistan recorded the highest ASR prevalence (20 800), followed by Albania (20 600) and Slovenia (20 100). Afghanistan also had the highest ASR YLDs burden (621), followed by Eritrea (399) and Burundi (369).

**FIGURE 2:**
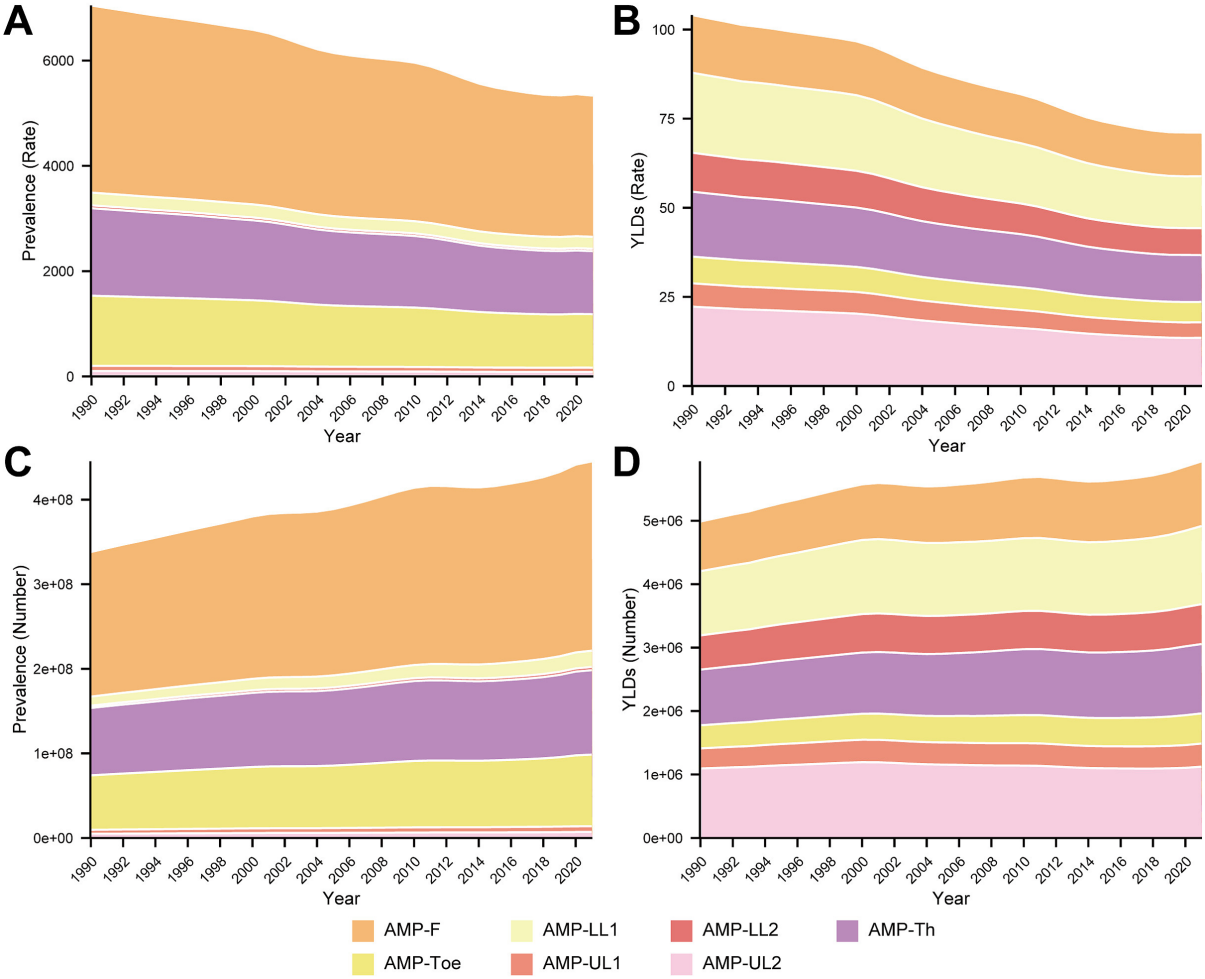
Temporal trends in the burden of seven amputation subtypes, from 1990 to 2021. The figure presents global trends across four metrics: (A) ASR of prevalence, (B) ASR of YLDs, (C) total number of prevalent cases and (D) total number of YLDs. Different coloured areas represent the seven amputation subtypes as indicated in the legend.

From 1990 to 2021, global ASR prevalence and YLDs declined, with EAPCs of −0.99, and −1.39, respectively (Figure 2 and Table S2). Regionally, High‐income North America saw the steepest reduction (EAPC=−1.70); East Asia recorded the sharpest ASR YLDs decrease (EAPC=−2.36) (Table S2). Among all geographical regions, Portugal and Republic of Korea experienced the fastest ASR prevalence declines, with EAPCs of −2.07 and −1.88, respectively, whereas Lebanon and Estonia showed the most rapid ASR YLDs reductions, with EAPCs of −3.53 and −2.71, respectively. In contrast, the Burundi, Syrian Arab Republic and Palestine exhibited ASR prevalence increases, with EAPCs of 3.74, 2.91 and 1.07, respectively. Meanwhile Burundi, the Syrian Arab Republic, and Haiti demonstrated ASR YLDs rises, with EAPCs of 5.34, 3.54, and 1.83, respectively (Table S3).

### 3.2 Regional Differences in the Burden of Seven Amputations

In 2021, the global prevalence of amputations was as follows: amputation of fingers (excluding thumb) (AMP‐F) totalled 224 million cases, amputation of lower limb, unilateral (AMP‐LL1) 19.3 million cases, amputation of lower limbs, bilateral (AMP‐LL2) 3.80 million cases, amputation of thumb (AMP‐Th) 100 million cases, amputation of toe/toes (AMP‐Toe) 84.6 million cases, unilateral amputation of upper limb (AMP‐UL1) 6.80 million cases and amputation of upper limbs, bilateral (AMP‐UL2) 7.14 million cases. Correspondingly, the ASR prevalence for these types of amputations was 2680, 228, 45.4, 1200, 1020, 81.2 and 85.4 per 100 000 population, respectively. Regarding YLDs, AMP‐F reached 1.02 million cases, AMP‐LL1 1.23 million cases, AMP‐LL2 629 000 cases, AMP‐Th 1.09 million cases, AMP‐Toe 476 000 cases, AMP‐UL1 364 000 cases and AMP‐UL2 1.12 million cases, with the corresponding ASR of YLDs of 12.2, 14.6, 7.56, 13.1, 5.71, 4.37 and 13.5 (Table S4).

**FIGURE 3:**
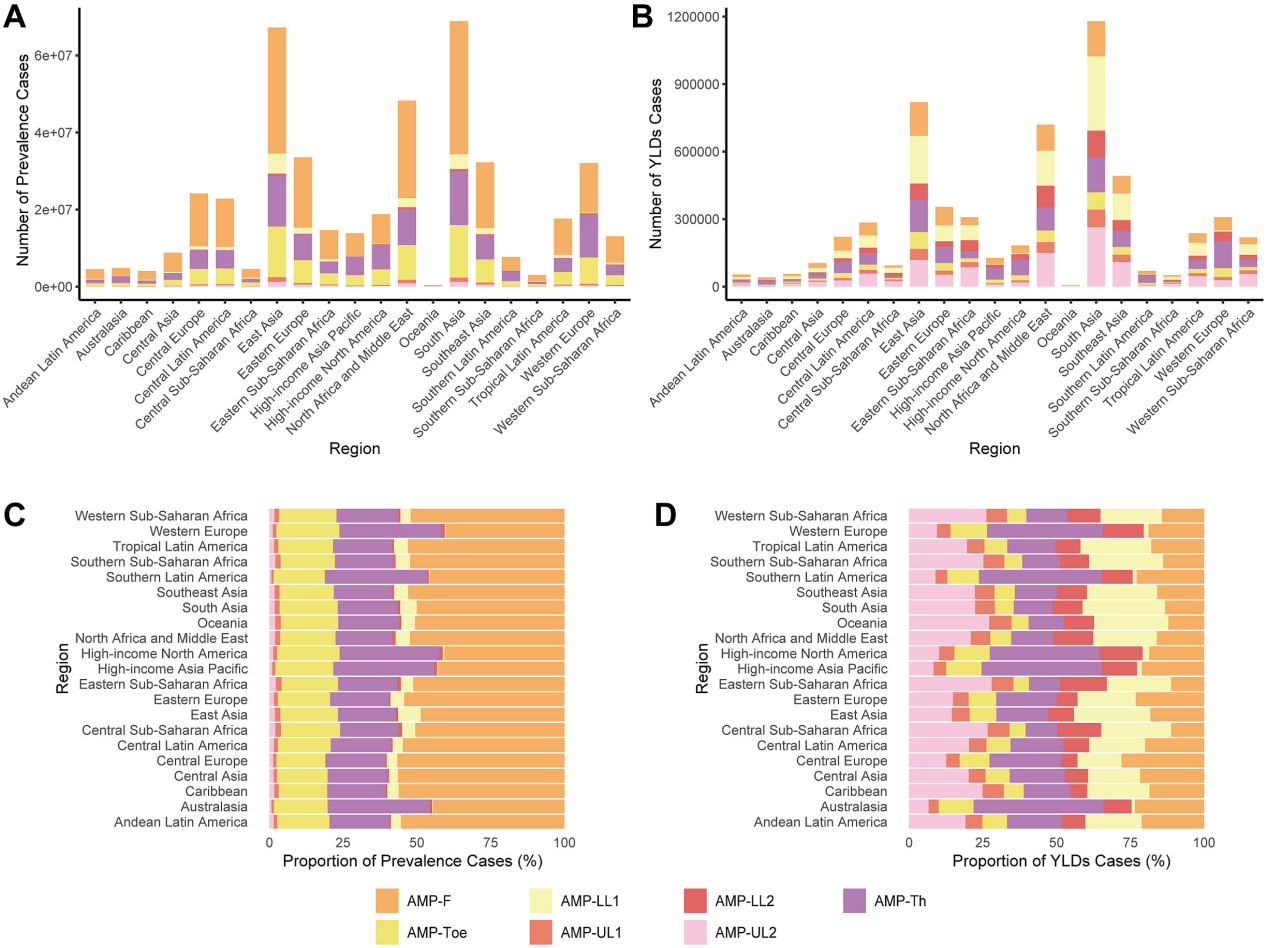
Distribution and proportion of burden of prevalence cases and YLDs cases across 21 GBD regions for seven amputation subtypes in 2021. (A, B) Distribution of burden of prevalence cases and YLDs cases across 21 GBD regions for seven amputation subtypes in 2021. (C, D) Proportion of burden of prevalence cases and YLDs cases attributable to seven amputation subtypes across 21 GBD regions in 2021.

The burden across the seven subtypes varied significantly across the 21 GBD regions. Central Europe reported the highest overall ASR prevalence (16 100.1), with AMP‐F contributing most (9180), followed by AMP‐Th (3330) and AMP‐Toe (2720) (Figure 3 and Tables S5, S8, S9). Globally, AMP‐F ranked first in prevalence and fourth in YLDs. In Central and Eastern Europe, it accounted for over 54% of cases and 23% of YLDs, exceeding 28% YLDs in Central Europe (Figure 3 and Table S5). AMP‐Th ranks second in prevalence and third in YLDs. Prominent in Australasia and Southern South America, it accounted for 34% of cases and over 40% of YLDs (Figure 3 and Table S8). AMP‐Toe ranks third in prevalence and sixth in YLDs, accounting for more than 16% of cases in Central Europe and Australasia but only approximately 8% of YLDs (Figure 3 and Table S9). AMP‐LL1 ranked fourth in prevalence but first in YLDs. It affects over 5% of the population in East Asia and South Asia, accounting for more than 20% of global YLDs, presenting a stark contrast between low prevalence and high YLD contribution (Figure 3 and Table S6). AMP‐UL2 exhibits similar characteristics: fifth in prevalence and ranked second in YLDs globally. Despite affecting just over 1% of the population in Eastern Europe, North Africa and the Middle East, it contributes over 18% of global YLDs (Figure 3 and Table S11). As for AMP‐UL1, it ranks sixth in prevalence and seventh in YLDs, with its highest prevalence in Eastern Europe, North Africa and the Middle East, where cases account for over 1% of the global total (Figure 3 and Table S10). AMP‐LL2 ranks fifth in YLDs and seventh in prevalence, with prevalence below 1% in most regions (Figure 3 and Table S7).

At the national level, Afghanistan recorded the highest ASR for AMP‐LL1, AMP‐LL2, AMP‐Toe, AMP‐UL1 and AMP‐UL2 in 2021, at 1570, 510, 4070, 592 and 656 respectively (Figures S2, S3, S6 and S7 and Tables S13, S14, S16, S17 and S18). Regarding ASR for AMP‐F, Albania led with 12 000 followed by Slovenia (11 400) and Bulgaria (10 800) (Figures S1–S7 and Table S12). For the ASR of AMP‐Th prevalence, New Zealand led with 5300, followed by Australia (4420) and Albania (4290) (Figure S4 and Table S15). Excluding Afghanistan's outlier value, the Syrian Arab Republic (1260) and Iraq (1200) exhibited relatively high ASR for AMP‐LL1 prevalence (Figure S3 and Table S13). For AMP‐LL2, the Syrian Arab Republic (375) and Eritrea (325) exhibited notably high ASR (Figure S2 and Table S14); in AMP‐Toe, Slovenia (3370) and Iraq (3360) stood out (Figure S5 and Table S16); regarding ASR for AMP‐UL1 prevalence, the Syrian Arab Republic (437) and Iraq (394) exhibited relatively high rates (Figure S7 and Table S17); for AMP‐UL2 prevalence, the Syrian Arab Republic (466) and Iraq (439) also ranked among the highest (Figure S6 and Table S18).

Meanwhile, in terms of the highest ASR of YLDs for these amputations, Albania led for AMP‐F (54.9) and AMP‐Toe (19.0), New Zealand led for AMP‐Th (58.4) and Afghanistan dominated the remaining five types: AMP‐LL1 (168), AMP‐LL2 (126), AMP‐UL1 (46.5) and AMP‐UL2 (174) (Tables S13, S14, S17 and S18). Within the AMP‐F subtype, Slovenia (52.2) and Bulgaria (49.4) ranked second and third, respectively, in YLDs (Table S12); within the AMP‐LL1 subtype, Eritrea (103) and Burundi (102) ranked second and third, respectively, immediately following the highest burden region (Table S13); within the AMP‐LL2 subtype, Eritrea (84.9) and Rwanda (76.5) ranked second and third, respectively, in YLDs (Table S14); for the AMP‐Th subtype, Australia (48.7) and Albania (47.3) ranked second and third in YLDs, respectively, following the highest ranking region (Table S15); for the AMP‐Toe subtype, Slovenia (19.0) and Iraq (18.7) ranked second and third in YLDs, respectively, following the leading region (Table S16); for the AMP‐UL1 subtype, Eritrea (31.3) demonstrated outstanding performance, with Rwanda (27.8) following closely in the second and third places, respectively (Table S17); for the AMP‐UL2 subtype, Eritrea (117) ranked first, with Burundi (104) performing notably well in the second and third places, respectively (Table S18).

**FIGURE 4:**
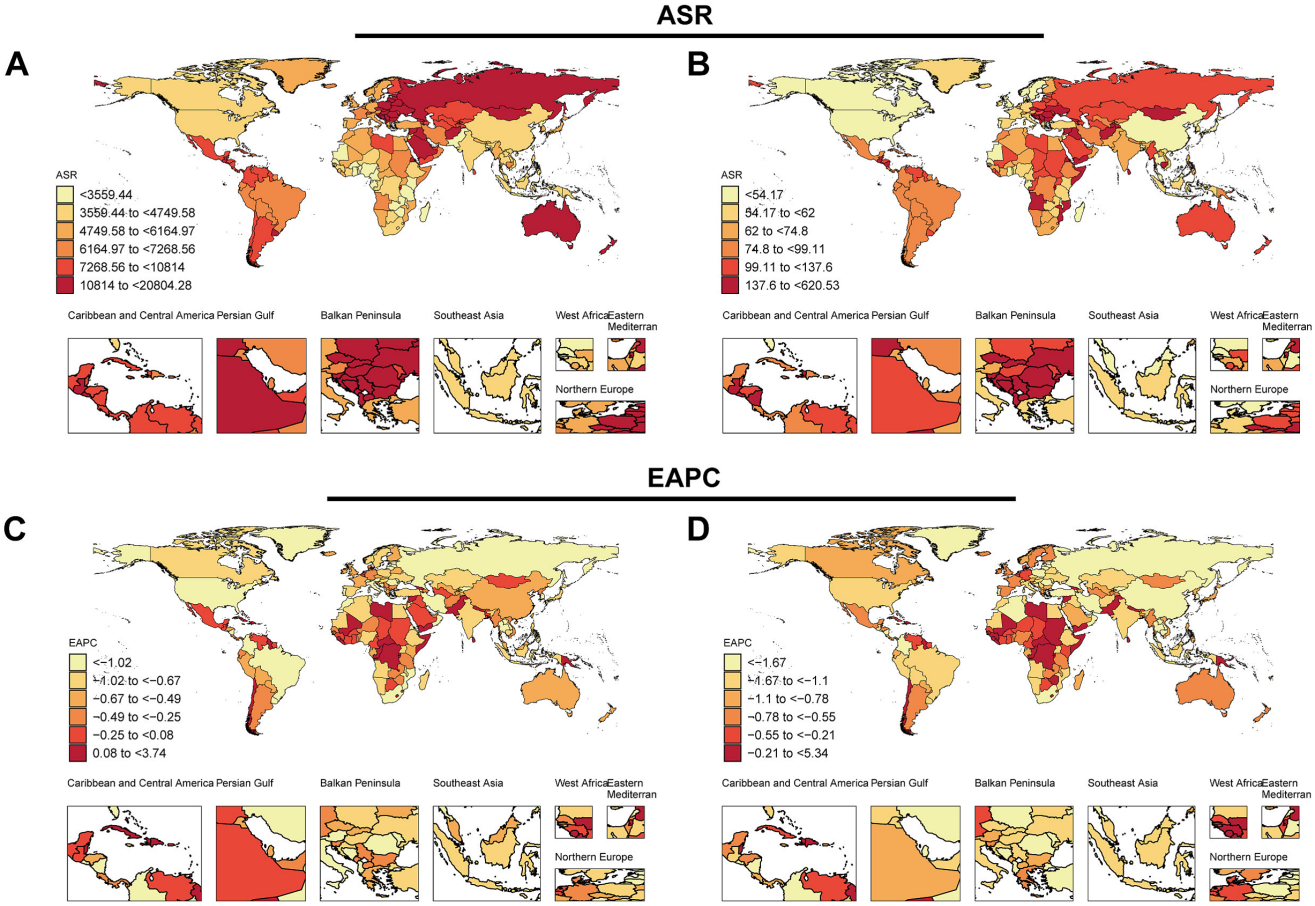
Global distribution of the burden and EAPC for overall amputations from 1990 to 2021. (A) Global distribution of the burden for overall amputations in 2021. (B) Global distribution of ASR YLDs of overall amputations in 2021. (C) Global distribution of EAPC of overall amputations from 1990 to 2021. (D) Global distribution of EAPC of ASR YLDs of overall amputations from 1990 to 2021.

From 1990 to 2021, all amputation types showed declining ASR prevalence, with notable reductions for AMP‐Th (EAPC = −1.15), AMP‐F (−0.99) and AMP‐Toe (−0.99); AMP‐LL1 had the lowest decline (−0.35). Global ASR YLD patterns mirrored prevalence, with significant declines in AMP‐UL2 (EAPC = −1.81), AMP‐LL1 (−1.64) and AMP‐UL1 (−1.48) (Figures 4 and S1–S7 and Table S19).

The seven amputation types exhibit distinct disease patterns and regional variations in ASR. For prevalence, AMP‐F constitutes the primary prevalence contributor (ASR prevalence = 2680), particularly in Central Europe (9180), Eastern Europe (6860) and Australasia (5820) (Figure S1 and Tables S4 and S5). Regarding YLDs, AMP‐LL1 (ASR YLDs = 14.6) emerged as the primary contributor, with the highest rates in Eastern Sub‐Saharan Africa (28.1), Central Sub‐Saharan Africa (27.7) and North Africa and the Middle East (27.6) (Figure S3 and Tables S4 and S5). Despite the global decline, AMP‐LL2 and AMP‐LL1 prevalence ASRs increased in North Africa and the Middle East, with EAPCs of 3.25 and 1.38, respectively. Nevertheless, AMP‐LL1 prevalence remained the highest in Eastern Europe (517) and Central Europe (466). AMP‐Th prevalence was the highest in Australasia (4570), Southern Latin America (3530) and Central Europe (3330), while its highest YLDs occurred in Australasia (50.3), Southern Latin America (38.9) and Central Europe (36.6) (Figure S4 and Tables S4 and S8). AMP‐Toe was prevalent in Europe, with the top three regions overlapping for prevalence and YLDs: Central Europe (ASR prevalence = 2720; ASR YLDs = 15.4), Australasia (2410; 13.6) and Eastern Europe (2250; 12.7) (Figure S5 and Tables S4 and S9). Regarding upper limb amputations, AMP‐UL1 exhibited lower ASR prevalence, yet Central Europe (168), Eastern Europe (165) and North Africa and the Middle East (142) remained peak prevalence zones; the regions with the highest YLDs were concentrated in the Caribbean (8.17), North Africa and the Middle East (7.82) and Eastern Sub‐Saharan Africa (7.69) (Figure S7 and Tables S4 and S10). AMP‐UL2 had comparable global prevalence, with similar prevalence peaks in Central Europe (204), Eastern Europe (187) and North Africa and the Middle East (152) and the highest YLDs in Eastern Sub‐Saharan Africa (28.8), the Caribbean (28.3) and Central Sub‐Saharan Africa (25.1) (Figure S6 and Tables S4 and S11).

### 3.3 Age‐Specific Patterns of Amputation Burden in Population Health

**FIGURE 5:**
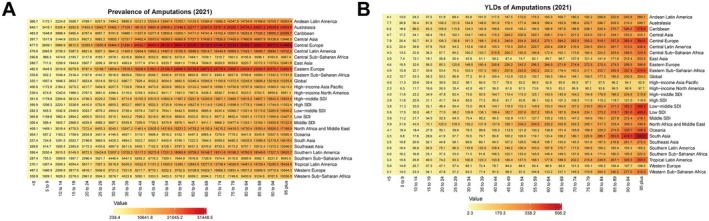
Gender‐specific burden and YLDs of overall amputations across 21 GBD regions in 2021. The number of cases of prevalence (A) and YLDs (B) for overall amputations across 21 GBD regions.

The age distribution of amputation burden varied markedly by subtype. Overall prevalence followed a spindle‐shaped curve, peaking at 40–80 years (Figure 5). AMP‐F showed a similar pattern, peaking at 40–89 years (Figure S8A). AMP‐LL1 increased almost continuously with age, peaked at ≥ 95 years (narrow single‐peak distribution) (Figure S10A). AMP‐Th and AMP‐Toe both increased then declined, peaking at 40–94 years (Figures S11A and S12A). AMP‐LL2, AMP‐UL1 and AMP‐UL2 showed similar age patterns but substantial regional differences. AMP‐LL2 peaked in the over‐95 age group in Australia (maximum prevalence = 280) and Western Europe (203), whereas in North Africa and the Middle East, the peak occurred at 50–64 years, with the highest prevalence 223.5 observed in the 55–59 age cohort (Figure S9A). AMP‐UL1 and AMP‐UL2 peaked in the 60–95 age group in Central Europe (maximum prevalence: unilateral 452, bilateral 463) and Eastern Europe (unilateral 368, bilateral 369). In North Africa and the Middle East, the peak similarly shifted to the 50–64 age group, with the highest prevalence in the 55–59 age cohort at 330 and 353, respectively (Figures S13A and S14A).

On the YLDs dimension, the overall burden peaked at ≥ 80 years, with subtype variations: AMP‐LL2 and AMP‐UL2 peaked earlier (35–59 years); AMP‐UL1 shows dual peaks (35–59 and ≥ 85 years), reflecting impacts on working‐age and elderly populations; finger/toe amputations peaked in middle‐to‐late adulthood (50–79 years), with AMP‐F (50–74), AMP‐Th (55–79), and AMP‐Toe (50–79) declining gradually thereafter. This ‘early peak‐slow decline’ pattern highlights the need for functional rehabilitation in middle age. Conversely, AMP‐LL1 exhibits a monotonically increasing YLD trajectory, peaking at ≥ 95 years, aligning with its prevalence pattern and indicating sustained health demands among the super‐elderly (Figures S8–S14).

### 3.4 Gender‐Specific Patterns of Amputation Burden in Population Health

**FIGURE 6:**
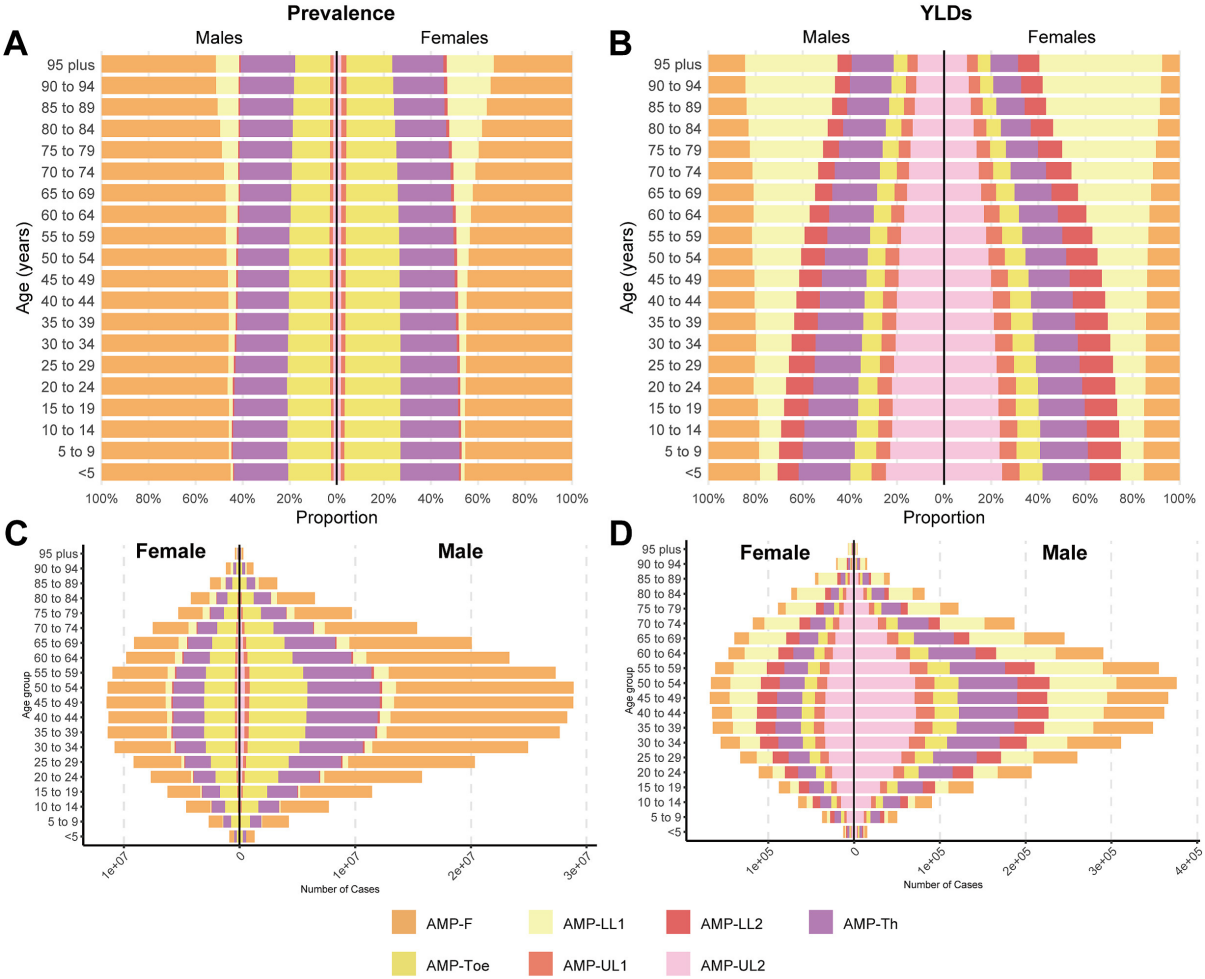
Age‐ and gender‐specific prevalence, and YLDs of seven amputation subtypes in 2021. (A, B) Proportional distribution of prevalence (A), and YLDs (B) by age group and sex. (C, D) The number of cases for prevalence (C) and YLDs (D) by age group and sex.

Gender disparities were analysed across prevalence, YLDs and subtype composition. AMP‐F dominated in ages 0–69 (males ~55%, females > 40%), declining with age. AMP‐LL1 rose sharply after age 65, ranking among the top four subtypes, with females constituting twice the proportion of males in the elderly. AMP‐Toe showed a spindle‐shaped age distribution, peaking in adults aged 19–59 (Figure 6). Regarding YLDs, AMP‐LL1 contributed > 50% of the burden in those aged ≥ 85, increasing with age, while AMP‐F accounted for < 20%, declining more rapidly in females after age 60. AMP‐LL2 was twice as prevalent in females as males, decreasing with age. AMP‐UL2's proportion declined sharply in ages 0–9 and ≥ 60 years (from 20% to 10%) (Figure 6).

### 3.5 The Influence of Socio‐Demographic Index (SDI) on Amputation Burden

**FIGURE 7:**
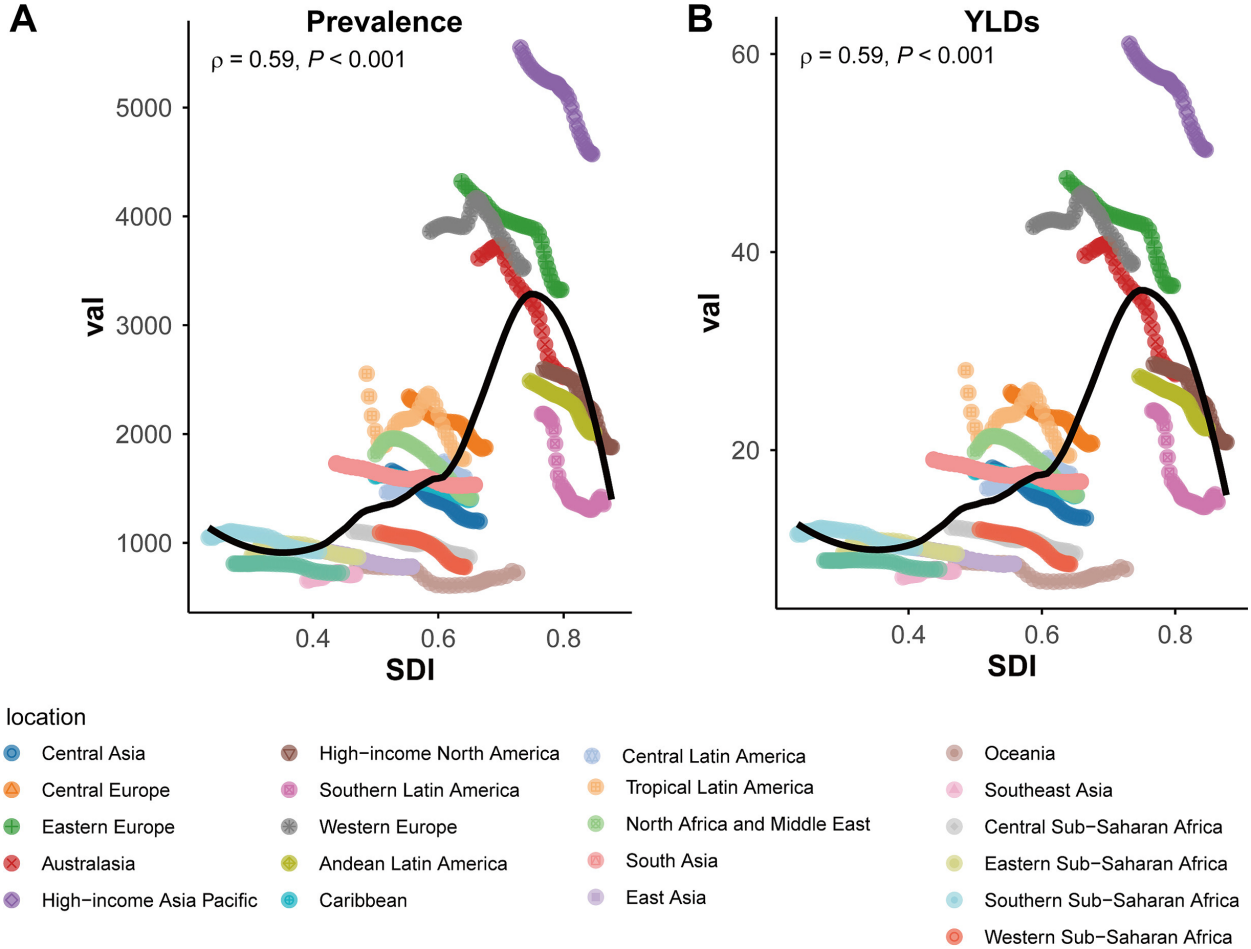
Curves of the correlation between the prevalence of overall amputations and SDI and YLDs with SDI in 2021. (A) Prevalence and SDI. (B) YLDs and SDI.

The ASR of amputation prevalence across 21 GBD regions showed an inverted spoon‐shaped correlation with SDI (*ρ* = 0.43, *p* < 0.01). As SDI rises, prevalence slightly declines at low levels (SDI < 0.3), increases rapidly after ~SDI 0.3, peaks around SDI 0.7–0.8, then decreases (Figure 7), suggesting that the burden rises with socioeconomic development but may be reduced in high SDI settings through improved healthcare. Subtypes varied in SDI responsiveness: AMP‐Th (*ρ* = 0.59, *p* < 0.01), AMP‐Toe (*ρ* = 0.45, *p* < 0.01) and AMP‐F (*ρ* = 0.37, *p* < 0.01) rose sharply from low to medium to high SDI regions, peaking at ~SDI 0.8 before declining (Figures S15A, S18A and S19A). AMP‐LL2 (ρ = 0.43, *p* < 0.01) followed an S‐shaped pattern, initially declining rapidly to a minimum around SDI 0.3–0.4, then sharply ascending to peak at SDI 0.7–0.8 before decreasing (Figure S16A). In contrast, AMP‐UL2 (*ρ* = 0.08, *p* < 0.01) and AMP‐UL1 (*ρ* = 0.18, *p* < 0.01) showed weaker, more stable associations with SDI (Figures S20A and S21A), suggesting greater influence from physiological than socioeconomic factors. AMP‐LL1 (*ρ* = −0.08, *p* < 0.01) exhibited a weak negative correlation, initially declining slightly with SDI, reaching a minimum around SDI 0.35, remaining stable at SDI 0.5–0.7, then declining rapidly (Figure S17A).

### 3.6 Future Forecasts for Global Prevalence of Amputations

**FIGURE 8:**
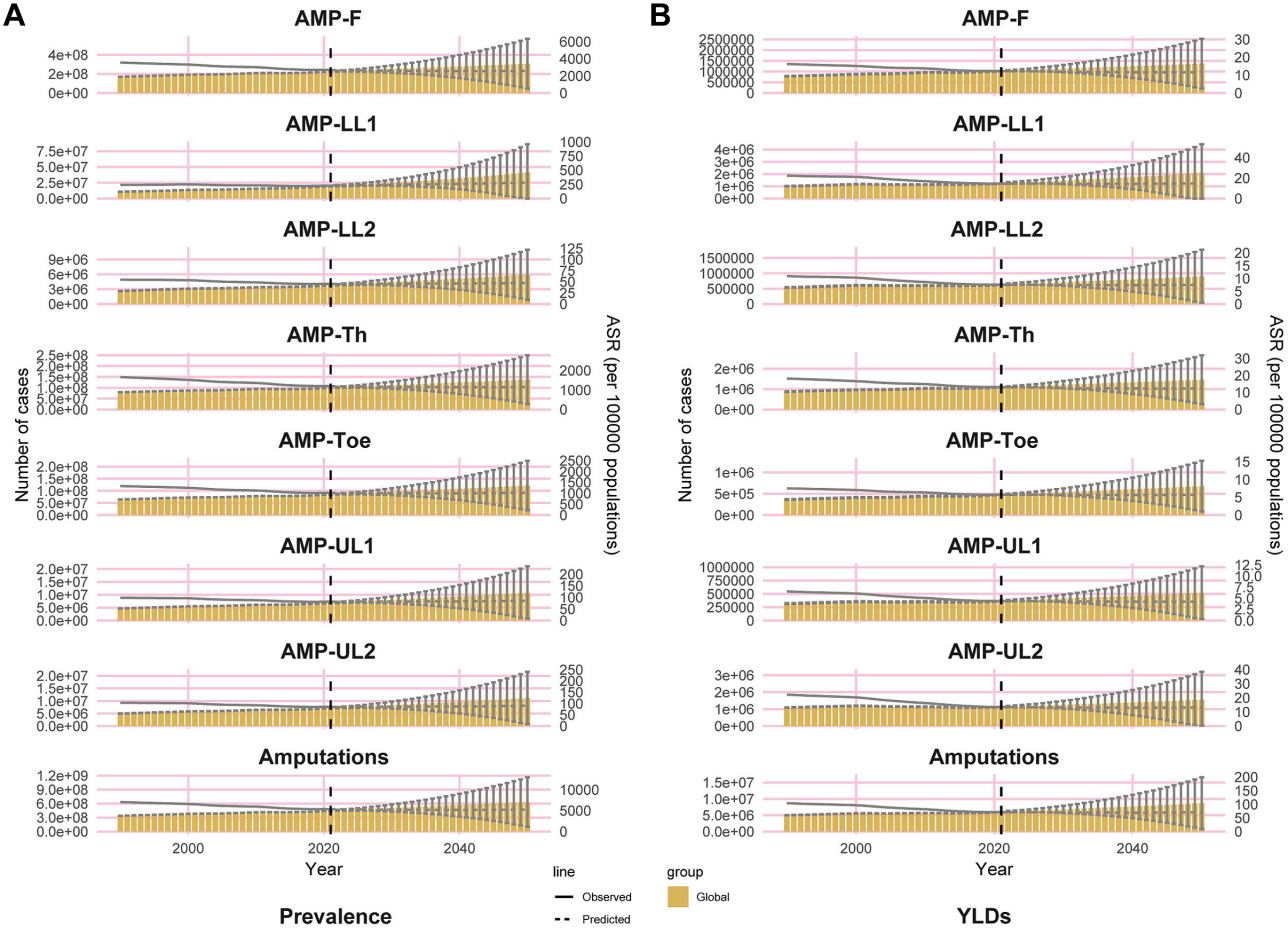
Projected prevalence and YLDs cases and ASR of prevalence and YLDs of overall amputation and seven subtypes, using BAPC modelling from the GBD study. (A) The projected prevalence cases and ASR of prevalence of overall amputation and seven subtypes. (B) The projected YLDs cases and ASR of YLDs of overall amputation and seven subtypes. Bars denote the quantity of cases, and the line is used to show the ASR. Solid lines correspond to observed rates spanning from 1990 to 2021, whereas dashed lines signify model projections for the period from 2022 to 2050.

From 2021 to 2050, the BAPC model projects slight global declines in ASR prevalence (from 5330 to 5270; 0.989 times baseline) and YLDs (from 71.1 to 70.9; 0.997 times baseline) (Figure 8A and Table S20). However, trends vary across amputation subtypes (Figure 8B). Temporal validation (predictions for 2016–2021 vs. observed GBD estimates) demonstrated good agreement, with low prediction errors and complete coverage of observed values by predicted 95% credible intervals (Figure S22).

Between 2021 and 2050, subtype YLDs showed limited overall variation, while total amputation YLDs gradually decline. By 2050, AMP‐Th YLDs will decrease from 13.1 to 12.4 (94% of baseline), one of the largest reductions among subtypes. AMP‐F YLDs will decline from 12.2 to 11.6 (95% of baseline), reflecting a more pronounced decrease. YLDs for AMP‐UL1 will decrease from 4.36 to 4.25 (2.5% reduction), AMP‐UL2 from 13.5 to 13.3 (1.5% reduction) and AMP‐LL2 from 7.55 to 7.51 (0.5% reduction). The decline for these three subtypes is limited. YLDs for AMP‐LL1 will increase from 14.6 to 14.8 (1.02 times baseline), representing a substantial rise. YLDs for AMP‐Toe will increase from 5.71 to 5.73, representing a limited rise and a gradual upward trend.

With the ageing population and the prevalence of diabetes, global AMP‐LL1 and AMP‐LL2, along with their associated YLDs, are expected to continue rising. Without effective primary and secondary prevention, amputation is projected to become one of the leading causes of disability among the elderly population in the coming decades.

### 3.7 Leading Injury Causes for Amputation by Type and Site

Among the Level 3 injury causes related to amputation prevalence, leading etiologies for different subtypes exhibited relatively stable overall patterns from 1990 to 2021, with significant changes in certain subtypes. For total amputations, the top three in 2021 were falls (Rank 1), exposure to mechanical forces (Rank 2) and other unintentional injuries (Rank 3) (Figure S23); falls maintained first position since 1990, and road injuries were prominent long‐term. The patterns for AMP‐F, AMP‐Th and AMP‐Toe were relatively similar, primarily driven by exposure to mechanical forces, other unintentional injuries and interpersonal violence (Figures S24, S27 and S28). Site‐specific analyses revealed that AMP‐LL1,2 was mainly attributed to falls, exposure to mechanical forces and road injuries, whereas AMP‐UL1,2 were more strongly associated with conflict and terrorism, interpersonal violence and exposure to mechanical forces (Figures S25, S26, S29 and S30). Notably, conflict and terrorism as well as interpersonal violence rapidly climbed 3–4 ranks for both upper and lower limb amputations between 1990 and 2021, becoming the second leading cause by 2021 and underscoring the rising burden of violence‐related amputations. This trend was accompanied by absolute burden increases: ASR for conflict and terrorism rose from 217.9 to 463.1, and prevalent cases surged by ~230.1% (from ~11.35 million to 37.49 million; Tables S21–28). Temporally, while unintentional injury categories have long dominated amputation prevalence, transport injuries and violence‐related injuries (such as conflict and terrorism and interpersonal violence) exhibited notable fluctuations across periods and amputation subtypes. These findings elucidate the primary epidemiological drivers of external injuries‐related amputations, providing an evidence base for targeted prevention.

## 4 Discussion

This study comprehensively assessed the burden of overall amputation and its seven subtypes within the GBD framework, revealing several key findings. From 1990 to 2021, although the global ASR of prevalence and YLDs showed a declining trend, the absolute number of cases increased substantially due to population growth and ageing, with some regions exhibiting upward trends. Second, three minor amputation subtypes—AMP‐F, AMP‐Th and AMP‐Toe—accounted for 91.7% of all cases, with AMP‐F alone representing approximately half of the global total. Third, while AMP‐F carried the highest prevalence burden, it contributed relatively little to YLDs; in contrast, AMP‐LL1, despite its lower prevalence, ranked first in YLDs contribution (20.8%), followed by AMP‐UL2 (18.9%). Fourth, the overall amputation burden increased with age, generally peaking in the elderly, although subtype‐specific patterns varied markedly, with certain major limb amputations (e.g., in North Africa and the Middle East) peaking earlier in the 50–59 age group. Fifth, high‐income regions such as Australia, Central Europe and Eastern Europe exhibited a significantly higher burden of finger‐related amputations (AMP‐F and AMP‐Th) compared to other regions. Sixth, men bore a greater overall amputation burden, particularly for finger amputations, whereas women experienced a higher burden of lower limb amputations (AMP‐LL1 and AMP‐LL2). Finally, the etiological landscape has shifted notably, with conflict and terrorism rising sharply in global ranking as a driver of amputation (from 8th in 1990 to 4th in 2021), exerting a particularly pronounced impact in North Africa and the Middle East through war‐related trauma.

From 1990 to 2021, the global ASRs of amputation prevalence and YLDs declined, reflecting the impact of advances in public health and preventive interventions, consistent with reports from the International Diabetes Federation and population‐based studies [9, 10]. However, the mechanisms underlying this decline differed substantially across amputation subtypes and regions. Although distal amputations (fingers, thumbs, and toes) accounted for 91.7% of all cases, the overall reduction cannot be explained solely by improved diabetes management. The regional distribution of AMP‐Toe and AMP‐Th closely mirrored that of diabetes‐related complications, with higher burdens in regions with high diabetes prevalence, including Australia and Central and Eastern Europe, likely reflecting the combined effects of diabetic foot disease, obesity and everyday trauma. In contrast, road traffic injuries and armed conflicts appeared to be the principal trauma‐related drivers of lower limb amputations. Consistent with our etiological analyses, falls remained the leading cause across amputation subtypes, whereas road traffic injuries and conflict‐related injuries contributed disproportionately to lower limb amputations. Regions affected by armed conflict, including Burundi, the Syrian Arab Republic, Yemen and Libya, showed increasing ASRs of lower limb amputation, in agreement with previous GBD analyses identifying road traffic injuries as a major cause of lower limb amputation and documenting the rapidly increasing burden of conflict‐related injuries in Yemen [11, 12, 13]. Meanwhile, the declining prevalence of AMP‐F (EAPC = −0.99) and AMP‐Th (EAPC = ‐1.15) is likely attributable to improved occupational safety and advances in microsurgery and digit replantation, whereas the marked reduction in AMP‐UL2 YLDs (EAPC = ‐1.81) may reflect advances in trauma care and rehabilitation [14]. Together, these findings indicate that, despite declining global ASRs, geopolitical conflicts, road safety and health‐system capacity remain major determinants of regional amputation burden, underscoring the importance of targeted prevention strategies and equitable resource allocation.

The high burden of finger‐related amputations in Australia and Central and Eastern Europe is likely driven by different combinations of metabolic, occupational and healthcare factors. In Australia, despite long‐standing diabetes prevention strategies, diabetes remains one of the fastest growing chronic diseases [14, 15]. Aboriginal and Torres Strait Islander populations continue to experience disproportionately high diabetes prevalence because of socioeconomic disadvantage and persistent gaps in multidisciplinary care [16]. Diabetic peripheral neuropathy further increases susceptibility to traumatic finger amputations, contributing to higher AMP‐F rates in Aboriginal populations. In Central and Eastern Europe, persistently high obesity and diabetes prevalence, together with unhealthy lifestyles, delayed treatment, occupational hand injuries and unequal healthcare access, contribute to the elevated burden of finger‐ and toe‐related amputations [17, 18]. Regional differences in YLDs are similarly influenced by chronic disease comorbidities, population ageing and healthcare disparities. Aboriginal Australians experience poorer rehabilitation because of diabetic foot complications and cardiovascular comorbidity, whereas inadequate diabetes management in low‐SDI Eastern Europe and ageing populations in Central Europe contribute to prolonged disability following amputation [19, 20, 21, 22].

The age distribution of amputation showed a spindle‐shaped prevalence curve peaking between 40 and 80 years, although substantial subtype‐specific differences were observed. In most regions, AMP‐LL2, AMP‐UL1 and AMP‐UL2 reached peak prevalence between 70 and 95 years of age, whereas the peak shifted to 55–59 years in North Africa and the Middle East. The predominance of amputation among older adults is largely attributable to the progressive accumulation of chronic degenerative diseases, particularly diabetic vasculopathy and peripheral artery disease (PAD), whose complications increase with age and make older individuals more susceptible to amputation. By contrast, the earlier peak observed in North Africa and the Middle East likely reflects the combined effects of conflict‐related trauma, occupational injuries, earlier diabetes onset and limited healthcare access. Restricted availability of limb‐salvage procedures, including vascular reconstruction, may further accelerate disease progression, resulting in earlier amputation in resource‐limited settings [23].

AMP‐F was the most prevalent amputation subtype throughout the study period. Consistent with our etiological analyses, exposure to mechanical forces remained the second leading cause of AMP‐F over the past three decades, highlighting the major contribution of occupational trauma to its burden. Previous studies have shown that up to 93% of upper limb amputations involve the wrist or fingers, with 80%–90% resulting from traumatic injuries [24]. Young male manual workers are particularly vulnerable because of occupational exposure to industrial machinery and inadequate protective measures. Reports from Shanghai further illustrate the high incidence of work‐related finger amputations, particularly those caused by meat grinders and other industrial equipment [25]. Although advances in microsurgical replantation have substantially improved limb salvage, severe neurovascular injuries still frequently require amputation. These findings support strengthening occupational safety through mandatory machine guarding, appropriate personal protective equipment and systematic safety training to reduce work‐related finger amputations.

AMP‐LL1 contributed the greatest YLD burden, largely reflecting the combined effects of vascular disease, amputation level and chronic disease comorbidities on long‐term functional outcomes. Approximately 70% of lower limb amputations are attributable to vascular disease, with diabetic PAD representing the leading cause [26]. In low‐ and middle‐income countries, particularly in South and Southeast Asia, inadequate diabetes management delays foot care and increases amputation risk; for example, rural Indian patients with diabetic foot ulcers (DFU) have a 3.2‐fold higher risk of amputation without standardized care [27]. Similar patterns are evident in East Asia, where severe vascular disease and limited access to specialized prosthetic rehabilitation contribute to persistent disability following amputation [28]. Above‐knee amputations are associated with poorer prosthetic function, lower walking recovery and more than fourfold higher 1‐year mortality than below‐knee amputations, particularly among patients with cardiovascular comorbidities [29, 30]. These challenges are further exacerbated in resource‐limited settings, where restricted access to vascular reconstruction contributes to poorer functional outcomes, as illustrated by the increasing burden observed in Burundi [31, 32]. Consistent with our age‐specific analyses, the YLD burden of AMP‐LL1 continued to increase with age, peaking among individuals aged ≥ 95 years because of chronic vascular disease, neuropathy and prolonged rehabilitation needs.

Marked sex differences were observed in the global burden of amputation, with men bearing a greater burden of finger and thumb amputations, whereas women experienced a higher burden of lower limb amputations (AMP‐LL1 and AMP‐LL2). The predominance of finger amputations in men is largely attributable to greater occupational exposure to industrial machinery and other high‐risk manual work. Regional studies report that men account for 78%–89% of finger amputations, most resulting from machinery‐related trauma [33, 34]. In contrast, the higher burden of lower limb amputation in women is primarily associated with falls, the leading cause of traumatic amputation among older adults [35]. Older women have substantially higher osteoporosis prevalence (20.7% vs. 11.3% in men), increasing the likelihood that fall‐related fractures or soft‐tissue injuries progress to infection and gangrene in the presence of vascular disease or diabetes [36, 37]. In addition, more rapid progression of PAD and diabetes, together with delayed diagnosis due to atypical presentation, contributes to older age at first amputation (78.9 vs. 68.1 years in men) and a greater proportion of major amputations [38]. These findings support sex‐specific prevention strategies, including occupational safety measures for men and fall prevention, osteoporosis screening and early multidisciplinary management of PAD and diabetes for older women.

In addition, this study demonstrates a substantial shift in the etiological landscape of amputation. Between 1990 and 2021, conflict and terrorism rose from the eighth to the fourth leading global cause of amputation, with a particularly pronounced impact in North Africa and the Middle East, where conflict became the second leading cause of lower limb amputation. A multicenter study of amputations during the Syrian Civil War reported that 89.3% of civilian amputations resulted from explosive events, with 74% involving the lower limbs and 25.4% occurring in children under 18 years of age [39]. Similarly, blast‐related multiple‐limb amputations increased substantially during the conflicts in Iraq and Afghanistan, primarily caused by landmines (61.0%–66.6%), improvised explosive devices or bombs (23.4%–25.9%) and gunshot wounds (7.41%–15.6%) [39]. These findings highlight the growing contribution of conflict‐related injuries to the global amputation burden and support strengthening trauma care, limb‐salvage capacity, rehabilitation services and humanitarian health systems in conflict‐affected regions. Ultimately, sustainable regional and global peace remains the most effective public health strategy for reducing the burden of war‐related amputations and their associated disability.

This study has several limitations. First, as a GBD‐based analysis, the estimates may underestimate the burden of amputation in regions with limited or low‐quality data, particularly in rural areas and conflict settings. Second, YLD estimates rely on standardized disability weights, which may not fully capture real‐world functional outcomes because differences in rehabilitation services, prosthetic access and social support across countries are not considered. Third, the GBD database does not distinguish detailed anatomical levels of lower limb amputation (e.g., foot, below‐knee or above‐knee), limiting more refined analyses of functional impairment and clinical relevance. Fourth, variations in diagnostic practices, uncertainty in modelled estimates and the exclusion of psychological consequences may introduce bias in cross‐country comparisons. Finally, although the BAPC model demonstrated good temporal validation, long‐term projections remain subject to uncertainty, particularly in the presence of future changes in healthcare policies, conflicts or advances in diabetes management.

## 5 Conclusion

Based on GBD 2021 estimates, this study provides a comprehensive assessment of the global burden of amputation‐related disability. Although age‐standardized prevalence and YLD rates declined between 1990 and 2021, absolute case numbers increased substantially, largely driven by population growth and ageing. Distal amputations accounted for most prevalent cases, whereas unilateral lower limb amputation contributed the greatest YLD burden and is projected to remain a major source of disability in the coming decades. The marked heterogeneity across amputation subtypes, regions, age groups and socioeconomic settings highlights the need for targeted prevention, improved rehabilitation and equitable allocation of healthcare resources. Future strategies should emphasize early management of diabetes and vascular disease, occupational injury prevention and trauma care in conflict‐affected settings to reduce the long‐term disability burden associated with amputation.

## Funding

This study was supported by the National Natural Science Foundation of China (Grant No. 82460548) and the Natural Science Foundation of Jiangxi Province (Grant No. 20242BAB20368), both awarded to J.Y.T.

## Ethics Statement

The authors have nothing to report.

## Conflicts of Interest

The authors declare no conflicts of interest.

## Supporting information


**Table S1:** List of International Classification of Diseases (ICD) codes mapped to fractures among Amputations in GBD 2021.
**Table S2:** Cases and ASR of prevalence and YLDs with EAPC for overall amputations, globally and by 21 GBD regions.
**Table S3:** ASR of prevalence and YLDs in 1990 and 2021, and their EAPC from 1990 to 2021 for overall amputations, by country.
**Table S4:** Cases and ASR of prevalence and YLDs in 2021, and their EAPC from 1990 to 2021 for overall amputations, globally and by 21 GBD regions.
**Table S5:** Cases and ASR of prevalence and YLDs in 2021, and their EAPC from 1990 to 2021 for AMP‐F, globally and by 21 GBD regions.
**Table S6:** Cases and ASR of prevalence and YLDs in 2021, and their EAPC from 1990 to 2021 for AMP‐LL1, globally and by 21 GBD regions.
**Table S7:** Cases and ASR of prevalence and YLDs in 2021, and their EAPC from 1990 to 2021 for AMP‐LL2, globally and by 21 GBD regions.
**Table S8:** Cases and ASR of prevalence and YLDs in 2021, and their EAPC from 1990 to 2021 for AMP‐Th, globally and by 21 GBD regions.
**Table S9:** Cases and ASR of prevalence and YLDs in 2021, and their EAPC from 1990 to 2021 for AMP‐Toe, globally and by 21 GBD regions.
**Table S10:** Cases and ASR of prevalence and YLDs in 2021, and their EAPC from 1990 to 2021 for AMP‐UL1, globally and by 21 GBD regions.
**Table S11:** Cases and ASR of prevalence and YLDs in 2021, and their EAPC from 1990 to 2021 for AMP‐UL2, globally and by 21 GBD regions.
**Table S12:** ASR of prevalence and YLDs in 1990 and 2021, and their EAPC from 1990 to 2021 for AMP‐F, by country.
**Table S13:** ASR of prevalence and YLDs in 1990 and 2021, and their EAPC from 1990 to 2021 for AMP‐LL1, by country.
**Table S14:** ASR of prevalence and YLDs in 1990 and 2021, and their EAPC from 1990 to 2021 for AMP‐LL2, by country.
**Table S15:** ASR of prevalence and YLDs in 1990 and 2021, and their EAPC from 1990 to 2021 for AMP‐Th, by country.
**Table S16:** ASR of prevalence and YLDs in 1990 and 2021, and their EAPC from 1990 to 2021 for AMP‐Toe, by country.
**Table S17:** ASR of prevalence and YLDs in 1990 and 2021, and their EAPC from 1990 to 2021 for AMP‐UL1, by country.
**Table S18:** ASR of prevalence and YLDs in 1990 and 2021, and their EAPC from 1990 to 2021 for AMP‐UL2, by country.
**Table S19:** Global cases and ASR of prevalence and YLDs in 2021, and their EAPC from 1990 to 2021 for seven amputation subtypes.
**Table S20:** Projected ASR of prevalence and YLDs from 2022 to 2050 for total amputations.
**Table S21:** Rankings, ASR of prevalence and number of cases of total amputation by injury type (1990–2021), with percentage changes in absolute cases (1990–2021).
**Table S22:** Rankings, ASR of prevalence and number of cases of AMP‐F by injury type (1990–2021), with percentage changes in absolute cases (1990–2021).
**Table S23:** Rankings, ASR of prevalence and number of cases of AMP‐LL2 by injury type (1990–2021), with percentage changes in absolute cases (1990–2021).
**Table S24:** Rankings, ASR of prevalence and number of cases of AMP‐LL1 by injury type (1990–2021), with percentage changes in absolute cases (1990–2021).
**Table S25:** Rankings, ASR of prevalence and number of cases of AMP‐Th by injury type (1990–2021), with percentage changes in absolute cases (1990–2021).
**Table S26:** Rankings, ASR of prevalence and number of cases of AMP‐Toe by injury type (1990–2021), with percentage changes in absolute cases (1990–2021).
**Table S27:** Rankings, ASR of prevalence and number of cases of AMP‐UL2 by injury type (1990–2021), with percentage changes in absolute cases (1990–2021).
**Table S28:** Rankings, ASR of prevalence and number of cases of AMP‐UL1 by injury type (1990–2021), with percentage changes in absolute cases (1990–2021).
**Figure S1:** The ASR and EAPC of Global distribution of the burden for AMP‐F from 1990 to 2021.
**Figure S2:** The ASR and EAPC of Global distribution of the burden for AMP‐LL2 from 1990 to 2021.
**Figure S3:** The ASR and EAPC of Global distribution of the burden for AMP‐LL1 from 1990 to 2021.
**Figure S4:** The ASR and EAPC of Global distribution of the burden for AMP‐Th from 1990 to 2021.
**Figure S5:** The ASR and EAPC of Global distribution of the burden for AMP‐Toe from 1990 to 2021.
**Figure S6:** The ASR and EAPC of Global distribution of the burden for AMP‐UL2 from 1990 to 2021.
**Figure S7:** The ASR and EAPC of Global distribution of the burden for AMP‐UL1 from 1990 to 2021.
**Figure S8:** Gender‐specific burden and YLDs of AMP‐F across 21 GBD regions in 2021.
**Figure S9:** Gender‐specific burden and YLDs of AMP‐LL2 across 21 GBD regions in 2021.
**Figure S10:** Gender‐specific burden and YLDs of AMP‐LL1 across 21 GBD regions in 2021.
**Figure S11:** Gender‐specific burden and YLDs of AMP‐Th across 21 GBD regions in 2021.
**Figure S12:** Gender‐specific burden and YLDs of AMP‐Toe across 21 GBD regions in 2021.
**Figure S13:** Gender‐specific burden and YLDs of AMP‐UL2 across 21 GBD regions in 2021.
**Figure S14:** Gender‐specific burden and YLDs of AMP‐UL1 across 21 GBD regions in 2021.
**Figure S15:** Curves of the correlation between the prevalence of AMP‐F and SDI and YLDs with SDI in 2021.
**Figure S16:** Curves of the correlation between the prevalence of AMP‐LL2 and SDI and YLDs with SDI in 2021.
**Figure S17:** Curves of the correlation between the prevalence of AMP‐LL1 and SDI and YLDs with SDI in 2021.
**Figure S18:** Curves of the correlation between the prevalence of AMP‐Th and SDI and YLDs with SDI in 2021.
**Figure S19:** Curves of the correlation between the prevalence of AMP‐Toe and SDI and YLDs with SDI in 2021.
**Figure S20:** Curves of the correlation between the prevalence of AMP‐UL2 and SDI and YLDs with SDI in 2021.
**Figure S21:** Curves of the correlation between the prevalence of AMP‐UL1 and SDI and YLDs with SDI in 2021.
**Figure S22:** Temporal validation of the Bayesian age–period–cohort (BAPC) prediction model.
**Figure S23:** Ranking of causes of injury leading to amputation worldwide, 1990–2021, and trends in prevalence.
**Figure S24:** Ranking of causes of injury leading to AMP‐F worldwide, 1990–2021, and trends in prevalence.
**Figure S25:** Ranking of causes of injury leading to AMP‐LL2 worldwide, 1990–2021, and trends in prevalence.
**Figure S26:** Ranking of causes of injury leading to AMP‐LL1 worldwide, 1990–2021, and trends in prevalence.
**Figure S27:** Ranking of causes of injury leading to AMP‐Th worldwide, 1990–2021, and trends in prevalence.
**Figure S28:** Ranking of causes of injury leading to AMP‐Toe worldwide, 1990–2021, and trends in prevalence.
**Figure S29:** Ranking of causes of injury leading to AMP‐UL2 worldwide, 1990–2021, and trends in prevalence.
**Figure S30:** Ranking of causes of injury leading to AMP‐UL1 worldwide, 1990–2021, and trends in prevalence.

## Data Availability

The data analysed are publicly provided by the Institute for Health Metrics and Evaluation (http://www.healthdata.org/; http://ghdx.healthdata.org/gbd‐results‐tool).
