# Supplementary material for: Global Epidemiology of Amputation‐Related Disability From 1990 to 2021: Geographic Variation and Projections to 2050
Source: J Cachexia Sarcopenia Muscle. 2026 Aug 7;17(4):e70356. doi: 10.1002/jcsm.70356 (PMC13448777; doi:10.1002/jcsm.70356)
Supplement: Supplementary file 1 [file JCSM-17-e70356-s001.docx]

**Supplementary Appendix**

**Global epidemiology of amputation-related disability from 1990 to 2021: geographic variation and projections to 2050**

Content

[Table S1. List of International Classification of Diseases (ICD) codes mapped to fractures among Amputations in GBD 2021.	5](#_Toc236494934)

[Table S2. Cases and ASR of prevalence and YLDs with EAPC for overall amputations, globally and by 21 GBD regions.	6](#_Toc236494935)

[Table S3. ASR of prevalence and YLDs in 1990 and 2021, and their EAPC from 1990 to 2021 for overall amputations, by country.	10](#_Toc236494936)

[Table S4. Cases and ASR of prevalence and YLDs in 2021, and their EAPC from 1990 to 2021 for overall amputations, globally and by 21 GBD regions.	28](#_Toc236494937)

[Table S5. Cases and ASR of prevalence and YLDs in 2021, and their EAPC from 1990 to 2021 for AMP-F, globally and by 21 GBD regions.	30](#_Toc236494938)

[Table S6. Cases and ASR of prevalence and YLDs in 2021, and their EAPC from 1990 to 2021 for AMP-LL1, globally and by 21 GBD regions.	32](#_Toc236494939)

[Table S7. Cases and ASR of prevalence and YLDs in 2021, and their EAPC from 1990 to 2021 for AMP-LL2, globally and by 21 GBD regions.	34](#_Toc236494940)

[Table S8. Cases and ASR of prevalence and YLDs in 2021, and their EAPC from 1990 to 2021 for AMP-Th, globally and by 21 GBD regions.	36](#_Toc236494941)

[Table S9. Cases and ASR of prevalence and YLDs in 2021, and their EAPC from 1990 to 2021 for AMP-Toe, globally and by 21 GBD regions.	38](#_Toc236494942)

[Table S10. Cases and ASR of prevalence and YLDs in 2021, and their EAPC from 1990 to 2021 for AMP-UL1, globally and by 21 GBD regions.	40](#_Toc236494943)

[Table S11. Cases and ASR of prevalence and YLDs in 2021, and their EAPC from 1990 to 2021 for AMP-UL2, globally and by 21 GBD regions.	42](#_Toc236494944)

[Table S12. ASR of prevalence and YLDs in 1990 and 2021, and their EAPC from 1990 to 2021 for AMP-F, by country.	44](#_Toc236494945)

[Table S13. ASR of prevalence and YLDs in 1990 and 2021, and their EAPC from 1990 to 2021 for AMP-LL1, by country.	62](#_Toc236494946)

[Table S14. ASR of prevalence and YLDs in 1990 and 2021, and their EAPC from 1990 to 2021 for AMP-LL2, by country.	80](#_Toc236494947)

[Table S15. ASR of prevalence and YLDs in 1990 and 2021, and their EAPC from 1990 to 2021 for AMP-Th, by country.	98](#_Toc236494948)

[Table S16. ASR of prevalence and YLDs in 1990 and 2021, and their EAPC from 1990 to 2021 for AMP-Toe, by country.	116](#_Toc236494949)

[Table S17. ASR of prevalence and YLDs in 1990 and 2021, and their EAPC from 1990 to 2021 for AMP-UL1, by country.	134](#_Toc236494950)

[Table S18. ASR of prevalence and YLDs in 1990 and 2021, and their EAPC from 1990 to 2021 for AMP-UL2, by country.	152](#_Toc236494951)

[Table S19. Global cases and ASR of prevalence and YLDs in 2021, and their EAPC from 1990 to 2021 for 7 amputation subtypes.	170](#_Toc236494952)

[Table S20. Projected ASR of prevalence and YLDs from 2022 to 2050 for total amputations.	171](#_Toc236494953)

[Table S21. Rankings, ASR of prevalence, and number of cases of total amputation by injury type (1990–2021), with percentage changes in absolute cases (1990–2021).	173](#_Toc236494954)

[Table S22. Rankings, ASR of prevalence, and number of cases of AMP-F by injury type (1990–2021), with percentage changes in absolute cases (1990–2021).	175](#_Toc236494955)

[Table S23. Rankings, ASR of prevalence, and number of cases of AMP-LL2 by injury type (1990–2021), with percentage changes in absolute cases (1990–2021).	177](#_Toc236494956)

[Table S24. Rankings, ASR of prevalence, and number of cases of AMP-LL1 by injury type (1990–2021), with percentage changes in absolute cases (1990–2021).	179](#_Toc236494957)

[Table S25. Rankings, ASR of prevalence, and number of cases of AMP-Th by injury type (1990–2021), with percentage changes in absolute cases (1990–2021).	181](#_Toc236494958)

[Table S26. Rankings, ASR of prevalence, and number of cases of AMP-Toe by injury type (1990–2021), with percentage changes in absolute cases (1990–2021).	183](#_Toc236494959)

[Table S27. Rankings, ASR of prevalence, and number of cases of AMP-UL2 by injury type (1990–2021), with percentage changes in absolute cases (1990–2021).	185](#_Toc236494960)

[Table S28. Rankings, ASR of prevalence, and number of cases of AMP-UL1 by injury type (1990–2021), with percentage changes in absolute cases (1990–2021).	187](#_Toc236494961)

[Figure S1. The ASR and EAPC of Global distribution of the burden for AMP-F from 1990 to 2021.	189](#_Toc236494962)

[Figure S2. The ASR and EAPC of Global distribution of the burden for AMP-LL2 from 1990 to 2021.	190](#_Toc236494963)

[Figure S3. The ASR and EAPC of Global distribution of the burden for AMP-LL1 from 1990 to 2021.	191](#_Toc236494964)

[Figure S4. The ASR and EAPC of Global distribution of the burden for AMP-Th from 1990 to 2021.	192](#_Toc236494965)

[Figure S5. The ASR and EAPC of Global distribution of the burden for AMP-Toe from 1990 to 2021.	193](#_Toc236494966)

[Figure S6. The ASR and EAPC of Global distribution of the burden for AMP-UL2 from 1990 to 2021.	194](#_Toc236494967)

[Figure S7. The ASR and EAPC of Global distribution of the burden for AMP-UL1 from 1990 to 2021.	195](#_Toc236494968)

[Figure S8. Gender-specific burden and YLDs of AMP-F across 21 GBD regions in 2021.	195](#_Toc236494969)

[Figure S9. Gender-specific burden and YLDs of AMP-LL2 across 21 GBD regions in 2021.	196](#_Toc236494970)

[Figure S10. Gender-specific burden and YLDs of AMP-LL1 across 21 GBD regions in 2021.	196](#_Toc236494971)

[Figure S11. Gender-specific burden and YLDs of AMP-Th across 21 GBD regions in 2021.	196](#_Toc236494972)

[Figure S12. Gender-specific burden and YLDs of AMP-Toe across 21 GBD regions in 2021.	197](#_Toc236494973)

[Figure S13. Gender-specific burden and YLDs of AMP-UL2 across 21 GBD regions in 2021.	197](#_Toc236494974)

[Figure S14. Gender-specific burden and YLDs of AMP-UL1 across 21 GBD regions in 2021.	197](#_Toc236494975)

[Figure S15. Curves of the correlation between the prevalence of AMP-F and SDI, and YLDs with SDI in 2021.	198](#_Toc236494976)

[Figure S16. Curves of the correlation between the prevalence of AMP-LL2 and SDI, and YLDs with SDI in 2021.	199](#_Toc236494977)

[Figure S17. Curves of the correlation between the prevalence of AMP-LL1 and SDI, and YLDs with SDI in 2021.	200](#_Toc236494978)

[Figure S18. Curves of the correlation between the prevalence of AMP-Th and SDI, and YLDs with SDI in 2021.	201](#_Toc236494979)

[Figure S19. Curves of the correlation between the prevalence of AMP-Toe and SDI, and YLDs with SDI in 2021.	202](#_Toc236494980)

[Figure S20. Curves of the correlation between the prevalence of AMP-UL2 and SDI, and YLDs with SDI in 2021.	203](#_Toc236494981)

[Figure S21. Curves of the correlation between the prevalence of AMP-UL1 and SDI, and YLDs with SDI in 2021.	204](#_Toc236494982)

[Figure S22. Temporal validation of the Bayesian age–period–cohort (BAPC) prediction model.	205](#_Toc236494983)

[Figure S23. Ranking of Causes of Injury Leading to Amputation Worldwide, 1990–2021, and Trends in Prevalence.	206](#_Toc236494984)

[Figure S24. Ranking of Causes of Injury Leading to AMP-F Worldwide, 1990–2021, and Trends in Prevalence.	207](#_Toc236494985)

[Figure S25. Ranking of Causes of Injury Leading to AMP-LL2 Worldwide, 1990–2021, and Trends in Prevalence.	208](#_Toc236494986)

[Figure S26. Ranking of Causes of Injury Leading to AMP-LL1 Worldwide, 1990–2021, and Trends in Prevalence.	209](#_Toc236494987)

[Figure S27. Ranking of Causes of Injury Leading to AMP-Th Worldwide, 1990–2021, and Trends in Prevalence.	210](#_Toc236494988)

[Figure S28. Ranking of Causes of Injury Leading to AMP-Toe Worldwide, 1990–2021, and Trends in Prevalence.	211](#_Toc236494989)

[Figure S29. Ranking of Causes of Injury Leading to AMP-UL2 Worldwide, 1990–2021, and Trends in Prevalence.	212](#_Toc236494990)

[Figure S30. Ranking of Causes of Injury Leading to AMP-UL1 Worldwide, 1990–2021, and Trends in Prevalence.	213](#_Toc236494991)

**Table S1. List of International Classification of Diseases (ICD) codes mapped to fractures among Amputations in GBD 2021.**

| **Subtype** | **ICD-9** | **ICD-10** | **Definition** |
| --- | --- | --- | --- |
| Amputations of lower limbs, bilateral | 896.2, 896.3, 897.6, 897.7 | - | Loss of part of both legs, leaving pain and tingling in the stumps; may include use of prosthetic limbs. |
| Amputations of upper limbs, bilateral | 887.6, 887.7, 888.1, 888.9 | 568.4 | Loss of part of both arms, leaving pain ang tingling in the stumps; may include use of prosthetic limbs. |
| Amputations of fingers (excluding thumb) | 886.0, 886.1 | 568.1, 568.6 | Loss of one or more fingers (excluding thumb) of a single hand. |
| Amputations of lower limb, unilateral | 896.0, 896.1, 897.0, 897.1, 897.2, 897.3, 897.4, 897.5 | 578.0, 578.1, 578.9, 588.9, 598.0, 598.3, 598.9 | Loss of one or more leg; may include use of a prosthetic leg. |
| Amputations of upper limb, unilateral | 887.0, 887.1, 887.2, 887.3, 887.4, 887.5 | 548.9, 558.1 | Loss of part of one arm; may include use of a prosthetic arm. |
| Amputations of thumb | 885.0, 885.1 | 568.0 | Loss of the thumb of one hand. |
| Amputations of toe/toes | 895.0, 895.1 | 598.1 | Loss of one or more toes, leaving occasional pain and tingling in the stump. |

**Table S2. Cases and ASR of prevalence and YLDs with EAPC for overall amputations, globally and by 21 GBD regions.**

| **Characteristics** | **1990** | | **2021** | | **1990** | | **2021** | | **1990-2021 EAPC No. (95% CI)** |
| --- | --- | --- | --- | --- | --- | --- | --- | --- | --- |
|  | **Prevalence Cases No. (95% UI)** | **ASR per 100,000 No. (95% UI)** | **Prevalence Cases No. (95% UI)** | **ASR per 100,000 No. (95% UI)** | **YLDs cases No. (95% UI)** | **ASR per 100,000 No. (95% UI)** | **YLDs cases No. (95% UI)** | **ASR per 100,000 No. (95% UI)** |  |
| Global | 338098873.3 (310644849.6 to 370806951.8) | 7045.9 (6496.2 to 7691.2) | 445237916.8 (408967911.0 to 485841239.1) | 5333.4 (4894.6 to 5818.4) | 4988017.4 (3481526.2 to 7331752.0) | 104.1 (72.7 to 153.3) | 5935830.8 (3987841.5 to 9066590.3) | 71.1 (47.8 to 108.6) | -1.4 (-1.5 to -1.3) |
| Andean Latin America | 2463620.4 (2246473.5 to 2732108.6) | 7987.9 (7378.6 to 8709.5) | 4526483.8 (4155418.8 to 4955015.5) | 6922.7 (6369.2 to 7567.9) | 38565.4 (27278.5 to 55481.4) | 125.4 (90.3 to 179.6) | 54189.9 (35713.7 to 85293.2) | 83.4 (55.1 to 130.8) | -1.4 (-1.5 to -1.3) |
| Australasia | 3482735.6 (3115546.1 to 3928397.6) | 15957.5 (14242.8 to 18047.9) | 4834466.2 (4326706.6 to 5434481.7) | 13110.4 (11638.3 to 14826.7) | 29827.9 (15045.0 to 56247.4) | 136.9 (68.9 to 258.1) | 41599.5 (20842.6 to 77699.3) | 113.4 (56.3 to 213.0) | -0.6 (-0.7 to -0.5) |
| Caribbean | 2304147.8 (2133543.3 to 2511839.9) | 7305.0 (6775.0 to 7939.9) | 4061195.4 (3750913.1 to 4420157.1) | 8027.0 (7399.9 to 8740.6) | 30684.0 (21229.7 to 45361.9) | 98.6 (68.6 to 145.1) | 56143.8 (38561.6 to 83779.5) | 111.8 (77.0 to 166.8) | 0.7 (0.5 to 0.9) |
| Central Asia | 6798586.8 (6248985.7 to 7427788.6) | 11407.3 (10538.0 to 12412.7) | 8818166.7 (8153838.8 to 9665919.4) | 9251.7 (8559.5 to 10132.1) | 87937.6 (60055.5 to 132407.7) | 149.5 (102.8 to 223.8) | 105471.9 (69116.7 to 165101.3) | 111.1 (73.0 to 173.6) | -1.2 (-1.3 to -1.0) |
| Central Europe | 28661985.9 (26343271.1 to 31356493.7) | 20939.1 (19215.2 to 22957.5) | 24195518.9 (22311758.1 to 26546009.5) | 16129.1 (14704.0 to 17802.5) | 323534.8 (208638.2 to 515033.4) | 235.6 (151.7 to 376.1) | 221534.9 (120161.7 to 395495.3) | 145.3 (78.6 to 262.7) | -1.6 (-1.7 to -1.6) |
| Central Latin America | 16704573.8 (15044405.7 to 18707572.3) | 12520.4 (11358.6 to 13913.4) | 22814633.7 (20713182.4 to 25323872.0) | 8699.4 (7896.9 to 9656.8) | 252602.2 (176191.2 to 363619.2) | 190.9 (135.3 to 273.3) | 284696.1 (189197.6 to 438031.3) | 108.7 (72.3 to 167.0) | -1.2 (-1.5 to -1.0) |
| Central Sub-Saharan Africa | 1854712.8 (1648031.8 to 2116791.6) | 4427.4 (4014.7 to 4913.7) | 4567871.6 (3859682.2 to 5589867.6) | 4518.0 (3836.6 to 5499.1) | 38968.2 (27205.9 to 54180.8) | 95.7 (68.8 to 130.8) | 94589.1 (64190.0 to 138734.0) | 98.9 (67.8 to 143.2) | 0.1 (-0.3 to 0.4) |
| East Asia | 44869937.2 (40642147.3 to 50337616.8) | 3940.7 (3587.9 to 4389.7) | 67248501.5 (60889834.6 to 74988845.7) | 3645.0 (3291.1 to 4100.4) | 850943.3 (607724.3 to 1167517.5) | 77.6 (55.7 to 105.6) | 819571.7 (499411.6 to 1320842.9) | 43.7 (26.5 to 70.4) | -2.4 (-2.7 to -2.1) |
| Eastern Europe | 44667147.4 (41089988.4 to 48724285.6) | 17740.5 (16301.1 to 19361.3) | 33534302.3 (30874286.0 to 36825645.3) | 12592.1 (11606.7 to 13833.9) | 542414.9 (354330.8 to 841709.8) | 213.6 (139.1 to 332.1) | 354036.8 (213524.0 to 588421.3) | 130.9 (78.3 to 218.9) | -2.0 (-2.4 to -1.7) |
| Eastern Sub-Saharan Africa | 7733995.5 (6565036.1 to 9566555.1) | 5335.7 (4682.9 to 6321.3) | 14680600.9 (12496276.0 to 18077377.2) | 4775.4 (4058.3 to 5926.3) | 176018.5 (116654.4 to 267480.7) | 120.4 (83.2 to 177.5) | 308216.7 (204116.3 to 471182.8) | 107.3 (71.9 to 163.0) | -0.6 (-0.9 to -0.3) |
| High-income Asia Pacific | 14544121.9 (13318650.2 to 15965856.1) | 7497.7 (6868.5 to 8238.8) | 13892083.5 (12756641.3 to 15213275.6) | 5435.1 (4966.2 to 6022.5) | 142601.9 (79457.5 to 247047.4) | 73.7 (41.1 to 127.9) | 127325.1 (68067.2 to 223782.0) | 50.1 (26.4 to 89.3) | -1.4 (-1.4 to -1.3) |
| High-income North America | 19676654.1 (18105584.5 to 21679605.4) | 6345.0 (5827.5 to 7002.6) | 18780323.3 (17391281.3 to 20367218.2) | 3989.6 (3699.3 to 4344.6) | 187212.1 (101995.2 to 330566.4) | 60.4 (32.8 to 106.9) | 183325.3 (102588.2 to 314518.0) | 38.8 (21.5 to 66.9) | -1.6 (-1.9 to -1.3) |
| North Africa and Middle East | 24092833.3 (21582845.0 to 27695942.6) | 8988.8 (8148.9 to 10163.1) | 48250179.0 (42179140.2 to 56635202.8) | 8034.7 (7058.7 to 9380.0) | 445323.7 (300707.0 to 689927.1) | 163.3 (112.4 to 250.4) | 719935.7 (471909.8 to 1106706.8) | 120.3 (79.0 to 185.0) | -1.1 (-1.1 to -1.0) |
| Oceania | 160249.4 (147711.4 to 174859.4) | 3297.6 (3068.6 to 3566.5) | 407527.8 (369139.3 to 455428.6) | 3542.4 (3224.1 to 3921.5) | 3008.8 (2190.6 to 4073.0) | 65.6 (48.1 to 87.8) | 7816.6 (5681.1 to 10609.5) | 71.2 (52.3 to 95.4) | 0.3 (0.2 to 0.4) |
| South Asia | 41490025.9 (37628778.6 to 46287119.5) | 4813.5 (4396.1 to 5317.6) | 68907502.2 (62351184.5 to 77093433.8) | 3900.7 (3545.3 to 4344.6) | 769349.5 (567836.4 to 1037412.5) | 95.3 (70.9 to 126.0) | 1180048.7 (847883.3 to 1638007.1) | 69.2 (50.0 to 96.3) | -1.2 (-1.3 to -1.1) |
| Southeast Asia | 21810390.0 (19545041.6 to 26223605.5) | 5638.8 (5130.2 to 6475.7) | 32246948.3 (29274253.2 to 36387942.8) | 4424.3 (4025.8 to 4962.3) | 384349.9 (263211.3 to 586652.4) | 100.4 (70.5 to 148.8) | 491802.3 (343590.0 to 741454.3) | 68.0 (47.5 to 101.4) | -1.3 (-1.4 to -1.2) |
| Southern Latin America | 5354981.6 (4821502.0 to 5987591.7) | 11150.7 (10044.7 to 12453.4) | 7726464.4 (6997868.8 to 8609801.8) | 10187.1 (9182.3 to 11381.7) | 53577.4 (31539.8 to 90894.8) | 111.5 (65.7 to 189.1) | 70348.0 (38972.1 to 126367.4) | 93.0 (51.2 to 167.3) | -0.5 (-0.7 to -0.4) |
| Southern Sub-Saharan Africa | 2295482.4 (2105772.0 to 2548226.2) | 5627.3 (5226.0 to 6096.4) | 2981298.7 (2761355.2 to 3233578.3) | 3976.5 (3698.2 to 4303.4) | 42922.2 (30890.3 to 60017.0) | 109.8 (80.4 to 151.4) | 50709.3 (36104.9 to 71253.0) | 69.2 (49.6 to 96.4) | -1.7 (-1.9 to -1.5) |
| Tropical Latin America | 11582285.5 (10395716.5 to 13003407.7) | 8935.3 (8041.2 to 9954.0) | 17666174.8 (16007599.9 to 19672543.0) | 7039.2 (6369.7 to 7850.0) | 176975.3 (124896.8 to 252611.5) | 141.2 (100.7 to 198.1) | 237158.7 (159964.8 to 354954.8) | 94.3 (63.5 to 141.3) | -1.5 (-1.6 to -1.4) |
| Western Europe | 31940698.3 (28990258.6 to 35418570.3) | 7150.4 (6452.4 to 7963.4) | 32070877.1 (28855355.4 to 36152850.7) | 5759.5 (5138.6 to 6600.3) | 316097.2 (176936.5 to 552052.7) | 70.8 (39.4 to 124.6) | 308368.5 (166821.9 to 547797.6) | 55.6 (29.7 to 99.4) | -0.8 (-0.8 to -0.7) |
| Western Sub-Saharan Africa | 5609708.0 (5112557.3 to 6045439.3) | 3914.4 (3619.0 to 4188.4) | 13026796.8 (11739374.5 to 14395265.2) | 3572.3 (3278.6 to 3904.9) | 95102.6 (69806.3 to 131220.0) | 70.1 (52.0 to 95.0) | 218942.6 (156980.9 to 303605.8) | 63.7 (46.5 to 86.9) | -0.4 (-0.5 to -0.3) |

**Table S3.** **ASR of prevalence and YLDs in 1990 and 2021, and their EAPC from 1990 to 2021 for overall amputations, by country.**

| **Countries** | **Prevalence** | | | **YLDs** | | |
| --- | --- | --- | --- | --- | --- | --- |
|  | **Age-standardized prevalence rate, 1990** | **Age-standardized prevalence rate, 2021** | **EAPC of prevalence rate No. (95% CI)** | **Age-standardized YLDs rate, 1990** | **Age-standardized YLDs rate, 2021** | **EAPC of YLDs rate No. (95% CI)** |
| Afghanistan | 21693.1 (10879.4 to 42291.6) | 20804.3 (13155.6 to 32892.7) | -0.3 (-0.4 to -0.2) | 829.6 (259.8 to 2256.1) | 620.5 (303.9 to 1274.8) | -1.2 (-1.3 to -1.1) |
| Albania | 26696.3 (24232.9 to 29378.2) | 20596.1 (18827.8 to 22760.6) | -1.3 (-1.4 to -1.1) | 292.7 (188.7 to 465.8) | 183.5 (98.1 to 331.5) | -2.0 (-2.1 to -1.8) |
| Algeria | 7666.0 (7185.0 to 8258.3) | 5768.5 (5314.3 to 6307.2) | -0.9 (-1.0 to -0.8) | 120.7 (85.2 to 172.8) | 72.4 (47.3 to 111.7) | -1.7 (-1.8 to -1.6) |
| American Samoa | 4216.4 (3917.9 to 4572.0) | 3795.4 (3476.3 to 4190.5) | -0.2 (-0.3 to -0.1) | 66.0 (46.6 to 94.3) | 60.6 (42.7 to 86.7) | 0.0 (-0.1 to 0.2) |
| Andorra | 6910.1 (6138.6 to 7873.1) | 6453.8 (5712.7 to 7385.1) | -0.2 (-0.3 to -0.1) | 68.1 (36.7 to 121.3) | 63.8 (34.9 to 113.2) | -0.2 (-0.3 to -0.1) |
| Angola | 7727.8 (6216.4 to 9881.4) | 6227.3 (4839.1 to 8285.3) | -0.8 (-1.1 to -0.6) | 203.5 (135.2 to 306.0) | 143.8 (93.2 to 219.9) | -1.3 (-1.6 to -0.9) |
| Antigua and Barbuda | 9962.1 (9103.7 to 10969.7) | 10871.4 (9832.2 to 12053.9) | 0.2 (0.0 to 0.4) | 110.8 (71.6 to 175.6) | 105.7 (64.1 to 176.9) | -0.2 (-0.4 to 0.0) |
| Argentina | 11313.6 (10167.1 to 12735.9) | 9849.7 (8854.7 to 11001.8) | -0.4 (-0.7 to -0.1) | 113.3 (66.9 to 193.7) | 92.1 (51.6 to 162.4) | -0.6 (-0.9 to -0.4) |
| Armenia | 16016.2 (14744.5 to 17694.9) | 10364.9 (9428.2 to 11540.8) | -1.7 (-1.8 to -1.5) | 207.2 (138.6 to 319.9) | 121.3 (76.5 to 198.1) | -2.3 (-2.5 to -2.1) |
| Australia | 15586.2 (13868.6 to 17628.2) | 12692.3 (11241.2 to 14402.7) | -0.6 (-0.7 to -0.5) | 133.2 (66.6 to 252.6) | 109.8 (54.5 to 206.1) | -0.6 (-0.6 to -0.5) |
| Austria | 9005.6 (8145.9 to 10052.2) | 6436.9 (5783.5 to 7336.8) | -1.1 (-1.1 to -1.1) | 86.6 (47.5 to 153.5) | 62.2 (33.4 to 111.9) | -1.0 (-1.1 to -1.0) |
| Azerbaijan | 9679.2 (8901.2 to 10616.9) | 8965.2 (8163.1 to 9888.0) | -0.4 (-0.5 to -0.3) | 130.2 (89.8 to 193.8) | 105.9 (68.6 to 167.1) | -1.1 (-1.2 to -1.0) |
| Bahamas | 5773.1 (5339.8 to 6330.6) | 6038.3 (5552.4 to 6592.7) | 0.2 (0.0 to 0.3) | 81.3 (56.9 to 119.8) | 76.5 (51.3 to 116.2) | -0.2 (-0.3 to -0.2) |
| Bahrain | 5755.5 (5352.8 to 6226.2) | 5309.5 (4874.6 to 5835.1) | -0.4 (-0.6 to -0.3) | 82.6 (57.5 to 121.3) | 53.2 (30.6 to 90.1) | -2.1 (-2.4 to -1.8) |
| Bangladesh | 3051.7 (2827.0 to 3334.5) | 3182.8 (2848.7 to 3569.7) | -0.1 (-0.3 to 0.0) | 57.5 (42.5 to 77.4) | 47.4 (33.1 to 69.3) | -1.1 (-1.3 to -0.9) |
| Barbados | 5983.6 (5495.4 to 6610.3) | 6036.5 (5538.8 to 6662.5) | -0.1 (-0.3 to 0.1) | 72.6 (48.4 to 111.4) | 63.5 (38.6 to 104.0) | -0.5 (-0.7 to -0.4) |
| Belarus | 14428.4 (13205.3 to 15815.7) | 12426.4 (11301.2 to 13701.2) | -0.6 (-0.8 to -0.3) | 172.1 (112.1 to 268.6) | 118.4 (65.5 to 203.8) | -1.4 (-1.7 to -1.1) |
| Belgium | 7482.1 (6713.9 to 8523.8) | 6474.1 (5769.8 to 7396.1) | -0.2 (-0.8 to 0.3) | 74.2 (40.1 to 130.6) | 63.2 (34.1 to 112.8) | -0.3 (-0.8 to 0.3) |
| Belize | 7746.5 (7092.8 to 8497.4) | 8679.7 (7965.0 to 9501.3) | 0.2 (-0.1 to 0.4) | 102.5 (71.2 to 151.9) | 107.1 (72.8 to 164.0) | -0.2 (-0.4 to 0.1) |
| Benin | 3946.7 (3647.5 to 4214.7) | 3514.1 (3263.0 to 3773.4) | -0.4 (-0.4 to -0.3) | 69.6 (51.5 to 94.7) | 58.6 (42.6 to 81.4) | -0.6 (-0.7 to -0.5) |
| Bermuda | 6428.2 (5898.3 to 7091.0) | 6373.5 (5855.5 to 7018.9) | 0.0 (-0.1 to 0.1) | 71.7 (45.4 to 115.3) | 55.5 (28.8 to 99.9) | -0.9 (-1.0 to -0.9) |
| Bhutan | 4731.9 (4390.3 to 5104.3) | 4915.1 (4551.4 to 5358.8) | 0.1 (0.0 to 0.2) | 76.9 (56.1 to 106.0) | 68.3 (47.2 to 99.6) | -0.4 (-0.6 to -0.3) |
| Bolivia (Plurinational State of) | 7428.4 (6932.7 to 8018.9) | 6186.5 (5748.3 to 6707.9) | -0.6 (-0.7 to -0.6) | 121.0 (88.4 to 169.1) | 84.3 (58.4 to 123.3) | -1.2 (-1.3 to -1.2) |
| Bosnia and Herzegovina | 24945.4 (22636.9 to 27643.6) | 18468.4 (16561.0 to 20693.3) | -1.3 (-1.5 to -1.1) | 269.8 (171.8 to 441.1) | 188.8 (107.9 to 323.4) | -1.9 (-2.2 to -1.6) |
| Botswana | 4052.2 (3770.4 to 4336.3) | 4031.6 (3762.9 to 4302.3) | -0.2 (-0.3 to 0.0) | 69.7 (50.5 to 96.9) | 66.9 (48.6 to 93.4) | -0.3 (-0.6 to -0.1) |
| Brazil | 8968.7 (8069.2 to 9991.4) | 7053.3 (6381.5 to 7875.4) | -1.1 (-1.3 to -0.9) | 142.1 (101.5 to 199.2) | 94.7 (63.8 to 141.8) | -1.5 (-1.6 to -1.4) |
| Brunei Darussalam | 7774.8 (7136.7 to 8527.1) | 6435.7 (5875.1 to 7126.0) | -0.6 (-0.7 to -0.6) | 91.7 (59.5 to 145.5) | 66.2 (38.8 to 110.7) | -1.1 (-1.1 to -1.0) |
| Bulgaria | 22073.4 (20274.6 to 24125.6) | 18840.2 (17243.3 to 20789.6) | -0.5 (-0.5 to -0.4) | 241.4 (152.0 to 390.8) | 179.7 (103.5 to 314.7) | -0.9 (-1.0 to -0.9) |
| Burkina Faso | 3631.9 (3353.3 to 3898.8) | 3547.2 (3266.8 to 3817.4) | -0.1 (-0.2 to -0.1) | 69.4 (51.5 to 92.9) | 66.7 (49.0 to 90.1) | -0.2 (-0.3 to -0.1) |
| Burundi | 3972.9 (3701.2 to 4249.1) | 11550.6 (8372.3 to 16094.5) | 3.7 (2.7 to 4.8) | 73.1 (53.9 to 98.9) | 368.6 (224.6 to 579.7) | 5.3 (3.7 to 7.0) |
| Cabo Verde | 4171.7 (3858.6 to 4461.8) | 3682.9 (3400.6 to 3988.0) | -0.4 (-0.4 to -0.3) | 66.7 (47.4 to 94.9) | 51.6 (35.5 to 76.3) | -0.9 (-1.0 to -0.8) |
| Cambodia | 9373.3 (6170.7 to 16544.9) | 6664.3 (4960.9 to 10417.2) | -1.0 (-1.1 to -0.8) | 285.6 (129.9 to 654.7) | 149.4 (82.3 to 296.1) | -2.2 (-2.3 to -2.0) |
| Cameroon | 3487.8 (3227.0 to 3768.9) | 3456.7 (3158.6 to 3790.0) | -0.1 (-0.1 to 0.0) | 63.4 (46.9 to 85.0) | 61.0 (44.1 to 84.3) | -0.3 (-0.3 to -0.2) |
| Canada | 5236.9 (4823.0 to 5774.6) | 3940.8 (3613.4 to 4337.9) | -0.9 (-1.0 to -0.9) | 49.5 (26.1 to 88.8) | 37.6 (20.2 to 65.7) | -0.9 (-0.9 to -0.9) |
| Central African Republic | 3841.7 (3589.9 to 4073.3) | 4963.8 (4312.4 to 5899.0) | 0.9 (0.7 to 1.0) | 80.0 (59.6 to 104.9) | 124.4 (88.2 to 172.7) | 1.5 (1.3 to 1.8) |
| Chad | 5375.4 (4588.0 to 6499.5) | 5313.8 (4567.8 to 6362.3) | 0.0 (-0.1 to 0.1) | 123.2 (84.4 to 178.5) | 112.5 (76.6 to 161.3) | -0.3 (-0.5 to -0.2) |
| Chile | 9520.9 (8634.3 to 10538.3) | 10711.5 (9626.2 to 12007.5) | 0.6 (0.4 to 0.8) | 98.3 (58.6 to 164.2) | 93.2 (48.0 to 173.6) | 0.0 (-0.2 to 0.1) |
| China | 3966.2 (3610.0 to 4421.5) | 3695.9 (3335.6 to 4161.0) | -0.5 (-0.8 to -0.3) | 78.4 (56.2 to 106.7) | 44.1 (26.7 to 71.2) | -2.4 (-2.7 to -2.1) |
| Colombia | 12318.7 (11289.9 to 13532.9) | 8402.1 (7524.0 to 9413.8) | -1.1 (-1.2 to -1.0) | 178.8 (125.5 to 255.9) | 97.7 (61.9 to 155.1) | -1.9 (-1.9 to -1.8) |
| Comoros | 4077.4 (3800.9 to 4353.1) | 3632.4 (3361.7 to 3913.2) | -0.5 (-0.6 to -0.4) | 72.9 (53.6 to 99.2) | 62.7 (45.3 to 86.9) | -0.7 (-0.8 to -0.6) |
| Congo | 3602.8 (3371.1 to 3830.7) | 4433.0 (3698.1 to 5504.5) | 0.8 (0.2 to 1.3) | 72.0 (53.7 to 95.6) | 102.6 (68.2 to 157.9) | 1.2 (0.3 to 2.2) |
| Cook Islands | 3343.8 (3096.4 to 3642.2) | 3265.9 (2946.8 to 3645.4) | -0.1 (-0.2 to 0.1) | 56.7 (40.3 to 80.0) | 41.6 (26.5 to 65.4) | -1.0 (-1.1 to -0.8) |
| Costa Rica | 8197.0 (7333.7 to 9200.8) | 7316.7 (6549.5 to 8231.9) | -0.4 (-0.5 to -0.3) | 99.3 (65.7 to 154.4) | 75.7 (44.4 to 126.3) | -0.9 (-1.0 to -0.8) |
| Coted'Ivoire | 3917.8 (3627.0 to 4194.1) | 3654.8 (3375.1 to 3954.3) | -0.2 (-0.3 to -0.2) | 69.4 (51.3 to 94.5) | 62.0 (45.4 to 85.7) | -0.4 (-0.5 to -0.3) |
| Croatia | 20104.7 (18410.4 to 22033.9) | 15133.8 (13744.8 to 16723.9) | -0.9 (-1.0 to -0.9) | 197.6 (116.2 to 340.6) | 139.3 (74.5 to 249.5) | -1.3 (-1.4 to -1.2) |
| Cuba | 7368.5 (6832.4 to 8005.9) | 7714.0 (7076.5 to 8455.8) | 0.2 (0.1 to 0.4) | 90.4 (58.7 to 139.5) | 77.4 (45.1 to 130.4) | -0.4 (-0.5 to -0.3) |
| Cyprus | 7628.5 (6933.8 to 8507.6) | 6256.1 (5603.5 to 7127.2) | -0.7 (-0.8 to -0.5) | 78.8 (45.8 to 134.5) | 60.1 (31.7 to 108.1) | -0.9 (-1.0 to -0.8) |
| Czechia | 24025.6 (21959.0 to 26386.1) | 17489.8 (15846.3 to 19365.0) | -0.6 (-0.9 to -0.4) | 248.3 (152.5 to 412.7) | 151.3 (79.0 to 277.8) | -1.1 (-1.3 to -0.9) |
| Democratic People's Republic of Korea | 2724.9 (2543.0 to 2969.3) | 2304.0 (2171.2 to 2465.5) | -0.5 (-0.5 to -0.4) | 57.8 (41.8 to 78.5) | 41.3 (29.2 to 59.1) | -1.1 (-1.1 to -1.1) |
| Democratic Republic of the Congo | 3658.0 (3405.0 to 3915.9) | 3998.0 (3478.3 to 4703.1) | 0.3 (0.1 to 0.6) | 70.2 (52.2 to 94.2) | 84.3 (59.1 to 121.0) | 0.6 (0.2 to 1.0) |
| Denmark | 6573.5 (5942.5 to 7378.1) | 5733.5 (5086.9 to 6596.4) | -0.4 (-0.5 to -0.3) | 65.5 (36.3 to 115.0) | 54.6 (28.3 to 97.9) | -0.5 (-0.6 to -0.4) |
| Djibouti | 4056.1 (3770.8 to 4346.6) | 3986.0 (3643.0 to 4383.9) | -0.3 (-0.4 to -0.1) | 70.6 (51.8 to 96.4) | 67.6 (48.8 to 95.1) | -0.5 (-0.8 to -0.3) |
| Dominica | 5605.3 (5202.2 to 6160.3) | 6194.3 (5740.1 to 6783.0) | 0.3 (0.2 to 0.5) | 78.4 (54.7 to 115.4) | 80.0 (54.3 to 119.8) | 0.1 (0.0 to 0.1) |
| Dominican Republic | 6024.1 (5575.6 to 6531.5) | 7023.1 (6503.5 to 7638.1) | 0.6 (0.4 to 0.8) | 88.2 (62.9 to 125.5) | 88.9 (60.1 to 134.9) | 0.2 (0.0 to 0.3) |
| Ecuador | 7531.5 (7015.5 to 8138.3) | 6685.0 (6204.2 to 7315.1) | -0.4 (-0.5 to -0.3) | 115.8 (82.7 to 163.2) | 84.8 (57.4 to 129.4) | -1.0 (-1.1 to -0.8) |
| Egypt | 6436.0 (5973.4 to 6951.0) | 4916.7 (4498.4 to 5485.7) | -0.8 (-0.9 to -0.8) | 109.0 (78.3 to 150.9) | 69.8 (46.8 to 105.2) | -1.3 (-1.4 to -1.2) |
| El Salvador | 15529.2 (12368.3 to 20085.5) | 10815.9 (9025.8 to 13362.6) | -1.3 (-1.3 to -1.2) | 397.6 (252.3 to 619.3) | 181.2 (115.9 to 284.0) | -2.6 (-2.7 to -2.6) |
| Equatorial Guinea | 3936.9 (3676.0 to 4172.7) | 2762.9 (2566.5 to 2987.5) | -1.2 (-1.2 to -1.2) | 77.2 (57.4 to 102.1) | 43.5 (31.0 to 62.3) | -2.0 (-2.1 to -1.9) |
| Eritrea | 17498.4 (12090.3 to 25042.9) | 12167.4 (8468.4 to 18295.9) | -1.4 (-1.5 to -1.2) | 693.9 (400.1 to 1139.1) | 398.7 (226.9 to 681.2) | -2.0 (-2.2 to -1.8) |
| Estonia | 17452.6 (16016.2 to 19049.6) | 11449.5 (10463.5 to 12576.0) | -1.7 (-1.8 to -1.6) | 206.1 (132.6 to 325.9) | 103.8 (55.0 to 185.3) | -2.7 (-2.9 to -2.5) |
| Eswatini | 4278.0 (4001.3 to 4539.3) | 4014.3 (3764.8 to 4258.2) | -0.2 (-0.3 to -0.1) | 82.6 (60.0 to 111.5) | 72.7 (53.1 to 97.7) | -0.4 (-0.6 to -0.2) |
| Ethiopia | 5983.0 (5446.1 to 6687.1) | 4664.6 (3962.9 to 6069.6) | -1.0 (-1.2 to -0.9) | 130.5 (94.7 to 177.9) | 90.7 (60.5 to 143.9) | -1.5 (-1.7 to -1.2) |
| Fiji | 3182.7 (2964.7 to 3458.5) | 2747.1 (2539.7 to 3015.4) | -0.5 (-0.6 to -0.5) | 55.2 (40.4 to 75.7) | 46.2 (33.4 to 64.4) | -0.6 (-0.7 to -0.6) |
| Finland | 8678.4 (7710.8 to 9866.9) | 7434.8 (6560.2 to 8632.1) | -0.6 (-1.2 to -0.1) | 89.7 (51.0 to 154.8) | 72.7 (38.3 to 129.3) | -0.7 (-1.2 to -0.2) |
| France | 7723.5 (6996.7 to 8587.8) | 6369.0 (5688.0 to 7263.1) | -0.5 (-0.6 to -0.4) | 78.2 (44.3 to 135.9) | 61.8 (33.4 to 110.6) | -0.6 (-0.7 to -0.5) |
| Gabon | 3889.2 (3614.8 to 4156.3) | 3142.3 (2926.0 to 3379.7) | -0.7 (-0.7 to -0.6) | 71.1 (52.0 to 96.0) | 51.3 (37.2 to 71.9) | -1.1 (-1.1 to -1.0) |
| Gambia | 4051.3 (3670.4 to 4458.9) | 3383.8 (3106.4 to 3689.3) | -0.7 (-0.8 to -0.6) | 73.1 (52.2 to 99.6) | 56.7 (40.7 to 77.9) | -0.9 (-1.0 to -0.8) |
| Georgia | 12920.0 (11815.1 to 14193.6) | 13774.2 (12664.9 to 15065.3) | 0.4 (0.3 to 0.5) | 150.8 (97.7 to 236.3) | 153.2 (97.7 to 246.9) | 0.3 (0.1 to 0.4) |
| Germany | 6423.8 (5774.8 to 7262.7) | 5750.3 (5134.3 to 6588.3) | -0.4 (-0.5 to -0.3) | 65.6 (36.4 to 114.5) | 55.3 (29.6 to 98.9) | -0.5 (-0.6 to -0.4) |
| Ghana | 3265.5 (3017.5 to 3503.2) | 3039.4 (2810.0 to 3290.7) | -0.2 (-0.3 to -0.1) | 57.4 (41.7 to 78.8) | 49.6 (35.4 to 69.5) | -0.4 (-0.5 to -0.3) |
| Greece | 7079.0 (6383.2 to 7921.8) | 5908.7 (5310.8 to 6682.1) | -0.6 (-0.7 to -0.5) | 69.3 (37.8 to 123.0) | 56.2 (29.6 to 101.1) | -0.7 (-0.8 to -0.6) |
| Greenland | 7235.5 (6651.9 to 7936.4) | 5186.8 (4749.0 to 5726.5) | -1.3 (-1.4 to -1.2) | 91.4 (61.1 to 142.0) | 57.1 (34.7 to 93.4) | -1.8 (-1.9 to -1.7) |
| Grenada | 8877.8 (7984.5 to 9949.8) | 8911.0 (8128.9 to 9896.9) | 0.1 (-0.1 to 0.2) | 140.4 (94.0 to 210.5) | 107.9 (70.9 to 172.0) | -0.7 (-0.8 to -0.7) |
| Guam | 3637.5 (3361.6 to 3966.0) | 2994.0 (2745.9 to 3306.2) | -0.7 (-0.7 to -0.6) | 52.0 (35.5 to 76.5) | 42.9 (28.6 to 63.9) | -0.6 (-0.7 to -0.5) |
| Guatemala | 13365.8 (11439.8 to 16347.4) | 12787.7 (11366.4 to 14589.7) | -0.1 (-0.1 to -0.1) | 282.5 (184.8 to 430.2) | 186.5 (126.4 to 282.3) | -1.4 (-1.4 to -1.3) |
| Guinea | 4068.0 (3750.9 to 4351.6) | 3902.5 (3608.2 to 4184.1) | -0.1 (-0.1 to 0.0) | 72.1 (53.1 to 98.2) | 69.9 (51.4 to 94.7) | 0.0 (-0.1 to 0.1) |
| Guinea-Bissau | 4507.6 (4203.2 to 4792.9) | 3834.1 (3553.0 to 4159.5) | -0.5 (-0.7 to -0.4) | 89.1 (66.0 to 118.0) | 77.1 (57.0 to 104.1) | -0.4 (-0.7 to -0.2) |
| Guyana | 6655.4 (6201.3 to 7174.0) | 7417.5 (6887.5 to 8020.2) | 0.3 (0.1 to 0.4) | 109.2 (79.2 to 152.1) | 109.5 (77.5 to 157.5) | 0.0 (-0.1 to 0.1) |
| Haiti | 8889.8 (8229.1 to 9656.5) | 10227.7 (9218.2 to 11483.6) | 0.8 (0.6 to 1.1) | 144.5 (106.1 to 197.9) | 202.4 (139.7 to 302.5) | 1.8 (1.3 to 2.4) |
| Honduras | 9882.3 (8998.7 to 10848.5) | 8177.8 (7510.5 to 9024.6) | -0.7 (-0.8 to -0.5) | 150.1 (108.0 to 212.6) | 119.7 (84.7 to 172.6) | -0.8 (-0.9 to -0.6) |
| Hungary | 19070.9 (17424.6 to 20935.1) | 15260.2 (13784.0 to 16843.1) | -0.7 (-0.8 to -0.6) | 219.7 (140.8 to 348.0) | 133.8 (70.2 to 244.5) | -1.6 (-1.7 to -1.6) |
| Iceland | 7570.6 (6856.7 to 8466.9) | 6183.7 (5537.7 to 7079.5) | -0.6 (-0.7 to -0.5) | 71.8 (38.6 to 128.4) | 59.2 (31.4 to 106.9) | -0.6 (-0.7 to -0.5) |
| India | 5202.8 (4730.9 to 5781.7) | 4006.9 (3638.6 to 4466.4) | -1.0 (-1.1 to -0.9) | 104.0 (77.5 to 137.5) | 71.0 (51.0 to 98.1) | -1.4 (-1.5 to -1.3) |
| Indonesia | 5231.9 (4775.8 to 5918.6) | 3740.4 (3427.0 to 4165.2) | -1.0 (-1.1 to -0.9) | 93.0 (66.4 to 136.5) | 59.0 (41.6 to 87.0) | -1.4 (-1.6 to -1.3) |
| Iran (Islamic Republic of) | 10190.1 (9213.1 to 11469.1) | 6997.6 (6358.7 to 7758.3) | -1.3 (-1.4 to -1.2) | 180.5 (127.8 to 257.1) | 85.4 (53.9 to 136.1) | -2.5 (-2.6 to -2.3) |
| Iraq | 20237.3 (17310.4 to 23810.0) | 18362.7 (14755.5 to 23297.1) | -0.1 (-0.2 to -0.1) | 447.7 (289.2 to 677.5) | 303.1 (185.0 to 482.1) | -1.2 (-1.2 to -1.1) |
| Ireland | 7442.6 (6723.4 to 8324.4) | 6146.3 (5463.4 to 7030.7) | -0.6 (-0.7 to -0.5) | 71.5 (38.4 to 127.6) | 58.6 (30.6 to 105.9) | -0.6 (-0.7 to -0.5) |
| Israel | 7227.1 (6503.8 to 8162.1) | 6103.2 (5421.6 to 7059.7) | -0.4 (-0.5 to -0.3) | 71.5 (39.5 to 123.8) | 59.9 (32.6 to 106.9) | -0.4 (-0.5 to -0.2) |
| Italy | 7993.8 (7164.4 to 8949.1) | 5761.6 (5044.9 to 6805.7) | -1.3 (-1.5 to -1.2) | 78.0 (42.8 to 139.1) | 57.0 (30.4 to 101.9) | -1.2 (-1.3 to -1.1) |
| Jamaica | 8848.1 (8140.0 to 9672.7) | 7999.6 (7328.7 to 8779.9) | -0.7 (-0.9 to -0.6) | 101.6 (66.7 to 157.3) | 86.1 (54.0 to 139.4) | -0.9 (-1.1 to -0.8) |
| Japan | 6996.8 (6422.0 to 7679.9) | 5212.7 (4765.6 to 5754.4) | -1.0 (-1.1 to -0.9) | 64.6 (34.2 to 114.6) | 47.8 (25.1 to 84.7) | -1.0 (-1.1 to -0.9) |
| Jordan | 5906.3 (5471.1 to 6404.6) | 4504.7 (4129.9 to 4943.1) | -0.9 (-0.9 to -0.8) | 83.2 (57.7 to 121.1) | 48.9 (29.9 to 80.1) | -1.9 (-2.0 to -1.8) |
| Kazakhstan | 13372.2 (12306.3 to 14570.9) | 10763.9 (9917.6 to 11814.3) | -0.8 (-0.9 to -0.7) | 173.7 (117.8 to 261.4) | 118.2 (73.0 to 193.6) | -1.5 (-1.7 to -1.2) |
| Kenya | 3899.6 (3626.3 to 4160.6) | 3480.3 (3245.3 to 3726.9) | -0.6 (-0.7 to -0.4) | 65.8 (48.5 to 91.6) | 59.0 (43.0 to 82.3) | -0.4 (-0.6 to -0.2) |
| Kiribati | 2794.7 (2610.4 to 3019.6) | 2309.8 (2141.9 to 2516.6) | -0.5 (-0.7 to -0.4) | 66.1 (48.7 to 85.9) | 50.9 (37.5 to 68.4) | -0.7 (-0.8 to -0.7) |
| Kuwait | 6904.3 (6352.0 to 7540.4) | 6251.0 (5598.9 to 7052.8) | -0.7 (-0.9 to -0.4) | 81.1 (51.7 to 129.0) | 62.4 (34.7 to 107.3) | -1.6 (-1.9 to -1.4) |
| Kyrgyzstan | 10460.0 (9581.6 to 11566.5) | 7560.4 (7001.1 to 8269.5) | -1.1 (-1.2 to -1.1) | 147.6 (102.5 to 215.3) | 89.9 (58.3 to 140.2) | -1.7 (-1.8 to -1.7) |
| Lao People's Democratic Republic | 5067.7 (4753.3 to 5403.4) | 3662.0 (3417.7 to 3948.9) | -1.1 (-1.2 to -1.1) | 101.0 (75.0 to 134.0) | 62.7 (45.3 to 87.0) | -1.7 (-1.8 to -1.6) |
| Latvia | 18316.9 (16852.0 to 19999.0) | 11894.8 (10938.6 to 13059.0) | -1.7 (-1.8 to -1.6) | 221.7 (145.9 to 343.1) | 115.4 (65.7 to 197.4) | -2.5 (-2.6 to -2.3) |
| Lebanon | 14489.1 (10031.8 to 22451.5) | 8654.9 (6468.5 to 12476.9) | -1.7 (-1.8 to -1.6) | 340.9 (169.3 to 735.8) | 117.1 (60.0 to 225.2) | -3.5 (-3.6 to -3.4) |
| Lesotho | 3637.8 (3388.8 to 3866.2) | 4308.3 (4041.5 to 4568.4) | 0.7 (0.5 to 0.8) | 71.2 (52.6 to 96.9) | 89.6 (66.2 to 117.5) | 0.9 (0.7 to 1.1) |
| Liberia | 4247.5 (3890.5 to 4619.8) | 5985.0 (4763.6 to 7728.4) | 0.7 (0.2 to 1.1) | 80.9 (59.0 to 108.3) | 137.3 (89.0 to 205.2) | 0.8 (0.1 to 1.5) |
| Libya | 7353.0 (6806.2 to 7971.7) | 8821.5 (7594.7 to 10466.7) | 0.9 (0.6 to 1.1) | 111.2 (77.1 to 161.1) | 128.7 (85.4 to 197.4) | 0.7 (0.4 to 1.1) |
| Lithuania | 16161.9 (14859.0 to 17703.4) | 11512.2 (10535.3 to 12621.3) | -1.2 (-1.4 to -1.1) | 192.0 (124.0 to 302.2) | 115.6 (67.1 to 194.3) | -1.7 (-1.9 to -1.6) |
| Luxembourg | 8237.9 (7450.5 to 9155.1) | 6435.5 (5735.8 to 7300.1) | -0.9 (-0.9 to -0.8) | 83.3 (47.0 to 143.9) | 62.1 (33.2 to 111.2) | -0.9 (-1.0 to -0.8) |
| Madagascar | 4028.4 (3741.3 to 4321.4) | 3116.0 (2903.6 to 3349.5) | -0.8 (-0.9 to -0.7) | 67.5 (49.5 to 92.6) | 51.4 (37.1 to 72.5) | -0.9 (-0.9 to -0.8) |
| Malawi | 3566.6 (3290.6 to 3839.8) | 2901.7 (2676.6 to 3136.6) | -0.7 (-0.8 to -0.6) | 62.3 (45.9 to 84.8) | 48.9 (35.0 to 68.4) | -0.9 (-1.0 to -0.8) |
| Malaysia | 4178.5 (3881.3 to 4507.7) | 3561.9 (3302.8 to 3854.3) | -0.6 (-0.6 to -0.5) | 65.0 (46.6 to 92.2) | 46.4 (30.6 to 70.6) | -1.2 (-1.2 to -1.2) |
| Maldives | 5534.7 (5137.4 to 6002.0) | 4203.9 (3863.6 to 4610.6) | -0.8 (-0.9 to -0.7) | 83.3 (59.2 to 118.9) | 48.2 (30.8 to 76.7) | -1.7 (-1.9 to -1.6) |
| Mali | 4108.4 (3783.7 to 4385.4) | 5170.1 (4528.8 to 6009.5) | 0.9 (0.7 to 1.1) | 76.4 (56.7 to 101.8) | 101.6 (70.3 to 143.5) | 1.1 (0.7 to 1.5) |
| Malta | 7164.4 (6441.1 to 8074.7) | 6629.2 (5902.0 to 7561.9) | -0.2 (-0.3 to -0.1) | 71.6 (39.7 to 126.1) | 63.3 (33.1 to 113.6) | -0.2 (-0.4 to -0.1) |
| Marshall Islands | 3752.7 (3497.3 to 4028.3) | 3201.1 (2959.9 to 3482.3) | -0.5 (-0.6 to -0.5) | 76.6 (56.2 to 102.6) | 62.2 (45.3 to 83.4) | -0.7 (-0.8 to -0.6) |
| Mauritania | 3852.0 (3603.4 to 4094.7) | 3129.6 (2909.2 to 3382.8) | -0.7 (-0.8 to -0.6) | 73.7 (54.5 to 100.2) | 49.8 (35.4 to 70.4) | -1.3 (-1.4 to -1.2) |
| Mauritius | 3806.5 (3527.1 to 4114.3) | 3192.1 (2924.6 to 3493.2) | -0.4 (-0.5 to -0.4) | 54.1 (37.9 to 78.6) | 40.4 (26.5 to 61.6) | -0.8 (-0.8 to -0.7) |
| Mexico | 12924.9 (11796.0 to 14296.5) | 7999.2 (7297.9 to 8812.2) | -0.1 (-0.6 to 0.5) | 184.8 (129.8 to 268.0) | 97.9 (64.6 to 150.5) | -0.8 (-1.3 to -0.3) |
| Micronesia (Federated States of) | 3772.7 (3529.1 to 4051.5) | 3456.0 (3198.0 to 3779.9) | -0.3 (-0.3 to -0.2) | 79.6 (58.9 to 105.4) | 67.1 (49.1 to 91.9) | -0.5 (-0.6 to -0.5) |
| Monaco | 6133.1 (5473.1 to 7011.6) | 5910.6 (5260.3 to 6774.9) | -0.1 (-0.2 to 0.0) | 58.2 (30.6 to 105.3) | 56.1 (29.5 to 100.6) | -0.1 (-0.2 to 0.0) |
| Mongolia | 11905.8 (10976.1 to 12970.1) | 11742.6 (10800.8 to 12895.5) | 0.1 (0.0 to 0.1) | 180.7 (128.7 to 256.4) | 147.3 (99.2 to 223.4) | -0.7 (-0.8 to -0.6) |
| Montenegro | 18479.7 (16865.6 to 20345.9) | 15999.1 (14495.3 to 17635.4) | -0.5 (-0.6 to -0.4) | 177.1 (101.1 to 307.2) | 139.1 (72.4 to 254.9) | -0.9 (-1.1 to -0.8) |
| Morocco | 8226.3 (7665.3 to 8859.5) | 6073.9 (5630.3 to 6577.8) | -1.1 (-1.2 to -1.0) | 125.6 (90.6 to 180.0) | 79.5 (53.9 to 120.2) | -1.6 (-1.7 to -1.4) |
| Mozambique | 7904.5 (5758.5 to 11878.8) | 5702.8 (4495.8 to 7860.2) | -1.1 (-1.2 to -1.0) | 234.1 (134.4 to 419.0) | 141.2 (89.0 to 226.1) | -1.6 (-1.7 to -1.6) |
| Myanmar | 6885.2 (5959.5 to 9073.9) | 6063.7 (5261.0 to 7607.6) | -0.4 (-0.5 to -0.3) | 133.6 (90.8 to 214.2) | 107.1 (71.4 to 160.7) | -0.7 (-0.9 to -0.6) |
| Namibia | 6084.6 (5143.9 to 7407.7) | 4792.1 (4229.1 to 5549.7) | -0.8 (-0.9 to -0.7) | 138.8 (94.7 to 198.1) | 86.9 (60.7 to 122.6) | -1.6 (-1.7 to -1.4) |
| Nauru | 4088.9 (3814.7 to 4397.2) | 3637.6 (3356.9 to 3961.0) | -0.5 (-0.6 to -0.4) | 82.4 (60.2 to 109.3) | 70.3 (51.1 to 94.7) | -0.6 (-0.8 to -0.4) |
| Nepal | 4576.3 (4125.6 to 5094.7) | 5079.3 (4461.7 to 5866.6) | 0.5 (0.4 to 0.7) | 87.4 (63.3 to 117.8) | 91.1 (64.0 to 127.7) | 0.4 (0.2 to 0.7) |
| Netherlands | 5412.9 (4930.0 to 6059.1) | 4926.7 (4473.4 to 5582.4) | -0.2 (-0.5 to 0.0) | 51.0 (27.0 to 91.9) | 46.5 (24.4 to 83.8) | -0.2 (-0.5 to 0.0) |
| New Zealand | 17829.2 (15862.0 to 20053.3) | 15207.4 (13541.3 to 17195.5) | -0.6 (-0.7 to -0.4) | 155.3 (79.9 to 286.1) | 131.8 (66.2 to 245.3) | -0.6 (-0.8 to -0.5) |
| Nicaragua | 14909.8 (11492.8 to 20072.4) | 10204.4 (8350.4 to 12907.4) | -1.2 (-1.3 to -1.1) | 360.3 (210.6 to 583.7) | 175.6 (105.7 to 288.8) | -2.4 (-2.5 to -2.3) |
| Niger | 4072.7 (3751.5 to 4372.2) | 3712.2 (3410.2 to 4036.5) | -0.4 (-0.4 to -0.3) | 77.3 (56.5 to 104.3) | 69.1 (50.9 to 92.3) | -0.5 (-0.6 to -0.5) |
| Nigeria | 3983.9 (3688.0 to 4252.2) | 3292.4 (3023.3 to 3577.8) | -0.7 (-0.8 to -0.6) | 69.1 (51.2 to 94.0) | 56.6 (41.3 to 78.3) | -0.7 (-0.8 to -0.6) |
| Niue | 3496.4 (3238.2 to 3792.1) | 3163.6 (2904.4 to 3489.3) | -0.4 (-0.5 to -0.3) | 61.0 (43.3 to 85.0) | 48.9 (34.5 to 70.3) | -0.8 (-0.9 to -0.7) |
| North Macedonia | 18578.1 (17039.2 to 20382.8) | 16228.6 (14741.5 to 17976.9) | -0.5 (-0.6 to -0.4) | 200.0 (126.8 to 327.7) | 148.0 (82.7 to 262.5) | -1.1 (-1.2 to -1.0) |
| Northern Mariana Islands | 5276.7 (4891.4 to 5731.3) | 4059.2 (3726.8 to 4437.5) | -0.9 (-1.0 to -0.9) | 75.1 (52.6 to 111.4) | 57.2 (38.8 to 85.2) | -0.8 (-0.9 to -0.7) |
| Norway | 7984.0 (7235.2 to 8887.5) | 5709.2 (5162.1 to 6463.9) | -1.0 (-1.1 to -0.9) | 75.8 (41.0 to 135.5) | 53.9 (28.3 to 97.8) | -1.0 (-1.1 to -0.9) |
| Oman | 9074.2 (8510.6 to 9758.2) | 6412.1 (5893.7 to 7010.3) | -1.2 (-1.3 to -1.1) | 141.6 (99.1 to 203.9) | 71.0 (42.8 to 115.5) | -2.3 (-2.4 to -2.2) |
| Pakistan | 3246.1 (3031.2 to 3509.3) | 3413.8 (3077.9 to 3841.2) | 0.3 (0.2 to 0.3) | 61.8 (45.8 to 83.1) | 66.7 (47.8 to 91.6) | 0.4 (0.3 to 0.5) |
| Palau | 5271.9 (4881.4 to 5739.9) | 5116.4 (4683.6 to 5654.8) | -0.1 (-0.1 to -0.1) | 88.0 (62.4 to 122.2) | 76.6 (52.3 to 109.7) | -0.4 (-0.4 to -0.4) |
| Palestine | 11586.2 (9266.9 to 15067.4) | 13278.3 (9980.0 to 18099.8) | 1.1 (0.8 to 1.4) | 268.8 (166.1 to 417.2) | 231.3 (137.4 to 368.7) | 0.2 (-0.1 to 0.6) |
| Panama | 8769.9 (7821.4 to 9870.8) | 7651.0 (6869.1 to 8615.1) | -0.5 (-0.6 to -0.4) | 119.1 (78.7 to 181.3) | 84.1 (51.8 to 137.5) | -1.1 (-1.2 to -1.0) |
| Papua New Guinea | 3175.8 (2949.4 to 3450.2) | 3651.7 (3317.8 to 4049.2) | 0.4 (0.3 to 0.5) | 66.1 (48.6 to 87.0) | 76.4 (56.0 to 102.9) | 0.5 (0.3 to 0.6) |
| Paraguay | 7596.7 (6864.8 to 8501.6) | 6620.2 (6035.2 to 7332.8) | -0.5 (-0.6 to -0.4) | 104.7 (72.2 to 153.7) | 82.6 (55.4 to 125.3) | -0.8 (-0.9 to -0.7) |
| Peru | 8354.0 (7617.3 to 9348.3) | 7254.6 (6604.5 to 8061.9) | -0.5 (-0.6 to -0.5) | 131.2 (90.5 to 190.3) | 82.5 (51.9 to 134.8) | -1.7 (-1.8 to -1.6) |
| Philippines | 5579.1 (4923.2 to 6789.0) | 4184.0 (3685.7 to 4969.7) | -0.8 (-0.9 to -0.8) | 103.9 (72.4 to 156.5) | 73.9 (51.6 to 109.5) | -1.1 (-1.1 to -1.0) |
| Poland | 19184.5 (17583.3 to 21048.1) | 14298.3 (13062.5 to 15774.3) | -0.9 (-1.0 to -0.9) | 222.6 (144.4 to 348.7) | 127.7 (67.3 to 229.6) | -1.8 (-1.9 to -1.8) |
| Portugal | 7967.6 (7251.3 to 8806.9) | 4574.7 (4139.1 to 5138.7) | -2.0 (-2.1 to -1.9) | 87.2 (53.4 to 143.2) | 44.4 (24.1 to 79.2) | -2.3 (-2.4 to -2.2) |
| Puerto Rico | 6977.0 (6399.3 to 7613.2) | 7183.3 (6535.3 to 7935.0) | 0.1 (-0.1 to 0.2) | 84.3 (54.6 to 131.5) | 68.1 (37.6 to 119.2) | -0.8 (-0.9 to -0.7) |
| Qatar | 9204.0 (8504.3 to 9995.8) | 7189.4 (6544.5 to 7916.2) | -0.7 (-0.8 to -0.5) | 117.1 (77.6 to 178.5) | 66.9 (36.7 to 118.5) | -1.9 (-2.0 to -1.7) |
| Republic of Korea | 9177.8 (8387.5 to 10118.5) | 5832.5 (5312.7 to 6510.0) | -1.9 (-2.1 to -1.7) | 104.9 (66.5 to 170.2) | 54.7 (29.0 to 98.4) | -2.4 (-2.6 to -2.2) |
| Republic of Moldova | 15318.9 (14086.9 to 16691.7) | 9892.8 (9080.5 to 10822.0) | -1.6 (-1.7 to -1.6) | 194.6 (130.7 to 296.4) | 105.7 (63.8 to 174.4) | -2.2 (-2.3 to -2.0) |
| Romania | 22313.1 (20366.9 to 24536.5) | 16821.4 (15343.0 to 18576.7) | -1.0 (-1.1 to -1.0) | 266.3 (177.1 to 414.9) | 154.3 (83.7 to 275.4) | -1.9 (-2.0 to -1.9) |
| Russian Federation | 18939.4 (17446.4 to 20659.8) | 12537.6 (11560.8 to 13781.9) | -1.7 (-1.9 to -1.4) | 227.4 (147.6 to 353.2) | 128.1 (75.4 to 215.7) | -2.3 (-2.7 to -1.9) |
| Rwanda | 4074.7 (3786.5 to 4344.7) | 8123.3 (6221.6 to 10497.2) | 0.9 (-0.2 to 2.1) | 83.9 (63.1 to 111.0) | 333.4 (190.5 to 558.3) | 1.8 (-0.5 to 4.1) |
| Saint Kitts and Nevis | 8858.4 (8131.7 to 9698.2) | 9672.4 (8825.2 to 10615.7) | 0.5 (0.3 to 0.7) | 122.0 (86.1 to 177.0) | 106.7 (67.2 to 170.3) | -0.2 (-0.4 to -0.1) |
| Saint Lucia | 6669.8 (6143.3 to 7275.4) | 7240.6 (6646.7 to 7932.2) | 0.3 (0.1 to 0.4) | 91.4 (63.4 to 133.1) | 82.1 (52.8 to 129.1) | -0.3 (-0.4 to -0.3) |
| Saint Vincent and the Grenadines | 7002.7 (6477.6 to 7644.2) | 7576.1 (7019.1 to 8249.6) | 0.2 (0.1 to 0.4) | 93.6 (65.5 to 138.4) | 90.7 (60.5 to 139.5) | -0.1 (-0.3 to 0.0) |
| Samoa | 3464.4 (3229.7 to 3752.4) | 3462.5 (3154.3 to 3830.3) | 0.1 (0.0 to 0.2) | 63.2 (45.7 to 86.3) | 59.4 (42.2 to 84.5) | 0.0 (-0.2 to 0.1) |
| San Marino | 6946.4 (6246.3 to 7831.3) | 6372.0 (5698.3 to 7234.4) | -0.2 (-0.3 to -0.2) | 64.4 (33.3 to 115.8) | 59.6 (31.0 to 107.9) | -0.2 (-0.3 to -0.1) |
| Sao Tome and Principe | 4328.4 (3934.6 to 4820.9) | 4013.2 (3645.4 to 4419.5) | -0.2 (-0.3 to -0.2) | 69.0 (49.2 to 96.1) | 60.3 (42.8 to 85.5) | -0.5 (-0.5 to -0.4) |
| Saudi Arabia | 13400.4 (12320.7 to 14618.5) | 12164.4 (11004.8 to 13545.1) | 0.0 (-0.4 to 0.4) | 191.4 (132.9 to 279.4) | 135.9 (85.0 to 217.5) | -0.9 (-1.1 to -0.7) |
| Senegal | 3577.4 (3281.4 to 3837.8) | 3214.7 (2951.0 to 3485.2) | -0.4 (-0.4 to -0.3) | 60.1 (44.6 to 82.6) | 52.3 (37.0 to 73.5) | -0.4 (-0.5 to -0.4) |
| Serbia | 18499.4 (16892.0 to 20422.5) | 16182.6 (14721.6 to 17911.8) | -0.4 (-0.5 to -0.3) | 204.1 (129.6 to 328.6) | 144.8 (77.4 to 261.8) | -1.2 (-1.3 to -1.0) |
| Seychelles | 5079.0 (4668.2 to 5499.9) | 3743.6 (3467.7 to 4069.8) | -0.9 (-1.0 to -0.9) | 74.4 (52.2 to 108.7) | 49.1 (33.3 to 74.5) | -1.3 (-1.4 to -1.2) |
| Sierra Leone | 3760.9 (3477.2 to 4070.0) | 5294.0 (4403.0 to 6525.1) | 0.5 (-0.1 to 1.0) | 64.6 (46.7 to 87.7) | 113.4 (75.2 to 165.4) | 1.2 (0.4 to 2.1) |
| Singapore | 7260.9 (6601.8 to 8028.7) | 6369.4 (5777.4 to 7083.9) | -0.5 (-0.6 to -0.4) | 73.1 (42.4 to 125.4) | 57.6 (29.5 to 104.9) | -0.8 (-0.8 to -0.8) |
| Slovakia | 21512.3 (19714.3 to 23709.2) | 17864.1 (16232.2 to 19774.1) | -0.6 (-0.6 to -0.6) | 233.8 (147.3 to 383.0) | 155.5 (80.9 to 287.0) | -1.3 (-1.4 to -1.3) |
| Slovenia | 25668.3 (23513.0 to 28159.8) | 20066.9 (18261.0 to 22078.6) | -0.4 (-0.7 to 0.0) | 249.7 (144.5 to 431.3) | 174.4 (90.9 to 320.3) | -0.7 (-1.0 to -0.4) |
| Solomon Islands | 4286.2 (3990.1 to 4613.3) | 4253.8 (3891.5 to 4739.8) | -0.1 (-0.1 to 0.0) | 94.6 (69.6 to 125.3) | 87.2 (63.5 to 116.0) | -0.3 (-0.3 to -0.2) |
| Somalia | 5135.0 (4214.1 to 8344.8) | 6440.3 (5205.2 to 8958.6) | 0.5 (0.4 to 0.5) | 117.6 (71.1 to 264.3) | 158.5 (101.5 to 260.1) | 0.5 (0.4 to 0.6) |
| South Africa | 6218.3 (5759.7 to 6810.9) | 4070.5 (3778.9 to 4447.0) | -1.5 (-1.7 to -1.4) | 121.7 (89.0 to 169.1) | 69.5 (49.4 to 97.5) | -2.0 (-2.3 to -1.8) |
| South Sudan | 5366.4 (4730.7 to 6155.8) | 6765.0 (5459.8 to 8680.3) | 1.0 (0.9 to 1.2) | 108.4 (75.3 to 151.6) | 145.9 (95.6 to 218.4) | 1.2 (1.0 to 1.5) |
| Spain | 7008.0 (6406.7 to 7744.6) | 5618.7 (5005.3 to 6449.3) | -0.7 (-0.8 to -0.6) | 67.5 (37.0 to 119.1) | 54.2 (28.6 to 96.8) | -0.7 (-0.8 to -0.6) |
| Sri Lanka | 7345.0 (6489.8 to 8549.6) | 9258.5 (7370.0 to 11959.7) | 0.6 (0.4 to 0.8) | 126.9 (87.2 to 187.6) | 140.4 (86.7 to 220.0) | 0.1 (-0.3 to 0.4) |
| Sudan | 7237.4 (6653.8 to 7949.1) | 7218.6 (6246.8 to 8563.2) | 0.1 (-0.1 to 0.2) | 129.4 (93.2 to 178.8) | 120.0 (80.2 to 176.2) | -0.2 (-0.4 to 0.1) |
| Suriname | 6706.7 (5850.8 to 8941.6) | 6609.7 (5955.5 to 7949.7) | 0.0 (-0.1 to 0.1) | 108.7 (71.7 to 174.9) | 90.9 (60.3 to 140.2) | -0.6 (-0.6 to -0.5) |
| Sweden | 7386.8 (6670.7 to 8256.8) | 5735.6 (5154.8 to 6503.5) | -0.8 (-0.9 to -0.7) | 69.2 (36.0 to 125.0) | 54.0 (28.2 to 97.8) | -0.8 (-0.9 to -0.6) |
| Switzerland | 10675.0 (9576.4 to 11898.7) | 7296.5 (6465.1 to 8296.0) | -1.5 (-1.7 to -1.3) | 99.0 (51.4 to 179.7) | 69.2 (36.5 to 124.8) | -1.4 (-1.7 to -1.2) |
| Syrian Arab Republic | 6201.6 (5459.1 to 7516.6) | 15382.5 (11077.9 to 21355.1) | 2.9 (2.1 to 3.8) | 101.1 (68.5 to 161.2) | 298.0 (170.5 to 491.8) | 3.5 (2.2 to 4.9) |
| Taiwan (Province of China) | 3735.4 (3421.4 to 4123.9) | 2203.8 (2035.0 to 2409.9) | -2.1 (-2.2 to -1.9) | 56.0 (37.5 to 83.2) | 25.8 (15.1 to 42.2) | -2.9 (-3.0 to -2.7) |
| Tajikistan | 9502.8 (8718.9 to 10434.1) | 8907.9 (7929.0 to 10062.2) | -0.6 (-0.9 to -0.4) | 134.8 (93.4 to 196.8) | 141.5 (95.3 to 208.1) | -0.7 (-1.1 to -0.2) |
| Thailand | 5786.3 (5415.2 to 6178.9) | 4428.6 (4071.7 to 4822.1) | -1.1 (-1.1 to -1.0) | 91.8 (65.7 to 130.0) | 54.1 (34.5 to 85.8) | -2.0 (-2.1 to -1.9) |
| Timor-Leste | 10815.2 (8281.2 to 14380.6) | 10385.3 (7669.0 to 14479.1) | -0.3 (-0.6 to 0.1) | 321.5 (198.8 to 490.3) | 281.0 (171.3 to 442.5) | -0.6 (-1.1 to -0.2) |
| Togo | 3752.6 (3469.4 to 4012.1) | 3239.5 (2996.9 to 3496.1) | -0.5 (-0.6 to -0.5) | 67.2 (49.9 to 92.0) | 57.3 (41.3 to 79.7) | -0.6 (-0.6 to -0.5) |
| Tokelau | 3356.0 (3126.3 to 3630.5) | 2827.8 (2597.1 to 3111.1) | -0.6 (-0.7 to -0.5) | 62.1 (45.2 to 85.0) | 44.4 (31.2 to 64.0) | -1.1 (-1.2 to -1.0) |
| Tonga | 2975.2 (2765.2 to 3245.8) | 2396.5 (2214.8 to 2653.0) | -0.7 (-0.8 to -0.6) | 50.7 (36.5 to 71.1) | 37.0 (26.0 to 53.1) | -1.0 (-1.0 to -0.9) |
| Trinidad and Tobago | 6109.3 (5642.5 to 6696.7) | 6288.9 (5776.3 to 6886.0) | 0.2 (0.1 to 0.4) | 86.2 (60.0 to 125.0) | 81.0 (54.3 to 123.6) | -0.2 (-0.3 to -0.1) |
| Tunisia | 9227.6 (8507.4 to 10087.2) | 7077.6 (6541.7 to 7758.7) | -0.9 (-0.9 to -0.8) | 115.6 (76.9 to 176.1) | 72.9 (42.9 to 120.0) | -1.5 (-1.6 to -1.5) |
| Turkey | 6567.8 (6074.9 to 7134.7) | 5251.3 (4841.5 to 5770.8) | -0.7 (-0.8 to -0.7) | 92.1 (64.2 to 135.2) | 56.4 (33.6 to 92.8) | -1.7 (-1.7 to -1.7) |
| Turkmenistan | 9648.8 (8831.1 to 10630.0) | 9277.4 (8542.5 to 10249.7) | -0.1 (-0.1 to 0.0) | 130.3 (90.4 to 192.4) | 107.0 (70.3 to 170.3) | -0.7 (-0.8 to -0.7) |
| Tuvalu | 3575.2 (3351.5 to 3831.1) | 3122.2 (2872.1 to 3423.1) | -1.1 (-1.4 to -0.8) | 77.3 (57.0 to 102.7) | 58.5 (41.9 to 79.6) | -1.4 (-1.6 to -1.2) |
| Uganda | 7130.8 (5365.6 to 9964.5) | 5241.0 (4186.3 to 6872.9) | -1.0 (-1.1 to -0.9) | 184.5 (111.5 to 297.8) | 113.8 (73.8 to 175.5) | -1.6 (-1.8 to -1.5) |
| Ukraine | 15263.0 (13864.3 to 16896.4) | 13182.5 (12095.9 to 14512.2) | -0.8 (-0.9 to -0.7) | 185.6 (121.7 to 291.0) | 147.3 (91.5 to 237.5) | -1.2 (-1.4 to -1.0) |
| United Arab Emirates | 8699.5 (8082.8 to 9386.0) | 7597.4 (6947.9 to 8356.8) | -0.4 (-0.5 to -0.4) | 126.4 (87.5 to 184.5) | 89.7 (57.5 to 142.5) | -1.1 (-1.2 to -1.1) |
| United Kingdom | 6247.1 (5649.2 to 6990.7) | 5020.9 (4498.4 to 5679.0) | -0.7 (-0.8 to -0.5) | 61.0 (33.7 to 108.1) | 47.7 (25.4 to 86.0) | -0.7 (-0.9 to -0.6) |
| United Republic of Tanzania | 3852.3 (3563.6 to 4127.1) | 3326.5 (3077.1 to 3590.0) | -0.5 (-0.5 to -0.4) | 66.2 (48.7 to 90.1) | 54.7 (39.4 to 76.8) | -0.6 (-0.7 to -0.6) |
| United States of America | 6465.8 (5936.2 to 7134.2) | 3995.5 (3706.4 to 4350.1) | -1.8 (-2.2 to -1.4) | 61.6 (33.5 to 108.9) | 39.0 (21.6 to 67.2) | -1.7 (-2.0 to -1.3) |
| United States Virgin Islands | 7170.4 (6613.4 to 7831.3) | 6468.4 (5948.8 to 7102.7) | -0.6 (-0.8 to -0.3) | 93.9 (64.2 to 141.2) | 72.6 (46.1 to 115.2) | -1.1 (-1.3 to -0.9) |
| Uruguay | 15659.9 (14021.4 to 17664.5) | 11638.2 (10537.1 to 12994.1) | -1.2 (-1.2 to -1.1) | 143.9 (81.1 to 255.6) | 102.9 (54.6 to 187.4) | -1.3 (-1.3 to -1.2) |
| Uzbekistan | 9594.4 (8809.3 to 10475.8) | 7946.3 (7347.6 to 8644.0) | -0.7 (-0.7 to -0.6) | 125.8 (86.2 to 188.8) | 96.8 (63.8 to 149.3) | -1.0 (-1.1 to -0.9) |
| Vanuatu | 3474.5 (3243.3 to 3768.3) | 3051.8 (2821.8 to 3336.6) | -0.5 (-0.5 to -0.4) | 67.0 (49.7 to 91.2) | 58.7 (43.2 to 81.1) | -0.4 (-0.5 to -0.3) |
| Venezuela (Bolivarian Republic of) | 11431.5 (10328.7 to 12694.7) | 10813.6 (9828.1 to 11940.4) | -0.1 (-0.2 to 0.0) | 154.7 (107.1 to 227.5) | 128.9 (84.0 to 200.9) | -0.5 (-0.6 to -0.4) |
| Viet Nam | 5273.7 (4873.4 to 5688.5) | 4550.6 (4230.4 to 4920.9) | -0.4 (-0.4 to -0.3) | 80.3 (57.3 to 115.9) | 58.6 (38.5 to 89.3) | -0.9 (-1.0 to -0.9) |
| Yemen | 7102.9 (6580.0 to 7869.0) | 8556.0 (7090.2 to 10589.3) | 0.3 (0.0 to 0.5) | 136.3 (98.7 to 186.8) | 171.0 (114.9 to 254.8) | 0.1 (-0.3 to 0.5) |
| Zambia | 3782.2 (3505.3 to 4034.5) | 3304.6 (3065.6 to 3568.7) | -0.5 (-0.6 to -0.4) | 69.4 (51.8 to 93.7) | 57.5 (41.3 to 78.9) | -0.7 (-0.8 to -0.5) |
| Zimbabwe | 3539.8 (3302.8 to 3782.8) | 3264.7 (3062.6 to 3487.4) | -0.3 (-0.4 to -0.2) | 64.9 (47.6 to 88.2) | 61.9 (45.2 to 83.1) | 0.0 (-0.1 to 0.1) |

**Table S4.** **Cases and ASR of prevalence and YLDs in 2021, and their EAPC from 1990 to 2021 for overall amputations, globally and by 21 GBD regions.**

| **Characteristics** | **Prevalence** | | | **YLDs** | | |
| --- | --- | --- | --- | --- | --- | --- |
|  | **Cases No. (95% UI)** | **ASR per 100,000 No. (95% UI)** | **EAPC of prevalence rate No. (95% CI)** | **Cases No. (95% UI)** | **ASR per 100,000 No. (95% UI)** | **EAPC of YLDs rate No. (95% CI)** |
| Global | 445237916.8 (408967911.0 to 485841239.1) | 5333.4 (4894.6 to 5818.4) | -1.0 (-1.0 to -1.0) | 5935830.8 (3987841.5 to 9066590.3) | 71.1 (47.8 to 108.6) | -1.4 (-1.5 to -1.3) |
| Andean Latin America | 4526483.8 (4155418.8 to 4955015.5) | 6922.7 (6369.2 to 7567.9) | -0.5 (-0.6 to -0.5) | 54189.9 (35713.7 to 85293.2) | 83.4 (55.1 to 130.8) | -1.4 (-1.5 to -1.3) |
| Australasia | 4834466.2 (4326706.6 to 5434481.7) | 13110.4 (11638.3 to 14826.7) | -0.6 (-0.7 to -0.5) | 41599.5 (20842.6 to 77699.3) | 113.4 (56.3 to 213.0) | -0.6 (-0.7 to -0.5) |
| Caribbean | 4061195.4 (3750913.1 to 4420157.1) | 8027.0 (7399.9 to 8740.6) | 0.4 (0.3 to 0.5) | 56143.8 (38561.6 to 83779.5) | 111.8 (77.0 to 166.8) | 0.7 (0.5 to 0.9) |
| Central Asia | 8818166.7 (8153838.8 to 9665919.4) | 9251.7 (8559.5 to 10132.1) | -0.7 (-0.8 to -0.7) | 105471.9 (69116.7 to 165101.3) | 111.1 (73.0 to 173.6) | -1.2 (-1.3 to -1.0) |
| Central Europe | 24195518.9 (22311758.1 to 26546009.5) | 16129.1 (14704.0 to 17802.5) | -0.8 (-0.9 to -0.7) | 221534.9 (120161.7 to 395495.3) | 145.3 (78.6 to 262.7) | -1.6 (-1.7 to -1.6) |
| Central Latin America | 22814633.7 (20713182.4 to 25323872.0) | 8699.4 (7896.9 to 9656.8) | -0.4 (-0.7 to -0.1) | 284696.1 (189197.6 to 438031.3) | 108.7 (72.3 to 167.0) | -1.2 (-1.5 to -1.0) |
| Central Sub-Saharan Africa | 4567871.6 (3859682.2 to 5589867.6) | 4518.0 (3836.6 to 5499.1) | 0.1 (-0.2 to 0.3) | 94589.1 (64190.0 to 138734.0) | 98.9 (67.8 to 143.2) | 0.1 (-0.3 to 0.4) |
| East Asia | 67248501.5 (60889834.6 to 74988845.7) | 3645.0 (3291.1 to 4100.4) | -0.5 (-0.8 to -0.3) | 819571.7 (499411.6 to 1320842.9) | 43.7 (26.5 to 70.4) | -2.4 (-2.7 to -2.1) |
| Eastern Europe | 33534302.3 (30874286.0 to 36825645.3) | 12592.1 (11606.7 to 13833.9) | -1.4 (-1.6 to -1.3) | 354036.8 (213524.0 to 588421.3) | 130.9 (78.3 to 218.9) | -2.0 (-2.4 to -1.7) |
| Eastern Sub-Saharan Africa | 14680600.9 (12496276.0 to 18077377.2) | 4775.4 (4058.3 to 5926.3) | -0.5 (-0.6 to -0.3) | 308216.7 (204116.3 to 471182.8) | 107.3 (71.9 to 163.0) | -0.6 (-0.9 to -0.3) |
| High-income Asia Pacific | 13892083.5 (12756641.3 to 15213275.6) | 5435.1 (4966.2 to 6022.5) | -1.2 (-1.3 to -1.1) | 127325.1 (68067.2 to 223782.0) | 50.1 (26.4 to 89.3) | -1.4 (-1.4 to -1.3) |
| High-income North America | 18780323.3 (17391281.3 to 20367218.2) | 3989.6 (3699.3 to 4344.6) | -1.7 (-2.1 to -1.4) | 183325.3 (102588.2 to 314518.0) | 38.8 (21.5 to 66.9) | -1.6 (-1.9 to -1.3) |
| North Africa and Middle East | 48250179.0 (42179140.2 to 56635202.8) | 8034.7 (7058.7 to 9380.0) | -0.4 (-0.4 to -0.3) | 719935.7 (471909.8 to 1106706.8) | 120.3 (79.0 to 185.0) | -1.1 (-1.1 to -1.0) |
| Oceania | 407527.8 (369139.3 to 455428.6) | 3542.4 (3224.1 to 3921.5) | 0.2 (0.1 to 0.3) | 7816.6 (5681.1 to 10609.5) | 71.2 (52.3 to 95.4) | 0.3 (0.2 to 0.4) |
| South Asia | 68907502.2 (62351184.5 to 77093433.8) | 3900.7 (3545.3 to 4344.6) | -0.8 (-0.9 to -0.7) | 1180048.7 (847883.3 to 1638007.1) | 69.2 (50.0 to 96.3) | -1.2 (-1.3 to -1.1) |
| Southeast Asia | 32246948.3 (29274253.2 to 36387942.8) | 4424.3 (4025.8 to 4962.3) | -0.8 (-0.8 to -0.7) | 491802.3 (343590.0 to 741454.3) | 68.0 (47.5 to 101.4) | -1.3 (-1.4 to -1.2) |
| Southern Latin America | 7726464.4 (6997868.8 to 8609801.8) | 10187.1 (9182.3 to 11381.7) | -0.2 (-0.4 to -0.1) | 70348.0 (38972.1 to 126367.4) | 93.0 (51.2 to 167.3) | -0.5 (-0.7 to -0.4) |
| Southern Sub-Saharan Africa | 2981298.7 (2761355.2 to 3233578.3) | 3976.5 (3698.2 to 4303.4) | -1.3 (-1.4 to -1.1) | 50709.3 (36104.9 to 71253.0) | 69.2 (49.6 to 96.4) | -1.7 (-1.9 to -1.5) |
| Tropical Latin America | 17666174.8 (16007599.9 to 19672543.0) | 7039.2 (6369.7 to 7850.0) | -1.1 (-1.2 to -0.9) | 237158.7 (159964.8 to 354954.8) | 94.3 (63.5 to 141.3) | -1.5 (-1.6 to -1.4) |
| Western Europe | 32070877.1 (28855355.4 to 36152850.7) | 5759.5 (5138.6 to 6600.3) | -0.7 (-0.8 to -0.7) | 308368.5 (166821.9 to 547797.6) | 55.6 (29.7 to 99.4) | -0.8 (-0.8 to -0.7) |
| Western Sub-Saharan Africa | 13026796.8 (11739374.5 to 14395265.2) | 3572.3 (3278.6 to 3904.9) | -0.4 (-0.5 to -0.3) | 218942.6 (156980.9 to 303605.8) | 63.7 (46.5 to 86.9) | -0.4 (-0.5 to -0.3) |

**Table S5.** **Cases and ASR of prevalence and YLDs in 2021,** **and their EAPC from 1990 to 2021 for AMP-F, globally and by 21 GBD regions.**

| **Characteristics** | **Prevalence** | | | **YLDs** | | |
| --- | --- | --- | --- | --- | --- | --- |
|  | **Cases No. (95% UI)** | **ASR per 100,000 No. (95% UI)** | **EAPC of prevalence rate No. (95% CI)** | **Cases No. (95% UI)** | **ASR per 100,000 No. (95% UI)** | **EAPC of YLDs rate No. (95% CI)** |
| Global | 223793376.6 (204337382.6 to 246011335.7) | 2681.1 (2446.5 to 2946.1) | -1.0 (-1.0 to -1.0) | 1019501.6 (369661.0 to 2253382.8) | 12.2 (4.4 to 27.0) | -1.0 (-1.0 to -0.9) |
| Andean Latin America | 2509650.9 (2291793.7 to 2774661.3) | 3836.1 (3504.2 to 4236.8) | -0.5 (-0.6 to -0.5) | 11492.9 (4157.8 to 25286.3) | 17.5 (6.4 to 38.6) | -0.5 (-0.6 to -0.5) |
| Australasia | 2156770.8 (1919891.1 to 2428973.2) | 5816.2 (5124.5 to 6611.2) | -0.7 (-0.7 to -0.6) | 9782.1 (3572.9 to 21558.9) | 26.6 (9.6 to 59.0) | -0.7 (-0.7 to -0.6) |
| Caribbean | 2282629.1 (2086999.4 to 2498353.2) | 4510.4 (4115.6 to 4949.0) | 0.4 (0.3 to 0.5) | 10412.7 (3809.1 to 22858.1) | 20.6 (7.5 to 45.2) | 0.4 (0.3 to 0.5) |
| Central Asia | 4983728.6 (4582347.7 to 5480290.7) | 5219.8 (4806.3 to 5726.5) | -0.7 (-0.8 to -0.7) | 22900.0 (8421.5 to 50614.4) | 23.9 (8.8 to 52.8) | -0.7 (-0.8 to -0.7) |
| Central Europe | 13717382.1 (12589797.2 to 15040701.3) | 9179.4 (8340.0 to 10115.1) | -0.8 (-0.9 to -0.7) | 62081.2 (23138.3 to 136263.9) | 42.0 (15.6 to 93.1) | -0.8 (-0.9 to -0.7) |
| Central Latin America | 12497997.5 (11273891.6 to 13830534.5) | 4762.8 (4298.1 to 5267.6) | -0.4 (-0.7 to 0.0) | 57085.2 (20849.8 to 125743.7) | 21.7 (7.9 to 47.9) | -0.4 (-0.7 to 0.0) |
| Central Sub-Saharan Africa | 2311653.6 (1818973.5 to 3133174.1) | 2269.4 (1791.0 to 3094.5) | 0.0 (-0.2 to 0.2) | 10631.0 (3782.7 to 23332.1) | 10.3 (3.7 to 22.6) | 0.0 (-0.2 to 0.2) |
| East Asia | 32754573.8 (29407970.0 to 36575976.5) | 1781.2 (1602.8 to 2000.1) | -0.5 (-0.7 to -0.2) | 149389.2 (56493.1 to 329766.2) | 8.2 (3.1 to 18.1) | -0.5 (-0.7 to -0.2) |
| Eastern Europe | 18214277.7 (16798574.0 to 19910119.0) | 6861.0 (6336.2 to 7493.5) | -1.5 (-1.7 to -1.3) | 82230.7 (30732.8 to 177564.2) | 31.2 (11.7 to 67.5) | -1.5 (-1.7 to -1.3) |
| Eastern Sub-Saharan Africa | 7527543.1 (6159849.3 to 9988125.1) | 2426.9 (1973.5 to 3256.9) | -0.5 (-0.7 to -0.4) | 34654.2 (12767.2 to 75881.2) | 11.1 (4.1 to 24.4) | -0.5 (-0.7 to -0.4) |
| High-income Asia Pacific | 5940769.8 (5424573.2 to 6568194.5) | 2297.2 (2074.1 to 2553.9) | -1.2 (-1.3 to -1.1) | 26913.8 (9898.1 to 58178.9) | 10.6 (3.9 to 23.1) | -1.2 (-1.3 to -1.1) |
| High-income North America | 7633727.7 (6997889.4 to 8322394.5) | 1618.8 (1484.5 to 1775.5) | -1.8 (-2.2 to -1.5) | 34179.9 (12633.8 to 74087.2) | 7.3 (2.7 to 16.0) | -1.9 (-2.2 to -1.5) |
| North Africa and Middle East | 25281356.5 (21360741.7 to 30952779.7) | 4198.9 (3559.4 to 5129.4) | -0.4 (-0.5 to -0.4) | 115903.5 (42417.5 to 253424.0) | 19.2 (7.0 to 41.8) | -0.4 (-0.5 to -0.4) |
| Oceania | 206548.9 (185133.5 to 232235.3) | 1780.1 (1610.2 to 1972.5) | 0.1 (0.0 to 0.2) | 948.0 (337.6 to 2077.3) | 8.1 (2.9 to 17.8) | 0.1 (0.0 to 0.2) |
| South Asia | 34525662.0 (31304827.1 to 38258424.4) | 1936.8 (1762.3 to 2138.7) | -0.9 (-1.0 to -0.8) | 156915.9 (57357.9 to 347444.9) | 8.8 (3.2 to 19.4) | -0.9 (-1.0 to -0.8) |
| Southeast Asia | 17092198.9 (15348259.0 to 19983193.9) | 2336.4 (2103.5 to 2720.7) | -0.8 (-0.9 to -0.7) | 78462.6 (27571.7 to 170637.1) | 10.7 (3.8 to 23.3) | -0.8 (-0.9 to -0.7) |
| Southern Latin America | 3535282.5 (3178594.5 to 3936193.7) | 4646.1 (4156.9 to 5183.4) | -0.3 (-0.4 to -0.1) | 16128.6 (5913.9 to 35409.7) | 21.3 (7.8 to 46.9) | -0.3 (-0.4 to -0.1) |
| Southern Sub-Saharan Africa | 1560573.0 (1443013.4 to 1704453.0) | 2071.4 (1920.8 to 2256.9) | -1.3 (-1.4 to -1.1) | 7098.3 (2609.7 to 15461.9) | 9.4 (3.4 to 20.4) | -1.3 (-1.4 to -1.1) |
| Tropical Latin America | 9389739.8 (8523294.5 to 10456518.4) | 3737.6 (3393.2 to 4164.3) | -1.1 (-1.2 to -0.9) | 42645.6 (15975.8 to 94491.6) | 17.0 (6.4 to 37.8) | -1.1 (-1.2 to -0.9) |
| Western Europe | 12872619.0 (11683246.2 to 14311191.2) | 2288.2 (2060.8 to 2563.2) | -0.8 (-0.8 to -0.7) | 58301.3 (21118.9 to 127376.8) | 10.5 (3.8 to 23.3) | -0.8 (-0.8 to -0.7) |
| Western Sub-Saharan Africa | 6798691.5 (6061866.8 to 7594880.6) | 1858.5 (1678.0 to 2059.4) | -0.4 (-0.5 to -0.3) | 31345.2 (11412.4 to 69009.6) | 8.5 (3.1 to 18.7) | -0.4 (-0.5 to -0.3) |

**Table S6. Cases and ASR of prevalence and YLDs in 2021, and their EAPC from 1990 to 2021 for AMP-LL1, globally and by 21 GBD regions.**

| **Characteristics** | **Prevalence** | | | **YLDs** | | |
| --- | --- | --- | --- | --- | --- | --- |
|  | **Cases No. (95% UI)** | **ASR per 100,000 No. (95% UI)** | **EAPC of prevalence rate No. (95% CI)** | **Cases No. (95% UI)** | **ASR per 100,000 No. (95% UI)** | **EAPC of YLDs rate No. (95% CI)** |
| Global | 19271753.1 (17546973.8 to 21791173.5) | 227.7 (206.9 to 257.2) | -0.4 (-0.4 to -0.3) | 1231468.3 (845240.7 to 1675869.5) | 14.6 (10.0 to 19.9) | -1.6 (-1.8 to -1.5) |
| Andean Latin America | 154962.6 (142363.6 to 173577.8) | 247.6 (227.7 to 277.0) | -0.4 (-0.5 to -0.3) | 10347.9 (7087.2 to 14039.4) | 16.5 (11.3 to 22.4) | -2.0 (-2.1 to -1.9) |
| Australasia | 12442.7 (11542.1 to 13260.5) | 28.1 (26.2 to 29.9) | -0.5 (-0.5 to -0.4) | 448.1 (273.7 to 675.1) | 1.0 (0.6 to 1.5) | -0.6 (-0.6 to -0.5) |
| Caribbean | 157105.6 (137751.7 to 197337.8) | 302.2 (262.7 to 385.4) | 0.9 (0.7 to 1.0) | 11842.0 (7722.1 to 17880.0) | 23.2 (15.0 to 35.9) | 0.7 (0.4 to 1.0) |
| Central Asia | 261669.6 (242022.1 to 287754.8) | 289.2 (268.5 to 315.8) | -0.6 (-0.7 to -0.5) | 18590.9 (12765.5 to 25155.2) | 20.4 (14.0 to 27.4) | -1.4 (-1.6 to -1.2) |
| Central Europe | 858105.8 (793347.3 to 919330.2) | 466.4 (433.5 to 499.5) | -1.0 (-1.1 to -0.9) | 33167.4 (21553.5 to 48354.7) | 18.3 (11.8 to 26.8) | -2.9 (-3.0 to -2.8) |
| Central Latin America | 795095.9 (727668.2 to 897037.4) | 308.5 (282.4 to 346.4) | -0.8 (-1.0 to -0.7) | 54021.3 (37701.4 to 72512.2) | 20.9 (14.6 to 28.0) | -2.0 (-2.1 to -1.8) |
| Central Sub-Saharan Africa | 202487.5 (151048.3 to 294714.2) | 255.9 (198.4 to 357.7) | 0.4 (0.1 to 0.8) | 22423.6 (13377.2 to 35213.9) | 27.7 (17.0 to 42.4) | -0.1 (-0.5 to 0.3) |
| East Asia | 5179213.1 (4797321.1 to 5539596.0) | 258.2 (239.4 to 275.9) | -0.2 (-0.3 to 0.0) | 212456.3 (140322.5 to 307572.0) | 10.6 (7.0 to 15.4) | -3.2 (-3.4 to -2.9) |
| Eastern Europe | 1561507.5 (1447516.5 to 1665768.8) | 516.7 (479.5 to 552.3) | -1.0 (-1.3 to -0.7) | 69804.8 (46536.7 to 99156.9) | 23.3 (15.5 to 33.2) | -2.5 (-3.0 to -2.1) |
| Eastern Sub-Saharan Africa | 613393.3 (429043.3 to 947785.4) | 262.8 (188.6 to 399.0) | -0.3 (-0.6 to 0.0) | 67154.3 (38826.4 to 113971.7) | 28.1 (16.8 to 47.0) | -0.9 (-1.2 to -0.5) |
| High-income Asia Pacific | 59109.4 (55427.2 to 63377.4) | 17.0 (15.9 to 18.2) | -1.2 (-1.3 to -1.1) | 2127.7 (1343.3 to 3144.4) | 0.6 (0.4 to 0.9) | -1.9 (-2.0 to -1.8) |
| High-income North America | 117704.8 (109337.1 to 125976.0) | 20.6 (19.2 to 22.0) | -0.5 (-0.6 to -0.4) | 4166.0 (2654.0 to 6217.4) | 0.7 (0.5 to 1.1) | -0.7 (-0.8 to -0.5) |
| North Africa and Middle East | 2345186.9 (1789043.4 to 3214708.4) | 426.1 (330.5 to 573.7) | -0.2 (-0.3 to -0.1) | 155115.6 (90640.5 to 246000.6) | 27.6 (16.5 to 43.0) | -1.7 (-1.8 to -1.6) |
| Oceania | 18837.9 (17451.7 to 21337.4) | 205.9 (191.9 to 228.2) | 0.4 (0.3 to 0.5) | 1971.3 (1333.6 to 2652.9) | 21.0 (14.3 to 28.0) | 0.2 (0.2 to 0.3) |
| South Asia | 3833568.1 (3518377.2 to 4192954.6) | 254.1 (231.7 to 278.5) | -0.1 (-0.2 to -0.1) | 329804.8 (232423.8 to 439557.7) | 21.5 (15.1 to 28.7) | -1.1 (-1.3 to -1.0) |
| Southeast Asia | 1529240.0 (1337897.1 to 1840931.3) | 225.0 (197.4 to 265.8) | -0.5 (-0.5 to -0.5) | 117097.1 (80070.9 to 163373.3) | 17.0 (11.7 to 23.3) | -1.6 (-1.7 to -1.5) |
| Southern Latin America | 17775.0 (16826.3 to 18780.4) | 22.3 (21.1 to 23.6) | -0.3 (-0.3 to -0.2) | 945.4 (621.3 to 1314.3) | 1.2 (0.8 to 1.7) | -1.6 (-1.7 to -1.5) |
| Southern Sub-Saharan Africa | 145924.2 (136556.6 to 157415.9) | 217.3 (203.9 to 234.2) | -1.4 (-1.5 to -1.2) | 12720.3 (8837.4 to 17027.8) | 18.7 (13.1 to 25.0) | -1.9 (-2.1 to -1.7) |
| Tropical Latin America | 826698.5 (763522.9 to 913772.1) | 325.0 (299.9 to 360.1) | -0.7 (-0.7 to -0.6) | 56785.2 (39444.0 to 76648.7) | 22.3 (15.5 to 30.1) | -1.7 (-1.8 to -1.7) |
| Western Europe | 137012.3 (126655.6 to 147982.1) | 18.4 (17.0 to 19.7) | -0.9 (-1.0 to -0.9) | 4898.8 (3084.2 to 7393.6) | 0.7 (0.4 to 1.0) | -1.2 (-1.2 to -1.1) |
| Western Sub-Saharan Africa | 444712.5 (392246.4 to 523652.6) | 168.5 (152.4 to 192.3) | 0.2 (0.1 to 0.3) | 45579.6 (31207.7 to 62519.5) | 16.8 (11.5 to 22.6) | -0.3 (-0.5 to -0.2) |

**Table S7. Cases and ASR of prevalence and YLDs in 2021, and their EAPC from 1990 to 2021 for AMP-LL2, globally and by 21 GBD regions.**

| **Characteristics** | **Prevalence** | | | **YLDs** | | |
| --- | --- | --- | --- | --- | --- | --- |
|  | **Cases No. (95% UI)** | **ASR per 100,000 No. (95% UI)** | **EAPC of prevalence rate No. (95% CI)** | **Cases No. (95% UI)** | **ASR per 100,000 No. (95% UI)** | **EAPC of YLDs rate No. (95% CI)** |
| Global | 3795491.8 (3169630.2 to 4964859.5) | 45.4 (37.9 to 59.4) | -0.8 (-0.8 to -0.7) | 628584.6 (409123.0 to 966723.6) | 7.6 (4.9 to 11.6) | -1.4 (-1.5 to -1.3) |
| Andean Latin America | 24881.2 (20125.8 to 33635.6) | 38.2 (30.9 to 51.8) | -0.6 (-0.7 to -0.5) | 4250.8 (2755.9 to 6423.2) | 6.5 (4.2 to 9.9) | -1.9 (-2.0 to -1.8) |
| Australasia | 34893.6 (30370.0 to 40957.0) | 93.3 (80.5 to 110.5) | -0.5 (-0.6 to -0.3) | 3972.9 (2539.7 to 5645.6) | 10.8 (6.8 to 15.5) | -0.5 (-0.6 to -0.4) |
| Caribbean | 17290.8 (15836.8 to 19057.0) | 34.0 (31.1 to 37.5) | -0.1 (-0.1 to 0.0) | 3233.1 (2147.5 to 4403.9) | 6.4 (4.2 to 8.7) | -0.5 (-0.5 to -0.4) |
| Central Asia | 44926.8 (38003.4 to 55770.0) | 47.1 (40.0 to 58.2) | -0.6 (-0.8 to -0.5) | 8273.9 (5539.6 to 12086.0) | 8.6 (5.8 to 12.5) | -1.2 (-1.5 to -1.0) |
| Central Europe | 103400.3 (92934.6 to 117324.0) | 67.8 (60.6 to 77.4) | -0.8 (-0.9 to -0.7) | 12311.2 (8074.7 to 17320.0) | 8.2 (5.4 to 11.7) | -2.1 (-2.2 to -2.1) |
| Central Latin America | 141402.1 (114866.8 to 189080.9) | 53.7 (43.8 to 71.4) | -0.7 (-0.9 to -0.5) | 24768.5 (16376.3 to 37061.0) | 9.4 (6.2 to 14.0) | -1.7 (-1.9 to -1.5) |
| Central Sub-Saharan Africa | 55326.9 (29359.0 to 109199.1) | 56.0 (29.8 to 109.9) | 1.2 (0.7 to 1.7) | 14183.6 (6615.5 to 30140.3) | 14.1 (6.6 to 29.6) | 0.7 (0.2 to 1.3) |
| East Asia | 575346.1 (521115.4 to 642676.4) | 30.9 (28.0 to 34.5) | -0.9 (-1.2 to -0.7) | 71391.6 (48424.2 to 99867.2) | 3.9 (2.6 to 5.4) | -3.1 (-3.4 to -2.8) |
| Eastern Europe | 193344.2 (174890.4 to 214491.7) | 71.8 (65.0 to 79.9) | -1.0 (-1.2 to -0.7) | 25005.8 (16831.6 to 34822.6) | 9.4 (6.3 to 13.1) | -2.1 (-2.4 to -1.7) |
| Eastern Sub-Saharan Africa | 190673.2 (95148.5 to 398016.5) | 65.4 (32.0 to 137.2) | 0.3 (-0.1 to 0.7) | 48906.6 (21448.3 to 107915.7) | 16.5 (7.0 to 36.9) | -0.2 (-0.6 to 0.2) |
| High-income Asia Pacific | 134395.0 (121736.1 to 152598.0) | 51.0 (45.7 to 58.7) | -1.2 (-1.4 to -1.1) | 15253.3 (10095.1 to 21741.9) | 5.9 (3.9 to 8.6) | -1.7 (-1.8 to -1.7) |
| High-income North America | 241700.5 (220392.0 to 263384.9) | 49.0 (44.7 to 53.7) | -1.2 (-1.4 to -1.0) | 26833.4 (18084.0 to 37445.9) | 5.5 (3.7 to 7.7) | -1.3 (-1.5 to -1.0) |
| North Africa and Middle East | 541260.6 (305053.5 to 1032539.9) | 89.0 (50.8 to 168.3) | 0.2 (0.1 to 0.3) | 97724.7 (46782.0 to 208924.8) | 15.9 (7.7 to 33.6) | -0.8 (-0.9 to -0.7) |
| Oceania | 3266.1 (2951.0 to 3635.0) | 28.9 (26.2 to 31.9) | 0.3 (0.3 to 0.4) | 807.3 (542.5 to 1104.7) | 7.0 (4.8 to 9.6) | 0.2 (0.1 to 0.2) |
| South Asia | 568875.7 (485886.5 to 690765.5) | 32.3 (27.9 to 38.7) | -0.5 (-0.5 to -0.4) | 121300.5 (81808.6 to 171045.7) | 6.8 (4.6 to 9.5) | -1.3 (-1.4 to -1.2) |
| Southeast Asia | 255359.3 (187877.1 to 404081.3) | 34.6 (25.8 to 53.7) | -0.7 (-0.8 to -0.7) | 50121.9 (30672.6 to 83837.5) | 6.8 (4.2 to 11.1) | -1.6 (-1.6 to -1.5) |
| Southern Latin America | 50279.3 (45795.6 to 56782.9) | 66.1 (59.9 to 74.8) | 0.0 (-0.2 to 0.1) | 7304.4 (4939.7 to 10221.1) | 9.6 (6.5 to 13.5) | -1.1 (-1.2 to -0.9) |
| Southern Sub-Saharan Africa | 23641.5 (20698.3 to 29370.9) | 31.9 (28.0 to 39.6) | -1.3 (-1.4 to -1.1) | 4986.0 (3379.6 to 7056.2) | 6.7 (4.5 to 9.4) | -1.7 (-1.9 to -1.5) |
| Tropical Latin America | 114503.0 (98243.5 to 139167.3) | 46.0 (39.2 to 56.8) | -0.7 (-0.8 to -0.6) | 20241.3 (13818.5 to 29062.2) | 8.1 (5.6 to 11.9) | -1.5 (-1.5 to -1.4) |
| Western Europe | 379733.0 (337352.9 to 437389.6) | 66.2 (58.5 to 77.4) | -0.6 (-0.7 to -0.5) | 42899.8 (28200.5 to 60637.7) | 7.6 (5.0 to 10.8) | -0.8 (-0.9 to -0.7) |
| Western Sub-Saharan Africa | 100992.8 (76964.8 to 145746.3) | 27.6 (21.6 to 38.9) | 0.6 (0.4 to 0.7) | 24814.0 (15564.2 to 40255.1) | 6.6 (4.2 to 10.5) | 0.2 (0.0 to 0.3) |

**Table S8. Cases and ASR of prevalence and YLDs in 2021, and their EAPC from 1990 to 2021 for AMP-Th, globally and by 21 GBD regions.**

| **Characteristics** | **Prevalence** | | | **YLDs** | | |
| --- | --- | --- | --- | --- | --- | --- |
|  | **Cases No. (95% UI)** | **ASR per 100,000 No. (95% UI)** | **EAPC of prevalence rate No. (95% CI)** | **Cases No. (95% UI)** | **ASR per 100,000 No. (95% UI)** | **EAPC of YLDs rate No. (95% CI)** |
| Global | 99830660.7 (90285572.0 to 112852542.0) | 1197.2 (1081.7 to 1355.4) | -1.2 (-1.2 to -1.1) | 1093176.4 (520575.9 to 2020114.6) | 13.1 (6.2 to 24.3) | -1.2 (-1.2 to -1.1) |
| Andean Latin America | 917620.9 (833697.0 to 1033237.4) | 1398.5 (1272.8 to 1572.0) | -0.5 (-0.6 to -0.5) | 10120.4 (4833.5 to 18824.4) | 15.4 (7.4 to 28.6) | -0.5 (-0.6 to -0.5) |
| Australasia | 1678025.6 (1496479.6 to 1906526.0) | 4572.0 (4035.5 to 5223.7) | -0.6 (-0.7 to -0.5) | 18315.9 (8507.0 to 33978.9) | 50.3 (23.3 to 94.0) | -0.6 (-0.7 to -0.5) |
| Caribbean | 808730.7 (731636.3 to 903012.0) | 1602.3 (1447.8 to 1792.1) | 0.4 (0.3 to 0.5) | 8868.5 (4214.1 to 16418.0) | 17.6 (8.4 to 32.7) | 0.4 (0.3 to 0.5) |
| Central Asia | 1790188.5 (1626523.7 to 1998906.3) | 1874.1 (1704.8 to 2091.5) | -0.8 (-0.8 to -0.7) | 19794.4 (9417.9 to 37038.9) | 20.7 (9.8 to 38.7) | -0.8 (-0.8 to -0.7) |
| Central Europe | 4924787.3 (4472115.2 to 5462297.1) | 3325.2 (3009.2 to 3716.3) | -0.8 (-0.9 to -0.7) | 53615.0 (25460.6 to 100368.2) | 36.6 (17.2 to 69.0) | -0.8 (-0.9 to -0.7) |
| Central Latin America | 4646090.0 (4165429.6 to 5268047.5) | 1770.7 (1587.9 to 2007.5) | -0.4 (-0.7 to 0.0) | 51060.2 (24259.3 to 95066.2) | 19.4 (9.2 to 36.2) | -0.4 (-0.7 to 0.0) |
| Central Sub-Saharan Africa | 904405.7 (717006.9 to 1262193.4) | 867.8 (692.8 to 1204.2) | -0.1 (-0.3 to 0.1) | 10005.3 (4758.3 to 19648.8) | 9.5 (4.6 to 19.0) | -0.1 (-0.3 to 0.2) |
| East Asia | 13136656.4 (11610333.2 to 14996479.6) | 724.4 (635.2 to 832.6) | -0.6 (-0.9 to -0.3) | 144229.4 (68118.5 to 267743.8) | 8.0 (3.7 to 14.9) | -0.7 (-0.9 to -0.4) |
| Eastern Europe | 6682431.1 (6090230.8 to 7450318.6) | 2540.4 (2308.3 to 2830.9) | -1.5 (-1.7 to -1.3) | 72585.5 (34749.9 to 134488.1) | 27.8 (13.2 to 51.9) | -1.5 (-1.7 to -1.3) |
| Eastern Sub-Saharan Africa | 2934297.7 (2389080.2 to 3922895.3) | 922.0 (753.5 to 1256.2) | -0.5 (-0.6 to -0.3) | 32501.4 (15866.7 to 64216.3) | 10.1 (4.9 to 20.3) | -0.5 (-0.6 to -0.3) |
| High-income Asia Pacific | 4752340.7 (4349160.6 to 5228396.4) | 1880.0 (1712.6 to 2089.8) | -1.2 (-1.3 to -1.1) | 51809.2 (24651.6 to 94349.0) | 20.8 (9.9 to 38.2) | -1.2 (-1.3 to -1.1) |
| High-income North America | 6321143.3 (5822002.4 to 6883057.9) | 1355.6 (1248.4 to 1482.3) | -1.8 (-2.1 to -1.4) | 68087.7 (32395.4 to 125613.0) | 14.8 (7.0 to 27.2) | -1.8 (-2.1 to -1.4) |
| North Africa and Middle East | 9282445.9 (7769404.6 to 11878779.8) | 1532.5 (1288.8 to 1942.3) | -0.4 (-0.4 to -0.4) | 102348.3 (50170.2 to 197206.9) | 16.8 (8.3 to 32.3) | -0.4 (-0.4 to -0.4) |
| Oceania | 83726.2 (73402.3 to 97759.0) | 711.0 (629.9 to 819.5) | 0.2 (0.1 to 0.3) | 924.1 (437.4 to 1693.3) | 7.8 (3.7 to 14.3) | 0.2 (0.1 to 0.3) |
| South Asia | 14021874.6 (12241081.8 to 16100572.0) | 781.5 (686.0 to 893.8) | -0.9 (-1.0 to -0.8) | 153438.3 (74124.3 to 285426.6) | 8.5 (4.1 to 15.8) | -0.9 (-1.0 to -0.8) |
| Southeast Asia | 6334063.3 (5607047.5 to 7416192.9) | 865.9 (769.1 to 1011.9) | -0.8 (-0.8 to -0.7) | 70000.7 (33232.0 to 131480.3) | 9.6 (4.5 to 18.0) | -0.8 (-0.8 to -0.7) |
| Southern Latin America | 2674995.2 (2391797.7 to 2999713.4) | 3532.6 (3158.5 to 3976.5) | -0.2 (-0.4 to -0.1) | 29351.5 (13827.1 to 54840.5) | 38.9 (18.3 to 72.8) | -0.2 (-0.4 to -0.1) |
| Southern Sub-Saharan Africa | 589918.5 (540573.6 to 654409.9) | 777.9 (714.5 to 861.5) | -1.2 (-1.4 to -1.1) | 6458.1 (3015.8 to 12021.1) | 8.5 (4.0 to 15.7) | -1.2 (-1.4 to -1.1) |
| Tropical Latin America | 3531609.3 (3143156.8 to 4007960.4) | 1411.4 (1254.7 to 1602.7) | -1.1 (-1.3 to -0.9) | 38572.2 (17946.2 to 71701.8) | 15.4 (7.2 to 28.7) | -1.1 (-1.3 to -0.9) |
| Western Europe | 11090966.6 (9929442.5 to 12641578.9) | 2013.5 (1780.1 to 2321.3) | -0.7 (-0.8 to -0.6) | 120856.2 (56789.6 to 226383.3) | 22.2 (10.4 to 41.7) | -0.7 (-0.8 to -0.6) |
| Western Sub-Saharan Africa | 2724343.1 (2405361.9 to 3128950.1) | 724.4 (648.8 to 824.4) | -0.4 (-0.5 to -0.3) | 30234.2 (14804.8 to 56249.6) | 8.0 (3.9 to 14.8) | -0.4 (-0.5 to -0.3) |

**Table S9. Cases and ASR of prevalence and YLDs in 2021, and their EAPC from 1990 to 2021 for AMP-Toe, globally and by 21 GBD regions.**

| **Characteristics** | **Prevalence** | | | **YLDs** | | |
| --- | --- | --- | --- | --- | --- | --- |
|  | **Cases No. (95% UI)** | **ASR per 100,000 No. (95% UI)** | **EAPC of prevalence rate No. (95% CI)** | **Cases No. (95% UI)** | **ASR per 100,000 No. (95% UI)** | **EAPC of YLDs rate No. (95% CI)** |
| Global | 84609371.9 (75613661.6 to 96295253.5) | 1015.5 (907.0 to 1156.8) | -1.0 (-1.0 to -1.0) | 475576.6 (190824.6 to 998735.3) | 5.7 (2.3 to 12.0) | -1.0 (-1.0 to -0.9) |
| Andean Latin America | 798800.0 (714329.5 to 904097.1) | 1217.0 (1088.1 to 1377.1) | -0.5 (-0.6 to -0.5) | 4517.4 (1830.6 to 9623.3) | 6.9 (2.8 to 14.6) | -0.5 (-0.6 to -0.5) |
| Australasia | 880261.1 (787281.9 to 1006728.4) | 2410.6 (2145.4 to 2781.8) | -0.5 (-0.6 to -0.4) | 4928.8 (2036.8 to 10318.6) | 13.6 (5.6 to 28.6) | -0.5 (-0.6 to -0.4) |
| Caribbean | 668689.6 (605114.5 to 752247.6) | 1324.8 (1197.4 to 1494.7) | 0.3 (0.2 to 0.4) | 3770.0 (1534.3 to 7807.7) | 7.5 (3.0 to 15.5) | 0.3 (0.2 to 0.4) |
| Central Asia | 1510763.9 (1358415.0 to 1689088.2) | 1582.7 (1425.8 to 1766.9) | -0.8 (-0.8 to -0.7) | 8574.0 (3527.6 to 17832.5) | 9.0 (3.7 to 18.6) | -0.8 (-0.8 to -0.7) |
| Central Europe | 4019010.4 (3620411.4 to 4477318.9) | 2718.5 (2435.2 to 3053.2) | -0.8 (-0.9 to -0.8) | 22464.2 (9331.4 to 46739.4) | 15.4 (6.3 to 32.1) | -0.8 (-0.9 to -0.7) |
| Central Latin America | 4083841.6 (3626102.3 to 4659986.0) | 1556.1 (1382.0 to 1771.9) | -0.4 (-0.7 to -0.1) | 23044.5 (9316.2 to 48872.0) | 8.8 (3.6 to 18.6) | -0.4 (-0.7 to -0.1) |
| Central Sub-Saharan Africa | 916595.0 (696975.7 to 1291519.3) | 887.5 (675.7 to 1263.7) | 0.1 (-0.2 to 0.3) | 5194.5 (1969.8 to 12535.7) | 5.0 (1.9 to 12.0) | 0.1 (-0.2 to 0.4) |
| East Asia | 13106148.2 (11536987.8 to 15039239.9) | 717.7 (629.1 to 827.3) | -0.7 (-1.0 to -0.4) | 73798.3 (30110.0 to 152764.8) | 4.1 (1.6 to 8.4) | -0.7 (-1.0 to -0.4) |
| Eastern Europe | 5925970.9 (5341594.7 to 6681605.7) | 2250.7 (2016.9 to 2530.3) | -1.4 (-1.6 to -1.2) | 33038.2 (13941.9 to 68057.0) | 12.7 (5.3 to 26.0) | -1.4 (-1.6 to -1.2) |
| Eastern Sub-Saharan Africa | 2816459.8 (2207146.1 to 4022399.4) | 894.3 (691.6 to 1304.0) | -0.5 (-0.6 to -0.3) | 15987.8 (6066.2 to 38261.4) | 5.0 (1.9 to 11.9) | -0.5 (-0.6 to -0.3) |
| High-income Asia Pacific | 2728550.3 (2484964.7 to 3027752.3) | 1086.9 (981.0 to 1222.2) | -1.2 (-1.3 to -1.1) | 15261.4 (6305.7 to 31563.5) | 6.2 (2.5 to 12.9) | -1.2 (-1.3 to -1.1) |
| High-income North America | 3967836.3 (3679881.8 to 4303921.7) | 845.4 (783.3 to 920.5) | -1.5 (-1.8 to -1.2) | 21914.1 (9131.5 to 44686.2) | 4.7 (2.0 to 9.7) | -1.5 (-1.9 to -1.2) |
| North Africa and Middle East | 9034178.1 (7275062.2 to 12355507.3) | 1494.5 (1204.8 to 2035.2) | -0.3 (-0.4 to -0.3) | 51048.7 (20151.9 to 122391.5) | 8.4 (3.3 to 20.2) | -0.3 (-0.4 to -0.3) |
| Oceania | 79550.1 (69971.5 to 91907.9) | 678.4 (604.2 to 775.1) | 0.3 (0.2 to 0.4) | 451.7 (184.2 to 961.6) | 3.8 (1.6 to 8.1) | 0.3 (0.2 to 0.4) |
| South Asia | 13580545.1 (11898982.7 to 15604741.1) | 759.7 (669.0 to 868.1) | -0.8 (-0.9 to -0.7) | 76206.9 (30828.5 to 159269.9) | 4.2 (1.7 to 8.8) | -0.8 (-0.9 to -0.7) |
| Southeast Asia | 5943077.0 (5175225.6 to 7177090.5) | 812.3 (710.2 to 973.0) | -0.8 (-0.8 to -0.7) | 33709.0 (13588.1 to 74082.5) | 4.6 (1.9 to 10.0) | -0.8 (-0.8 to -0.7) |
| Southern Latin America | 1338598.8 (1215400.4 to 1503948.2) | 1776.7 (1608.8 to 2004.8) | -0.1 (-0.3 to 0.0) | 7540.1 (3134.4 to 15756.2) | 10.0 (4.2 to 21.1) | -0.1 (-0.3 to 0.0) |
| Southern Sub-Saharan Africa | 548750.3 (499857.7 to 609219.5) | 725.8 (663.3 to 806.2) | -1.3 (-1.4 to -1.1) | 3083.3 (1255.3 to 6388.7) | 4.1 (1.7 to 8.4) | -1.3 (-1.4 to -1.1) |
| Tropical Latin America | 3277294.8 (2871043.5 to 3755590.0) | 1310.2 (1144.1 to 1504.0) | -1.1 (-1.3 to -0.9) | 18369.4 (7576.7 to 37143.2) | 7.4 (3.0 to 15.0) | -1.1 (-1.3 to -0.9) |
| Western Europe | 6845121.1 (5986585.6 to 8019575.6) | 1245.6 (1082.4 to 1497.1) | -0.6 (-0.7 to -0.6) | 38221.7 (15751.4 to 79666.4) | 7.0 (2.9 to 14.8) | -0.6 (-0.7 to -0.6) |
| Western Sub-Saharan Africa | 2539329.9 (2219871.1 to 2942618.6) | 674.6 (597.1 to 775.0) | -0.3 (-0.4 to -0.2) | 14453.0 (5822.4 to 31410.9) | 3.8 (1.5 to 8.2) | -0.3 (-0.4 to -0.2) |

**Table S10. Cases and ASR of prevalence and YLDs in 2021, and their EAPC from 1990 to 2021 for AMP-UL1, globally and by 21 GBD regions.**

| **Characteristics** | **Prevalence** | | | **YLDs** | | |
| --- | --- | --- | --- | --- | --- | --- |
|  | **Cases No. (95% UI)** | **ASR per 100,000 No. (95% UI)** | **EAPC of prevalence rate No. (95% CI)** | **Cases No. (95% UI)** | **ASR per 100,000 No. (95% UI)** | **EAPC of YLDs rate No. (95% CI)** |
| Global | 6799995.8 (6034872.3 to 8169092.7) | 81.2 (72.0 to 97.4) | -0.7 (-0.8 to -0.7) | 364149.0 (239439.6 to 525869.3) | 4.4 (2.9 to 6.3) | -1.5 (-1.6 to -1.4) |
| Andean Latin America | 56427.2 (49773.3 to 67284.4) | 86.8 (76.7 to 103.7) | -0.6 (-0.7 to -0.5) | 3109.3 (2051.4 to 4405.0) | 4.8 (3.2 to 6.8) | -1.8 (-1.9 to -1.7) |
| Australasia | 38530.1 (34428.5 to 43589.5) | 101.2 (89.5 to 115.9) | -0.6 (-0.7 to -0.5) | 1415.5 (880.2 to 2155.0) | 3.8 (2.3 to 5.7) | -0.6 (-0.7 to -0.5) |
| Caribbean | 60084.5 (46947.2 to 88419.3) | 120.3 (92.8 to 179.2) | 1.5 (1.2 to 1.9) | 4037.9 (2339.2 to 6713.9) | 8.2 (4.7 to 13.8) | 1.7 (1.2 to 2.2) |
| Central Asia | 104243.5 (94842.3 to 118437.1) | 109.9 (100.2 to 124.4) | -0.8 (-0.9 to -0.7) | 6034.6 (4030.0 to 8536.8) | 6.3 (4.3 to 9.0) | -1.4 (-1.6 to -1.2) |
| Central Europe | 260382.4 (240336.3 to 288049.0) | 167.9 (154.5 to 187.2) | -0.9 (-1.0 to -0.8) | 10001.8 (6337.0 to 15101.6) | 6.5 (4.1 to 9.9) | -2.2 (-2.2 to -2.2) |
| Central Latin America | 301430.5 (268660.8 to 354298.2) | 114.8 (102.5 to 134.5) | -0.6 (-0.8 to -0.4) | 16875.5 (11319.4 to 23745.4) | 6.4 (4.3 to 9.0) | -1.5 (-1.7 to -1.3) |
| Central Sub-Saharan Africa | 86225.2 (58426.8 to 143743.1) | 88.8 (60.8 to 148.0) | 0.5 (0.2 to 0.8) | 6988.7 (4085.0 to 12444.4) | 7.1 (4.2 to 12.7) | 0.1 (-0.3 to 0.4) |
| East Asia | 1252215.5 (1159342.1 to 1357761.5) | 66.2 (61.3 to 72.3) | -0.8 (-0.9 to -0.6) | 49915.3 (31765.2 to 74479.5) | 2.7 (1.7 to 4.0) | -2.9 (-3.2 to -2.6) |
| Eastern Europe | 450566.6 (414911.8 to 490015.6) | 164.9 (151.1 to 180.0) | -1.2 (-1.5 to -1.0) | 18790.2 (12227.3 to 27582.5) | 6.9 (4.5 to 10.2) | -2.3 (-2.7 to -1.9) |
| Eastern Sub-Saharan Africa | 282138.4 (180919.9 to 502522.5) | 96.7 (60.4 to 173.9) | 0.0 (-0.3 to 0.3) | 22734.3 (12940.9 to 43250.0) | 7.7 (4.3 to 14.8) | -0.5 (-0.8 to -0.2) |
| High-income Asia Pacific | 146973.8 (135843.7 to 161330.2) | 54.1 (49.8 to 60.3) | -1.3 (-1.4 to -1.2) | 5388.3 (3361.5 to 8291.6) | 2.0 (1.3 to 3.1) | -1.7 (-1.8 to -1.7) |
| High-income North America | 265344.1 (245489.0 to 284000.8) | 53.1 (49.2 to 56.8) | -1.2 (-1.4 to -1.0) | 9558.9 (5964.6 to 14392.0) | 1.9 (1.2 to 2.9) | -1.3 (-1.5 to -1.0) |
| North Africa and Middle East | 851912.4 (600497.5 to 1377039.1) | 142.1 (101.3 to 228.7) | -0.1 (-0.2 to -0.1) | 47364.9 (27405.5 to 88418.2) | 7.8 (4.6 to 14.5) | -1.2 (-1.3 to -1.1) |
| Oceania | 7578.2 (6769.4 to 9134.9) | 67.4 (60.8 to 79.5) | 0.5 (0.4 to 0.6) | 594.0 (388.8 to 866.4) | 5.2 (3.4 to 7.4) | 0.4 (0.2 to 0.5) |
| South Asia | 1162676.0 (1041373.9 to 1332582.5) | 67.0 (60.4 to 76.0) | -0.5 (-0.6 to -0.5) | 78615.0 (54267.3 to 108879.6) | 4.5 (3.1 to 6.2) | -1.3 (-1.4 to -1.2) |
| Southeast Asia | 529266.1 (438558.5 to 710132.6) | 72.8 (60.6 to 96.7) | -0.5 (-0.6 to -0.4) | 33044.4 (21515.7 to 49605.3) | 4.5 (3.0 to 6.7) | -1.3 (-1.4 to -1.2) |
| Southern Latin America | 59170.2 (54610.6 to 64930.9) | 77.3 (71.2 to 85.0) | -0.1 (-0.3 to 0.0) | 2778.3 (1837.0 to 4025.8) | 3.6 (2.4 to 5.3) | -1.1 (-1.3 to -1.0) |
| Southern Sub-Saharan Africa | 53251.5 (48505.1 to 59356.3) | 72.4 (65.9 to 80.5) | -1.3 (-1.5 to -1.2) | 3599.0 (2471.0 to 5077.8) | 4.9 (3.4 to 6.8) | -1.7 (-2.0 to -1.5) |
| Tropical Latin America | 248741.1 (227997.2 to 275579.0) | 98.8 (90.3 to 109.6) | -0.9 (-1.0 to -0.8) | 14020.8 (9554.4 to 19647.2) | 5.6 (3.8 to 7.8) | -1.7 (-1.7 to -1.6) |
| Western Europe | 385958.3 (349901.0 to 435029.7) | 65.6 (59.5 to 74.9) | -0.8 (-0.8 to -0.7) | 14089.0 (8759.4 to 21260.0) | 2.4 (1.5 to 3.7) | -0.9 (-1.0 to -0.8) |
| Western Sub-Saharan Africa | 196880.2 (168929.9 to 246375.2) | 56.1 (49.1 to 68.7) | 0.0 (-0.1 to 0.1) | 15193.6 (9909.1 to 22009.6) | 4.3 (2.8 to 6.1) | -0.4 (-0.5 to -0.3) |

**Table S11. Cases and ASR of prevalence and YLDs in 2021, and their EAPC from 1990 to 2021 for AMP-UL2, globally and by 21 GBD regions.**

| **Characteristics** | **Prevalence** | | | **YLDs** | | |
| --- | --- | --- | --- | --- | --- | --- |
|  | **Cases No. (95% UI)** | **ASR per 100,000 No. (95% UI)** | **EAPC of prevalence rate No. (95% CI)** | **Cases No. (95% UI)** | **ASR per 100,000 No. (95% UI)** | **EAPC of YLDs rate No. (95% CI)** |
| Global | 7139997.2 (6309192.6 to 8588696.2) | 85.4 (75.4 to 102.9) | -0.8 (-0.8 to -0.7) | 1123374.3 (779923.9 to 1558792.5) | 13.5 (9.4 to 18.8) | -1.8 (-1.9 to -1.7) |
| Andean Latin America | 64141.0 (56377.0 to 76069.7) | 98.5 (86.7 to 116.7) | -0.6 (-0.7 to -0.5) | 10351.2 (7153.8 to 13699.4) | 15.8 (10.9 to 21.0) | -2.3 (-2.4 to -2.2) |
| Australasia | 33542.4 (29895.8 to 38813.9) | 89.1 (78.9 to 104.2) | -0.5 (-0.6 to -0.4) | 2736.2 (1776.4 to 3907.9) | 7.4 (4.8 to 10.7) | -0.6 (-0.7 to -0.4) |
| Caribbean | 66665.2 (54459.3 to 90959.2) | 133.0 (107.7 to 183.9) | 1.3 (1.1 to 1.5) | 13979.6 (8530.4 to 22310.7) | 28.3 (17.1 to 45.7) | 1.3 (0.9 to 1.8) |
| Central Asia | 122645.7 (110682.1 to 138941.2) | 128.9 (116.6 to 145.7) | -0.8 (-0.9 to -0.7) | 21304.1 (14938.8 to 28267.5) | 22.3 (15.7 to 29.5) | -1.7 (-1.9 to -1.5) |
| Central Europe | 312450.7 (284803.7 to 346235.0) | 203.9 (184.7 to 227.2) | -0.8 (-0.9 to -0.7) | 27894.2 (18946.5 to 38155.6) | 18.4 (12.4 to 25.2) | -3.0 (-3.0 to -2.9) |
| Central Latin America | 348776.2 (312063.9 to 408335.8) | 132.7 (118.9 to 154.9) | -0.6 (-0.8 to -0.3) | 57841.0 (40949.6 to 77618.8) | 22.0 (15.6 to 29.5) | -1.8 (-2.0 to -1.6) |
| Central Sub-Saharan Africa | 91177.8 (61053.2 to 155228.5) | 92.6 (62.0 to 157.8) | 0.5 (0.2 to 0.9) | 25162.4 (14467.3 to 42829.6) | 25.1 (14.5 to 43.2) | 0.0 (-0.4 to 0.4) |
| East Asia | 1244348.4 (1148229.2 to 1362621.2) | 66.5 (61.3 to 73.1) | -0.8 (-1.1 to -0.6) | 118391.7 (82264.1 to 164424.8) | 6.4 (4.4 to 8.8) | -4.1 (-4.4 to -3.7) |
| Eastern Europe | 506206.4 (466200.9 to 554760.2) | 186.6 (171.4 to 205.2) | -1.3 (-1.6 to -1.1) | 52581.6 (36009.1 to 70819.7) | 19.6 (13.4 to 26.4) | -3.1 (-3.5 to -2.6) |
| Eastern Sub-Saharan Africa | 316100.9 (203935.4 to 565785.3) | 107.3 (67.7 to 195.1) | 0.0 (-0.3 to 0.3) | 86278.2 (48316.8 to 155058.0) | 28.8 (15.6 to 52.6) | -0.6 (-1.0 to -0.3) |
| High-income Asia Pacific | 129944.6 (119427.5 to 143939.4) | 48.9 (44.6 to 55.1) | -1.2 (-1.3 to -1.1) | 10571.3 (6931.5 to 14646.5) | 4.1 (2.7 to 5.7) | -2.1 (-2.2 to -2.0) |
| High-income North America | 232866.8 (214315.1 to 253505.2) | 47.1 (43.3 to 51.0) | -1.1 (-1.3 to -0.9) | 18585.6 (12220.2 to 25761.4) | 3.8 (2.5 to 5.3) | -1.3 (-1.5 to -1.0) |
| North Africa and Middle East | 916561.4 (639132.3 to 1529727.7) | 152.2 (106.8 to 251.3) | -0.1 (-0.1 to 0.0) | 150430.2 (87906.8 to 266413.6) | 24.6 (14.4 to 43.5) | -1.6 (-1.6 to -1.5) |
| Oceania | 8020.5 (7039.5 to 9529.4) | 70.9 (62.8 to 83.0) | 0.6 (0.4 to 0.7) | 2120.3 (1433.2 to 2840.4) | 18.4 (12.7 to 24.5) | 0.4 (0.3 to 0.5) |
| South Asia | 1214300.7 (1089573.3 to 1382630.5) | 69.4 (62.4 to 78.2) | -0.6 (-0.7 to -0.5) | 263767.3 (184618.8 to 348909.7) | 14.9 (10.4 to 19.6) | -1.7 (-1.8 to -1.5) |
| Southeast Asia | 563743.7 (463326.8 to 750672.1) | 77.3 (63.9 to 101.9) | -0.6 (-0.6 to -0.5) | 109366.6 (72871.7 to 163771.6) | 14.9 (10.0 to 22.2) | -1.6 (-1.7 to -1.5) |
| Southern Latin America | 50363.4 (46348.3 to 55821.0) | 66.1 (60.9 to 73.5) | -0.1 (-0.3 to 0.1) | 6299.8 (4368.5 to 8436.7) | 8.3 (5.8 to 11.2) | -1.6 (-1.7 to -1.5) |
| Southern Sub-Saharan Africa | 59239.8 (53861.8 to 66304.0) | 79.8 (72.5 to 89.2) | -1.3 (-1.4 to -1.2) | 12764.3 (9038.8 to 17099.5) | 17.0 (12.1 to 22.7) | -1.9 (-2.1 to -1.6) |
| Tropical Latin America | 277588.2 (252034.1 to 314825.8) | 110.3 (99.7 to 125.9) | -0.9 (-1.0 to -0.8) | 46524.1 (32318.4 to 61272.7) | 18.5 (12.9 to 24.4) | -2.0 (-2.1 to -1.9) |
| Western Europe | 359466.8 (324748.5 to 407810.3) | 62.2 (55.9 to 71.8) | -0.7 (-0.8 to -0.6) | 29101.7 (18844.9 to 41324.1) | 5.1 (3.3 to 7.3) | -0.9 (-1.0 to -0.8) |
| Western Sub-Saharan Africa | 221846.7 (190860.4 to 274247.3) | 62.5 (54.7 to 75.7) | -0.1 (-0.2 to 0.0) | 57323.0 (39507.1 to 76452.9) | 15.8 (11.0 to 20.9) | -0.6 (-0.7 to -0.5) |

**Table S12. ASR of prevalence and YLDs in 1990 and 2021, and their EAPC from 1990 to 2021 for AMP-F, by country.**

| **Countries** | **Prevalence** | | | **YLDs** | | |
| --- | --- | --- | --- | --- | --- | --- |
|  | **Age-standardized prevalence rate, 1990** | **Age-standardized prevalence rate, 2021** | **EAPC of prevalence rate No. (95% CI)** | **Age-standardized YLDs rate, 1990** | **Age-standardized YLDs rate, 2021** | **EAPC of YLDs rate No. (95% CI)** |
| Afghanistan | 10006.4 (5372.0 to 20888.5) | 9838.8 (5493.2 to 18275.7) | -0.2 (-0.3 to -0.1) | 45.6 (13.2 to 116.5) | 44.5 (13.9 to 107.9) | -0.2 (-0.3 to -0.2) |
| Albania | 15707.9 (14289.2 to 17304.0) | 11975.5 (10939.4 to 13177.4) | -1.3 (-1.5 to -1.1) | 71.9 (26.4 to 159.0) | 54.9 (20.2 to 120.8) | -1.3 (-1.5 to -1.1) |
| Algeria | 4111.9 (3842.6 to 4424.2) | 3049.8 (2801.9 to 3343.9) | -0.9 (-1.0 to -0.9) | 18.8 (6.9 to 41.3) | 13.9 (5.1 to 30.7) | -0.9 (-1.0 to -0.9) |
| American Samoa | 2317.3 (2152.0 to 2503.1) | 1989.6 (1803.8 to 2216.0) | -0.4 (-0.5 to -0.3) | 10.6 (4.0 to 23.6) | 9.0 (3.3 to 19.7) | -0.4 (-0.5 to -0.3) |
| Andorra | 2707.1 (2426.5 to 3037.5) | 2515.6 (2242.2 to 2846.4) | -0.2 (-0.3 to -0.2) | 12.4 (4.5 to 27.5) | 11.5 (4.2 to 25.6) | -0.3 (-0.3 to -0.2) |
| Angola | 3898.0 (2828.3 to 5785.0) | 3096.0 (2130.2 to 4801.9) | -0.9 (-1.1 to -0.6) | 17.8 (6.1 to 40.2) | 14.1 (4.8 to 32.0) | -0.9 (-1.1 to -0.6) |
| Antigua and Barbuda | 5986.6 (5476.3 to 6605.2) | 6512.7 (5888.4 to 7200.1) | 0.2 (0.0 to 0.4) | 27.4 (9.9 to 59.9) | 29.7 (11.0 to 65.5) | 0.2 (0.0 to 0.4) |
| Argentina | 5221.0 (4678.1 to 5902.5) | 4499.0 (4014.1 to 5038.7) | -0.5 (-0.8 to -0.1) | 23.9 (8.8 to 53.6) | 20.6 (7.5 to 45.7) | -0.5 (-0.8 to -0.1) |
| Armenia | 9055.6 (8248.5 to 10122.7) | 5793.6 (5144.1 to 6550.8) | -1.6 (-1.8 to -1.5) | 41.6 (15.0 to 92.7) | 26.6 (9.5 to 58.4) | -1.6 (-1.8 to -1.5) |
| Australia | 7039.1 (6238.2 to 7986.8) | 5635.6 (4953.7 to 6437.5) | -0.7 (-0.7 to -0.6) | 32.2 (11.6 to 71.2) | 25.8 (9.3 to 57.4) | -0.7 (-0.7 to -0.6) |
| Austria | 3750.7 (3404.0 to 4166.1) | 2572.1 (2317.6 to 2893.7) | -1.2 (-1.3 to -1.2) | 17.2 (6.2 to 37.6) | 11.8 (4.3 to 26.1) | -1.2 (-1.3 to -1.2) |
| Azerbaijan | 5499.3 (5035.1 to 6040.8) | 5088.3 (4603.2 to 5661.5) | -0.4 (-0.5 to -0.3) | 25.3 (9.3 to 54.7) | 23.4 (8.5 to 52.2) | -0.4 (-0.5 to -0.3) |
| Bahamas | 3243.0 (2976.9 to 3559.7) | 3361.5 (3083.3 to 3673.9) | 0.1 (0.0 to 0.3) | 14.9 (5.5 to 33.1) | 15.4 (5.6 to 33.7) | 0.1 (0.0 to 0.3) |
| Bahrain | 3146.1 (2922.5 to 3422.7) | 2919.0 (2688.1 to 3177.9) | -0.3 (-0.4 to -0.2) | 14.4 (5.3 to 32.1) | 13.3 (5.0 to 29.5) | -0.3 (-0.4 to -0.2) |
| Bangladesh | 1597.1 (1479.3 to 1731.5) | 1647.2 (1458.3 to 1902.7) | -0.2 (-0.3 to 0.0) | 7.3 (2.6 to 15.6) | 7.5 (2.7 to 16.2) | -0.2 (-0.3 to 0.0) |
| Barbados | 3444.3 (3161.5 to 3816.3) | 3464.5 (3172.6 to 3818.2) | -0.1 (-0.3 to 0.1) | 15.8 (5.8 to 34.8) | 15.9 (5.8 to 34.9) | -0.1 (-0.3 to 0.1) |
| Belarus | 7913.4 (7283.7 to 8652.7) | 6763.0 (6158.3 to 7435.2) | -0.6 (-0.8 to -0.4) | 36.2 (13.6 to 78.4) | 30.9 (11.7 to 67.0) | -0.6 (-0.8 to -0.4) |
| Belgium | 2958.7 (2662.3 to 3322.8) | 2562.8 (2297.3 to 2876.9) | -0.2 (-0.8 to 0.3) | 13.6 (4.9 to 29.9) | 11.7 (4.3 to 25.8) | -0.2 (-0.8 to 0.3) |
| Belize | 4506.4 (4110.0 to 4955.5) | 5019.0 (4585.4 to 5493.5) | 0.2 (-0.1 to 0.4) | 20.7 (7.6 to 45.2) | 22.9 (8.3 to 50.2) | 0.1 (-0.1 to 0.4) |
| Benin | 2118.3 (1953.4 to 2262.1) | 1868.8 (1732.3 to 1993.7) | -0.4 (-0.5 to -0.3) | 9.6 (3.5 to 21.3) | 8.5 (3.1 to 18.8) | -0.4 (-0.4 to -0.3) |
| Bermuda | 3701.9 (3363.3 to 4121.0) | 3652.7 (3326.2 to 4019.8) | 0.0 (-0.1 to 0.2) | 17.0 (6.3 to 37.9) | 16.8 (6.2 to 37.3) | 0.0 (-0.1 to 0.2) |
| Bhutan | 2656.5 (2465.3 to 2851.9) | 2704.6 (2501.0 to 2952.6) | 0.0 (-0.1 to 0.1) | 12.1 (4.5 to 27.1) | 12.3 (4.6 to 27.4) | 0.0 (-0.1 to 0.1) |
| Bolivia (Plurinational State of) | 4179.6 (3884.3 to 4522.1) | 3480.0 (3224.7 to 3774.6) | -0.6 (-0.7 to -0.6) | 19.1 (7.1 to 42.4) | 15.9 (5.9 to 34.8) | -0.7 (-0.7 to -0.6) |
| Bosnia and Herzegovina | 14553.0 (13198.2 to 16038.8) | 10308.7 (9071.8 to 11720.5) | -1.4 (-1.6 to -1.1) | 66.5 (24.4 to 146.8) | 47.1 (17.2 to 103.0) | -1.4 (-1.6 to -1.1) |
| Botswana | 2197.2 (2038.7 to 2355.8) | 2140.1 (2002.2 to 2286.3) | -0.3 (-0.4 to -0.1) | 10.0 (3.7 to 22.0) | 9.7 (3.6 to 21.1) | -0.3 (-0.4 to -0.1) |
| Brazil | 4740.5 (4306.7 to 5236.0) | 3741.6 (3395.8 to 4169.6) | -1.1 (-1.3 to -0.9) | 21.5 (8.0 to 46.7) | 17.0 (6.4 to 37.9) | -1.1 (-1.2 to -0.9) |
| Brunei Darussalam | 3319.5 (3013.2 to 3663.2) | 2777.8 (2509.5 to 3087.7) | -0.6 (-0.6 to -0.5) | 15.2 (5.6 to 32.9) | 12.7 (4.7 to 27.3) | -0.6 (-0.6 to -0.5) |
| Bulgaria | 12584.6 (11623.0 to 13790.3) | 10809.2 (9874.6 to 11906.5) | -0.4 (-0.5 to -0.3) | 57.5 (21.3 to 128.1) | 49.4 (18.3 to 108.8) | -0.4 (-0.5 to -0.3) |
| Burkina Faso | 1887.5 (1742.6 to 2018.3) | 1833.7 (1690.3 to 1979.7) | -0.1 (-0.2 to -0.1) | 8.6 (3.1 to 18.6) | 8.4 (3.0 to 18.4) | -0.1 (-0.1 to -0.1) |
| Burundi | 2143.1 (1990.8 to 2294.6) | 5457.5 (3257.3 to 9176.6) | 3.3 (2.4 to 4.3) | 9.8 (3.6 to 21.4) | 25.1 (8.3 to 60.7) | 3.3 (2.4 to 4.3) |
| Cabo Verde | 2228.0 (2055.7 to 2371.3) | 1953.2 (1804.3 to 2095.5) | -0.4 (-0.5 to -0.4) | 10.2 (3.7 to 22.3) | 8.9 (3.3 to 19.5) | -0.4 (-0.5 to -0.4) |
| Cambodia | 4576.8 (3009.7 to 8710.0) | 3271.5 (2357.1 to 5324.3) | -0.9 (-1.1 to -0.7) | 21.0 (6.9 to 49.3) | 14.9 (5.1 to 33.8) | -0.9 (-1.1 to -0.7) |
| Cameroon | 1842.8 (1704.4 to 1981.9) | 1799.8 (1639.8 to 1988.4) | -0.1 (-0.2 to -0.1) | 8.4 (3.0 to 18.2) | 8.2 (3.0 to 17.8) | -0.1 (-0.2 to 0.0) |
| Canada | 2203.4 (1998.2 to 2466.7) | 1646.2 (1488.9 to 1833.9) | -1.0 (-1.0 to -1.0) | 10.1 (3.7 to 22.2) | 7.5 (2.7 to 16.5) | -1.0 (-1.0 to -1.0) |
| Central African Republic | 2008.0 (1884.3 to 2130.2) | 2502.2 (2049.7 to 3314.2) | 0.8 (0.6 to 0.9) | 9.1 (3.3 to 19.6) | 11.4 (4.2 to 25.1) | 0.8 (0.6 to 1.0) |
| Chad | 2782.9 (2235.2 to 3734.6) | 2774.5 (2261.9 to 3643.9) | 0.0 (-0.1 to 0.1) | 12.7 (4.6 to 27.8) | 12.6 (4.6 to 27.6) | 0.0 (-0.1 to 0.1) |
| Chile | 4308.4 (3893.6 to 4794.4) | 4841.3 (4344.5 to 5431.1) | 0.6 (0.3 to 0.9) | 19.7 (7.2 to 43.7) | 22.1 (8.2 to 48.7) | 0.6 (0.3 to 0.9) |
| China | 1921.0 (1755.5 to 2123.3) | 1805.6 (1623.6 to 2029.4) | -0.4 (-0.7 to -0.2) | 8.8 (3.3 to 19.6) | 8.3 (3.1 to 18.3) | -0.5 (-0.7 to -0.2) |
| Colombia | 6846.3 (6238.7 to 7529.3) | 4551.7 (4020.4 to 5131.3) | -1.1 (-1.2 to -1.0) | 31.3 (11.5 to 68.4) | 20.9 (7.6 to 46.0) | -1.1 (-1.2 to -1.0) |
| Comoros | 2209.7 (2062.3 to 2358.5) | 1930.8 (1773.8 to 2095.5) | -0.6 (-0.6 to -0.5) | 10.1 (3.7 to 21.9) | 8.8 (3.2 to 19.4) | -0.5 (-0.6 to -0.5) |
| Congo | 1887.8 (1768.4 to 2013.8) | 2193.2 (1720.9 to 3068.8) | 0.6 (0.1 to 1.1) | 8.6 (3.2 to 18.6) | 10.0 (3.6 to 21.9) | 0.6 (0.1 to 1.1) |
| Cook Islands | 1703.5 (1571.7 to 1858.9) | 1632.3 (1439.3 to 1859.2) | -0.1 (-0.3 to 0.1) | 7.8 (2.9 to 17.0) | 7.4 (2.7 to 16.5) | -0.1 (-0.3 to 0.0) |
| Costa Rica | 4481.2 (4020.3 to 5017.6) | 3977.0 (3564.3 to 4451.2) | -0.4 (-0.5 to -0.3) | 20.5 (7.5 to 45.4) | 18.2 (6.6 to 40.3) | -0.4 (-0.5 to -0.3) |
| Coted'Ivoire | 2109.2 (1951.0 to 2251.6) | 1934.4 (1789.1 to 2088.2) | -0.3 (-0.3 to -0.2) | 9.5 (3.5 to 20.7) | 8.8 (3.2 to 19.5) | -0.3 (-0.3 to -0.2) |
| Croatia | 11504.3 (10514.5 to 12619.8) | 8433.3 (7622.9 to 9322.5) | -1.0 (-1.1 to -0.9) | 52.7 (19.6 to 118.3) | 38.6 (14.1 to 85.5) | -1.0 (-1.1 to -0.9) |
| Cuba | 4117.8 (3798.7 to 4478.4) | 4321.5 (3956.1 to 4749.1) | 0.3 (0.1 to 0.5) | 18.9 (7.0 to 42.3) | 19.8 (7.3 to 43.8) | 0.3 (0.1 to 0.5) |
| Cyprus | 3183.1 (2894.5 to 3536.1) | 2515.1 (2268.3 to 2821.5) | -0.8 (-1.0 to -0.7) | 14.6 (5.3 to 32.1) | 11.5 (4.1 to 25.5) | -0.8 (-1.0 to -0.7) |
| Czechia | 13718.0 (12532.5 to 15087.8) | 9989.1 (9047.6 to 11050.7) | -0.6 (-0.9 to -0.3) | 62.7 (23.3 to 139.7) | 45.7 (16.9 to 101.7) | -0.6 (-0.8 to -0.3) |
| Democratic People's Republic of Korea | 1318.5 (1227.9 to 1432.3) | 1138.1 (1065.1 to 1222.9) | -0.4 (-0.5 to -0.3) | 6.1 (2.2 to 13.7) | 5.2 (1.9 to 11.6) | -0.4 (-0.5 to -0.3) |
| Democratic Republic of the Congo | 1894.7 (1773.2 to 2025.0) | 2018.6 (1677.0 to 2609.5) | 0.2 (0.0 to 0.5) | 8.5 (3.1 to 18.4) | 9.2 (3.4 to 20.1) | 0.3 (0.1 to 0.5) |
| Denmark | 2673.9 (2420.9 to 2973.1) | 2304.9 (2063.0 to 2601.6) | -0.5 (-0.5 to -0.4) | 12.2 (4.4 to 27.0) | 10.6 (3.8 to 23.6) | -0.5 (-0.5 to -0.4) |
| Djibouti | 2224.0 (2059.1 to 2395.1) | 2148.4 (1951.5 to 2397.1) | -0.3 (-0.5 to -0.2) | 10.2 (3.7 to 22.2) | 9.8 (3.5 to 21.4) | -0.3 (-0.5 to -0.2) |
| Dominica | 3181.4 (2933.6 to 3483.0) | 3517.7 (3236.3 to 3854.4) | 0.3 (0.2 to 0.5) | 14.6 (5.3 to 32.4) | 16.1 (5.9 to 35.4) | 0.3 (0.2 to 0.5) |
| Dominican Republic | 3436.7 (3166.9 to 3747.4) | 4045.8 (3724.2 to 4405.3) | 0.6 (0.4 to 0.9) | 15.7 (5.8 to 34.8) | 18.5 (6.8 to 40.8) | 0.6 (0.4 to 0.9) |
| Ecuador | 4169.8 (3870.0 to 4519.5) | 3631.2 (3360.1 to 3940.3) | -0.4 (-0.5 to -0.3) | 19.1 (7.1 to 42.0) | 16.6 (6.2 to 36.2) | -0.4 (-0.5 to -0.3) |
| Egypt | 3440.1 (3180.4 to 3743.2) | 2609.6 (2379.9 to 2946.7) | -0.8 (-0.9 to -0.8) | 15.7 (5.8 to 35.4) | 11.9 (4.4 to 26.5) | -0.8 (-0.9 to -0.8) |
| El Salvador | 7605.4 (5580.5 to 11431.3) | 5428.0 (4346.5 to 7238.5) | -1.2 (-1.3 to -1.1) | 34.9 (12.2 to 77.8) | 24.7 (8.9 to 54.4) | -1.2 (-1.3 to -1.1) |
| Equatorial Guinea | 2076.3 (1944.1 to 2203.9) | 1433.2 (1331.7 to 1547.9) | -1.2 (-1.3 to -1.2) | 9.4 (3.4 to 20.3) | 6.5 (2.4 to 14.3) | -1.2 (-1.2 to -1.2) |
| Eritrea | 7954.3 (4424.8 to 14553.4) | 5656.0 (3328.0 to 10266.1) | -1.3 (-1.4 to -1.1) | 36.6 (11.6 to 89.2) | 25.7 (8.2 to 61.2) | -1.3 (-1.5 to -1.2) |
| Estonia | 9593.9 (8798.8 to 10459.6) | 6384.7 (5850.1 to 6994.5) | -1.6 (-1.7 to -1.5) | 43.8 (16.5 to 96.1) | 29.2 (10.9 to 64.8) | -1.6 (-1.7 to -1.5) |
| Eswatini | 2273.1 (2115.7 to 2429.6) | 2124.7 (1983.9 to 2263.5) | -0.2 (-0.3 to -0.1) | 10.4 (3.8 to 22.3) | 9.6 (3.6 to 20.8) | -0.2 (-0.4 to -0.1) |
| Ethiopia | 3186.0 (2866.3 to 3682.9) | 2440.8 (2013.5 to 3388.6) | -1.1 (-1.2 to -0.9) | 14.4 (5.3 to 31.5) | 11.1 (4.1 to 24.9) | -1.0 (-1.2 to -0.9) |
| Fiji | 1702.1 (1589.0 to 1838.4) | 1434.8 (1324.4 to 1573.5) | -0.6 (-0.7 to -0.6) | 7.8 (2.9 to 17.1) | 6.5 (2.4 to 14.3) | -0.6 (-0.7 to -0.6) |
| Finland | 3423.0 (3076.8 to 3816.2) | 2899.3 (2572.1 to 3305.6) | -0.7 (-1.2 to -0.1) | 15.7 (5.7 to 34.8) | 13.3 (4.8 to 29.8) | -0.7 (-1.2 to -0.1) |
| France | 3087.4 (2813.0 to 3415.0) | 2517.9 (2268.0 to 2816.8) | -0.5 (-0.6 to -0.5) | 14.1 (5.2 to 31.0) | 11.5 (4.1 to 25.8) | -0.5 (-0.6 to -0.5) |
| Gabon | 2063.8 (1924.8 to 2204.3) | 1635.2 (1530.2 to 1750.9) | -0.7 (-0.8 to -0.7) | 9.4 (3.5 to 20.5) | 7.4 (2.8 to 16.3) | -0.7 (-0.8 to -0.7) |
| Gambia | 2166.8 (1941.2 to 2423.0) | 1789.7 (1623.9 to 1950.0) | -0.7 (-0.8 to -0.6) | 9.9 (3.6 to 22.0) | 8.2 (3.0 to 18.0) | -0.7 (-0.8 to -0.6) |
| Georgia | 7253.0 (6640.6 to 7903.4) | 7781.4 (7141.0 to 8499.1) | 0.4 (0.3 to 0.5) | 33.3 (12.3 to 73.9) | 35.6 (13.0 to 77.8) | 0.4 (0.3 to 0.5) |
| Germany | 2543.2 (2298.2 to 2832.0) | 2298.2 (2059.1 to 2589.8) | -0.4 (-0.4 to -0.3) | 11.7 (4.2 to 25.3) | 10.5 (3.8 to 23.0) | -0.4 (-0.4 to -0.3) |
| Ghana | 1721.3 (1589.5 to 1847.9) | 1587.6 (1468.8 to 1709.9) | -0.3 (-0.4 to -0.1) | 7.8 (2.9 to 17.1) | 7.3 (2.7 to 15.9) | -0.2 (-0.3 to -0.1) |
| Greece | 2771.8 (2529.2 to 3085.0) | 2393.6 (2162.1 to 2675.0) | -0.5 (-0.6 to -0.4) | 12.7 (4.6 to 28.1) | 11.0 (3.9 to 24.5) | -0.5 (-0.6 to -0.4) |
| Greenland | 3039.9 (2766.4 to 3381.9) | 2164.6 (1960.8 to 2406.8) | -1.3 (-1.4 to -1.2) | 13.9 (5.1 to 30.0) | 9.9 (3.6 to 21.5) | -1.3 (-1.4 to -1.2) |
| Grenada | 5049.1 (4496.9 to 5792.2) | 5169.5 (4693.1 to 5726.5) | 0.1 (0.0 to 0.3) | 23.1 (8.3 to 50.1) | 23.6 (8.4 to 51.1) | 0.1 (0.0 to 0.3) |
| Guam | 1957.3 (1813.9 to 2131.8) | 1536.2 (1412.4 to 1683.1) | -0.8 (-0.9 to -0.8) | 9.0 (3.3 to 20.0) | 7.0 (2.6 to 15.8) | -0.8 (-0.9 to -0.8) |
| Guatemala | 7030.8 (5911.9 to 9028.1) | 7074.6 (6244.5 to 8320.1) | 0.1 (0.0 to 0.1) | 32.1 (11.7 to 71.4) | 32.1 (11.5 to 69.1) | 0.1 (0.0 to 0.1) |
| Guinea | 2183.4 (2017.2 to 2338.0) | 2078.5 (1915.1 to 2233.4) | -0.1 (-0.1 to 0.0) | 10.0 (3.6 to 21.7) | 9.5 (3.5 to 21.0) | -0.1 (-0.1 to 0.0) |
| Guinea-Bissau | 2402.8 (2239.0 to 2550.3) | 2001.2 (1835.4 to 2195.5) | -0.6 (-0.7 to -0.5) | 10.9 (4.0 to 24.0) | 9.1 (3.3 to 20.1) | -0.6 (-0.7 to -0.5) |
| Guyana | 3718.3 (3451.2 to 4010.7) | 4128.7 (3820.0 to 4462.2) | 0.2 (0.1 to 0.4) | 16.9 (6.2 to 37.3) | 18.7 (6.9 to 41.3) | 0.2 (0.1 to 0.4) |
| Haiti | 5197.5 (4801.5 to 5646.2) | 5696.1 (5002.4 to 6782.4) | 0.6 (0.4 to 0.8) | 23.6 (8.7 to 51.4) | 25.9 (9.6 to 55.5) | 0.6 (0.4 to 0.8) |
| Honduras | 5575.3 (5062.1 to 6128.0) | 4520.1 (4120.4 to 4965.6) | -0.7 (-0.8 to -0.6) | 25.5 (9.5 to 56.0) | 20.6 (7.6 to 44.4) | -0.7 (-0.9 to -0.6) |
| Hungary | 10727.2 (9783.5 to 11827.5) | 8658.3 (7799.0 to 9560.5) | -0.6 (-0.7 to -0.6) | 48.9 (17.8 to 108.6) | 39.7 (14.7 to 88.1) | -0.6 (-0.7 to -0.5) |
| Iceland | 3137.8 (2842.3 to 3486.3) | 2488.6 (2226.5 to 2806.6) | -0.7 (-0.8 to -0.6) | 14.4 (5.2 to 31.5) | 11.4 (4.2 to 25.3) | -0.7 (-0.8 to -0.6) |
| India | 2624.7 (2401.1 to 2877.7) | 1974.5 (1802.8 to 2175.4) | -1.1 (-1.2 to -1.0) | 11.8 (4.4 to 25.9) | 8.9 (3.3 to 19.6) | -1.1 (-1.2 to -1.0) |
| Indonesia | 2791.6 (2544.4 to 3156.2) | 2000.9 (1824.0 to 2237.1) | -1.0 (-1.1 to -0.9) | 12.8 (4.6 to 28.5) | 9.2 (3.3 to 20.1) | -1.0 (-1.1 to -0.9) |
| Iran (Islamic Republic of) | 5261.2 (4628.2 to 6199.3) | 3672.5 (3285.1 to 4139.1) | -1.2 (-1.3 to -1.1) | 24.1 (8.6 to 52.8) | 16.7 (6.0 to 36.9) | -1.2 (-1.3 to -1.1) |
| Iraq | 10512.3 (8549.2 to 13405.6) | 9382.6 (6919.7 to 13474.1) | -0.2 (-0.3 to -0.2) | 48.0 (17.4 to 105.7) | 42.6 (14.8 to 94.4) | -0.2 (-0.3 to -0.2) |
| Ireland | 3119.3 (2809.9 to 3492.2) | 2469.4 (2209.4 to 2766.8) | -0.8 (-0.8 to -0.7) | 14.3 (5.3 to 31.5) | 11.3 (4.1 to 25.2) | -0.8 (-0.8 to -0.7) |
| Israel | 3047.6 (2724.4 to 3429.2) | 2416.0 (2122.7 to 2739.9) | -0.6 (-0.7 to -0.5) | 14.0 (5.1 to 30.5) | 11.1 (4.0 to 24.6) | -0.6 (-0.7 to -0.5) |
| Italy | 3204.9 (2885.0 to 3554.9) | 2195.6 (1967.4 to 2497.9) | -1.6 (-1.7 to -1.4) | 14.7 (5.4 to 32.2) | 10.1 (3.6 to 22.7) | -1.5 (-1.7 to -1.4) |
| Jamaica | 5199.4 (4755.3 to 5683.8) | 4693.6 (4286.4 to 5148.6) | -0.8 (-1.0 to -0.6) | 23.9 (8.9 to 52.6) | 21.5 (7.9 to 47.3) | -0.8 (-1.0 to -0.6) |
| Japan | 2967.2 (2694.6 to 3279.4) | 2213.2 (1998.3 to 2464.2) | -1.0 (-1.1 to -0.8) | 13.6 (5.0 to 29.6) | 10.2 (3.7 to 22.2) | -1.0 (-1.1 to -0.8) |
| Jordan | 3213.3 (2984.1 to 3488.5) | 2408.8 (2211.0 to 2626.9) | -0.9 (-1.0 to -0.8) | 14.7 (5.4 to 32.8) | 11.0 (4.1 to 24.0) | -0.9 (-1.0 to -0.8) |
| Kazakhstan | 7547.7 (6924.2 to 8192.4) | 6060.0 (5555.7 to 6661.0) | -0.8 (-0.9 to -0.7) | 34.6 (12.8 to 75.6) | 27.7 (10.4 to 60.4) | -0.8 (-0.9 to -0.7) |
| Kenya | 2091.2 (1945.3 to 2228.3) | 1846.9 (1710.9 to 1977.4) | -0.6 (-0.8 to -0.4) | 9.5 (3.5 to 20.7) | 8.4 (3.1 to 18.4) | -0.6 (-0.8 to -0.4) |
| Kiribati | 1456.1 (1359.4 to 1572.7) | 1182.7 (1093.0 to 1294.5) | -0.6 (-0.7 to -0.5) | 6.6 (2.5 to 14.5) | 5.4 (1.9 to 11.8) | -0.6 (-0.7 to -0.5) |
| Kuwait | 3733.0 (3422.8 to 4069.3) | 3302.5 (2904.3 to 3763.6) | -0.7 (-0.9 to -0.5) | 17.1 (6.3 to 37.8) | 15.0 (5.6 to 32.9) | -0.7 (-0.9 to -0.5) |
| Kyrgyzstan | 5757.6 (5293.7 to 6347.5) | 4241.8 (3900.5 to 4658.4) | -1.1 (-1.1 to -1.0) | 26.4 (9.7 to 58.0) | 19.5 (7.2 to 42.8) | -1.0 (-1.1 to -1.0) |
| Lao People's Democratic Republic | 2789.8 (2605.9 to 2982.7) | 1990.9 (1857.8 to 2143.1) | -1.1 (-1.2 to -1.1) | 12.8 (4.7 to 27.8) | 9.1 (3.4 to 20.5) | -1.1 (-1.2 to -1.1) |
| Latvia | 10011.2 (9208.5 to 10905.9) | 6629.2 (6098.8 to 7286.3) | -1.6 (-1.7 to -1.5) | 45.6 (17.2 to 99.1) | 30.3 (11.4 to 67.1) | -1.6 (-1.7 to -1.5) |
| Lebanon | 6935.4 (4665.3 to 11676.3) | 4281.8 (3118.1 to 6592.6) | -1.6 (-1.7 to -1.5) | 31.8 (10.8 to 74.5) | 19.4 (6.8 to 44.0) | -1.6 (-1.7 to -1.5) |
| Lesotho | 1910.1 (1783.2 to 2037.6) | 2244.8 (2105.3 to 2381.3) | 0.6 (0.5 to 0.7) | 8.7 (3.2 to 18.9) | 10.1 (3.7 to 21.8) | 0.6 (0.5 to 0.7) |
| Liberia | 2254.2 (2033.1 to 2494.9) | 2968.3 (2089.1 to 4438.8) | 0.5 (0.1 to 0.9) | 10.2 (3.6 to 22.4) | 13.5 (4.7 to 30.5) | 0.5 (0.1 to 1.0) |
| Libya | 3972.8 (3637.9 to 4358.3) | 4501.5 (3685.3 to 5856.1) | 0.7 (0.5 to 0.9) | 18.2 (6.6 to 40.5) | 20.6 (7.6 to 46.0) | 0.7 (0.5 to 0.9) |
| Lithuania | 8840.0 (8100.8 to 9660.3) | 6343.1 (5792.5 to 6932.9) | -1.2 (-1.4 to -1.0) | 40.3 (15.1 to 87.4) | 28.9 (10.8 to 63.7) | -1.2 (-1.3 to -1.0) |
| Luxembourg | 3313.8 (2990.6 to 3649.6) | 2574.1 (2320.1 to 2866.7) | -0.9 (-1.0 to -0.8) | 15.2 (5.6 to 33.6) | 11.8 (4.3 to 25.7) | -0.9 (-1.0 to -0.8) |
| Madagascar | 2200.1 (2047.3 to 2353.6) | 1675.9 (1556.1 to 1802.2) | -0.8 (-0.9 to -0.8) | 10.0 (3.7 to 22.1) | 7.7 (2.8 to 16.7) | -0.8 (-0.9 to -0.7) |
| Malawi | 1903.7 (1761.1 to 2054.4) | 1528.7 (1408.5 to 1642.4) | -0.7 (-0.8 to -0.7) | 8.7 (3.1 to 18.5) | 7.0 (2.6 to 15.3) | -0.7 (-0.8 to -0.6) |
| Malaysia | 2309.4 (2140.3 to 2505.1) | 1924.5 (1778.2 to 2087.0) | -0.6 (-0.7 to -0.6) | 10.5 (3.9 to 23.3) | 8.8 (3.3 to 19.6) | -0.6 (-0.7 to -0.6) |
| Maldives | 3126.8 (2886.9 to 3394.4) | 2296.7 (2116.4 to 2520.6) | -0.9 (-1.0 to -0.8) | 14.3 (5.3 to 31.8) | 10.5 (3.9 to 23.3) | -0.9 (-1.0 to -0.8) |
| Mali | 2175.9 (2011.7 to 2330.4) | 2694.0 (2247.7 to 3432.9) | 0.9 (0.7 to 1.1) | 9.9 (3.6 to 21.6) | 12.3 (4.6 to 26.8) | 0.9 (0.7 to 1.1) |
| Malta | 2898.7 (2625.6 to 3222.2) | 2668.7 (2397.5 to 2987.6) | -0.3 (-0.3 to -0.2) | 13.3 (4.9 to 29.4) | 12.3 (4.4 to 27.3) | -0.3 (-0.3 to -0.2) |
| Marshall Islands | 2006.7 (1872.1 to 2151.9) | 1667.0 (1547.0 to 1807.5) | -0.6 (-0.7 to -0.6) | 9.1 (3.4 to 20.2) | 7.6 (2.8 to 16.6) | -0.6 (-0.7 to -0.6) |
| Mauritania | 2027.6 (1899.4 to 2165.7) | 1630.8 (1519.1 to 1755.0) | -0.7 (-0.8 to -0.7) | 9.3 (3.4 to 20.1) | 7.5 (2.7 to 16.4) | -0.7 (-0.8 to -0.7) |
| Mauritius | 2083.1 (1923.8 to 2255.2) | 1701.8 (1554.0 to 1854.5) | -0.5 (-0.6 to -0.5) | 9.5 (3.5 to 21.1) | 7.8 (2.8 to 17.0) | -0.5 (-0.6 to -0.5) |
| Mexico | 7131.2 (6536.6 to 7847.2) | 4395.7 (4010.1 to 4840.6) | 0.0 (-0.6 to 0.6) | 32.4 (12.1 to 71.3) | 20.0 (7.4 to 43.7) | 0.0 (-0.6 to 0.6) |
| Micronesia (Federated States of) | 1995.4 (1863.6 to 2148.4) | 1771.4 (1629.2 to 1937.3) | -0.4 (-0.4 to -0.3) | 9.1 (3.4 to 19.8) | 8.1 (3.0 to 17.4) | -0.4 (-0.4 to -0.3) |
| Monaco | 2499.9 (2229.6 to 2820.1) | 2397.9 (2152.6 to 2711.6) | -0.1 (-0.2 to 0.0) | 11.5 (4.1 to 25.4) | 11.0 (3.9 to 24.3) | -0.1 (-0.2 to -0.1) |
| Mongolia | 6836.7 (6265.5 to 7482.1) | 6658.2 (6110.8 to 7335.4) | 0.0 (0.0 to 0.1) | 31.3 (11.5 to 68.7) | 30.5 (11.2 to 67.3) | 0.0 (0.0 to 0.1) |
| Montenegro | 10495.5 (9583.5 to 11589.1) | 9112.5 (8233.9 to 10083.3) | -0.5 (-0.5 to -0.4) | 48.1 (17.9 to 107.2) | 41.7 (15.4 to 93.4) | -0.5 (-0.5 to -0.4) |
| Morocco | 4601.5 (4287.0 to 4967.0) | 3337.9 (3098.8 to 3604.2) | -1.1 (-1.2 to -1.0) | 21.0 (7.8 to 47.2) | 15.2 (5.6 to 33.7) | -1.1 (-1.2 to -1.0) |
| Mozambique | 3851.5 (2510.6 to 6792.2) | 2840.2 (2086.8 to 4343.9) | -1.0 (-1.1 to -1.0) | 17.6 (6.0 to 41.2) | 12.9 (4.6 to 28.6) | -1.1 (-1.1 to -1.0) |
| Myanmar | 3740.7 (3258.8 to 4916.1) | 3186.1 (2683.0 to 4116.0) | -0.5 (-0.6 to -0.4) | 17.1 (5.9 to 39.1) | 14.6 (5.1 to 32.1) | -0.5 (-0.6 to -0.4) |
| Namibia | 3140.2 (2492.9 to 4318.5) | 2495.9 (2120.0 to 3115.9) | -0.8 (-0.9 to -0.7) | 14.4 (5.2 to 32.0) | 11.3 (4.3 to 24.5) | -0.8 (-0.9 to -0.7) |
| Nauru | 2161.7 (2022.3 to 2321.3) | 1837.8 (1696.6 to 1995.9) | -0.6 (-0.7 to -0.5) | 9.8 (3.7 to 21.6) | 8.4 (3.1 to 18.3) | -0.6 (-0.7 to -0.5) |
| Nepal | 2316.8 (2098.8 to 2558.0) | 2497.7 (2147.6 to 2987.0) | 0.4 (0.2 to 0.5) | 10.5 (3.9 to 23.1) | 11.4 (4.1 to 24.4) | 0.4 (0.3 to 0.6) |
| Netherlands | 2218.5 (2001.1 to 2468.6) | 2018.1 (1818.3 to 2262.6) | -0.2 (-0.5 to 0.1) | 10.2 (3.7 to 22.6) | 9.3 (3.3 to 20.3) | -0.2 (-0.5 to 0.1) |
| New Zealand | 8010.7 (7116.9 to 9021.5) | 6723.4 (5985.5 to 7579.9) | -0.6 (-0.7 to -0.5) | 36.5 (13.2 to 80.7) | 30.7 (11.2 to 67.2) | -0.6 (-0.7 to -0.5) |
| Nicaragua | 7451.8 (5561.6 to 11264.8) | 5196.3 (4128.3 to 7006.8) | -1.1 (-1.2 to -1.1) | 34.3 (12.1 to 77.0) | 23.7 (8.5 to 51.8) | -1.2 (-1.2 to -1.1) |
| Niger | 2153.1 (1988.3 to 2298.5) | 1938.9 (1780.6 to 2109.7) | -0.4 (-0.4 to -0.3) | 9.8 (3.6 to 21.4) | 8.9 (3.2 to 19.3) | -0.4 (-0.4 to -0.3) |
| Nigeria | 2129.0 (1966.6 to 2270.6) | 1705.3 (1554.3 to 1859.5) | -0.8 (-0.9 to -0.7) | 9.7 (3.5 to 21.3) | 7.8 (2.8 to 17.2) | -0.8 (-0.9 to -0.7) |
| Niue | 1845.7 (1713.1 to 1997.6) | 1621.5 (1485.6 to 1793.3) | -0.5 (-0.6 to -0.4) | 8.4 (3.2 to 18.8) | 7.4 (2.7 to 16.5) | -0.5 (-0.6 to -0.4) |
| North Macedonia | 10893.0 (9982.9 to 12033.0) | 9403.7 (8526.6 to 10457.3) | -0.5 (-0.6 to -0.4) | 49.9 (18.5 to 111.7) | 43.0 (15.9 to 95.8) | -0.5 (-0.6 to -0.4) |
| Northern Mariana Islands | 2902.8 (2681.9 to 3130.9) | 2102.4 (1938.1 to 2292.9) | -1.1 (-1.2 to -1.1) | 13.3 (5.0 to 29.3) | 9.6 (3.6 to 21.4) | -1.1 (-1.2 to -1.1) |
| Norway | 3387.3 (3059.5 to 3783.5) | 2339.1 (2109.0 to 2613.7) | -1.1 (-1.2 to -1.1) | 15.5 (5.7 to 33.6) | 10.7 (3.9 to 23.6) | -1.1 (-1.2 to -1.0) |
| Oman | 4840.7 (4534.3 to 5184.6) | 3372.1 (3105.0 to 3656.8) | -1.2 (-1.3 to -1.1) | 22.1 (8.1 to 48.3) | 15.4 (5.7 to 33.6) | -1.2 (-1.3 to -1.1) |
| Pakistan | 1713.7 (1604.2 to 1831.6) | 1764.7 (1560.7 to 2071.6) | 0.2 (0.1 to 0.2) | 7.8 (2.9 to 17.0) | 8.0 (3.0 to 17.5) | 0.2 (0.1 to 0.3) |
| Palau | 2815.1 (2598.6 to 3055.6) | 2680.9 (2452.3 to 2965.4) | -0.2 (-0.2 to -0.1) | 12.8 (4.8 to 27.9) | 12.2 (4.6 to 26.5) | -0.2 (-0.2 to -0.1) |
| Palestine | 5716.0 (4311.9 to 8207.1) | 6473.3 (4236.7 to 10391.8) | 1.0 (0.7 to 1.3) | 26.4 (9.0 to 59.6) | 29.6 (10.0 to 68.5) | 1.0 (0.7 to 1.3) |
| Panama | 4801.7 (4299.4 to 5452.8) | 4222.3 (3784.4 to 4730.8) | -0.4 (-0.5 to -0.3) | 22.0 (7.9 to 49.0) | 19.3 (7.0 to 43.2) | -0.4 (-0.5 to -0.4) |
| Papua New Guinea | 1622.2 (1505.9 to 1745.8) | 1820.7 (1642.6 to 2030.6) | 0.3 (0.2 to 0.4) | 7.4 (2.7 to 16.4) | 8.3 (3.0 to 18.2) | 0.3 (0.2 to 0.5) |
| Paraguay | 4177.7 (3793.9 to 4635.4) | 3640.4 (3327.0 to 3988.3) | -0.5 (-0.6 to -0.4) | 19.1 (7.0 to 42.5) | 16.6 (6.1 to 36.4) | -0.5 (-0.6 to -0.4) |
| Peru | 4645.4 (4165.7 to 5322.8) | 4039.7 (3629.5 to 4577.2) | -0.5 (-0.6 to -0.4) | 21.3 (7.6 to 46.3) | 18.5 (6.6 to 40.4) | -0.5 (-0.6 to -0.5) |
| Philippines | 2942.3 (2580.9 to 3665.4) | 2173.4 (1865.6 to 2696.0) | -0.9 (-0.9 to -0.8) | 13.4 (4.7 to 29.8) | 9.9 (3.6 to 21.6) | -0.8 (-0.9 to -0.8) |
| Poland | 10899.0 (9983.3 to 11973.0) | 8113.9 (7397.4 to 8965.9) | -0.9 (-1.0 to -0.8) | 49.6 (18.5 to 110.2) | 37.0 (13.8 to 81.8) | -0.9 (-1.0 to -0.8) |
| Portugal | 3206.8 (2920.1 to 3544.0) | 1822.4 (1659.0 to 2025.7) | -2.0 (-2.1 to -1.9) | 14.7 (5.2 to 32.2) | 8.3 (3.0 to 18.1) | -2.0 (-2.1 to -1.9) |
| Puerto Rico | 3894.0 (3557.4 to 4276.9) | 4000.6 (3645.9 to 4422.2) | 0.1 (-0.1 to 0.2) | 17.8 (6.5 to 39.5) | 18.3 (6.7 to 40.1) | 0.1 (-0.1 to 0.2) |
| Qatar | 5200.5 (4801.5 to 5659.1) | 3971.6 (3628.5 to 4356.3) | -0.7 (-0.8 to -0.6) | 23.8 (8.8 to 52.4) | 18.1 (6.8 to 39.7) | -0.7 (-0.8 to -0.6) |
| Republic of Korea | 3795.9 (3443.8 to 4223.5) | 2432.4 (2196.6 to 2712.9) | -1.9 (-2.1 to -1.7) | 17.4 (6.3 to 38.5) | 11.2 (4.1 to 24.6) | -1.9 (-2.1 to -1.7) |
| Republic of Moldova | 8477.6 (7805.6 to 9238.8) | 5468.3 (5036.3 to 5969.9) | -1.7 (-1.7 to -1.6) | 38.8 (14.5 to 84.6) | 25.0 (9.3 to 55.3) | -1.7 (-1.7 to -1.6) |
| Romania | 12600.4 (11528.8 to 13820.6) | 9596.0 (8724.2 to 10560.0) | -1.0 (-1.0 to -0.9) | 57.5 (21.1 to 126.2) | 44.0 (16.4 to 97.8) | -1.0 (-1.0 to -0.9) |
| Russian Federation | 10582.4 (9754.1 to 11530.9) | 6818.2 (6296.9 to 7457.7) | -1.8 (-2.0 to -1.6) | 48.2 (18.1 to 106.0) | 31.0 (11.7 to 67.0) | -1.8 (-2.0 to -1.6) |
| Rwanda | 2145.3 (1988.3 to 2292.0) | 3391.5 (2307.8 to 5154.2) | 0.5 (-0.3 to 1.4) | 9.8 (3.5 to 21.4) | 15.5 (5.3 to 35.5) | 0.6 (-0.3 to 1.4) |
| Saint Kitts and Nevis | 5217.9 (4762.1 to 5724.2) | 5703.0 (5199.7 to 6227.5) | 0.5 (0.3 to 0.7) | 23.8 (8.8 to 52.4) | 26.0 (9.5 to 57.5) | 0.5 (0.3 to 0.7) |
| Saint Lucia | 3842.1 (3512.6 to 4202.0) | 4197.5 (3838.8 to 4596.5) | 0.3 (0.2 to 0.4) | 17.6 (6.5 to 38.7) | 19.2 (7.0 to 42.2) | 0.3 (0.1 to 0.4) |
| Saint Vincent and the Grenadines | 4046.2 (3730.1 to 4418.0) | 4386.6 (4058.6 to 4783.7) | 0.2 (0.1 to 0.4) | 18.5 (6.8 to 40.6) | 20.0 (7.3 to 44.6) | 0.2 (0.0 to 0.4) |
| Samoa | 1839.9 (1719.4 to 1977.1) | 1782.0 (1594.6 to 2044.5) | 0.0 (-0.1 to 0.1) | 8.4 (3.1 to 18.6) | 8.1 (3.0 to 17.6) | 0.0 (-0.1 to 0.1) |
| San Marino | 2920.4 (2624.5 to 3278.3) | 2641.2 (2372.4 to 2950.7) | -0.3 (-0.3 to -0.3) | 13.4 (4.9 to 29.2) | 12.1 (4.3 to 26.8) | -0.3 (-0.3 to -0.3) |
| Sao Tome and Principe | 2310.8 (2112.0 to 2528.9) | 2099.2 (1927.3 to 2314.3) | -0.3 (-0.4 to -0.2) | 10.6 (3.8 to 23.6) | 9.6 (3.5 to 21.4) | -0.3 (-0.4 to -0.2) |
| Saudi Arabia | 7488.7 (6903.9 to 8205.0) | 6563.1 (5969.7 to 7267.7) | -0.1 (-0.5 to 0.3) | 34.1 (12.8 to 74.8) | 29.7 (11.2 to 63.7) | -0.1 (-0.5 to 0.3) |
| Senegal | 1918.4 (1764.1 to 2060.8) | 1706.3 (1568.1 to 1835.9) | -0.4 (-0.5 to -0.3) | 8.7 (3.2 to 19.1) | 7.8 (2.8 to 17.3) | -0.4 (-0.4 to -0.3) |
| Serbia | 10636.2 (9713.0 to 11727.0) | 9219.9 (8328.5 to 10295.8) | -0.4 (-0.5 to -0.3) | 48.7 (18.0 to 108.9) | 42.2 (15.4 to 93.5) | -0.4 (-0.5 to -0.3) |
| Seychelles | 2869.5 (2635.9 to 3127.6) | 2035.8 (1884.4 to 2212.8) | -1.1 (-1.2 to -1.0) | 13.2 (4.8 to 28.5) | 9.3 (3.4 to 20.7) | -1.1 (-1.2 to -1.0) |
| Sierra Leone | 2005.3 (1854.5 to 2149.7) | 2689.1 (2077.9 to 3732.6) | 0.2 (-0.3 to 0.8) | 9.1 (3.3 to 20.2) | 12.3 (4.3 to 27.2) | 0.3 (-0.3 to 0.8) |
| Singapore | 3125.1 (2832.8 to 3479.9) | 2758.0 (2491.4 to 3083.1) | -0.5 (-0.6 to -0.4) | 14.4 (5.2 to 31.6) | 12.7 (4.7 to 27.9) | -0.5 (-0.6 to -0.4) |
| Slovakia | 12244.0 (11219.7 to 13466.8) | 10184.9 (9271.1 to 11240.0) | -0.6 (-0.6 to -0.6) | 55.8 (20.9 to 122.7) | 46.6 (17.3 to 103.6) | -0.6 (-0.6 to -0.5) |
| Slovenia | 14564.3 (13347.0 to 15928.0) | 11420.9 (10396.3 to 12557.2) | -0.3 (-0.7 to 0.0) | 66.4 (24.7 to 147.4) | 52.2 (19.4 to 115.9) | -0.3 (-0.7 to 0.0) |
| Solomon Islands | 2219.8 (2050.1 to 2390.8) | 2122.0 (1923.4 to 2361.6) | -0.2 (-0.2 to -0.2) | 10.1 (3.7 to 22.0) | 9.7 (3.6 to 21.1) | -0.2 (-0.2 to -0.2) |
| Somalia | 2699.8 (2270.0 to 4274.8) | 3315.8 (2535.6 to 5111.0) | 0.4 (0.4 to 0.5) | 12.3 (4.1 to 27.7) | 15.2 (5.5 to 34.3) | 0.4 (0.4 to 0.5) |
| South Africa | 3221.9 (2972.9 to 3539.4) | 2114.0 (1948.5 to 2321.3) | -1.5 (-1.7 to -1.4) | 14.7 (5.4 to 32.6) | 9.6 (3.5 to 21.2) | -1.5 (-1.7 to -1.4) |
| South Sudan | 2868.5 (2448.7 to 3561.5) | 3510.2 (2592.2 to 5151.9) | 0.9 (0.8 to 1.1) | 13.1 (4.9 to 28.4) | 16.0 (5.6 to 36.1) | 1.0 (0.8 to 1.1) |
| Spain | 2909.1 (2641.6 to 3207.5) | 2231.5 (2002.1 to 2507.1) | -0.9 (-0.9 to -0.8) | 13.4 (4.8 to 29.2) | 10.2 (3.7 to 22.4) | -0.9 (-0.9 to -0.8) |
| Sri Lanka | 3987.6 (3450.6 to 4887.1) | 4661.9 (3356.1 to 6875.0) | 0.3 (0.1 to 0.6) | 18.3 (6.6 to 40.1) | 21.4 (7.5 to 48.1) | 0.3 (0.1 to 0.6) |
| Sudan | 3939.5 (3571.6 to 4403.3) | 3813.4 (3153.8 to 4880.2) | 0.0 (-0.2 to 0.1) | 18.0 (6.4 to 39.8) | 17.4 (6.5 to 38.6) | 0.0 (-0.2 to 0.1) |
| Suriname | 3763.9 (3280.5 to 5028.0) | 3734.8 (3342.9 to 4486.2) | 0.0 (-0.1 to 0.2) | 17.3 (6.0 to 39.7) | 17.0 (6.2 to 39.1) | 0.0 (-0.1 to 0.1) |
| Sweden | 3065.9 (2768.6 to 3425.7) | 2349.0 (2120.6 to 2636.5) | -0.8 (-0.9 to -0.7) | 14.1 (5.2 to 30.9) | 10.8 (3.9 to 23.8) | -0.8 (-0.9 to -0.7) |
| Switzerland | 4514.3 (4057.3 to 5005.7) | 2970.8 (2651.4 to 3338.7) | -1.6 (-1.8 to -1.4) | 20.7 (7.6 to 45.2) | 13.6 (4.9 to 30.3) | -1.6 (-1.8 to -1.4) |
| Syrian Arab Republic | 3293.9 (2907.4 to 4009.9) | 7099.7 (4159.2 to 12192.2) | 2.4 (1.7 to 3.2) | 15.1 (5.3 to 34.2) | 32.7 (10.4 to 78.4) | 2.5 (1.7 to 3.2) |
| Taiwan (Province of China) | 1825.1 (1671.2 to 2010.2) | 1084.2 (997.4 to 1194.0) | -2.0 (-2.2 to -1.9) | 8.4 (3.1 to 18.6) | 5.0 (1.8 to 11.1) | -2.0 (-2.2 to -1.9) |
| Tajikistan | 5303.3 (4862.7 to 5826.3) | 4873.3 (4252.4 to 5696.2) | -0.6 (-0.8 to -0.4) | 24.4 (9.0 to 53.5) | 22.4 (8.1 to 49.3) | -0.6 (-0.8 to -0.4) |
| Thailand | 3118.2 (2906.1 to 3346.5) | 2329.1 (2140.4 to 2530.9) | -1.1 (-1.2 to -1.1) | 14.2 (5.3 to 31.3) | 10.7 (3.9 to 23.5) | -1.1 (-1.2 to -1.1) |
| Timor-Leste | 5369.8 (3590.5 to 8342.9) | 4982.4 (3027.9 to 8254.9) | -0.4 (-0.7 to -0.1) | 24.7 (8.4 to 57.8) | 22.8 (7.5 to 54.7) | -0.4 (-0.7 to -0.1) |
| Togo | 1983.0 (1834.4 to 2123.0) | 1681.3 (1560.7 to 1800.6) | -0.6 (-0.7 to -0.5) | 9.0 (3.3 to 19.5) | 7.7 (2.8 to 16.8) | -0.6 (-0.6 to -0.5) |
| Tokelau | 1783.8 (1656.9 to 1921.2) | 1462.3 (1349.7 to 1599.8) | -0.7 (-0.7 to -0.6) | 8.1 (3.1 to 17.9) | 6.7 (2.5 to 14.6) | -0.7 (-0.8 to -0.6) |
| Tonga | 1569.1 (1447.5 to 1689.8) | 1241.9 (1143.2 to 1364.0) | -0.7 (-0.8 to -0.7) | 7.2 (2.7 to 15.8) | 5.7 (2.1 to 12.7) | -0.7 (-0.8 to -0.7) |
| Trinidad and Tobago | 3454.7 (3186.4 to 3796.8) | 3500.7 (3174.4 to 3890.1) | 0.2 (0.1 to 0.3) | 15.8 (5.8 to 34.8) | 16.0 (5.9 to 36.1) | 0.2 (0.1 to 0.3) |
| Tunisia | 5274.8 (4847.9 to 5767.4) | 3988.5 (3668.8 to 4377.5) | -0.9 (-0.9 to -0.9) | 24.2 (8.9 to 53.8) | 18.2 (6.8 to 40.2) | -0.9 (-1.0 to -0.9) |
| Turkey | 3679.8 (3406.6 to 3984.2) | 2840.7 (2591.2 to 3124.8) | -0.9 (-0.9 to -0.8) | 16.9 (6.2 to 37.5) | 13.0 (4.7 to 28.9) | -0.9 (-0.9 to -0.8) |
| Turkmenistan | 5426.4 (4956.9 to 5942.0) | 5392.4 (4929.9 to 5955.8) | 0.1 (0.0 to 0.1) | 24.9 (9.1 to 54.9) | 24.8 (9.1 to 55.0) | 0.1 (0.0 to 0.1) |
| Tuvalu | 1862.0 (1746.3 to 1988.6) | 1605.9 (1482.8 to 1746.4) | -1.2 (-1.5 to -0.9) | 8.5 (3.2 to 18.7) | 7.3 (2.8 to 16.2) | -1.2 (-1.5 to -0.9) |
| Uganda | 3552.0 (2424.3 to 5854.8) | 2636.3 (1924.2 to 3938.7) | -1.0 (-1.1 to -0.9) | 16.2 (5.6 to 37.9) | 12.0 (4.3 to 26.7) | -1.0 (-1.1 to -0.9) |
| Ukraine | 8180.0 (7490.7 to 8978.8) | 7205.8 (6619.0 to 7895.5) | -0.7 (-0.8 to -0.6) | 37.3 (14.1 to 80.3) | 32.8 (12.2 to 71.2) | -0.7 (-0.8 to -0.6) |
| United Arab Emirates | 4843.4 (4489.4 to 5245.7) | 4201.0 (3844.1 to 4591.6) | -0.4 (-0.5 to -0.4) | 22.1 (8.2 to 48.0) | 19.1 (7.2 to 41.5) | -0.5 (-0.5 to -0.4) |
| United Kingdom | 2548.8 (2304.1 to 2839.6) | 2020.8 (1822.8 to 2254.0) | -0.7 (-0.8 to -0.6) | 11.7 (4.3 to 25.8) | 9.2 (3.4 to 20.4) | -0.7 (-0.8 to -0.6) |
| United Republic of Tanzania | 2061.9 (1909.0 to 2204.5) | 1761.3 (1635.5 to 1889.7) | -0.5 (-0.6 to -0.4) | 9.4 (3.4 to 20.5) | 8.0 (3.0 to 17.6) | -0.5 (-0.5 to -0.4) |
| United States of America | 2713.7 (2460.6 to 3029.0) | 1615.7 (1481.6 to 1769.9) | -1.9 (-2.3 to -1.5) | 12.4 (4.5 to 26.9) | 7.3 (2.7 to 15.9) | -1.9 (-2.3 to -1.5) |
| United States Virgin Islands | 4086.5 (3743.9 to 4483.9) | 3699.7 (3377.3 to 4088.0) | -0.5 (-0.8 to -0.3) | 18.7 (6.9 to 40.8) | 16.9 (6.2 to 37.0) | -0.6 (-0.8 to -0.3) |
| Uruguay | 7513.9 (6694.4 to 8417.5) | 5444.2 (4885.9 to 6133.0) | -1.2 (-1.3 to -1.2) | 34.4 (12.5 to 74.8) | 24.9 (9.0 to 53.7) | -1.3 (-1.3 to -1.2) |
| Uzbekistan | 5400.1 (4957.1 to 5877.9) | 4500.2 (4160.9 to 4928.5) | -0.6 (-0.7 to -0.5) | 24.8 (9.1 to 54.3) | 20.6 (7.6 to 45.9) | -0.6 (-0.7 to -0.5) |
| Vanuatu | 1861.4 (1719.7 to 2024.3) | 1592.3 (1456.1 to 1756.8) | -0.5 (-0.6 to -0.5) | 8.5 (3.1 to 18.5) | 7.3 (2.7 to 15.7) | -0.5 (-0.6 to -0.5) |
| Venezuela (Bolivarian Republic of) | 6391.8 (5778.8 to 7103.5) | 6050.3 (5494.4 to 6661.0) | -0.1 (-0.2 to 0.1) | 29.3 (10.7 to 64.1) | 27.7 (10.0 to 61.3) | -0.1 (-0.2 to 0.1) |
| Viet Nam | 2960.3 (2732.7 to 3202.4) | 2454.8 (2279.2 to 2662.8) | -0.5 (-0.6 to -0.5) | 13.6 (5.0 to 29.6) | 11.3 (4.2 to 25.0) | -0.5 (-0.6 to -0.5) |
| Yemen | 3780.3 (3435.8 to 4286.1) | 4366.2 (3365.9 to 6161.8) | 0.2 (-0.1 to 0.4) | 17.2 (6.2 to 38.2) | 20.0 (7.1 to 44.5) | 0.2 (-0.1 to 0.4) |
| Zambia | 2030.5 (1885.5 to 2174.7) | 1745.3 (1618.9 to 1876.0) | -0.5 (-0.7 to -0.4) | 9.2 (3.4 to 20.2) | 7.9 (2.9 to 17.3) | -0.5 (-0.7 to -0.4) |
| Zimbabwe | 1870.5 (1744.7 to 2007.7) | 1724.4 (1609.8 to 1844.7) | -0.3 (-0.4 to -0.2) | 8.6 (3.1 to 18.6) | 7.8 (2.9 to 17.5) | -0.3 (-0.4 to -0.3) |

**Table S13. ASR of prevalence and YLDs in 1990 and 2021, and their EAPC from 1990 to 2021 for AMP-LL1, by country.**

| **Countries** | **Prevalence** | | | **YLDs** | | |
| --- | --- | --- | --- | --- | --- | --- |
|  | **Age-standardized prevalence rate, 1990** | **Age-standardized prevalence rate, 2021** | **EAPC of prevalence rate No. (95% CI)** | **Age-standardized YLDs rate, 1990** | **Age-standardized YLDs rate, 2021** | **EAPC of YLDs rate No. (95% CI)** |
| Afghanistan | 1920.7 (519.7 to 5481.0) | 1565.1 (674.3 to 3373.3) | -0.9 (-1.1 to -0.8) | 250.7 (64.2 to 753.8) | 168.1 (66.2 to 361.2) | -1.6 (-1.7 to -1.5) |
| Albania | 496.6 (464.4 to 531.1) | 446.4 (414.8 to 479.7) | -0.5 (-0.7 to -0.4) | 41.1 (28.8 to 54.5) | 19.4 (12.5 to 27.8) | -2.9 (-3.0 to -2.7) |
| Algeria | 347.2 (326.5 to 367.2) | 272.5 (249.3 to 301.2) | -0.9 (-1.0 to -0.8) | 32.7 (22.4 to 43.8) | 16.1 (10.9 to 22.1) | -2.4 (-2.5 to -2.3) |
| American Samoa | 182.9 (173.5 to 193.2) | 200.9 (180.7 to 232.5) | 0.6 (0.5 to 0.8) | 16.7 (11.8 to 22.3) | 16.1 (11.0 to 21.8) | 0.3 (0.1 to 0.5) |
| Andorra | 25.0 (23.2 to 27.1) | 24.6 (22.8 to 26.6) | -0.1 (-0.1 to 0.0) | 0.9 (0.6 to 1.4) | 0.9 (0.5 to 1.3) | -0.1 (-0.2 to -0.1) |
| Angola | 446.9 (296.0 to 708.9) | 358.2 (243.2 to 565.5) | -0.9 (-1.2 to -0.6) | 59.6 (32.1 to 98.5) | 37.9 (20.8 to 62.3) | -1.7 (-2.0 to -1.3) |
| Antigua and Barbuda | 214.3 (201.7 to 228.9) | 222.1 (207.3 to 240.8) | 0.1 (-0.1 to 0.2) | 17.5 (12.3 to 23.2) | 13.5 (9.4 to 17.9) | -0.9 (-1.0 to -0.8) |
| Argentina | 24.1 (22.8 to 25.7) | 21.9 (20.8 to 23.2) | -0.3 (-0.5 to -0.2) | 2.0 (1.4 to 2.8) | 1.3 (0.9 to 1.8) | -1.4 (-1.5 to -1.2) |
| Armenia | 494.8 (398.6 to 721.9) | 377.1 (297.8 to 497.1) | -1.7 (-2.0 to -1.4) | 38.9 (25.0 to 61.4) | 20.7 (12.6 to 31.3) | -3.0 (-3.3 to -2.7) |
| Australia | 32.0 (30.0 to 33.7) | 27.3 (25.5 to 29.1) | -0.4 (-0.5 to -0.4) | 1.2 (0.7 to 1.8) | 1.0 (0.6 to 1.5) | -0.5 (-0.5 to -0.4) |
| Austria | 27.8 (25.7 to 29.6) | 20.2 (18.6 to 21.8) | -1.0 (-1.1 to -1.0) | 1.1 (0.7 to 1.7) | 0.7 (0.5 to 1.1) | -1.2 (-1.2 to -1.1) |
| Azerbaijan | 258.5 (246.3 to 271.4) | 253.6 (223.4 to 299.2) | -0.6 (-0.8 to -0.4) | 24.3 (17.0 to 32.5) | 17.8 (12.0 to 25.1) | -1.7 (-1.9 to -1.5) |
| Bahamas | 206.2 (196.3 to 216.1) | 214.7 (201.4 to 237.7) | 0.0 (0.0 to 0.1) | 18.3 (12.7 to 24.3) | 15.2 (10.4 to 20.2) | -0.7 (-0.7 to -0.6) |
| Bahrain | 255.7 (234.9 to 284.7) | 198.6 (187.0 to 211.4) | -2.0 (-2.5 to -1.5) | 21.3 (15.0 to 28.6) | 8.6 (5.6 to 12.4) | -4.2 (-4.8 to -3.6) |
| Bangladesh | 114.8 (107.5 to 125.7) | 124.7 (110.3 to 150.8) | -0.2 (-0.3 to 0.0) | 13.9 (9.5 to 18.7) | 10.3 (6.8 to 14.1) | -1.5 (-1.7 to -1.3) |
| Barbados | 165.1 (157.3 to 173.6) | 167.5 (159.2 to 176.5) | -0.1 (-0.2 to 0.0) | 13.2 (9.1 to 17.3) | 9.9 (6.8 to 13.4) | -1.0 (-1.1 to -0.9) |
| Belarus | 515.4 (483.9 to 546.2) | 481.8 (447.3 to 517.7) | -0.4 (-0.7 to 0.0) | 34.1 (23.8 to 46.4) | 18.5 (11.6 to 27.2) | -2.3 (-2.9 to -1.8) |
| Belgium | 25.6 (23.7 to 27.3) | 23.0 (21.2 to 24.7) | -0.2 (-0.5 to 0.0) | 1.0 (0.6 to 1.5) | 0.8 (0.5 to 1.3) | -0.4 (-0.6 to -0.1) |
| Belize | 213.0 (202.2 to 226.4) | 263.9 (248.6 to 284.6) | 0.3 (0.1 to 0.6) | 20.2 (13.7 to 26.9) | 20.4 (14.0 to 27.1) | -0.4 (-0.7 to -0.2) |
| Benin | 151.5 (144.5 to 159.6) | 147.5 (139.5 to 155.8) | -0.2 (-0.2 to -0.1) | 18.3 (12.7 to 24.6) | 15.3 (10.7 to 20.6) | -0.7 (-0.7 to -0.6) |
| Bermuda | 190.1 (181.0 to 199.9) | 174.0 (164.2 to 184.1) | -0.8 (-1.0 to -0.6) | 12.6 (8.5 to 17.0) | 6.4 (3.9 to 9.5) | -2.8 (-3.0 to -2.6) |
| Bhutan | 156.6 (147.6 to 166.2) | 202.0 (184.5 to 220.8) | 0.8 (0.7 to 1.0) | 18.9 (13.2 to 25.6) | 16.9 (11.8 to 22.5) | -0.4 (-0.5 to -0.2) |
| Bolivia (Plurinational State of) | 249.4 (238.8 to 261.8) | 219.5 (208.4 to 231.0) | -0.5 (-0.5 to -0.4) | 30.7 (21.1 to 41.4) | 19.7 (13.7 to 26.1) | -1.5 (-1.5 to -1.5) |
| Bosnia and Herzegovina | 532.4 (475.8 to 604.0) | 590.8 (460.9 to 812.3) | -0.4 (-0.9 to 0.1) | 39.5 (27.3 to 53.5) | 25.7 (14.9 to 40.0) | -2.6 (-3.1 to -2.1) |
| Botswana | 167.7 (160.4 to 176.0) | 187.7 (179.1 to 197.0) | 0.2 (0.0 to 0.4) | 18.9 (13.0 to 25.2) | 17.6 (12.4 to 23.2) | -0.5 (-0.8 to -0.2) |
| Brazil | 394.6 (366.4 to 420.5) | 327.0 (301.5 to 362.7) | -0.7 (-0.7 to -0.7) | 37.8 (26.3 to 50.8) | 22.4 (15.5 to 30.2) | -1.7 (-1.8 to -1.7) |
| Brunei Darussalam | 27.9 (26.4 to 29.3) | 20.8 (19.6 to 22.0) | -1.0 (-1.1 to -0.9) | 2.4 (1.7 to 3.2) | 1.3 (0.9 to 1.8) | -2.0 (-2.1 to -1.8) |
| Bulgaria | 564.0 (530.4 to 598.2) | 457.8 (429.3 to 487.1) | -0.7 (-0.8 to -0.7) | 38.8 (27.7 to 52.8) | 23.3 (15.8 to 32.5) | -1.8 (-1.9 to -1.7) |
| Burkina Faso | 168.7 (160.2 to 179.3) | 174.8 (165.0 to 187.1) | 0.1 (0.0 to 0.1) | 20.1 (14.0 to 27.1) | 19.1 (13.0 to 25.6) | -0.3 (-0.4 to -0.2) |
| Burundi | 156.3 (148.9 to 164.7) | 861.5 (453.8 to 1585.7) | 5.6 (3.9 to 7.4) | 20.3 (13.8 to 27.5) | 101.6 (44.7 to 190.5) | 5.2 (3.4 to 7.0) |
| Cabo Verde | 152.4 (145.8 to 160.3) | 160.4 (151.2 to 169.0) | 0.2 (0.2 to 0.3) | 15.1 (10.7 to 19.9) | 11.8 (8.2 to 15.7) | -0.9 (-1.0 to -0.9) |
| Cambodia | 659.6 (291.2 to 1639.2) | 429.2 (259.9 to 845.7) | -1.5 (-1.5 to -1.4) | 84.1 (34.7 to 223.3) | 39.9 (21.5 to 82.7) | -2.5 (-2.7 to -2.4) |
| Cameroon | 154.7 (145.1 to 165.8) | 169.4 (155.1 to 186.3) | 0.1 (0.1 to 0.2) | 18.0 (12.6 to 24.1) | 17.1 (11.7 to 22.8) | -0.3 (-0.4 to -0.2) |
| Canada | 21.2 (20.1 to 22.5) | 17.6 (16.5 to 18.8) | -0.5 (-0.6 to -0.5) | 0.8 (0.5 to 1.2) | 0.6 (0.4 to 1.0) | -0.6 (-0.6 to -0.5) |
| Central African Republic | 181.2 (172.7 to 190.8) | 285.9 (227.0 to 382.2) | 1.5 (1.3 to 1.8) | 24.3 (16.8 to 32.3) | 37.5 (23.3 to 55.6) | 1.5 (1.2 to 1.7) |
| Chad | 265.8 (190.3 to 393.5) | 250.2 (193.1 to 351.1) | -0.2 (-0.3 to -0.1) | 33.9 (19.4 to 53.4) | 29.2 (17.6 to 44.4) | -0.5 (-0.7 to -0.4) |
| Chile | 22.7 (21.4 to 23.9) | 22.7 (21.1 to 24.7) | 0.1 (0.0 to 0.2) | 1.8 (1.2 to 2.4) | 0.9 (0.6 to 1.4) | -2.2 (-2.3 to -2.0) |
| China | 257.2 (240.2 to 272.5) | 261.4 (242.3 to 279.4) | -0.1 (-0.3 to 0.0) | 24.0 (16.5 to 32.1) | 10.6 (7.0 to 15.4) | -3.2 (-3.5 to -2.9) |
| Colombia | 386.3 (360.7 to 421.5) | 288.1 (253.9 to 338.0) | -1.0 (-1.0 to -0.9) | 36.3 (25.1 to 48.3) | 16.4 (11.1 to 23.0) | -2.6 (-2.6 to -2.5) |
| Comoros | 159.8 (152.7 to 168.6) | 159.1 (147.1 to 175.8) | -0.2 (-0.3 to -0.1) | 19.4 (13.3 to 26.2) | 16.5 (11.2 to 22.1) | -0.7 (-0.8 to -0.6) |
| Congo | 178.4 (169.4 to 187.7) | 283.7 (205.1 to 425.8) | 1.6 (0.7 to 2.5) | 22.1 (15.3 to 29.5) | 28.8 (17.2 to 45.7) | 0.9 (-0.1 to 1.8) |
| Cook Islands | 201.3 (189.9 to 215.9) | 191.0 (175.9 to 215.6) | -0.1 (-0.2 to 0.0) | 16.4 (11.3 to 21.8) | 9.7 (6.6 to 13.6) | -1.6 (-1.7 to -1.5) |
| Costa Rica | 269.8 (253.7 to 285.4) | 246.8 (232.8 to 263.6) | -0.3 (-0.3 to -0.3) | 19.2 (13.5 to 25.4) | 12.1 (8.3 to 16.9) | -1.6 (-1.6 to -1.5) |
| Coted'Ivoire | 160.4 (153.1 to 169.6) | 165.2 (155.0 to 176.8) | 0.0 (-0.1 to 0.1) | 18.5 (12.9 to 24.3) | 16.4 (11.4 to 22.1) | -0.5 (-0.6 to -0.4) |
| Croatia | 592.6 (556.5 to 629.7) | 532.1 (476.1 to 590.8) | -0.6 (-0.8 to -0.5) | 30.1 (20.5 to 41.4) | 19.2 (12.0 to 29.1) | -1.8 (-2.0 to -1.7) |
| Cuba | 278.4 (260.8 to 296.0) | 288.0 (264.0 to 311.9) | 0.0 (0.0 to 0.1) | 19.3 (13.4 to 25.4) | 13.3 (8.8 to 18.7) | -1.2 (-1.2 to -1.1) |
| Cyprus | 25.6 (24.1 to 27.0) | 19.8 (18.4 to 21.3) | -1.0 (-1.1 to -0.8) | 1.4 (0.9 to 1.9) | 0.7 (0.5 to 1.1) | -2.1 (-2.2 to -2.0) |
| Czechia | 704.7 (652.8 to 759.5) | 475.9 (444.1 to 509.7) | -1.3 (-1.5 to -1.2) | 40.5 (28.3 to 55.2) | 17.2 (10.8 to 26.0) | -2.5 (-2.7 to -2.2) |
| Democratic People's Republic of Korea | 198.8 (187.8 to 210.3) | 184.1 (173.1 to 195.8) | -0.2 (-0.2 to -0.2) | 18.8 (13.2 to 25.3) | 13.3 (9.3 to 17.9) | -1.1 (-1.2 to -1.1) |
| Democratic Republic of the Congo | 165.3 (156.9 to 174.5) | 222.6 (183.1 to 288.2) | 1.0 (0.6 to 1.3) | 20.4 (14.0 to 27.3) | 24.3 (15.8 to 35.5) | 0.6 (0.2 to 1.0) |
| Denmark | 23.0 (21.6 to 24.6) | 16.3 (15.0 to 17.6) | -1.3 (-1.3 to -1.2) | 1.0 (0.6 to 1.5) | 0.6 (0.4 to 0.9) | -1.6 (-1.7 to -1.5) |
| Djibouti | 149.8 (142.7 to 158.4) | 167.3 (152.1 to 193.4) | -0.1 (-0.3 to 0.1) | 17.7 (12.3 to 23.6) | 17.0 (11.8 to 22.8) | -0.6 (-0.9 to -0.3) |
| Dominica | 179.4 (170.2 to 190.4) | 193.1 (179.7 to 217.6) | 0.2 (0.1 to 0.2) | 17.0 (11.7 to 22.8) | 15.9 (10.9 to 21.2) | -0.3 (-0.3 to -0.2) |
| Dominican Republic | 188.5 (180.1 to 197.7) | 224.2 (213.8 to 236.4) | 0.6 (0.5 to 0.8) | 19.7 (13.6 to 26.4) | 18.2 (12.6 to 24.2) | -0.1 (-0.2 to 0.0) |
| Ecuador | 268.6 (252.6 to 290.8) | 267.1 (251.9 to 284.3) | 0.0 (-0.1 to 0.2) | 28.1 (19.7 to 37.4) | 18.7 (13.0 to 25.2) | -1.2 (-1.4 to -1.1) |
| Egypt | 297.3 (279.3 to 333.0) | 268.1 (242.1 to 352.7) | -0.3 (-0.3 to -0.2) | 31.4 (21.5 to 41.3) | 18.9 (12.7 to 26.2) | -1.4 (-1.5 to -1.4) |
| El Salvador | 1026.8 (601.3 to 1738.7) | 589.6 (406.2 to 909.1) | -1.9 (-2.0 to -1.9) | 108.9 (54.1 to 197.7) | 38.7 (21.8 to 64.4) | -3.4 (-3.5 to -3.4) |
| Equatorial Guinea | 170.3 (161.9 to 179.7) | 143.3 (134.7 to 151.8) | -0.6 (-0.6 to -0.5) | 22.3 (15.6 to 29.7) | 12.2 (8.4 to 16.2) | -2.2 (-2.3 to -2.1) |
| Eritrea | 1572.4 (743.6 to 2974.0) | 898.9 (449.7 to 1751.3) | -2.0 (-2.2 to -1.9) | 211.4 (84.9 to 418.3) | 103.0 (43.6 to 215.4) | -2.5 (-2.7 to -2.3) |
| Estonia | 653.5 (612.1 to 692.0) | 383.6 (357.7 to 409.5) | -2.2 (-2.4 to -2.0) | 41.4 (28.7 to 56.6) | 14.0 (8.7 to 21.3) | -4.4 (-4.7 to -4.0) |
| Eswatini | 200.0 (191.1 to 209.9) | 201.0 (191.0 to 210.7) | 0.1 (-0.1 to 0.3) | 22.6 (15.6 to 29.9) | 20.3 (14.1 to 26.9) | -0.3 (-0.6 to 0.0) |
| Ethiopia | 276.0 (237.7 to 344.0) | 229.4 (173.8 to 348.2) | -0.9 (-1.1 to -0.7) | 37.3 (25.2 to 52.4) | 23.3 (14.6 to 39.3) | -1.9 (-2.1 to -1.6) |
| Fiji | 140.1 (132.9 to 148.4) | 129.2 (120.7 to 139.5) | -0.3 (-0.4 to -0.3) | 14.4 (10.0 to 19.0) | 11.9 (8.2 to 16.0) | -0.7 (-0.7 to -0.6) |
| Finland | 30.7 (28.5 to 32.8) | 24.3 (22.4 to 26.1) | -0.9 (-1.3 to -0.5) | 1.4 (0.9 to 2.0) | 0.9 (0.5 to 1.4) | -1.2 (-1.5 to -0.9) |
| France | 27.8 (26.1 to 29.5) | 21.8 (20.1 to 23.5) | -0.8 (-0.8 to -0.7) | 1.2 (0.7 to 1.8) | 0.8 (0.5 to 1.2) | -1.0 (-1.1 to -0.9) |
| Gabon | 181.2 (172.4 to 190.4) | 164.7 (154.7 to 173.6) | -0.4 (-0.4 to -0.3) | 20.9 (14.4 to 27.8) | 14.8 (10.3 to 19.7) | -1.2 (-1.3 to -1.1) |
| Gambia | 163.7 (141.2 to 199.5) | 150.6 (137.0 to 167.7) | -0.3 (-0.4 to -0.3) | 18.7 (12.5 to 26.2) | 14.9 (10.3 to 19.9) | -0.8 (-0.9 to -0.8) |
| Georgia | 350.5 (330.1 to 372.0) | 410.1 (379.5 to 448.8) | 0.7 (0.5 to 0.8) | 26.0 (18.2 to 35.5) | 26.2 (18.0 to 36.0) | 0.3 (0.2 to 0.5) |
| Germany | 21.6 (20.0 to 23.1) | 17.6 (16.3 to 19.0) | -0.8 (-0.8 to -0.7) | 1.0 (0.6 to 1.4) | 0.6 (0.4 to 1.0) | -1.0 (-1.2 to -0.9) |
| Ghana | 137.0 (129.9 to 144.9) | 140.0 (131.6 to 148.3) | 0.0 (0.0 to 0.1) | 15.6 (10.9 to 21.0) | 13.5 (9.2 to 18.0) | -0.4 (-0.5 to -0.3) |
| Greece | 23.7 (22.0 to 25.3) | 17.5 (16.2 to 18.6) | -1.1 (-1.2 to -1.0) | 0.9 (0.5 to 1.3) | 0.6 (0.4 to 1.0) | -1.1 (-1.2 to -1.0) |
| Greenland | 32.7 (30.6 to 34.7) | 24.9 (23.4 to 26.6) | -1.0 (-1.1 to -1.0) | 2.7 (1.8 to 3.6) | 1.4 (0.9 to 1.9) | -2.4 (-2.5 to -2.3) |
| Grenada | 315.1 (239.9 to 450.7) | 256.9 (219.0 to 321.4) | -0.7 (-0.8 to -0.7) | 31.2 (19.6 to 48.9) | 19.1 (12.9 to 27.0) | -1.5 (-1.5 to -1.4) |
| Guam | 171.3 (162.6 to 181.4) | 165.4 (154.6 to 177.6) | 0.0 (-0.1 to 0.0) | 12.4 (8.7 to 16.5) | 10.5 (7.0 to 14.3) | -0.4 (-0.6 to -0.3) |
| Guatemala | 595.2 (400.9 to 946.4) | 454.3 (363.6 to 629.1) | -0.9 (-1.0 to -0.9) | 71.4 (42.6 to 121.3) | 38.6 (25.8 to 56.9) | -2.0 (-2.1 to -2.0) |
| Guinea | 145.4 (138.8 to 153.9) | 164.6 (154.9 to 175.6) | 0.5 (0.4 to 0.6) | 18.2 (12.6 to 24.3) | 18.4 (12.5 to 24.4) | 0.1 (0.0 to 0.3) |
| Guinea-Bissau | 192.4 (183.5 to 202.4) | 192.2 (177.9 to 212.4) | 0.1 (-0.2 to 0.3) | 25.4 (17.6 to 34.0) | 22.4 (15.5 to 30.2) | -0.4 (-0.6 to -0.1) |
| Guyana | 250.8 (237.8 to 264.8) | 280.3 (266.2 to 294.5) | 0.3 (0.2 to 0.4) | 28.4 (19.6 to 37.8) | 26.4 (18.3 to 34.4) | -0.2 (-0.3 to -0.1) |
| Haiti | 248.2 (236.9 to 262.2) | 416.8 (284.2 to 748.8) | 2.5 (1.9 to 3.2) | 33.5 (23.2 to 44.7) | 49.4 (28.2 to 93.0) | 2.1 (1.5 to 2.8) |
| Honduras | 274.0 (262.3 to 287.2) | 276.8 (259.4 to 306.6) | 0.0 (-0.2 to 0.1) | 31.4 (22.1 to 41.8) | 25.6 (18.2 to 34.5) | -0.7 (-0.9 to -0.6) |
| Hungary | 698.5 (646.0 to 751.5) | 447.9 (417.2 to 483.0) | -1.8 (-1.9 to -1.6) | 45.0 (31.5 to 61.0) | 16.2 (10.2 to 24.4) | -3.6 (-3.7 to -3.5) |
| Iceland | 23.6 (22.0 to 25.5) | 19.2 (17.2 to 21.8) | -0.8 (-0.9 to -0.7) | 0.9 (0.5 to 1.4) | 0.7 (0.4 to 1.1) | -0.8 (-0.9 to -0.7) |
| India | 286.1 (262.9 to 313.2) | 275.0 (251.2 to 302.7) | -0.3 (-0.3 to -0.2) | 32.5 (22.4 to 44.1) | 22.9 (16.1 to 30.6) | -1.3 (-1.4 to -1.2) |
| Indonesia | 229.1 (204.9 to 277.3) | 175.4 (157.9 to 201.7) | -1.0 (-1.0 to -0.9) | 25.5 (17.8 to 36.1) | 15.4 (10.6 to 20.7) | -1.7 (-1.8 to -1.6) |
| Iran (Islamic Republic of) | 582.8 (481.4 to 743.4) | 374.6 (324.0 to 448.7) | -1.6 (-1.7 to -1.5) | 51.2 (33.5 to 73.9) | 18.3 (11.9 to 26.8) | -3.4 (-3.5 to -3.2) |
| Iraq | 1353.0 (792.9 to 2237.9) | 1202.7 (723.3 to 1976.7) | -0.1 (-0.3 to 0.0) | 129.1 (61.0 to 226.1) | 72.7 (34.3 to 123.9) | -1.8 (-1.8 to -1.7) |
| Ireland | 20.5 (19.2 to 21.7) | 17.0 (15.7 to 18.3) | -0.7 (-0.8 to -0.5) | 0.9 (0.6 to 1.3) | 0.6 (0.4 to 1.0) | -0.9 (-1.0 to -0.8) |
| Israel | 18.7 (17.6 to 20.0) | 19.2 (16.3 to 24.4) | 0.3 (0.1 to 0.4) | 0.9 (0.6 to 1.3) | 0.7 (0.4 to 1.1) | -0.6 (-0.7 to -0.4) |
| Italy | 28.8 (26.8 to 30.8) | 19.4 (17.8 to 20.8) | -1.5 (-1.6 to -1.4) | 1.1 (0.7 to 1.6) | 0.7 (0.4 to 1.1) | -1.6 (-1.7 to -1.5) |
| Jamaica | 196.2 (185.3 to 209.7) | 196.5 (184.3 to 211.0) | -0.3 (-0.4 to -0.1) | 16.1 (11.1 to 21.6) | 13.3 (9.1 to 17.5) | -0.9 (-1.0 to -0.8) |
| Japan | 21.4 (20.2 to 22.6) | 15.8 (14.7 to 17.0) | -1.1 (-1.2 to -1.0) | 0.8 (0.5 to 1.2) | 0.6 (0.4 to 0.9) | -1.1 (-1.2 to -1.0) |
| Jordan | 230.8 (219.2 to 242.8) | 177.4 (168.0 to 187.5) | -0.9 (-1.0 to -0.9) | 19.7 (13.5 to 26.0) | 8.8 (5.9 to 12.2) | -3.0 (-3.1 to -2.8) |
| Kazakhstan | 393.5 (374.8 to 412.9) | 348.6 (331.0 to 368.4) | -0.4 (-0.6 to -0.2) | 33.6 (23.4 to 44.9) | 20.7 (14.3 to 28.2) | -1.8 (-2.2 to -1.4) |
| Kenya | 162.6 (154.8 to 172.1) | 167.5 (157.6 to 178.3) | 0.0 (-0.1 to 0.1) | 17.4 (12.3 to 23.2) | 16.1 (11.3 to 21.3) | -0.2 (-0.4 to 0.0) |
| Kiribati | 147.0 (139.6 to 155.7) | 128.9 (120.8 to 139.2) | -0.4 (-0.5 to -0.3) | 19.0 (13.3 to 25.7) | 15.0 (10.4 to 19.8) | -0.7 (-0.8 to -0.6) |
| Kuwait | 286.1 (267.4 to 311.1) | 267.8 (231.0 to 325.3) | -1.0 (-1.3 to -0.7) | 16.6 (11.1 to 22.9) | 9.7 (5.8 to 15.3) | -2.9 (-3.2 to -2.6) |
| Kyrgyzstan | 342.2 (325.5 to 359.0) | 244.7 (232.5 to 257.7) | -1.1 (-1.2 to -1.1) | 31.5 (22.5 to 42.1) | 16.9 (11.7 to 22.6) | -2.1 (-2.2 to -2.0) |
| Lao People's Democratic Republic | 205.6 (195.3 to 216.7) | 165.5 (156.2 to 176.6) | -0.8 (-0.9 to -0.8) | 27.4 (19.2 to 36.6) | 16.9 (11.9 to 22.5) | -1.8 (-1.8 to -1.7) |
| Latvia | 732.2 (685.4 to 778.1) | 427.1 (398.8 to 455.7) | -2.3 (-2.5 to -2.1) | 47.3 (33.2 to 63.2) | 18.2 (11.8 to 26.1) | -3.6 (-3.9 to -3.3) |
| Lebanon | 1177.0 (533.3 to 2766.0) | 513.3 (271.0 to 1029.9) | -2.7 (-2.8 to -2.6) | 95.6 (41.5 to 245.3) | 20.9 (9.3 to 45.6) | -5.0 (-5.1 to -4.9) |
| Lesotho | 168.5 (161.5 to 177.3) | 235.4 (224.9 to 247.1) | 1.3 (1.1 to 1.5) | 19.7 (13.8 to 25.9) | 26.2 (18.3 to 34.7) | 1.2 (0.9 to 1.5) |
| Liberia | 169.8 (147.4 to 202.1) | 359.0 (227.5 to 585.9) | 1.4 (0.6 to 2.3) | 20.9 (14.2 to 29.1) | 35.8 (18.3 to 60.6) | 0.7 (-0.2 to 1.5) |
| Libya | 335.5 (304.6 to 375.3) | 523.5 (420.1 to 681.5) | 1.8 (1.5 to 2.1) | 28.7 (20.1 to 38.9) | 30.7 (19.4 to 45.0) | 0.5 (0.1 to 0.8) |
| Lithuania | 658.8 (612.8 to 700.5) | 454.5 (422.3 to 481.9) | -1.4 (-1.6 to -1.2) | 40.7 (28.4 to 55.4) | 20.0 (13.4 to 28.4) | -2.4 (-2.6 to -2.2) |
| Luxembourg | 27.9 (26.1 to 29.6) | 20.4 (18.9 to 21.9) | -1.1 (-1.2 to -1.0) | 1.3 (0.8 to 1.8) | 0.8 (0.5 to 1.2) | -1.4 (-1.6 to -1.2) |
| Madagascar | 131.9 (126.3 to 138.4) | 117.5 (112.0 to 124.4) | -0.4 (-0.5 to -0.4) | 16.2 (11.2 to 21.3) | 12.9 (8.8 to 17.6) | -0.8 (-0.8 to -0.7) |
| Malawi | 127.7 (121.9 to 134.1) | 121.8 (114.7 to 128.8) | -0.3 (-0.3 to -0.2) | 15.9 (11.0 to 21.2) | 13.0 (9.1 to 17.3) | -0.8 (-0.9 to -0.7) |
| Malaysia | 189.0 (179.7 to 200.2) | 183.5 (172.7 to 195.0) | -0.1 (-0.2 to -0.1) | 17.9 (12.4 to 24.0) | 11.8 (8.1 to 15.8) | -1.5 (-1.5 to -1.4) |
| Maldives | 187.6 (176.3 to 199.4) | 170.7 (157.7 to 187.2) | -0.2 (-0.3 to -0.1) | 20.0 (14.1 to 27.3) | 9.5 (6.3 to 13.1) | -2.5 (-2.6 to -2.3) |
| Mali | 161.5 (153.3 to 170.4) | 240.6 (187.8 to 325.8) | 1.4 (0.9 to 1.8) | 19.8 (13.7 to 26.3) | 25.8 (16.2 to 38.7) | 1.0 (0.5 to 1.4) |
| Malta | 20.4 (18.9 to 21.8) | 17.9 (16.4 to 19.4) | -0.4 (-0.5 to -0.3) | 0.9 (0.6 to 1.3) | 0.7 (0.4 to 1.0) | -0.8 (-0.9 to -0.6) |
| Marshall Islands | 192.1 (183.2 to 202.8) | 182.4 (173.4 to 192.2) | -0.2 (-0.2 to -0.1) | 22.3 (15.6 to 30.0) | 18.6 (13.0 to 24.5) | -0.6 (-0.7 to -0.5) |
| Mauritania | 185.5 (175.0 to 195.1) | 154.6 (145.7 to 164.4) | -0.7 (-0.7 to -0.6) | 22.1 (15.3 to 29.3) | 13.6 (9.2 to 18.1) | -1.6 (-1.7 to -1.6) |
| Mauritius | 146.6 (139.4 to 154.8) | 139.7 (131.7 to 148.0) | -0.1 (-0.1 to 0.0) | 12.4 (8.6 to 16.6) | 8.9 (6.0 to 12.0) | -0.8 (-0.9 to -0.7) |
| Mexico | 437.4 (409.6 to 466.8) | 291.8 (274.4 to 308.9) | -0.6 (-0.9 to -0.3) | 42.2 (29.3 to 56.9) | 20.0 (14.0 to 26.7) | -1.6 (-1.9 to -1.3) |
| Micronesia (Federated States of) | 205.1 (195.7 to 215.9) | 208.8 (196.9 to 223.1) | 0.1 (0.1 to 0.2) | 24.1 (16.6 to 32.3) | 20.2 (14.1 to 26.8) | -0.5 (-0.6 to -0.5) |
| Monaco | 17.6 (16.3 to 18.8) | 16.6 (15.4 to 17.9) | -0.1 (-0.2 to -0.1) | 0.7 (0.4 to 1.0) | 0.6 (0.4 to 0.9) | -0.1 (-0.2 to -0.1) |
| Mongolia | 346.8 (328.9 to 365.9) | 364.9 (345.1 to 384.7) | 0.2 (0.1 to 0.3) | 39.7 (28.1 to 53.3) | 29.3 (20.5 to 39.0) | -1.1 (-1.3 to -1.0) |
| Montenegro | 509.8 (476.1 to 542.4) | 440.2 (410.7 to 468.1) | -0.5 (-0.5 to -0.4) | 24.4 (16.3 to 34.4) | 16.0 (9.8 to 24.3) | -1.8 (-2.0 to -1.6) |
| Morocco | 305.2 (287.4 to 324.4) | 253.7 (239.9 to 269.6) | -0.7 (-0.8 to -0.6) | 32.0 (22.4 to 42.9) | 18.8 (12.9 to 25.0) | -1.8 (-1.9 to -1.7) |
| Mozambique | 525.3 (276.8 to 1013.7) | 326.1 (215.9 to 542.1) | -1.6 (-1.7 to -1.6) | 68.1 (31.8 to 138.3) | 37.0 (20.9 to 65.4) | -2.0 (-2.0 to -1.9) |
| Myanmar | 286.3 (225.6 to 451.7) | 284.7 (228.3 to 384.0) | 0.0 (-0.2 to 0.2) | 35.8 (22.7 to 62.5) | 26.9 (17.1 to 42.1) | -1.0 (-1.1 to -0.9) |
| Namibia | 338.0 (249.1 to 490.4) | 240.7 (202.6 to 307.9) | -1.2 (-1.3 to -1.1) | 39.1 (23.2 to 60.9) | 21.8 (14.4 to 30.8) | -2.0 (-2.2 to -1.8) |
| Nauru | 228.9 (217.7 to 240.6) | 228.6 (216.3 to 241.6) | -0.1 (-0.2 to 0.0) | 25.2 (17.4 to 33.6) | 21.7 (15.1 to 29.0) | -0.5 (-0.7 to -0.3) |
| Nepal | 201.9 (187.8 to 217.4) | 271.1 (232.4 to 326.5) | 1.3 (1.1 to 1.5) | 24.4 (16.7 to 33.3) | 24.5 (16.4 to 33.6) | 0.3 (0.1 to 0.5) |
| Netherlands | 16.6 (15.5 to 17.7) | 16.2 (14.9 to 17.7) | 0.0 (-0.3 to 0.4) | 0.6 (0.4 to 0.9) | 0.6 (0.4 to 0.9) | 0.0 (-0.3 to 0.3) |
| New Zealand | 40.4 (37.9 to 42.8) | 32.0 (29.8 to 34.5) | -0.7 (-0.8 to -0.7) | 1.6 (1.0 to 2.4) | 1.2 (0.7 to 1.8) | -1.0 (-1.0 to -0.9) |
| Nicaragua | 948.2 (484.2 to 1784.0) | 530.6 (323.0 to 895.3) | -1.9 (-2.0 to -1.8) | 95.8 (43.8 to 188.6) | 37.5 (19.9 to 66.5) | -3.1 (-3.2 to -3.1) |
| Niger | 157.8 (149.7 to 167.0) | 162.7 (149.2 to 178.5) | 0.0 (-0.1 to 0.1) | 20.1 (14.2 to 27.0) | 18.3 (12.6 to 24.2) | -0.5 (-0.5 to -0.4) |
| Nigeria | 152.3 (144.1 to 161.4) | 156.9 (143.9 to 171.8) | 0.0 (0.0 to 0.1) | 17.3 (12.1 to 23.3) | 14.7 (10.3 to 19.4) | -0.6 (-0.7 to -0.5) |
| Niue | 175.2 (166.6 to 184.9) | 167.8 (157.3 to 181.1) | -0.2 (-0.3 to -0.1) | 16.7 (11.5 to 22.1) | 12.6 (8.6 to 16.8) | -1.0 (-1.1 to -0.9) |
| North Macedonia | 393.4 (372.4 to 417.5) | 381.6 (356.3 to 409.2) | -0.1 (-0.2 to 0.1) | 30.1 (20.9 to 40.0) | 17.6 (11.7 to 24.9) | -2.0 (-2.1 to -2.0) |
| Northern Mariana Islands | 257.9 (244.2 to 272.9) | 237.0 (223.0 to 253.0) | -0.3 (-0.4 to -0.3) | 19.4 (13.4 to 25.9) | 15.1 (10.4 to 20.2) | -0.6 (-0.7 to -0.5) |
| Norway | 29.1 (27.3 to 31.2) | 18.9 (17.5 to 20.5) | -1.4 (-1.5 to -1.3) | 1.1 (0.7 to 1.7) | 0.7 (0.4 to 1.0) | -1.5 (-1.6 to -1.4) |
| Oman | 501.4 (469.8 to 531.3) | 346.0 (325.4 to 366.5) | -1.4 (-1.6 to -1.2) | 42.2 (29.3 to 56.0) | 15.6 (10.4 to 22.4) | -3.3 (-3.6 to -3.1) |
| Pakistan | 144.2 (137.1 to 152.2) | 181.0 (156.3 to 220.3) | 0.9 (0.8 to 1.0) | 16.7 (11.9 to 22.4) | 18.1 (12.2 to 24.6) | 0.4 (0.3 to 0.5) |
| Palau | 277.3 (260.5 to 294.8) | 283.5 (265.9 to 303.0) | 0.1 (0.0 to 0.1) | 25.3 (17.6 to 33.5) | 21.1 (14.8 to 28.7) | -0.5 (-0.5 to -0.5) |
| Palestine | 812.7 (482.1 to 1347.1) | 847.9 (493.7 to 1438.0) | 0.9 (0.5 to 1.2) | 73.9 (37.6 to 127.2) | 51.1 (24.0 to 91.6) | -0.4 (-0.8 to 0.0) |
| Panama | 287.0 (257.3 to 367.5) | 231.1 (212.8 to 266.5) | -0.8 (-0.8 to -0.7) | 24.4 (16.7 to 35.7) | 13.4 (9.3 to 18.8) | -1.9 (-1.9 to -1.8) |
| Papua New Guinea | 182.2 (172.2 to 193.1) | 219.9 (204.5 to 244.7) | 0.6 (0.5 to 0.6) | 20.5 (14.0 to 27.3) | 23.0 (15.6 to 30.8) | 0.4 (0.3 to 0.5) |
| Paraguay | 243.2 (227.5 to 263.1) | 251.3 (237.1 to 267.3) | 0.1 (0.0 to 0.2) | 22.7 (16.0 to 30.8) | 17.9 (12.7 to 24.0) | -0.8 (-0.9 to -0.6) |
| Peru | 288.3 (245.2 to 364.7) | 247.2 (218.7 to 297.8) | -0.6 (-0.7 to -0.5) | 30.3 (20.1 to 43.4) | 14.6 (9.8 to 20.9) | -2.6 (-2.7 to -2.4) |
| Philippines | 266.4 (221.5 to 355.0) | 211.2 (179.1 to 267.4) | -0.8 (-0.9 to -0.8) | 26.6 (18.0 to 39.7) | 18.7 (12.7 to 26.6) | -1.1 (-1.2 to -1.1) |
| Poland | 595.3 (554.2 to 633.9) | 453.5 (419.7 to 485.2) | -1.0 (-1.1 to -0.9) | 42.7 (30.2 to 57.2) | 17.0 (10.9 to 25.4) | -3.2 (-3.2 to -3.1) |
| Portugal | 27.9 (26.1 to 30.4) | 16.2 (15.1 to 17.2) | -2.0 (-2.1 to -1.9) | 1.6 (1.1 to 2.2) | 0.6 (0.4 to 0.9) | -3.2 (-3.5 to -2.9) |
| Puerto Rico | 240.8 (228.9 to 253.4) | 247.2 (229.1 to 269.9) | 0.0 (0.0 to 0.1) | 16.4 (11.2 to 21.8) | 9.7 (6.2 to 14.6) | -2.0 (-2.1 to -1.9) |
| Qatar | 360.0 (340.2 to 380.8) | 292.3 (274.3 to 312.0) | -0.7 (-0.9 to -0.6) | 26.9 (18.7 to 35.8) | 10.5 (6.6 to 16.1) | -3.4 (-3.7 to -3.1) |
| Republic of Korea | 32.7 (30.7 to 34.3) | 20.8 (19.5 to 22.2) | -1.8 (-1.9 to -1.7) | 2.4 (1.6 to 3.3) | 0.8 (0.5 to 1.1) | -3.8 (-4.2 to -3.5) |
| Republic of Moldova | 519.9 (489.8 to 546.8) | 344.1 (324.3 to 366.4) | -1.5 (-1.6 to -1.4) | 40.2 (28.1 to 53.4) | 18.3 (12.2 to 25.3) | -2.7 (-2.9 to -2.5) |
| Romania | 604.7 (563.0 to 641.4) | 455.8 (425.1 to 485.0) | -1.0 (-1.1 to -1.0) | 47.8 (33.8 to 63.5) | 19.4 (12.8 to 28.1) | -3.1 (-3.2 to -3.1) |
| Russian Federation | 649.9 (609.3 to 686.0) | 524.1 (485.6 to 559.5) | -1.0 (-1.4 to -0.6) | 44.6 (31.2 to 59.7) | 22.4 (14.6 to 32.1) | -2.8 (-3.4 to -2.2) |
| Rwanda | 181.2 (172.2 to 191.9) | 874.2 (406.5 to 1700.9) | 2.2 (-0.3 to 4.8) | 24.1 (16.7 to 32.5) | 91.3 (37.4 to 183.0) | 1.3 (-1.3 to 3.9) |
| Saint Kitts and Nevis | 241.2 (227.6 to 256.5) | 240.3 (224.8 to 259.6) | 0.1 (-0.1 to 0.2) | 25.3 (17.5 to 34.6) | 17.2 (12.0 to 23.1) | -1.2 (-1.2 to -1.2) |
| Saint Lucia | 205.5 (195.2 to 216.8) | 200.5 (191.0 to 210.8) | -0.1 (-0.2 to -0.1) | 19.6 (13.6 to 25.7) | 14.1 (9.8 to 18.5) | -1.1 (-1.1 to -1.0) |
| Saint Vincent and the Grenadines | 196.9 (186.9 to 207.3) | 212.6 (201.7 to 225.0) | 0.2 (0.1 to 0.3) | 18.7 (13.0 to 25.2) | 16.6 (11.7 to 22.0) | -0.5 (-0.5 to -0.4) |
| Samoa | 176.3 (167.8 to 186.3) | 195.4 (177.5 to 231.0) | 0.5 (0.4 to 0.6) | 17.8 (12.5 to 23.9) | 16.3 (11.3 to 22.1) | -0.1 (-0.2 to 0.0) |
| San Marino | 19.2 (17.8 to 20.4) | 17.5 (16.3 to 18.8) | -0.2 (-0.3 to -0.2) | 0.7 (0.4 to 1.1) | 0.7 (0.4 to 1.0) | -0.3 (-0.3 to -0.2) |
| Sao Tome and Principe | 143.4 (134.1 to 152.5) | 178.0 (164.4 to 191.4) | 0.6 (0.6 to 0.7) | 16.0 (10.8 to 21.4) | 15.5 (10.7 to 20.4) | -0.2 (-0.3 to -0.1) |
| Saudi Arabia | 520.1 (488.4 to 550.8) | 628.6 (583.4 to 674.8) | 0.8 (0.8 to 0.9) | 49.3 (34.1 to 65.3) | 31.2 (21.3 to 44.0) | -1.4 (-1.5 to -1.3) |
| Senegal | 130.2 (122.9 to 138.0) | 137.4 (127.6 to 147.4) | 0.1 (0.0 to 0.2) | 15.3 (10.4 to 20.6) | 13.7 (9.5 to 18.3) | -0.3 (-0.4 to -0.3) |
| Serbia | 464.2 (436.7 to 491.5) | 445.6 (401.2 to 506.0) | -0.2 (-0.4 to 0.1) | 32.8 (22.8 to 44.1) | 16.7 (10.4 to 25.4) | -2.5 (-2.7 to -2.2) |
| Seychelles | 187.0 (176.0 to 201.0) | 164.7 (155.9 to 174.5) | -0.5 (-0.5 to -0.4) | 17.4 (12.0 to 23.2) | 11.2 (7.8 to 15.2) | -1.4 (-1.5 to -1.4) |
| Sierra Leone | 134.4 (126.9 to 142.0) | 275.9 (195.8 to 415.6) | 1.8 (0.9 to 2.6) | 16.1 (10.9 to 21.9) | 29.4 (16.8 to 46.7) | 1.4 (0.5 to 2.3) |
| Singapore | 19.3 (18.3 to 20.4) | 16.2 (15.1 to 17.5) | -0.7 (-0.8 to -0.6) | 1.1 (0.7 to 1.6) | 0.6 (0.4 to 0.9) | -1.9 (-2.1 to -1.7) |
| Slovakia | 649.3 (603.1 to 692.6) | 518.3 (482.0 to 555.3) | -0.7 (-0.8 to -0.6) | 41.3 (29.0 to 56.0) | 18.8 (11.7 to 28.1) | -2.6 (-2.6 to -2.5) |
| Slovenia | 789.7 (731.8 to 842.8) | 576.9 (533.1 to 623.8) | -0.7 (-0.9 to -0.5) | 38.0 (25.7 to 53.2) | 20.8 (13.1 to 31.0) | -1.6 (-1.7 to -1.4) |
| Solomon Islands | 254.8 (238.8 to 271.3) | 274.7 (253.3 to 299.6) | 0.2 (0.2 to 0.2) | 30.6 (21.1 to 41.0) | 28.9 (19.7 to 38.8) | -0.2 (-0.2 to -0.1) |
| Somalia | 243.9 (169.5 to 580.0) | 332.8 (227.0 to 554.2) | 0.5 (0.3 to 0.6) | 32.6 (18.1 to 85.9) | 42.4 (24.6 to 73.9) | 0.3 (0.2 to 0.5) |
| South Africa | 352.3 (323.7 to 388.0) | 227.3 (211.6 to 247.0) | -1.6 (-1.8 to -1.5) | 34.8 (24.5 to 46.3) | 18.7 (13.0 to 25.1) | -2.3 (-2.6 to -2.0) |
| South Sudan | 232.3 (181.0 to 319.7) | 339.6 (226.2 to 541.2) | 1.5 (1.2 to 1.8) | 28.6 (17.8 to 43.0) | 37.0 (20.2 to 60.4) | 1.1 (0.8 to 1.4) |
| Spain | 22.0 (20.7 to 23.4) | 16.5 (15.1 to 17.7) | -1.1 (-1.2 to -1.0) | 0.9 (0.6 to 1.4) | 0.6 (0.4 to 0.9) | -1.2 (-1.3 to -1.1) |
| Sri Lanka | 354.4 (287.0 to 486.6) | 558.9 (381.6 to 877.3) | 1.3 (1.0 to 1.6) | 32.7 (21.4 to 49.0) | 31.4 (16.5 to 51.6) | -0.5 (-0.8 to -0.1) |
| Sudan | 313.1 (276.9 to 364.0) | 352.7 (279.5 to 469.6) | 0.5 (0.3 to 0.7) | 35.9 (24.2 to 49.3) | 29.8 (18.9 to 44.3) | -0.5 (-0.8 to -0.3) |
| Suriname | 244.6 (202.9 to 402.7) | 225.8 (202.4 to 305.5) | -0.2 (-0.3 to -0.2) | 25.6 (16.5 to 41.6) | 19.6 (13.1 to 27.6) | -0.9 (-0.9 to -0.8) |
| Sweden | 23.1 (21.5 to 24.6) | 17.8 (16.4 to 19.2) | -0.9 (-0.9 to -0.8) | 0.8 (0.5 to 1.3) | 0.7 (0.4 to 1.0) | -0.9 (-0.9 to -0.8) |
| Switzerland | 31.9 (29.6 to 34.3) | 21.4 (19.6 to 23.1) | -1.5 (-1.6 to -1.4) | 1.2 (0.7 to 1.7) | 0.8 (0.5 to 1.2) | -1.5 (-1.6 to -1.4) |
| Syrian Arab Republic | 262.3 (207.5 to 407.0) | 1256.2 (646.5 to 2280.1) | 5.3 (3.8 to 6.9) | 24.6 (15.7 to 42.7) | 73.1 (30.1 to 137.1) | 3.6 (2.0 to 5.3) |
| Taiwan (Province of China) | 281.7 (264.5 to 299.7) | 168.7 (158.4 to 179.4) | -2.0 (-2.2 to -1.9) | 16.6 (11.5 to 22.5) | 6.1 (3.8 to 9.2) | -3.6 (-3.9 to -3.4) |
| Tajikistan | 276.2 (262.2 to 291.4) | 335.5 (259.0 to 464.2) | -0.4 (-1.0 to 0.2) | 27.7 (19.5 to 36.7) | 30.1 (18.9 to 44.9) | -0.8 (-1.5 to -0.2) |
| Thailand | 278.8 (264.2 to 296.6) | 232.8 (217.7 to 249.8) | -0.9 (-1.0 to -0.8) | 24.1 (17.0 to 32.5) | 12.2 (8.3 to 16.8) | -2.6 (-2.7 to -2.4) |
| Timor-Leste | 755.5 (419.2 to 1328.4) | 743.0 (404.3 to 1329.3) | -0.2 (-0.7 to 0.3) | 93.9 (42.2 to 167.1) | 73.5 (33.3 to 134.1) | -1.0 (-1.5 to -0.5) |
| Togo | 156.6 (148.4 to 165.9) | 156.3 (148.1 to 165.1) | 0.0 (-0.1 to 0.0) | 18.2 (12.4 to 24.2) | 15.9 (11.1 to 21.3) | -0.5 (-0.6 to -0.4) |
| Tokelau | 164.5 (156.6 to 173.8) | 150.8 (142.7 to 160.2) | -0.3 (-0.4 to -0.3) | 17.5 (12.1 to 23.4) | 11.7 (8.0 to 15.5) | -1.3 (-1.3 to -1.2) |
| Tonga | 139.6 (132.7 to 147.3) | 118.7 (111.8 to 126.9) | -0.5 (-0.6 to -0.5) | 13.2 (9.4 to 17.6) | 9.5 (6.6 to 12.6) | -1.0 (-1.1 to -1.0) |
| Trinidad and Tobago | 204.0 (194.4 to 214.6) | 228.7 (206.9 to 262.2) | 0.5 (0.3 to 0.6) | 19.2 (13.3 to 25.6) | 16.3 (11.2 to 22.6) | -0.6 (-0.7 to -0.5) |
| Tunisia | 285.4 (268.3 to 307.3) | 250.2 (235.1 to 266.8) | -0.5 (-0.5 to -0.4) | 23.5 (16.2 to 31.6) | 12.6 (8.4 to 17.8) | -2.1 (-2.2 to -2.0) |
| Turkey | 208.1 (198.2 to 219.8) | 208.4 (190.7 to 229.8) | 0.0 (0.0 to 0.0) | 20.1 (14.0 to 26.9) | 10.0 (6.6 to 14.1) | -2.4 (-2.5 to -2.3) |
| Turkmenistan | 255.0 (242.3 to 268.2) | 223.0 (209.3 to 240.1) | -0.6 (-0.7 to -0.6) | 24.7 (17.8 to 33.1) | 17.2 (12.0 to 23.5) | -1.4 (-1.5 to -1.3) |
| Tuvalu | 197.8 (188.2 to 208.0) | 186.3 (176.5 to 196.9) | -0.6 (-0.8 to -0.4) | 23.6 (16.5 to 31.9) | 17.3 (12.0 to 23.0) | -1.3 (-1.5 to -1.2) |
| Uganda | 429.2 (243.9 to 775.4) | 277.1 (189.0 to 440.6) | -1.5 (-1.6 to -1.4) | 51.8 (26.0 to 100.8) | 28.5 (16.5 to 49.2) | -2.0 (-2.2 to -1.9) |
| Ukraine | 606.3 (562.5 to 649.4) | 526.9 (487.5 to 566.2) | -1.0 (-1.2 to -0.8) | 39.0 (27.3 to 53.2) | 28.3 (19.5 to 39.3) | -1.7 (-2.0 to -1.4) |
| United Arab Emirates | 370.3 (349.3 to 393.2) | 345.0 (324.3 to 366.9) | -0.3 (-0.3 to -0.3) | 33.5 (22.9 to 44.2) | 20.5 (14.1 to 28.4) | -1.6 (-1.7 to -1.5) |
| United Kingdom | 18.6 (17.4 to 19.8) | 15.9 (14.7 to 17.0) | -0.5 (-0.6 to -0.4) | 0.8 (0.5 to 1.1) | 0.6 (0.4 to 0.9) | -0.9 (-1.0 to -0.9) |
| United Republic of Tanzania | 139.2 (132.7 to 146.7) | 133.2 (126.4 to 141.0) | -0.2 (-0.2 to -0.2) | 16.2 (11.3 to 21.7) | 13.5 (9.4 to 18.1) | -0.6 (-0.6 to -0.6) |
| United States of America | 23.6 (22.4 to 25.0) | 21.0 (19.5 to 22.4) | -0.5 (-0.7 to -0.4) | 0.9 (0.6 to 1.4) | 0.8 (0.5 to 1.1) | -0.7 (-0.8 to -0.5) |
| United States Virgin Islands | 235.3 (225.3 to 246.5) | 215.0 (204.8 to 225.7) | -0.5 (-0.6 to -0.4) | 19.1 (13.5 to 25.6) | 13.3 (9.2 to 18.0) | -1.4 (-1.4 to -1.3) |
| Uruguay | 28.6 (26.6 to 31.2) | 24.2 (22.8 to 26.0) | -0.7 (-0.7 to -0.6) | 2.1 (1.4 to 2.9) | 1.2 (0.8 to 1.8) | -1.9 (-1.9 to -1.8) |
| Uzbekistan | 263.9 (251.2 to 277.6) | 236.4 (225.0 to 248.8) | -0.4 (-0.5 to -0.3) | 24.1 (16.8 to 31.6) | 18.2 (12.8 to 24.4) | -1.1 (-1.2 to -0.9) |
| Vanuatu | 173.8 (163.2 to 188.7) | 170.0 (159.0 to 186.0) | -0.1 (-0.2 to -0.1) | 19.8 (13.6 to 26.5) | 17.9 (12.0 to 23.6) | -0.3 (-0.4 to -0.3) |
| Venezuela (Bolivarian Republic of) | 324.6 (308.3 to 341.4) | 318.1 (297.6 to 344.5) | 0.0 (-0.1 to 0.1) | 30.6 (21.5 to 40.5) | 22.2 (15.2 to 29.8) | -1.0 (-1.1 to -0.9) |
| Viet Nam | 215.4 (204.1 to 228.4) | 242.4 (226.1 to 259.6) | 0.6 (0.5 to 0.7) | 21.0 (14.8 to 28.4) | 15.1 (10.4 to 20.6) | -1.0 (-1.0 to -0.9) |
| Yemen | 358.6 (317.5 to 424.4) | 512.1 (370.3 to 744.1) | 0.6 (0.1 to 1.0) | 40.9 (27.8 to 56.5) | 47.9 (27.6 to 74.1) | -0.2 (-0.7 to 0.2) |
| Zambia | 147.9 (140.9 to 156.6) | 154.4 (147.1 to 162.9) | 0.1 (0.0 to 0.2) | 18.3 (12.5 to 24.3) | 15.9 (11.2 to 21.3) | -0.6 (-0.7 to -0.4) |
| Zimbabwe | 175.9 (167.8 to 184.1) | 164.6 (157.0 to 173.5) | -0.3 (-0.4 to -0.2) | 18.7 (13.0 to 25.0) | 17.8 (12.5 to 23.6) | 0.0 (-0.1 to 0.2) |

**Table S14. ASR of prevalence and YLDs in 1990 and 2021, and their EAPC from 1990 to 2021 for AMP-LL2, by country.**

| **Countries** | **Prevalence** | | | **YLDs** | | |
| --- | --- | --- | --- | --- | --- | --- |
|  | **Age-standardized prevalence rate, 1990** | **Age-standardized prevalence rate, 2021** | **EAPC of prevalence rate No. (95% CI)** | **Age-standardized YLDs rate, 1990** | **Age-standardized YLDs rate, 2021** | **EAPC of YLDs rate No. (95% CI)** |
| Afghanistan | 569.8 (109.5 to 1898.3) | 509.6 (143.5 to 1487.9) | -0.6 (-0.7 to -0.5) | 167.0 (29.2 to 575.3) | 125.9 (32.6 to 359.9) | -1.2 (-1.3 to -1.1) |
| Albania | 76.9 (70.2 to 84.8) | 76.2 (66.5 to 90.4) | -0.2 (-0.5 to 0.0) | 15.7 (10.6 to 21.2) | 9.8 (6.3 to 14.3) | -1.9 (-2.1 to -1.7) |
| Algeria | 44.7 (40.8 to 49.8) | 41.4 (33.8 to 54.8) | -0.3 (-0.5 to -0.1) | 10.1 (6.8 to 13.9) | 6.6 (4.1 to 10.2) | -1.4 (-1.6 to -1.2) |
| American Samoa | 25.6 (23.2 to 28.2) | 25.8 (22.6 to 29.7) | 0.2 (0.1 to 0.3) | 5.7 (3.8 to 8.1) | 5.1 (3.4 to 7.0) | -0.1 (-0.2 to 0.0) |
| Andorra | 85.1 (74.2 to 99.5) | 81.4 (70.4 to 96.1) | -0.2 (-0.3 to -0.1) | 9.8 (6.3 to 13.8) | 9.3 (6.0 to 13.4) | -0.2 (-0.3 to -0.1) |
| Angola | 107.3 (46.7 to 230.5) | 98.8 (42.1 to 216.7) | -0.5 (-0.8 to -0.1) | 32.6 (12.4 to 71.8) | 24.4 (9.1 to 57.3) | -1.1 (-1.6 to -0.7) |
| Antigua and Barbuda | 30.2 (27.8 to 33.2) | 30.9 (28.3 to 34.1) | 0.0 (-0.1 to 0.2) | 6.1 (4.1 to 8.4) | 5.0 (3.3 to 7.0) | -0.7 (-0.8 to -0.6) |
| Argentina | 67.3 (61.6 to 74.0) | 63.3 (57.6 to 71.9) | -0.2 (-0.4 to 0.0) | 13.8 (9.1 to 19.2) | 10.0 (6.8 to 14.1) | -1.0 (-1.1 to -0.8) |
| Armenia | 53.5 (48.8 to 59.3) | 65.4 (42.3 to 112.7) | -1.0 (-1.5 to -0.4) | 10.5 (7.4 to 14.7) | 9.9 (5.0 to 19.5) | -1.9 (-2.5 to -1.3) |
| Australia | 104.4 (92.0 to 120.1) | 90.6 (78.1 to 107.4) | -0.4 (-0.5 to -0.3) | 11.9 (7.7 to 17.0) | 10.4 (6.6 to 14.8) | -0.4 (-0.5 to -0.3) |
| Austria | 94.1 (83.7 to 108.8) | 74.2 (64.6 to 87.0) | -0.7 (-0.8 to -0.6) | 11.5 (7.6 to 16.3) | 8.6 (5.6 to 12.5) | -0.8 (-0.9 to -0.7) |
| Azerbaijan | 43.3 (39.8 to 48.2) | 49.0 (37.3 to 72.4) | -0.5 (-0.8 to -0.3) | 9.8 (6.7 to 13.4) | 8.8 (5.5 to 14.8) | -1.4 (-1.7 to -1.1) |
| Bahamas | 29.4 (27.2 to 31.9) | 30.6 (27.6 to 33.6) | 0.2 (0.1 to 0.3) | 6.3 (4.4 to 8.7) | 5.5 (3.6 to 7.6) | -0.4 (-0.5 to -0.3) |
| Bahrain | 32.4 (29.0 to 37.8) | 29.0 (25.8 to 32.9) | -2.0 (-2.7 to -1.3) | 6.7 (4.4 to 9.4) | 3.7 (2.4 to 5.4) | -3.6 (-4.3 to -2.9) |
| Bangladesh | 21.4 (19.5 to 23.8) | 20.5 (18.1 to 23.8) | -0.2 (-0.2 to -0.1) | 5.9 (4.1 to 8.2) | 4.2 (2.8 to 6.1) | -1.2 (-1.3 to -1.1) |
| Barbados | 25.6 (23.6 to 27.9) | 25.8 (23.6 to 29.0) | -0.1 (-0.3 to 0.0) | 5.1 (3.4 to 7.0) | 4.1 (2.7 to 5.8) | -0.8 (-0.9 to -0.7) |
| Belarus | 74.0 (67.0 to 82.2) | 64.5 (58.3 to 72.3) | -0.6 (-0.9 to -0.3) | 12.7 (8.6 to 17.7) | 7.6 (5.1 to 10.6) | -2.0 (-2.3 to -1.6) |
| Belgium | 90.3 (80.5 to 107.0) | 78.7 (68.8 to 95.2) | -0.2 (-0.7 to 0.3) | 10.8 (7.2 to 15.5) | 9.0 (6.0 to 12.8) | -0.3 (-0.7 to 0.2) |
| Belize | 32.3 (29.6 to 35.8) | 39.8 (34.9 to 47.9) | 0.3 (0.0 to 0.5) | 7.3 (4.9 to 10.2) | 7.7 (4.9 to 11.3) | -0.4 (-0.6 to -0.1) |
| Benin | 21.1 (19.5 to 23.0) | 19.8 (18.0 to 21.6) | -0.3 (-0.3 to -0.2) | 5.9 (4.0 to 8.3) | 4.9 (3.3 to 6.9) | -0.7 (-0.8 to -0.6) |
| Bermuda | 26.8 (24.8 to 29.2) | 25.8 (23.6 to 29.0) | -0.8 (-1.1 to -0.5) | 4.6 (3.1 to 6.5) | 3.0 (1.9 to 4.3) | -2.1 (-2.3 to -1.9) |
| Bhutan | 22.9 (21.0 to 25.1) | 24.0 (21.6 to 26.6) | 0.1 (0.0 to 0.2) | 6.4 (4.3 to 8.9) | 5.0 (3.3 to 6.9) | -0.9 (-1.0 to -0.8) |
| Bolivia (Plurinational State of) | 32.4 (29.9 to 35.3) | 27.0 (24.4 to 30.3) | -0.7 (-0.7 to -0.6) | 9.1 (6.1 to 12.6) | 5.9 (3.8 to 8.1) | -1.5 (-1.6 to -1.5) |
| Bosnia and Herzegovina | 84.5 (73.1 to 101.4) | 141.8 (82.9 to 260.6) | 0.5 (-0.3 to 1.3) | 15.9 (10.4 to 23.0) | 18.2 (8.9 to 38.0) | -1.1 (-1.9 to -0.3) |
| Botswana | 22.0 (20.2 to 23.6) | 26.0 (23.9 to 28.2) | 0.4 (0.2 to 0.7) | 5.8 (3.8 to 7.9) | 5.8 (4.1 to 8.0) | -0.1 (-0.4 to 0.2) |
| Brazil | 53.2 (48.0 to 59.2) | 46.3 (39.4 to 57.6) | -0.7 (-0.8 to -0.6) | 12.2 (8.4 to 16.7) | 8.2 (5.6 to 12.0) | -1.5 (-1.5 to -1.4) |
| Brunei Darussalam | 74.3 (67.4 to 83.1) | 56.2 (50.8 to 63.9) | -1.0 (-1.0 to -0.9) | 15.6 (10.6 to 21.2) | 9.3 (6.3 to 13.4) | -1.7 (-1.8 to -1.6) |
| Bulgaria | 87.0 (78.5 to 99.2) | 70.2 (63.7 to 80.6) | -0.7 (-0.8 to -0.7) | 15.4 (10.6 to 21.3) | 10.0 (6.7 to 13.7) | -1.5 (-1.5 to -1.4) |
| Burkina Faso | 22.4 (20.7 to 24.6) | 24.9 (21.8 to 29.6) | 0.1 (0.0 to 0.2) | 6.2 (4.1 to 8.7) | 6.4 (4.3 to 9.0) | -0.1 (-0.3 to 0.0) |
| Burundi | 21.0 (19.4 to 23.0) | 279.2 (94.3 to 652.9) | 8.2 (5.8 to 10.6) | 6.3 (4.1 to 8.5) | 75.5 (23.0 to 187.0) | 7.8 (5.4 to 10.3) |
| Cabo Verde | 24.4 (22.1 to 26.6) | 21.9 (19.5 to 24.0) | -0.3 (-0.4 to -0.3) | 5.7 (4.0 to 8.1) | 4.1 (2.7 to 5.8) | -1.2 (-1.3 to -1.1) |
| Cambodia | 177.2 (52.1 to 525.0) | 112.1 (41.4 to 312.1) | -1.6 (-1.7 to -1.4) | 51.4 (13.9 to 157.1) | 24.9 (8.0 to 69.6) | -2.5 (-2.6 to -2.3) |
| Cameroon | 19.3 (17.6 to 21.7) | 24.0 (19.7 to 31.5) | 0.4 (0.3 to 0.5) | 5.2 (3.5 to 7.4) | 5.8 (3.6 to 8.7) | 0.1 (-0.1 to 0.2) |
| Canada | 55.3 (50.7 to 61.6) | 43.5 (39.5 to 48.2) | -0.7 (-0.8 to -0.7) | 6.4 (4.1 to 9.0) | 5.0 (3.2 to 7.1) | -0.7 (-0.8 to -0.7) |
| Central African Republic | 23.0 (21.3 to 24.9) | 57.8 (34.0 to 106.1) | 3.2 (2.7 to 3.7) | 7.0 (4.8 to 9.5) | 17.3 (8.5 to 33.2) | 3.2 (2.7 to 3.7) |
| Chad | 60.0 (31.4 to 119.7) | 58.4 (32.1 to 111.8) | -0.1 (-0.2 to 0.0) | 17.6 (7.8 to 36.8) | 15.8 (7.6 to 32.1) | -0.4 (-0.6 to -0.3) |
| Chile | 67.5 (61.6 to 76.6) | 72.1 (63.4 to 84.3) | 0.3 (0.2 to 0.5) | 13.2 (9.0 to 18.3) | 8.8 (5.7 to 12.7) | -1.3 (-1.4 to -1.2) |
| China | 37.0 (33.9 to 40.8) | 31.2 (28.3 to 35.0) | -0.9 (-1.2 to -0.7) | 8.4 (5.9 to 11.4) | 3.9 (2.6 to 5.4) | -3.1 (-3.5 to -2.8) |
| Colombia | 65.9 (58.2 to 78.6) | 57.7 (44.7 to 82.2) | -0.5 (-0.6 to -0.4) | 15.0 (10.0 to 21.1) | 8.9 (5.5 to 14.4) | -1.7 (-1.8 to -1.6) |
| Comoros | 22.0 (20.3 to 23.9) | 23.5 (19.6 to 30.6) | -0.1 (-0.2 to 0.1) | 6.2 (4.0 to 8.5) | 5.8 (3.7 to 8.9) | -0.5 (-0.7 to -0.4) |
| Congo | 21.3 (19.7 to 23.0) | 66.6 (31.4 to 142.3) | 3.9 (2.4 to 5.5) | 6.1 (4.0 to 8.2) | 16.0 (6.5 to 35.7) | 3.4 (1.7 to 5.0) |
| Cook Islands | 26.6 (24.3 to 29.5) | 24.8 (22.3 to 27.9) | -0.2 (-0.2 to -0.1) | 5.4 (3.7 to 7.4) | 3.5 (2.5 to 5.0) | -1.2 (-1.3 to -1.2) |
| Costa Rica | 41.6 (37.3 to 47.6) | 38.3 (34.7 to 43.9) | -0.3 (-0.4 to -0.2) | 7.6 (5.1 to 10.4) | 5.4 (3.6 to 7.7) | -1.2 (-1.3 to -1.1) |
| Coted'Ivoire | 21.1 (19.3 to 23.1) | 23.4 (20.3 to 28.3) | 0.4 (0.3 to 0.4) | 5.7 (3.9 to 7.8) | 5.5 (3.7 to 8.0) | -0.1 (-0.2 to 0.1) |
| Croatia | 77.0 (70.5 to 86.7) | 80.5 (63.8 to 111.1) | -0.5 (-0.8 to -0.2) | 11.0 (7.3 to 15.4) | 9.3 (5.6 to 14.7) | -1.3 (-1.5 to -1.0) |
| Cuba | 36.2 (33.4 to 39.5) | 35.7 (32.7 to 39.4) | -0.1 (-0.2 to 0.0) | 6.5 (4.4 to 8.7) | 4.8 (3.2 to 7.0) | -0.9 (-1.0 to -0.9) |
| Cyprus | 79.1 (70.4 to 91.5) | 70.0 (61.1 to 82.4) | -0.4 (-0.6 to -0.2) | 11.7 (7.9 to 16.5) | 8.1 (5.1 to 11.5) | -1.1 (-1.2 to -1.0) |
| Czechia | 92.3 (84.2 to 103.9) | 68.9 (62.2 to 78.6) | -0.8 (-1.0 to -0.6) | 14.5 (9.9 to 19.9) | 7.9 (5.2 to 11.3) | -1.5 (-1.7 to -1.3) |
| Democratic People's Republic of Korea | 27.7 (25.7 to 29.8) | 22.4 (20.6 to 24.0) | -0.7 (-0.7 to -0.7) | 6.3 (4.2 to 8.9) | 4.1 (2.9 to 5.6) | -1.4 (-1.5 to -1.4) |
| Democratic Republic of the Congo | 21.4 (19.9 to 23.1) | 42.5 (25.5 to 77.6) | 2.3 (1.6 to 3.0) | 6.1 (4.1 to 8.2) | 10.8 (5.5 to 21.3) | 2.0 (1.2 to 2.7) |
| Denmark | 73.2 (64.8 to 84.4) | 62.6 (53.2 to 78.1) | -0.5 (-0.6 to -0.3) | 9.4 (6.2 to 13.0) | 7.2 (4.7 to 10.7) | -0.7 (-0.8 to -0.6) |
| Djibouti | 21.4 (19.4 to 23.8) | 26.6 (20.6 to 39.3) | 0.1 (-0.3 to 0.4) | 5.9 (3.9 to 8.2) | 6.4 (4.0 to 10.1) | -0.4 (-0.8 to 0.0) |
| Dominica | 27.5 (25.0 to 30.5) | 27.4 (25.0 to 30.2) | 0.0 (-0.2 to 0.1) | 6.2 (4.1 to 8.7) | 5.5 (3.7 to 7.6) | -0.4 (-0.5 to -0.3) |
| Dominican Republic | 27.8 (26.0 to 30.0) | 30.4 (28.0 to 33.3) | 0.3 (0.2 to 0.5) | 6.8 (4.6 to 9.3) | 6.1 (4.1 to 8.4) | -0.3 (-0.4 to -0.2) |
| Ecuador | 34.3 (31.3 to 37.9) | 34.2 (31.0 to 38.2) | 0.0 (-0.2 to 0.2) | 8.4 (5.7 to 11.3) | 6.1 (4.2 to 8.5) | -1.0 (-1.2 to -0.8) |
| Egypt | 39.4 (35.5 to 45.8) | 34.4 (29.0 to 49.6) | -0.4 (-0.5 to -0.3) | 9.8 (6.5 to 13.6) | 6.2 (4.0 to 9.4) | -1.4 (-1.4 to -1.3) |
| El Salvador | 281.2 (118.4 to 614.2) | 167.5 (81.4 to 350.6) | -1.8 (-1.8 to -1.7) | 70.0 (25.9 to 164.7) | 28.4 (12.7 to 62.7) | -3.0 (-3.0 to -2.9) |
| Equatorial Guinea | 22.2 (20.5 to 23.9) | 16.8 (15.3 to 18.2) | -1.0 (-1.1 to -0.9) | 6.6 (4.3 to 9.3) | 3.5 (2.4 to 5.0) | -2.3 (-2.4 to -2.2) |
| Eritrea | 476.9 (149.2 to 1157.2) | 324.9 (106.7 to 782.0) | -1.5 (-1.6 to -1.3) | 144.3 (40.4 to 368.9) | 84.9 (25.3 to 216.8) | -1.9 (-2.1 to -1.7) |
| Estonia | 89.7 (81.6 to 99.2) | 53.6 (48.5 to 59.6) | -2.0 (-2.2 to -1.9) | 14.9 (10.0 to 20.6) | 6.2 (4.1 to 8.7) | -3.5 (-3.7 to -3.2) |
| Eswatini | 28.7 (26.5 to 31.3) | 27.8 (25.5 to 30.2) | -0.1 (-0.2 to 0.1) | 7.5 (5.0 to 10.4) | 6.5 (4.5 to 8.9) | -0.4 (-0.6 to -0.2) |
| Ethiopia | 47.5 (33.6 to 75.5) | 50.0 (26.8 to 104.2) | -0.2 (-0.5 to 0.1) | 14.7 (8.5 to 25.4) | 12.1 (5.7 to 27.1) | -1.1 (-1.4 to -0.7) |
| Fiji | 22.0 (20.0 to 24.0) | 20.2 (18.4 to 22.2) | -0.4 (-0.4 to -0.3) | 5.3 (3.7 to 7.4) | 4.5 (3.0 to 6.2) | -0.6 (-0.7 to -0.5) |
| Finland | 106.4 (92.0 to 124.4) | 90.1 (78.1 to 111.4) | -0.6 (-1.1 to -0.1) | 13.9 (9.1 to 19.4) | 10.3 (6.5 to 15.3) | -0.8 (-1.3 to -0.4) |
| France | 90.6 (80.6 to 103.8) | 75.0 (65.6 to 88.5) | -0.6 (-0.7 to -0.5) | 11.6 (7.5 to 16.6) | 8.6 (5.4 to 12.2) | -0.7 (-0.8 to -0.6) |
| Gabon | 21.4 (19.6 to 23.2) | 18.9 (17.3 to 20.6) | -0.4 (-0.5 to -0.4) | 5.8 (3.9 to 7.9) | 4.1 (2.8 to 5.7) | -1.1 (-1.2 to -1.0) |
| Gambia | 28.9 (20.1 to 45.9) | 23.2 (18.1 to 32.5) | -0.8 (-0.8 to -0.7) | 7.8 (4.3 to 13.8) | 5.5 (3.4 to 8.7) | -1.2 (-1.3 to -1.1) |
| Georgia | 55.6 (50.4 to 63.1) | 65.8 (55.4 to 81.9) | 0.5 (0.4 to 0.6) | 10.4 (7.0 to 14.4) | 11.0 (7.4 to 16.2) | 0.3 (0.2 to 0.4) |
| Germany | 76.2 (67.6 to 89.4) | 65.7 (57.2 to 79.2) | -0.5 (-0.6 to -0.4) | 9.9 (6.5 to 13.8) | 7.5 (4.9 to 10.9) | -0.7 (-0.8 to -0.6) |
| Ghana | 18.1 (16.6 to 19.8) | 17.8 (16.1 to 19.6) | -0.1 (-0.2 to 0.0) | 4.8 (3.2 to 6.7) | 4.1 (2.8 to 5.8) | -0.5 (-0.6 to -0.4) |
| Greece | 85.4 (75.3 to 97.2) | 64.2 (55.7 to 76.5) | -0.9 (-1.1 to -0.8) | 9.9 (6.4 to 14.0) | 7.4 (4.8 to 10.4) | -0.9 (-1.1 to -0.8) |
| Greenland | 87.5 (79.8 to 96.1) | 62.9 (56.9 to 69.8) | -1.3 (-1.4 to -1.2) | 17.5 (12.0 to 23.8) | 9.4 (6.5 to 13.3) | -2.3 (-2.4 to -2.2) |
| Grenada | 65.4 (38.7 to 125.9) | 48.9 (33.5 to 82.2) | -1.0 (-1.0 to -0.9) | 15.5 (7.4 to 31.3) | 9.1 (5.0 to 16.2) | -1.6 (-1.7 to -1.6) |
| Guam | 25.5 (22.9 to 28.6) | 25.8 (22.7 to 29.2) | 0.1 (0.1 to 0.2) | 4.7 (3.2 to 6.7) | 4.3 (3.0 to 6.2) | -0.2 (-0.3 to -0.1) |
| Guatemala | 141.9 (72.8 to 295.3) | 98.0 (58.2 to 188.4) | -1.3 (-1.4 to -1.2) | 39.3 (17.5 to 84.4) | 20.1 (10.4 to 39.6) | -2.2 (-2.3 to -2.2) |
| Guinea | 21.1 (19.4 to 23.3) | 25.2 (21.9 to 30.5) | 0.7 (0.6 to 0.9) | 6.1 (4.2 to 8.6) | 6.6 (4.4 to 9.3) | 0.4 (0.3 to 0.6) |
| Guinea-Bissau | 26.1 (24.0 to 28.1) | 29.2 (23.7 to 40.0) | 0.5 (0.1 to 0.9) | 7.8 (5.3 to 11.0) | 7.9 (5.0 to 11.6) | 0.2 (-0.3 to 0.6) |
| Guyana | 33.6 (30.7 to 37.2) | 39.4 (35.9 to 43.8) | 0.4 (0.3 to 0.6) | 8.8 (5.9 to 12.4) | 8.9 (6.0 to 12.4) | 0.1 (-0.1 to 0.2) |
| Haiti | 35.2 (32.9 to 37.8) | 34.3 (31.1 to 39.1) | -0.2 (-0.2 to -0.1) | 10.7 (6.9 to 14.9) | 9.3 (6.0 to 12.8) | -0.5 (-0.6 to -0.5) |
| Honduras | 47.4 (43.1 to 52.9) | 42.7 (38.1 to 47.9) | -0.4 (-0.5 to -0.3) | 12.6 (8.4 to 17.2) | 9.5 (6.4 to 13.2) | -1.0 (-1.0 to -0.9) |
| Hungary | 82.4 (75.4 to 90.9) | 61.5 (55.8 to 69.5) | -1.1 (-1.1 to -1.0) | 14.0 (9.6 to 19.4) | 7.1 (4.7 to 10.3) | -2.3 (-2.3 to -2.2) |
| Iceland | 80.2 (71.0 to 93.8) | 67.9 (57.7 to 81.5) | -0.5 (-0.6 to -0.4) | 9.3 (6.0 to 13.2) | 7.8 (5.0 to 11.3) | -0.5 (-0.6 to -0.4) |
| India | 39.3 (35.7 to 44.5) | 33.0 (29.1 to 38.1) | -0.8 (-0.8 to -0.7) | 10.4 (7.4 to 14.4) | 6.8 (4.7 to 9.5) | -1.6 (-1.7 to -1.5) |
| Indonesia | 35.1 (28.6 to 52.4) | 24.3 (20.0 to 34.9) | -1.3 (-1.4 to -1.2) | 9.2 (6.0 to 14.6) | 5.2 (3.4 to 8.0) | -1.9 (-2.0 to -1.8) |
| Iran (Islamic Republic of) | 99.2 (66.8 to 159.6) | 63.7 (46.2 to 97.5) | -1.5 (-1.6 to -1.4) | 21.4 (12.1 to 38.6) | 8.8 (5.3 to 15.5) | -2.9 (-3.0 to -2.8) |
| Iraq | 313.2 (134.6 to 652.7) | 312.3 (132.6 to 678.8) | 0.3 (0.1 to 0.4) | 71.3 (25.1 to 162.5) | 50.0 (18.8 to 117.9) | -1.0 (-1.1 to -0.9) |
| Ireland | 74.1 (65.6 to 87.1) | 67.6 (58.6 to 81.5) | -0.3 (-0.5 to -0.1) | 9.3 (6.1 to 13.1) | 7.8 (5.0 to 11.1) | -0.4 (-0.6 to -0.3) |
| Israel | 68.0 (60.7 to 80.4) | 72.5 (58.7 to 95.8) | 0.4 (0.2 to 0.6) | 9.6 (6.3 to 13.6) | 8.4 (5.3 to 13.2) | -0.1 (-0.3 to 0.0) |
| Italy | 90.9 (81.1 to 104.0) | 71.7 (61.3 to 85.2) | -0.9 (-1.0 to -0.8) | 10.8 (7.2 to 15.3) | 8.3 (5.3 to 12.2) | -0.9 (-1.0 to -0.8) |
| Jamaica | 30.3 (27.6 to 33.5) | 30.5 (26.7 to 35.8) | -0.3 (-0.4 to -0.1) | 6.2 (4.1 to 8.4) | 5.3 (3.5 to 7.6) | -0.8 (-0.8 to -0.7) |
| Japan | 65.2 (59.6 to 73.7) | 47.4 (42.4 to 54.2) | -1.1 (-1.3 to -0.9) | 7.7 (5.0 to 10.9) | 5.5 (3.6 to 7.9) | -1.1 (-1.3 to -1.0) |
| Jordan | 32.8 (29.9 to 36.8) | 26.7 (24.0 to 30.2) | -0.8 (-0.8 to -0.7) | 6.9 (4.6 to 9.5) | 3.8 (2.6 to 5.2) | -2.3 (-2.4 to -2.1) |
| Kazakhstan | 61.4 (56.2 to 68.1) | 49.8 (44.9 to 55.5) | -0.8 (-0.9 to -0.6) | 12.8 (8.9 to 17.7) | 7.9 (5.3 to 10.9) | -1.9 (-2.2 to -1.5) |
| Kenya | 20.8 (19.1 to 22.4) | 22.2 (19.4 to 26.7) | 0.1 (0.0 to 0.3) | 5.3 (3.6 to 7.1) | 5.2 (3.5 to 7.3) | 0.0 (-0.2 to 0.2) |
| Kiribati | 25.1 (22.7 to 27.8) | 22.4 (19.3 to 26.7) | -0.4 (-0.5 to -0.2) | 7.3 (5.1 to 10.3) | 6.0 (3.9 to 8.5) | -0.6 (-0.8 to -0.5) |
| Kuwait | 41.2 (35.9 to 49.5) | 49.8 (35.3 to 77.9) | -0.6 (-1.0 to -0.2) | 6.5 (4.3 to 9.5) | 5.7 (3.2 to 10.4) | -1.9 (-2.4 to -1.5) |
| Kyrgyzstan | 54.2 (49.2 to 60.4) | 37.0 (33.1 to 41.6) | -1.3 (-1.4 to -1.3) | 12.0 (8.4 to 16.4) | 6.6 (4.5 to 9.2) | -2.1 (-2.1 to -2.0) |
| Lao People's Democratic Republic | 31.7 (28.9 to 35.0) | 23.5 (20.9 to 26.9) | -1.2 (-1.2 to -1.1) | 9.6 (6.4 to 13.4) | 5.7 (3.8 to 8.2) | -1.9 (-2.0 to -1.8) |
| Latvia | 96.0 (87.1 to 106.0) | 56.7 (51.4 to 63.4) | -2.2 (-2.3 to -2.0) | 16.2 (11.0 to 22.2) | 7.2 (4.7 to 10.1) | -3.0 (-3.2 to -2.8) |
| Lebanon | 300.1 (96.8 to 870.8) | 166.0 (57.1 to 465.6) | -1.9 (-2.0 to -1.9) | 60.9 (17.0 to 181.6) | 20.1 (6.1 to 57.2) | -3.6 (-3.7 to -3.6) |
| Lesotho | 24.7 (22.5 to 26.8) | 33.4 (30.2 to 36.7) | 1.1 (0.9 to 1.3) | 6.6 (4.5 to 9.2) | 8.6 (5.9 to 11.9) | 1.0 (0.8 to 1.3) |
| Liberia | 30.8 (22.9 to 45.8) | 101.6 (42.1 to 223.9) | 2.4 (1.3 to 3.4) | 8.7 (5.4 to 15.0) | 23.9 (8.9 to 57.5) | 1.7 (0.6 to 2.7) |
| Libya | 49.2 (39.2 to 66.7) | 102.8 (64.1 to 185.1) | 2.7 (2.3 to 3.1) | 10.3 (6.5 to 15.6) | 16.5 (8.8 to 32.8) | 1.7 (1.3 to 2.2) |
| Lithuania | 85.2 (77.2 to 94.9) | 57.5 (52.3 to 65.2) | -1.5 (-1.6 to -1.3) | 13.9 (9.4 to 19.0) | 7.4 (5.0 to 10.6) | -2.1 (-2.3 to -1.9) |
| Luxembourg | 93.1 (83.1 to 106.9) | 73.8 (63.5 to 86.2) | -0.8 (-0.9 to -0.8) | 12.2 (8.2 to 16.8) | 8.5 (5.4 to 12.1) | -1.0 (-1.1 to -0.9) |
| Madagascar | 20.1 (18.6 to 21.8) | 16.9 (15.4 to 18.6) | -0.6 (-0.7 to -0.6) | 5.7 (3.8 to 8.0) | 4.3 (2.9 to 6.1) | -0.9 (-1.0 to -0.9) |
| Malawi | 18.9 (17.5 to 20.7) | 16.4 (14.9 to 18.1) | -0.6 (-0.6 to -0.5) | 5.3 (3.5 to 7.4) | 4.1 (2.8 to 5.9) | -1.0 (-1.1 to -0.8) |
| Malaysia | 22.4 (20.7 to 24.3) | 20.7 (18.8 to 22.6) | -0.4 (-0.4 to -0.3) | 5.1 (3.4 to 7.0) | 3.5 (2.3 to 4.9) | -1.4 (-1.4 to -1.3) |
| Maldives | 26.8 (24.2 to 30.7) | 22.2 (19.7 to 25.0) | -0.6 (-0.7 to -0.5) | 6.7 (4.5 to 9.4) | 3.4 (2.2 to 4.8) | -2.3 (-2.4 to -2.1) |
| Mali | 24.1 (21.8 to 26.6) | 52.1 (30.4 to 97.1) | 2.7 (1.9 to 3.4) | 6.8 (4.6 to 9.6) | 13.2 (6.5 to 26.7) | 2.3 (1.5 to 3.1) |
| Malta | 77.3 (67.7 to 91.3) | 73.2 (62.7 to 88.3) | 0.0 (-0.3 to 0.3) | 10.2 (6.6 to 14.6) | 8.5 (5.4 to 12.1) | -0.3 (-0.5 to 0.0) |
| Marshall Islands | 28.1 (25.2 to 31.1) | 25.4 (22.8 to 28.2) | -0.3 (-0.4 to -0.2) | 7.5 (5.1 to 10.5) | 6.2 (4.1 to 8.4) | -0.6 (-0.7 to -0.6) |
| Mauritania | 22.6 (21.0 to 24.3) | 18.9 (17.5 to 20.6) | -0.6 (-0.7 to -0.6) | 6.3 (4.2 to 8.7) | 4.1 (2.8 to 5.6) | -1.4 (-1.5 to -1.3) |
| Mauritius | 22.6 (21.1 to 24.6) | 19.6 (17.8 to 21.6) | -0.4 (-0.4 to -0.4) | 4.7 (3.2 to 6.6) | 3.3 (2.2 to 4.7) | -1.0 (-1.0 to -0.9) |
| Mexico | 62.1 (56.6 to 69.2) | 41.1 (37.4 to 46.0) | -0.2 (-0.7 to 0.3) | 14.4 (9.9 to 19.8) | 7.3 (5.0 to 10.0) | -1.0 (-1.5 to -0.5) |
| Micronesia (Federated States of) | 29.0 (26.2 to 31.7) | 27.7 (25.0 to 30.6) | -0.1 (-0.2 to -0.1) | 7.8 (5.3 to 10.7) | 6.4 (4.1 to 9.0) | -0.7 (-0.7 to -0.6) |
| Monaco | 65.6 (56.9 to 79.1) | 63.6 (55.8 to 76.7) | 0.0 (-0.2 to 0.1) | 7.6 (4.9 to 10.9) | 7.3 (4.7 to 10.6) | 0.0 (-0.2 to 0.1) |
| Mongolia | 51.7 (47.5 to 56.8) | 51.4 (46.6 to 56.9) | 0.0 (0.0 to 0.1) | 13.7 (9.4 to 18.7) | 10.2 (7.0 to 14.2) | -1.0 (-1.2 to -0.9) |
| Montenegro | 74.2 (67.2 to 83.4) | 63.3 (57.4 to 71.8) | -0.5 (-0.6 to -0.5) | 10.1 (6.8 to 14.2) | 7.3 (4.7 to 10.5) | -1.4 (-1.6 to -1.2) |
| Morocco | 41.8 (37.4 to 47.5) | 33.4 (29.8 to 37.8) | -0.9 (-1.0 to -0.8) | 10.3 (6.8 to 14.0) | 6.3 (4.1 to 8.7) | -1.7 (-1.9 to -1.6) |
| Mozambique | 142.4 (51.7 to 357.1) | 91.8 (39.5 to 215.1) | -1.5 (-1.6 to -1.4) | 42.2 (13.7 to 107.9) | 23.8 (9.1 to 57.2) | -1.9 (-2.0 to -1.8) |
| Myanmar | 53.1 (33.8 to 108.9) | 49.3 (32.0 to 89.5) | -0.6 (-0.7 to -0.5) | 15.3 (8.0 to 33.8) | 11.2 (6.4 to 21.7) | -1.4 (-1.6 to -1.3) |
| Namibia | 73.0 (38.4 to 145.9) | 50.2 (31.2 to 89.3) | -1.3 (-1.4 to -1.1) | 19.7 (8.8 to 41.4) | 10.9 (5.8 to 20.5) | -2.0 (-2.1 to -1.8) |
| Nauru | 30.9 (28.4 to 33.9) | 30.7 (27.7 to 34.1) | -0.1 (-0.2 to 0.1) | 8.0 (5.4 to 11.2) | 7.0 (4.7 to 9.8) | -0.4 (-0.7 to -0.2) |
| Nepal | 32.2 (28.9 to 36.9) | 47.5 (35.0 to 72.4) | 1.9 (1.5 to 2.3) | 8.9 (6.0 to 12.6) | 10.4 (6.4 to 17.5) | 1.1 (0.7 to 1.6) |
| Netherlands | 55.7 (49.6 to 64.4) | 51.4 (45.4 to 60.3) | -0.2 (-0.4 to 0.0) | 6.4 (4.1 to 9.1) | 5.9 (3.9 to 8.4) | -0.2 (-0.4 to 0.0) |
| New Zealand | 122.0 (108.0 to 141.2) | 107.0 (90.9 to 126.1) | -0.5 (-0.8 to -0.3) | 14.8 (9.7 to 21.4) | 12.5 (7.9 to 18.2) | -0.7 (-0.9 to -0.4) |
| Nicaragua | 268.0 (98.4 to 642.6) | 160.3 (68.4 to 366.8) | -1.7 (-1.8 to -1.6) | 64.2 (20.8 to 160.4) | 28.7 (10.8 to 67.5) | -2.7 (-2.8 to -2.6) |
| Niger | 23.7 (21.6 to 26.1) | 25.6 (21.5 to 32.6) | 0.0 (-0.1 to 0.1) | 6.9 (4.6 to 9.9) | 6.8 (4.4 to 9.9) | -0.4 (-0.5 to -0.2) |
| Nigeria | 21.8 (19.9 to 23.8) | 24.5 (20.3 to 31.4) | 0.3 (0.3 to 0.4) | 5.8 (4.1 to 7.8) | 5.6 (3.7 to 8.4) | -0.2 (-0.2 to -0.1) |
| Niue | 26.0 (23.6 to 28.5) | 23.8 (21.2 to 26.5) | -0.4 (-0.5 to -0.3) | 5.9 (4.0 to 8.3) | 4.5 (3.0 to 6.3) | -1.0 (-1.1 to -0.9) |
| North Macedonia | 60.4 (55.6 to 66.4) | 58.3 (52.2 to 66.7) | -0.1 (-0.3 to 0.1) | 11.6 (8.0 to 16.0) | 7.8 (5.2 to 11.2) | -1.5 (-1.6 to -1.4) |
| Northern Mariana Islands | 31.4 (28.7 to 34.2) | 29.7 (26.8 to 32.9) | -0.2 (-0.2 to -0.1) | 6.0 (4.0 to 8.2) | 5.0 (3.4 to 6.9) | -0.4 (-0.5 to -0.3) |
| Norway | 81.1 (73.5 to 92.7) | 60.0 (53.7 to 70.3) | -0.9 (-1.1 to -0.8) | 9.8 (6.5 to 13.9) | 6.9 (4.6 to 9.9) | -1.0 (-1.1 to -0.8) |
| Oman | 53.6 (49.0 to 59.5) | 38.8 (34.9 to 43.3) | -1.2 (-1.3 to -1.1) | 11.1 (7.6 to 15.0) | 5.1 (3.4 to 7.2) | -2.5 (-2.7 to -2.4) |
| Pakistan | 22.0 (20.2 to 24.1) | 32.6 (23.5 to 50.6) | 1.6 (1.4 to 1.8) | 5.9 (4.0 to 8.3) | 7.8 (4.7 to 13.7) | 1.2 (1.0 to 1.4) |
| Palau | 34.7 (31.0 to 38.1) | 34.4 (30.7 to 38.5) | 0.0 (-0.1 to 0.0) | 7.6 (5.2 to 10.3) | 6.5 (4.4 to 9.0) | -0.5 (-0.5 to -0.4) |
| Palestine | 216.2 (96.5 to 443.8) | 267.7 (110.2 to 601.6) | 1.4 (1.0 to 1.7) | 47.9 (18.6 to 106.2) | 43.0 (16.2 to 102.7) | 0.4 (0.0 to 0.8) |
| Panama | 50.5 (41.2 to 75.6) | 42.3 (35.5 to 57.5) | -0.6 (-0.7 to -0.5) | 10.5 (6.8 to 16.7) | 6.6 (4.4 to 9.9) | -1.5 (-1.5 to -1.4) |
| Papua New Guinea | 25.7 (23.4 to 28.0) | 30.4 (27.5 to 34.0) | 0.5 (0.4 to 0.5) | 6.8 (4.5 to 9.7) | 7.5 (5.1 to 10.3) | 0.3 (0.3 to 0.4) |
| Paraguay | 37.6 (32.7 to 43.5) | 34.4 (30.2 to 39.7) | -0.3 (-0.4 to -0.2) | 8.4 (5.7 to 11.8) | 6.2 (4.3 to 8.7) | -1.0 (-1.1 to -0.9) |
| Peru | 50.2 (34.6 to 82.4) | 43.4 (31.0 to 67.1) | -0.7 (-0.9 to -0.6) | 12.5 (7.2 to 22.2) | 6.9 (4.0 to 11.7) | -2.3 (-2.4 to -2.1) |
| Philippines | 50.4 (35.3 to 87.3) | 39.9 (27.5 to 66.7) | -0.9 (-0.9 to -0.8) | 12.0 (7.0 to 21.4) | 8.6 (5.1 to 15.1) | -1.1 (-1.2 to -1.1) |
| Poland | 78.7 (72.1 to 86.1) | 58.3 (53.2 to 64.4) | -1.0 (-1.1 to -1.0) | 14.4 (9.8 to 19.8) | 6.8 (4.5 to 9.8) | -2.5 (-2.6 to -2.5) |
| Portugal | 92.5 (80.5 to 105.5) | 53.4 (47.4 to 62.8) | -2.0 (-2.1 to -1.9) | 14.2 (9.7 to 19.8) | 6.1 (4.0 to 9.0) | -2.8 (-3.0 to -2.6) |
| Puerto Rico | 34.8 (32.0 to 38.1) | 34.8 (31.5 to 39.1) | -0.1 (-0.2 to 0.0) | 6.1 (4.2 to 8.6) | 4.2 (2.7 to 6.1) | -1.5 (-1.6 to -1.4) |
| Qatar | 42.4 (38.8 to 47.0) | 35.3 (31.5 to 40.7) | -0.6 (-0.8 to -0.4) | 8.0 (5.5 to 11.3) | 4.0 (2.6 to 5.9) | -2.4 (-2.7 to -2.2) |
| Republic of Korea | 96.1 (87.5 to 109.5) | 59.4 (53.1 to 69.4) | -1.9 (-1.9 to -1.8) | 17.7 (11.9 to 24.6) | 6.9 (4.5 to 10.1) | -3.2 (-3.5 to -3.0) |
| Republic of Moldova | 76.6 (70.0 to 84.2) | 51.2 (46.9 to 56.5) | -1.5 (-1.6 to -1.4) | 14.8 (9.9 to 20.0) | 7.5 (5.1 to 10.7) | -2.4 (-2.5 to -2.2) |
| Romania | 93.8 (85.0 to 105.5) | 67.5 (61.2 to 76.6) | -1.2 (-1.3 to -1.2) | 18.4 (12.5 to 25.5) | 8.6 (5.6 to 12.1) | -2.7 (-2.8 to -2.6) |
| Russian Federation | 91.8 (84.9 to 100.7) | 72.7 (65.6 to 81.1) | -1.0 (-1.3 to -0.7) | 16.2 (11.2 to 22.0) | 9.2 (6.1 to 12.8) | -2.3 (-2.7 to -1.8) |
| Rwanda | 26.6 (23.8 to 30.6) | 312.3 (92.1 to 790.8) | 4.6 (1.6 to 7.7) | 8.1 (5.4 to 11.3) | 76.5 (20.0 to 197.6) | 3.7 (0.6 to 6.8) |
| Saint Kitts and Nevis | 33.4 (30.9 to 36.4) | 35.1 (31.9 to 39.2) | 0.4 (0.2 to 0.5) | 8.3 (5.6 to 11.4) | 6.4 (4.3 to 8.7) | -0.6 (-0.7 to -0.5) |
| Saint Lucia | 29.4 (27.3 to 32.0) | 29.9 (27.4 to 33.2) | 0.0 (-0.1 to 0.2) | 6.7 (4.5 to 9.2) | 5.4 (3.6 to 7.4) | -0.7 (-0.8 to -0.7) |
| Saint Vincent and the Grenadines | 29.4 (27.2 to 32.0) | 30.4 (28.1 to 33.1) | 0.1 (-0.1 to 0.2) | 6.7 (4.5 to 9.2) | 5.9 (3.9 to 8.1) | -0.4 (-0.5 to -0.3) |
| Samoa | 25.4 (23.0 to 28.0) | 24.4 (22.0 to 27.1) | -0.2 (-0.2 to -0.1) | 6.1 (4.1 to 8.3) | 5.0 (3.4 to 6.8) | -0.6 (-0.7 to -0.6) |
| San Marino | 67.8 (59.4 to 80.3) | 64.9 (55.8 to 78.4) | -0.1 (-0.3 to 0.0) | 7.8 (5.0 to 11.2) | 7.5 (4.9 to 10.7) | -0.1 (-0.3 to 0.0) |
| Sao Tome and Principe | 21.8 (19.7 to 25.0) | 22.4 (19.9 to 25.4) | 0.1 (0.0 to 0.1) | 5.7 (3.8 to 8.1) | 4.8 (3.2 to 6.6) | -0.6 (-0.6 to -0.6) |
| Saudi Arabia | 58.9 (53.3 to 66.0) | 64.7 (57.7 to 74.1) | 0.5 (0.4 to 0.5) | 13.4 (8.9 to 18.8) | 9.0 (6.2 to 12.7) | -1.2 (-1.3 to -1.2) |
| Senegal | 18.2 (16.8 to 20.1) | 19.4 (16.7 to 23.6) | 0.1 (0.0 to 0.3) | 5.0 (3.3 to 6.8) | 4.6 (3.0 to 6.5) | -0.3 (-0.4 to -0.2) |
| Serbia | 71.3 (65.4 to 79.1) | 78.6 (61.8 to 109.3) | 0.3 (0.0 to 0.6) | 13.0 (8.7 to 17.9) | 9.3 (5.5 to 15.1) | -1.3 (-1.6 to -0.9) |
| Seychelles | 27.1 (23.8 to 32.1) | 22.6 (20.2 to 25.6) | -0.6 (-0.6 to -0.6) | 6.1 (4.0 to 8.8) | 4.0 (2.6 to 5.6) | -1.4 (-1.4 to -1.3) |
| Sierra Leone | 19.8 (18.3 to 21.8) | 69.5 (33.7 to 144.0) | 3.2 (1.9 to 4.5) | 5.5 (3.8 to 7.7) | 17.4 (7.4 to 38.8) | 2.9 (1.5 to 4.2) |
| Singapore | 63.7 (57.8 to 72.0) | 54.4 (48.2 to 64.0) | -0.6 (-0.7 to -0.5) | 9.9 (6.9 to 13.7) | 6.3 (4.0 to 9.1) | -1.4 (-1.5 to -1.3) |
| Slovakia | 83.5 (75.6 to 93.3) | 68.3 (61.6 to 77.4) | -0.7 (-0.7 to -0.7) | 13.9 (9.2 to 19.4) | 7.9 (5.1 to 10.9) | -1.9 (-2.0 to -1.9) |
| Slovenia | 98.9 (90.6 to 110.9) | 76.5 (68.8 to 87.1) | -0.6 (-0.8 to -0.3) | 13.6 (8.9 to 19.4) | 8.8 (5.7 to 13.0) | -1.1 (-1.3 to -0.9) |
| Solomon Islands | 32.3 (29.4 to 35.1) | 33.0 (29.7 to 36.9) | 0.0 (0.0 to 0.1) | 9.0 (5.9 to 12.4) | 8.2 (5.5 to 11.5) | -0.3 (-0.4 to -0.2) |
| Somalia | 48.6 (25.3 to 156.1) | 85.1 (41.5 to 185.8) | 1.1 (0.9 to 1.3) | 14.9 (6.4 to 53.0) | 24.9 (10.6 to 56.8) | 0.9 (0.7 to 1.2) |
| South Africa | 51.0 (44.5 to 64.5) | 33.2 (29.1 to 42.1) | -1.6 (-1.7 to -1.4) | 11.9 (8.1 to 16.8) | 6.7 (4.5 to 9.4) | -2.1 (-2.3 to -1.9) |
| South Sudan | 47.4 (28.0 to 87.1) | 90.9 (41.3 to 193.9) | 2.4 (2.0 to 2.8) | 13.5 (6.7 to 26.6) | 23.2 (9.6 to 51.4) | 2.1 (1.7 to 2.5) |
| Spain | 72.1 (63.9 to 80.8) | 64.5 (55.6 to 76.4) | -0.3 (-0.5 to -0.2) | 8.8 (5.9 to 12.2) | 7.5 (4.7 to 10.9) | -0.4 (-0.5 to -0.3) |
| Sri Lanka | 66.0 (43.0 to 116.2) | 135.4 (60.4 to 294.1) | 2.0 (1.6 to 2.4) | 14.8 (8.1 to 28.4) | 20.8 (8.1 to 49.9) | 0.6 (0.2 to 1.1) |
| Sudan | 49.9 (38.0 to 73.8) | 74.3 (44.0 to 138.8) | 1.4 (1.1 to 1.7) | 13.3 (8.1 to 21.7) | 15.4 (7.8 to 30.5) | 0.6 (0.3 to 0.9) |
| Suriname | 44.0 (29.8 to 100.2) | 37.1 (28.3 to 70.6) | -0.5 (-0.6 to -0.4) | 10.9 (5.9 to 25.8) | 7.8 (4.8 to 15.0) | -1.1 (-1.1 to -1.0) |
| Sweden | 74.7 (66.3 to 87.0) | 58.8 (51.5 to 69.0) | -0.7 (-0.9 to -0.6) | 8.6 (5.4 to 12.2) | 6.8 (4.4 to 9.6) | -0.7 (-0.9 to -0.6) |
| Switzerland | 106.5 (92.7 to 123.6) | 79.4 (67.6 to 95.0) | -1.3 (-1.6 to -1.1) | 12.2 (7.9 to 17.3) | 9.1 (5.8 to 13.3) | -1.3 (-1.6 to -1.0) |
| Syrian Arab Republic | 50.0 (32.3 to 97.9) | 375.1 (127.3 to 918.3) | 6.9 (5.0 to 8.9) | 11.3 (6.2 to 23.8) | 59.1 (17.8 to 151.0) | 5.6 (3.6 to 7.7) |
| Taiwan (Province of China) | 32.2 (29.0 to 35.7) | 20.2 (18.6 to 21.7) | -1.9 (-2.0 to -1.7) | 5.1 (3.4 to 7.1) | 2.3 (1.6 to 3.5) | -2.9 (-3.1 to -2.8) |
| Tajikistan | 45.7 (41.7 to 50.9) | 81.3 (46.5 to 151.7) | 0.4 (-0.5 to 1.3) | 10.8 (7.4 to 15.3) | 17.6 (9.0 to 35.6) | 0.1 (-0.9 to 1.0) |
| Thailand | 37.7 (34.3 to 41.1) | 30.7 (27.1 to 35.6) | -0.9 (-1.0 to -0.8) | 8.0 (5.5 to 10.9) | 4.5 (3.0 to 6.6) | -2.2 (-2.3 to -2.1) |
| Timor-Leste | 209.0 (77.1 to 475.2) | 241.7 (84.8 to 557.9) | 0.3 (-0.2 to 0.9) | 59.7 (19.2 to 142.2) | 56.7 (17.8 to 138.7) | -0.4 (-0.9 to 0.2) |
| Togo | 21.6 (19.9 to 23.6) | 21.3 (19.3 to 23.6) | -0.1 (-0.1 to 0.0) | 5.9 (3.9 to 8.1) | 5.1 (3.5 to 7.1) | -0.4 (-0.5 to -0.3) |
| Tokelau | 24.5 (22.3 to 26.9) | 22.0 (19.5 to 24.5) | -0.4 (-0.5 to -0.3) | 6.1 (4.1 to 8.4) | 4.3 (2.9 to 6.0) | -1.1 (-1.2 to -1.0) |
| Tonga | 21.4 (19.6 to 23.4) | 17.5 (15.9 to 19.2) | -0.7 (-0.7 to -0.6) | 4.9 (3.2 to 6.9) | 3.5 (2.3 to 4.9) | -1.0 (-1.1 to -1.0) |
| Trinidad and Tobago | 29.9 (27.5 to 33.0) | 39.4 (32.6 to 50.9) | 0.9 (0.8 to 1.0) | 6.7 (4.5 to 9.3) | 7.1 (4.8 to 10.6) | 0.0 (-0.1 to 0.1) |
| Tunisia | 39.9 (35.7 to 47.4) | 34.1 (30.5 to 39.4) | -0.5 (-0.5 to -0.5) | 8.1 (5.4 to 11.3) | 4.8 (3.2 to 7.1) | -1.7 (-1.8 to -1.7) |
| Turkey | 32.0 (29.2 to 35.4) | 32.5 (27.2 to 41.9) | -0.1 (-0.2 to 0.0) | 7.4 (5.2 to 10.2) | 4.5 (2.9 to 6.7) | -1.8 (-2.0 to -1.7) |
| Turkmenistan | 44.4 (41.1 to 49.3) | 37.9 (33.7 to 43.4) | -0.7 (-0.8 to -0.7) | 10.2 (7.0 to 14.3) | 7.3 (4.9 to 10.3) | -1.4 (-1.5 to -1.3) |
| Tuvalu | 28.8 (26.4 to 31.5) | 25.8 (23.3 to 28.7) | -1.2 (-1.5 to -0.8) | 7.9 (5.2 to 11.3) | 5.8 (3.8 to 8.1) | -1.8 (-2.1 to -1.5) |
| Uganda | 112.3 (43.7 to 259.5) | 76.1 (34.6 to 166.8) | -1.3 (-1.4 to -1.1) | 31.4 (10.7 to 75.9) | 18.4 (7.2 to 42.5) | -1.8 (-1.9 to -1.6) |
| Ukraine | 84.2 (76.8 to 95.5) | 74.5 (66.2 to 84.0) | -0.8 (-1.0 to -0.6) | 14.1 (9.4 to 19.2) | 11.0 (7.4 to 15.3) | -1.4 (-1.6 to -1.1) |
| United Arab Emirates | 42.2 (38.5 to 46.4) | 38.5 (34.7 to 43.1) | -0.4 (-0.4 to -0.3) | 9.2 (6.2 to 12.7) | 6.1 (4.1 to 8.6) | -1.4 (-1.4 to -1.3) |
| United Kingdom | 65.7 (59.3 to 76.1) | 54.8 (48.7 to 63.9) | -0.6 (-0.8 to -0.4) | 8.3 (5.6 to 11.7) | 6.3 (4.1 to 8.9) | -0.8 (-1.0 to -0.7) |
| United Republic of Tanzania | 21.3 (19.6 to 23.3) | 19.4 (17.7 to 21.4) | -0.3 (-0.4 to -0.3) | 5.8 (3.8 to 8.0) | 4.7 (3.0 to 6.7) | -0.6 (-0.7 to -0.6) |
| United States of America | 68.0 (62.3 to 75.4) | 49.7 (45.3 to 54.6) | -1.2 (-1.5 to -1.0) | 8.1 (5.4 to 11.2) | 5.6 (3.7 to 7.7) | -1.3 (-1.6 to -1.1) |
| United States Virgin Islands | 34.6 (31.6 to 37.7) | 28.8 (26.4 to 31.4) | -0.9 (-1.1 to -0.7) | 7.0 (4.7 to 9.5) | 4.7 (3.1 to 6.6) | -1.6 (-1.7 to -1.4) |
| Uruguay | 74.9 (67.7 to 84.5) | 68.8 (62.0 to 79.2) | -0.4 (-0.4 to -0.3) | 14.0 (9.4 to 19.2) | 9.7 (6.5 to 13.6) | -1.3 (-1.3 to -1.2) |
| Uzbekistan | 44.2 (40.3 to 48.9) | 36.5 (33.2 to 41.1) | -0.7 (-0.8 to -0.6) | 9.7 (6.5 to 13.2) | 7.1 (4.9 to 9.7) | -1.3 (-1.4 to -1.1) |
| Vanuatu | 23.3 (21.4 to 25.7) | 22.0 (20.1 to 24.0) | -0.2 (-0.3 to -0.1) | 6.1 (4.0 to 8.6) | 5.4 (3.6 to 7.5) | -0.4 (-0.5 to -0.3) |
| Venezuela (Bolivarian Republic of) | 53.8 (49.2 to 59.9) | 54.1 (48.2 to 61.1) | 0.0 (-0.1 to 0.1) | 12.2 (8.4 to 16.7) | 9.7 (6.6 to 13.4) | -0.8 (-0.8 to -0.7) |
| Viet Nam | 25.8 (23.9 to 27.6) | 25.3 (22.8 to 27.4) | 0.1 (0.0 to 0.2) | 6.1 (4.1 to 8.4) | 4.2 (2.8 to 5.9) | -1.1 (-1.2 to -1.0) |
| Yemen | 53.8 (40.7 to 80.0) | 109.0 (55.6 to 225.2) | 1.4 (0.8 to 2.1) | 14.2 (8.5 to 23.6) | 24.7 (11.0 to 52.2) | 0.8 (0.1 to 1.5) |
| Zambia | 21.4 (19.8 to 23.3) | 19.9 (18.0 to 21.8) | -0.3 (-0.4 to -0.2) | 6.1 (4.2 to 8.5) | 4.8 (3.2 to 6.8) | -0.9 (-1.0 to -0.7) |
| Zimbabwe | 22.9 (21.3 to 24.8) | 22.8 (20.9 to 24.8) | 0.0 (-0.1 to 0.1) | 5.7 (3.7 to 8.0) | 5.8 (3.9 to 7.9) | 0.3 (0.1 to 0.4) |

**Table S15. ASR of prevalence and YLDs in 1990 and 2021, and their EAPC from 1990 to 2021 for AMP-Th, by country.**

| **Countries** | **Prevalence** | | | **YLDs** | | |
| --- | --- | --- | --- | --- | --- | --- |
|  | **Age-standardized prevalence rate, 1990** | **Age-standardized prevalence rate, 2021** | **EAPC of prevalence rate No. (95% CI)** | **Age-standardized YLDs rate, 1990** | **Age-standardized YLDs rate, 2021** | **EAPC of YLDs rate No. (95% CI)** |
| Afghanistan | 3651.7 (1864.2 to 7680.4) | 3612.4 (2043.3 to 7087.2) | -0.2 (-0.3 to -0.1) | 40.2 (15.3 to 97.6) | 39.3 (16.0 to 90.9) | -0.2 (-0.3 to -0.2) |
| Albania | 5601.5 (5026.2 to 6271.0) | 4288.7 (3841.9 to 4799.5) | -1.3 (-1.5 to -1.1) | 61.7 (29.3 to 114.4) | 47.3 (22.2 to 89.0) | -1.3 (-1.4 to -1.1) |
| Algeria | 1521.0 (1405.5 to 1662.6) | 1143.8 (1021.8 to 1294.7) | -0.9 (-1.0 to -0.9) | 16.7 (8.0 to 30.9) | 12.6 (6.0 to 23.6) | -0.9 (-1.0 to -0.9) |
| American Samoa | 831.9 (760.0 to 923.9) | 748.0 (658.7 to 871.4) | -0.2 (-0.3 to -0.1) | 9.1 (4.4 to 17.1) | 8.2 (3.9 to 15.6) | -0.2 (-0.3 to -0.1) |
| Andorra | 2399.9 (2121.9 to 2763.2) | 2243.3 (1969.7 to 2590.6) | -0.2 (-0.3 to -0.2) | 26.5 (12.3 to 50.3) | 24.7 (11.4 to 47.2) | -0.2 (-0.3 to -0.2) |
| Angola | 1440.6 (1063.6 to 2192.3) | 1161.1 (814.8 to 1913.5) | -0.8 (-1.1 to -0.6) | 15.9 (7.3 to 32.5) | 12.7 (5.8 to 27.3) | -0.8 (-1.1 to -0.6) |
| Antigua and Barbuda | 2008.5 (1821.3 to 2227.4) | 2209.8 (1984.2 to 2482.3) | 0.2 (0.0 to 0.4) | 22.1 (10.5 to 41.4) | 24.3 (11.5 to 45.6) | 0.2 (0.0 to 0.4) |
| Argentina | 3919.0 (3478.4 to 4419.4) | 3411.0 (3041.9 to 3831.1) | -0.4 (-0.7 to -0.1) | 43.2 (20.4 to 83.0) | 37.6 (17.7 to 70.3) | -0.4 (-0.7 to -0.1) |
| Armenia | 3275.6 (2906.0 to 3807.1) | 2053.6 (1791.2 to 2405.4) | -1.7 (-1.9 to -1.6) | 36.1 (16.9 to 67.7) | 22.7 (11.1 to 42.2) | -1.7 (-1.8 to -1.5) |
| Australia | 5423.2 (4788.1 to 6181.8) | 4424.9 (3899.9 to 5064.6) | -0.6 (-0.7 to -0.5) | 59.7 (27.7 to 112.4) | 48.7 (22.7 to 91.3) | -0.6 (-0.7 to -0.5) |
| Austria | 3128.1 (2804.1 to 3514.7) | 2248.5 (2000.1 to 2585.3) | -1.1 (-1.1 to -1.0) | 34.5 (16.0 to 64.8) | 24.8 (11.5 to 47.2) | -1.1 (-1.1 to -1.0) |
| Azerbaijan | 1980.1 (1814.5 to 2186.5) | 1812.2 (1628.4 to 2061.2) | -0.4 (-0.5 to -0.3) | 21.9 (10.2 to 41.2) | 20.1 (9.6 to 37.5) | -0.4 (-0.5 to -0.3) |
| Bahamas | 1147.2 (1054.2 to 1271.8) | 1213.0 (1099.9 to 1356.0) | 0.2 (0.1 to 0.3) | 12.7 (6.0 to 24.0) | 13.3 (6.4 to 24.8) | 0.2 (0.1 to 0.3) |
| Bahrain | 1133.1 (1035.0 to 1257.2) | 1066.1 (961.4 to 1198.7) | -0.3 (-0.4 to -0.2) | 12.5 (5.9 to 23.4) | 11.7 (5.5 to 22.1) | -0.3 (-0.4 to -0.2) |
| Bangladesh | 633.9 (574.8 to 706.0) | 665.8 (568.5 to 798.5) | -0.1 (-0.3 to 0.0) | 6.9 (3.3 to 12.9) | 7.3 (3.5 to 13.7) | -0.1 (-0.3 to 0.1) |
| Barbados | 1204.2 (1093.1 to 1342.4) | 1221.0 (1105.2 to 1352.7) | -0.1 (-0.3 to 0.1) | 13.3 (6.2 to 24.8) | 13.5 (6.3 to 25.4) | -0.1 (-0.3 to 0.1) |
| Belarus | 2950.6 (2662.5 to 3264.4) | 2536.7 (2272.7 to 2841.0) | -0.6 (-0.8 to -0.3) | 32.5 (15.3 to 60.0) | 27.9 (13.2 to 52.0) | -0.6 (-0.8 to -0.3) |
| Belgium | 2611.6 (2331.4 to 2996.5) | 2260.9 (1996.4 to 2617.5) | -0.2 (-0.8 to 0.4) | 28.8 (13.5 to 54.9) | 24.9 (11.5 to 47.1) | -0.2 (-0.8 to 0.4) |
| Belize | 1553.9 (1422.8 to 1712.6) | 1730.3 (1584.2 to 1912.3) | 0.2 (-0.1 to 0.4) | 17.2 (8.1 to 32.2) | 19.0 (9.2 to 35.8) | 0.2 (-0.1 to 0.4) |
| Benin | 816.5 (744.8 to 883.8) | 725.1 (663.0 to 792.9) | -0.4 (-0.4 to -0.3) | 8.9 (4.3 to 16.6) | 8.0 (3.8 to 14.8) | -0.4 (-0.4 to -0.3) |
| Bermuda | 1289.0 (1178.1 to 1428.6) | 1295.0 (1171.1 to 1442.5) | 0.1 (-0.1 to 0.2) | 14.3 (6.8 to 26.4) | 14.3 (6.7 to 26.7) | 0.1 (-0.1 to 0.2) |
| Bhutan | 962.6 (881.6 to 1054.5) | 996.3 (902.2 to 1105.9) | 0.1 (0.0 to 0.2) | 10.6 (5.0 to 19.8) | 10.9 (5.3 to 20.2) | 0.1 (0.0 to 0.2) |
| Bolivia (Plurinational State of) | 1500.8 (1381.5 to 1648.0) | 1251.3 (1146.9 to 1372.7) | -0.6 (-0.7 to -0.6) | 16.5 (7.9 to 31.0) | 13.7 (6.5 to 25.7) | -0.6 (-0.7 to -0.6) |
| Bosnia and Herzegovina | 5220.0 (4659.7 to 5883.5) | 3729.9 (3233.8 to 4388.3) | -1.3 (-1.6 to -1.1) | 57.4 (27.5 to 107.9) | 41.0 (19.7 to 78.1) | -1.3 (-1.6 to -1.1) |
| Botswana | 819.6 (757.9 to 883.6) | 808.3 (744.3 to 871.9) | -0.2 (-0.4 to -0.1) | 9.0 (4.2 to 16.5) | 8.8 (4.2 to 16.3) | -0.2 (-0.4 to -0.1) |
| Brazil | 1820.7 (1612.7 to 2085.5) | 1413.9 (1255.8 to 1606.1) | -1.1 (-1.3 to -0.9) | 19.9 (9.4 to 37.5) | 15.5 (7.2 to 28.7) | -1.1 (-1.3 to -0.9) |
| Brunei Darussalam | 2671.4 (2439.1 to 2952.9) | 2212.1 (2003.8 to 2459.3) | -0.6 (-0.7 to -0.6) | 29.4 (13.9 to 54.4) | 24.3 (11.4 to 44.2) | -0.6 (-0.7 to -0.6) |
| Bulgaria | 4589.9 (4155.5 to 5083.5) | 3925.0 (3549.3 to 4376.3) | -0.4 (-0.5 to -0.3) | 50.5 (24.1 to 94.8) | 43.2 (20.2 to 80.7) | -0.4 (-0.5 to -0.3) |
| Burkina Faso | 750.4 (682.1 to 817.8) | 724.4 (657.5 to 798.3) | -0.1 (-0.2 to -0.1) | 8.2 (3.9 to 14.9) | 8.0 (3.8 to 14.9) | -0.1 (-0.2 to -0.1) |
| Burundi | 817.0 (752.3 to 888.9) | 2029.1 (1216.8 to 3739.2) | 3.3 (2.3 to 4.2) | 9.0 (4.2 to 16.9) | 22.4 (9.4 to 51.3) | 3.3 (2.3 to 4.2) |
| Cabo Verde | 867.2 (791.5 to 938.4) | 756.2 (688.0 to 832.7) | -0.4 (-0.5 to -0.4) | 9.6 (4.6 to 17.8) | 8.3 (4.0 to 15.9) | -0.4 (-0.5 to -0.4) |
| Cambodia | 1690.5 (1113.1 to 2935.5) | 1249.8 (904.9 to 2022.9) | -0.8 (-1.0 to -0.7) | 18.7 (8.3 to 41.0) | 13.7 (6.4 to 27.7) | -0.9 (-1.0 to -0.7) |
| Cameroon | 716.8 (652.4 to 783.0) | 700.4 (628.6 to 794.1) | -0.1 (-0.2 to 0.0) | 7.8 (3.8 to 14.6) | 7.7 (3.7 to 14.5) | -0.1 (-0.1 to 0.0) |
| Canada | 1797.1 (1650.7 to 1991.0) | 1345.5 (1234.1 to 1494.0) | -1.0 (-1.0 to -0.9) | 19.8 (9.3 to 37.2) | 14.8 (7.0 to 27.1) | -1.0 (-1.0 to -0.9) |
| Central African Republic | 780.9 (719.4 to 842.6) | 949.0 (785.6 to 1259.0) | 0.7 (0.5 to 0.8) | 8.5 (4.1 to 15.7) | 10.4 (5.1 to 20.1) | 0.7 (0.6 to 0.9) |
| Chad | 1042.5 (848.1 to 1412.8) | 1034.0 (847.7 to 1379.5) | 0.0 (-0.1 to 0.1) | 11.5 (5.6 to 22.6) | 11.3 (5.6 to 21.7) | 0.0 (-0.1 to 0.1) |
| Chile | 3284.3 (2977.3 to 3643.1) | 3726.8 (3322.6 to 4179.9) | 0.6 (0.4 to 0.8) | 36.1 (17.0 to 66.9) | 41.0 (19.1 to 76.4) | 0.6 (0.4 to 0.8) |
| China | 799.4 (712.5 to 923.3) | 735.2 (643.6 to 846.2) | -0.6 (-0.9 to -0.3) | 8.8 (4.1 to 16.3) | 8.1 (3.8 to 15.1) | -0.6 (-0.9 to -0.3) |
| Colombia | 2520.4 (2274.8 to 2815.1) | 1709.2 (1499.1 to 1989.8) | -1.1 (-1.2 to -1.0) | 27.7 (13.2 to 51.0) | 18.9 (9.2 to 35.1) | -1.1 (-1.2 to -1.0) |
| Comoros | 831.2 (769.4 to 898.4) | 738.5 (673.4 to 825.2) | -0.5 (-0.6 to -0.4) | 9.1 (4.4 to 17.2) | 8.1 (4.0 to 15.1) | -0.5 (-0.6 to -0.4) |
| Congo | 730.0 (674.7 to 787.8) | 836.3 (655.7 to 1175.6) | 0.5 (0.1 to 1.0) | 8.0 (3.8 to 14.8) | 9.2 (4.5 to 18.7) | 0.5 (0.1 to 1.0) |
| Cook Islands | 656.7 (594.0 to 738.0) | 660.8 (571.3 to 783.0) | 0.0 (-0.2 to 0.2) | 7.2 (3.4 to 13.3) | 7.2 (3.5 to 13.5) | 0.0 (-0.2 to 0.2) |
| Costa Rica | 1705.4 (1498.5 to 1955.4) | 1522.0 (1336.2 to 1749.2) | -0.4 (-0.5 to -0.3) | 18.8 (8.7 to 34.9) | 16.8 (7.8 to 31.5) | -0.4 (-0.5 to -0.3) |
| Coted'Ivoire | 802.1 (733.1 to 872.3) | 743.2 (676.8 to 830.5) | -0.3 (-0.3 to -0.2) | 8.7 (4.1 to 16.2) | 8.2 (3.9 to 15.1) | -0.2 (-0.3 to -0.2) |
| Croatia | 4126.9 (3723.0 to 4584.8) | 3107.3 (2768.6 to 3521.9) | -0.9 (-0.9 to -0.8) | 45.5 (21.4 to 86.0) | 34.2 (16.3 to 64.4) | -0.9 (-0.9 to -0.8) |
| Cuba | 1477.9 (1350.0 to 1631.5) | 1553.2 (1412.0 to 1717.2) | 0.3 (0.1 to 0.5) | 16.3 (7.7 to 30.4) | 17.1 (7.9 to 31.7) | 0.3 (0.1 to 0.5) |
| Cyprus | 2651.0 (2380.5 to 2960.1) | 2184.8 (1945.0 to 2506.5) | -0.7 (-0.8 to -0.5) | 29.2 (13.6 to 54.9) | 24.1 (11.3 to 45.7) | -0.6 (-0.8 to -0.5) |
| Czechia | 4967.4 (4523.6 to 5529.4) | 3622.9 (3272.6 to 4039.4) | -0.6 (-0.9 to -0.3) | 54.6 (25.9 to 102.4) | 39.8 (18.6 to 75.0) | -0.6 (-0.9 to -0.3) |
| Democratic People's Republic of Korea | 528.8 (486.9 to 591.3) | 430.5 (401.2 to 471.2) | -0.6 (-0.7 to -0.5) | 5.9 (2.8 to 10.7) | 4.8 (2.3 to 8.8) | -0.6 (-0.7 to -0.5) |
| Democratic Republic of the Congo | 755.0 (691.3 to 819.2) | 779.5 (651.6 to 996.4) | 0.1 (-0.1 to 0.4) | 8.2 (3.9 to 15.2) | 8.6 (4.2 to 16.4) | 0.2 (-0.1 to 0.4) |
| Denmark | 2284.0 (2038.4 to 2571.2) | 2010.1 (1762.8 to 2353.8) | -0.4 (-0.5 to -0.3) | 25.2 (11.7 to 47.6) | 22.2 (10.3 to 42.1) | -0.4 (-0.5 to -0.3) |
| Djibouti | 825.4 (763.0 to 898.8) | 801.6 (722.8 to 902.7) | -0.3 (-0.4 to -0.1) | 9.1 (4.3 to 16.8) | 8.8 (4.3 to 16.7) | -0.3 (-0.4 to -0.1) |
| Dominica | 1122.9 (1027.0 to 1246.4) | 1245.0 (1129.4 to 1398.3) | 0.3 (0.2 to 0.5) | 12.4 (5.9 to 23.4) | 13.7 (6.6 to 25.4) | 0.3 (0.2 to 0.5) |
| Dominican Republic | 1200.9 (1103.3 to 1314.0) | 1396.4 (1283.2 to 1535.8) | 0.6 (0.4 to 0.8) | 13.3 (6.3 to 24.8) | 15.3 (7.2 to 28.6) | 0.6 (0.4 to 0.8) |
| Ecuador | 1535.3 (1402.3 to 1691.9) | 1360.9 (1248.3 to 1518.5) | -0.4 (-0.5 to -0.3) | 16.9 (8.2 to 31.1) | 15.0 (7.2 to 27.8) | -0.4 (-0.5 to -0.3) |
| Egypt | 1257.4 (1148.9 to 1374.2) | 942.8 (848.0 to 1053.3) | -0.9 (-0.9 to -0.8) | 13.8 (6.6 to 25.8) | 10.3 (4.9 to 19.7) | -0.9 (-0.9 to -0.8) |
| El Salvador | 2853.5 (2106.7 to 4322.7) | 2073.6 (1623.6 to 2895.6) | -1.1 (-1.2 to -1.0) | 31.5 (15.1 to 64.7) | 22.7 (11.0 to 44.5) | -1.1 (-1.2 to -1.1) |
| Equatorial Guinea | 808.6 (745.2 to 870.3) | 561.9 (512.8 to 619.2) | -1.2 (-1.3 to -1.2) | 8.8 (4.2 to 16.4) | 6.1 (2.9 to 11.7) | -1.2 (-1.2 to -1.2) |
| Eritrea | 2931.4 (1610.5 to 5577.3) | 2152.2 (1275.6 to 3979.2) | -1.2 (-1.3 to -1.0) | 32.4 (12.9 to 80.2) | 23.5 (9.8 to 55.2) | -1.2 (-1.4 to -1.1) |
| Estonia | 3556.5 (3216.6 to 3939.9) | 2341.0 (2110.5 to 2612.3) | -1.6 (-1.7 to -1.5) | 39.1 (18.5 to 74.0) | 25.8 (12.1 to 48.3) | -1.6 (-1.7 to -1.5) |
| Eswatini | 854.3 (789.5 to 919.1) | 791.7 (735.6 to 854.0) | -0.2 (-0.3 to -0.1) | 9.4 (4.4 to 17.5) | 8.6 (4.1 to 16.0) | -0.3 (-0.4 to -0.2) |
| Ethiopia | 1184.8 (1059.3 to 1374.3) | 908.7 (748.3 to 1239.1) | -1.0 (-1.2 to -0.9) | 12.9 (6.3 to 24.7) | 10.0 (4.9 to 19.4) | -1.0 (-1.2 to -0.9) |
| Fiji | 636.5 (584.1 to 706.8) | 553.2 (495.1 to 627.7) | -0.5 (-0.6 to -0.5) | 7.0 (3.4 to 12.8) | 6.1 (3.0 to 11.4) | -0.5 (-0.6 to -0.5) |
| Finland | 3017.3 (2651.9 to 3487.0) | 2608.3 (2268.2 to 3066.1) | -0.6 (-1.1 to 0.0) | 33.2 (15.3 to 63.7) | 28.7 (13.2 to 54.5) | -0.6 (-1.1 to 0.0) |
| France | 2685.4 (2402.9 to 3021.0) | 2223.3 (1965.4 to 2554.7) | -0.5 (-0.6 to -0.4) | 29.6 (13.8 to 56.4) | 24.5 (11.4 to 46.5) | -0.5 (-0.6 to -0.4) |
| Gabon | 789.5 (726.1 to 860.1) | 635.6 (579.7 to 700.8) | -0.7 (-0.7 to -0.6) | 8.6 (4.1 to 16.0) | 7.0 (3.4 to 13.0) | -0.7 (-0.7 to -0.6) |
| Gambia | 822.3 (732.3 to 946.9) | 691.3 (623.2 to 778.6) | -0.6 (-0.7 to -0.5) | 9.1 (4.4 to 16.9) | 7.6 (3.7 to 14.0) | -0.7 (-0.7 to -0.6) |
| Georgia | 2694.0 (2418.2 to 3013.0) | 2821.8 (2565.9 to 3166.9) | 0.3 (0.2 to 0.4) | 29.8 (14.1 to 56.7) | 31.1 (14.8 to 58.9) | 0.3 (0.2 to 0.4) |
| Germany | 2239.5 (1987.5 to 2556.7) | 2011.8 (1776.3 to 2336.7) | -0.4 (-0.4 to -0.3) | 24.7 (11.5 to 46.5) | 22.2 (10.4 to 41.7) | -0.4 (-0.4 to -0.3) |
| Ghana | 677.3 (618.6 to 735.9) | 627.7 (570.3 to 691.7) | -0.2 (-0.3 to -0.1) | 7.4 (3.5 to 13.8) | 6.9 (3.3 to 12.8) | -0.2 (-0.3 to -0.1) |
| Greece | 2474.4 (2207.5 to 2786.9) | 2063.9 (1843.1 to 2357.9) | -0.6 (-0.7 to -0.5) | 27.3 (12.7 to 51.2) | 22.8 (10.6 to 43.5) | -0.6 (-0.7 to -0.5) |
| Greenland | 2456.3 (2246.6 to 2702.4) | 1757.7 (1595.2 to 1951.7) | -1.3 (-1.4 to -1.2) | 26.9 (12.7 to 49.5) | 19.2 (9.2 to 35.6) | -1.3 (-1.4 to -1.2) |
| Grenada | 1733.4 (1534.6 to 2047.8) | 1773.2 (1587.1 to 2020.8) | 0.1 (0.0 to 0.3) | 19.2 (9.1 to 36.5) | 19.5 (9.2 to 35.7) | 0.1 (0.0 to 0.3) |
| Guam | 722.7 (657.0 to 804.0) | 595.3 (530.8 to 680.9) | -0.7 (-0.8 to -0.6) | 8.0 (3.7 to 14.8) | 6.6 (3.2 to 12.3) | -0.7 (-0.8 to -0.6) |
| Guatemala | 2635.6 (2194.1 to 3446.9) | 2554.5 (2231.9 to 3088.0) | -0.1 (-0.1 to 0.0) | 29.0 (13.9 to 56.0) | 27.9 (13.3 to 53.0) | -0.1 (-0.1 to -0.1) |
| Guinea | 849.0 (772.9 to 921.6) | 795.1 (725.9 to 873.4) | -0.1 (-0.2 to -0.1) | 9.3 (4.4 to 17.2) | 8.7 (4.2 to 15.9) | -0.1 (-0.2 to -0.1) |
| Guinea-Bissau | 920.3 (845.3 to 993.9) | 767.5 (695.1 to 864.3) | -0.6 (-0.7 to -0.5) | 10.1 (4.7 to 18.7) | 8.4 (4.0 to 15.7) | -0.6 (-0.7 to -0.5) |
| Guyana | 1325.3 (1225.4 to 1452.8) | 1480.6 (1358.6 to 1626.4) | 0.3 (0.1 to 0.4) | 14.5 (6.9 to 27.0) | 16.1 (7.7 to 29.9) | 0.3 (0.1 to 0.4) |
| Haiti | 1773.1 (1633.9 to 1949.1) | 2032.8 (1712.6 to 2580.2) | 0.8 (0.5 to 1.0) | 19.4 (9.2 to 35.8) | 22.2 (10.6 to 41.9) | 0.8 (0.5 to 1.0) |
| Honduras | 2030.8 (1834.3 to 2273.0) | 1671.6 (1495.2 to 1880.7) | -0.7 (-0.8 to -0.6) | 22.3 (10.6 to 41.6) | 18.3 (8.7 to 33.8) | -0.7 (-0.8 to -0.6) |
| Hungary | 3890.0 (3523.3 to 4341.8) | 3167.1 (2847.4 to 3539.0) | -0.6 (-0.7 to -0.5) | 42.7 (20.0 to 79.2) | 34.9 (16.3 to 65.5) | -0.6 (-0.7 to -0.5) |
| Iceland | 2630.7 (2360.7 to 2982.8) | 2155.1 (1913.2 to 2478.4) | -0.6 (-0.7 to -0.5) | 29.1 (13.4 to 55.3) | 23.8 (11.2 to 45.2) | -0.6 (-0.7 to -0.5) |
| India | 1067.9 (950.6 to 1207.1) | 802.8 (704.9 to 913.8) | -1.1 (-1.2 to -1.0) | 11.6 (5.4 to 21.2) | 8.7 (4.2 to 16.2) | -1.1 (-1.2 to -1.0) |
| Indonesia | 1038.8 (941.7 to 1173.6) | 741.5 (673.1 to 843.5) | -1.0 (-1.1 to -0.9) | 11.5 (5.4 to 21.4) | 8.2 (3.8 to 15.3) | -1.0 (-1.1 to -0.9) |
| Iran (Islamic Republic of) | 1935.8 (1690.4 to 2321.6) | 1347.5 (1189.7 to 1575.6) | -1.2 (-1.3 to -1.1) | 21.3 (10.3 to 39.2) | 14.7 (7.2 to 27.5) | -1.2 (-1.3 to -1.1) |
| Iraq | 3652.7 (2973.8 to 4890.2) | 3268.7 (2393.5 to 4979.3) | -0.2 (-0.3 to -0.2) | 40.1 (19.5 to 80.1) | 35.6 (17.0 to 74.5) | -0.2 (-0.3 to -0.2) |
| Ireland | 2605.8 (2345.5 to 2945.3) | 2153.3 (1893.2 to 2487.3) | -0.6 (-0.7 to -0.5) | 28.8 (13.3 to 55.0) | 23.8 (11.1 to 45.0) | -0.6 (-0.7 to -0.5) |
| Israel | 2527.3 (2242.9 to 2861.0) | 2142.4 (1847.4 to 2608.3) | -0.4 (-0.5 to -0.2) | 28.0 (13.1 to 52.0) | 23.7 (11.5 to 44.2) | -0.4 (-0.5 to -0.2) |
| Italy | 2774.6 (2471.5 to 3127.0) | 2020.4 (1745.4 to 2431.8) | -1.3 (-1.4 to -1.1) | 30.5 (14.1 to 57.9) | 22.3 (10.4 to 42.3) | -1.3 (-1.4 to -1.1) |
| Jamaica | 1807.2 (1640.6 to 1982.9) | 1618.4 (1469.9 to 1804.1) | -0.8 (-0.9 to -0.6) | 20.0 (9.5 to 36.9) | 17.8 (8.4 to 33.2) | -0.8 (-1.0 to -0.6) |
| Japan | 2422.4 (2211.8 to 2673.6) | 1804.2 (1648.7 to 2009.6) | -1.0 (-1.1 to -0.8) | 26.8 (12.6 to 49.2) | 20.0 (9.5 to 36.5) | -1.0 (-1.1 to -0.8) |
| Jordan | 1186.9 (1081.5 to 1306.8) | 918.0 (822.7 to 1025.3) | -0.8 (-0.9 to -0.7) | 13.1 (6.2 to 24.2) | 10.1 (4.8 to 18.9) | -0.8 (-0.9 to -0.7) |
| Kazakhstan | 2739.9 (2486.5 to 3047.4) | 2194.9 (2002.4 to 2444.2) | -0.8 (-0.9 to -0.7) | 30.2 (14.1 to 56.4) | 24.2 (11.5 to 45.4) | -0.8 (-0.9 to -0.7) |
| Kenya | 800.5 (737.4 to 863.7) | 701.9 (644.3 to 766.5) | -0.6 (-0.8 to -0.4) | 8.8 (4.2 to 16.2) | 7.7 (3.7 to 14.2) | -0.6 (-0.8 to -0.4) |
| Kiribati | 548.8 (506.5 to 607.7) | 453.1 (413.8 to 520.2) | -0.5 (-0.7 to -0.4) | 6.0 (2.8 to 11.1) | 5.0 (2.3 to 9.2) | -0.5 (-0.6 to -0.4) |
| Kuwait | 1379.9 (1251.6 to 1539.7) | 1251.4 (1085.7 to 1476.1) | -0.6 (-0.8 to -0.4) | 15.2 (7.2 to 28.2) | 13.7 (6.5 to 25.9) | -0.6 (-0.8 to -0.4) |
| Kyrgyzstan | 2155.0 (1940.1 to 2426.2) | 1530.1 (1399.0 to 1704.3) | -1.2 (-1.2 to -1.1) | 23.8 (11.1 to 44.6) | 16.9 (8.0 to 31.6) | -1.2 (-1.2 to -1.1) |
| Lao People's Democratic Republic | 1001.1 (930.4 to 1076.7) | 718.8 (657.5 to 797.8) | -1.1 (-1.2 to -1.1) | 11.0 (5.2 to 20.7) | 7.9 (3.8 to 14.6) | -1.1 (-1.2 to -1.1) |
| Latvia | 3729.3 (3375.0 to 4107.4) | 2418.8 (2199.7 to 2698.1) | -1.7 (-1.8 to -1.6) | 40.9 (19.1 to 77.5) | 26.6 (12.7 to 49.5) | -1.7 (-1.8 to -1.6) |
| Lebanon | 2547.6 (1748.6 to 4330.3) | 1611.8 (1177.7 to 2543.6) | -1.5 (-1.6 to -1.4) | 28.1 (13.1 to 60.3) | 17.6 (8.4 to 35.2) | -1.6 (-1.7 to -1.5) |
| Lesotho | 736.5 (679.8 to 797.6) | 845.0 (785.3 to 912.7) | 0.5 (0.4 to 0.7) | 8.1 (3.9 to 15.1) | 9.2 (4.4 to 17.2) | 0.5 (0.4 to 0.6) |
| Liberia | 868.4 (778.9 to 977.3) | 1115.3 (810.2 to 1764.3) | 0.5 (0.1 to 0.8) | 9.5 (4.6 to 17.5) | 12.2 (5.7 to 25.1) | 0.5 (0.1 to 0.9) |
| Libya | 1435.5 (1301.6 to 1609.7) | 1667.7 (1379.7 to 2235.4) | 0.8 (0.6 to 1.0) | 15.8 (7.5 to 29.5) | 18.3 (8.8 to 35.8) | 0.8 (0.6 to 1.0) |
| Lithuania | 3265.1 (2946.1 to 3632.1) | 2337.1 (2112.5 to 2598.6) | -1.2 (-1.4 to -1.1) | 35.8 (16.8 to 66.8) | 25.7 (12.0 to 47.9) | -1.2 (-1.4 to -1.0) |
| Luxembourg | 2869.1 (2578.0 to 3217.2) | 2243.6 (1978.2 to 2552.7) | -0.9 (-0.9 to -0.8) | 31.6 (14.9 to 59.0) | 24.8 (11.7 to 46.3) | -0.8 (-0.9 to -0.8) |
| Madagascar | 835.7 (765.0 to 910.9) | 645.3 (592.9 to 704.8) | -0.8 (-0.9 to -0.7) | 9.2 (4.3 to 17.0) | 7.1 (3.4 to 13.1) | -0.8 (-0.8 to -0.7) |
| Malawi | 748.8 (681.6 to 819.2) | 604.7 (550.5 to 669.8) | -0.7 (-0.8 to -0.6) | 8.2 (3.9 to 15.5) | 6.7 (3.2 to 12.7) | -0.7 (-0.8 to -0.6) |
| Malaysia | 819.7 (753.5 to 896.7) | 696.8 (636.8 to 768.3) | -0.6 (-0.6 to -0.5) | 9.0 (4.2 to 16.7) | 7.7 (3.6 to 14.1) | -0.6 (-0.6 to -0.5) |
| Maldives | 1100.0 (1004.1 to 1217.0) | 845.7 (761.5 to 948.9) | -0.7 (-0.9 to -0.6) | 12.1 (5.8 to 22.5) | 9.4 (4.5 to 17.7) | -0.7 (-0.8 to -0.6) |
| Mali | 853.3 (773.4 to 927.3) | 1021.9 (853.5 to 1298.8) | 0.7 (0.6 to 0.9) | 9.3 (4.4 to 17.2) | 11.2 (5.4 to 21.4) | 0.8 (0.6 to 0.9) |
| Malta | 2514.6 (2238.2 to 2863.3) | 2317.6 (2044.4 to 2669.3) | -0.2 (-0.3 to -0.1) | 27.8 (12.9 to 52.1) | 25.6 (12.0 to 48.8) | -0.2 (-0.3 to -0.1) |
| Marshall Islands | 733.5 (672.1 to 807.5) | 625.6 (569.9 to 697.7) | -0.5 (-0.6 to -0.5) | 8.0 (3.8 to 15.0) | 6.8 (3.3 to 12.9) | -0.6 (-0.6 to -0.5) |
| Mauritania | 781.3 (720.0 to 842.5) | 638.7 (581.3 to 705.2) | -0.7 (-0.8 to -0.6) | 8.6 (4.1 to 16.0) | 7.0 (3.3 to 13.1) | -0.7 (-0.7 to -0.6) |
| Mauritius | 765.5 (702.6 to 841.9) | 644.2 (581.5 to 721.8) | -0.4 (-0.5 to -0.3) | 8.4 (4.0 to 15.6) | 7.1 (3.4 to 13.0) | -0.4 (-0.5 to -0.4) |
| Mexico | 2662.0 (2396.4 to 2988.9) | 1634.6 (1470.3 to 1829.4) | 0.0 (-0.6 to 0.6) | 29.2 (13.7 to 55.1) | 17.9 (8.4 to 33.0) | 0.0 (-0.6 to 0.6) |
| Micronesia (Federated States of) | 734.5 (678.5 to 810.1) | 675.2 (608.0 to 758.9) | -0.2 (-0.3 to -0.2) | 8.1 (3.8 to 15.0) | 7.4 (3.5 to 13.7) | -0.2 (-0.3 to -0.2) |
| Monaco | 2141.2 (1902.8 to 2461.2) | 2067.6 (1827.0 to 2374.0) | -0.1 (-0.2 to 0.0) | 23.7 (11.0 to 44.9) | 22.8 (10.7 to 43.1) | -0.1 (-0.2 to 0.0) |
| Mongolia | 2420.5 (2208.8 to 2675.6) | 2395.7 (2175.4 to 2677.5) | 0.1 (0.0 to 0.2) | 26.7 (12.7 to 49.4) | 26.4 (12.5 to 50.1) | 0.1 (0.0 to 0.2) |
| Montenegro | 3820.0 (3449.4 to 4262.8) | 3306.3 (2966.8 to 3687.7) | -0.5 (-0.6 to -0.4) | 42.2 (19.8 to 78.9) | 36.4 (17.0 to 67.9) | -0.5 (-0.6 to -0.4) |
| Morocco | 1622.4 (1492.4 to 1784.3) | 1208.1 (1097.7 to 1331.8) | -1.1 (-1.2 to -0.9) | 17.8 (8.6 to 33.1) | 13.2 (6.3 to 24.5) | -1.1 (-1.2 to -1.0) |
| Mozambique | 1443.6 (976.6 to 2462.8) | 1083.7 (807.4 to 1682.7) | -1.0 (-1.0 to -0.9) | 15.9 (7.2 to 33.3) | 11.8 (5.6 to 23.8) | -1.0 (-1.1 to -0.9) |
| Myanmar | 1356.6 (1163.9 to 1780.3) | 1200.0 (982.5 to 1537.7) | -0.4 (-0.5 to -0.3) | 15.0 (7.0 to 29.5) | 13.2 (6.4 to 25.7) | -0.4 (-0.5 to -0.3) |
| Namibia | 1142.2 (912.5 to 1598.5) | 927.2 (786.7 to 1185.0) | -0.7 (-0.8 to -0.6) | 12.6 (6.1 to 25.3) | 10.1 (5.0 to 19.2) | -0.8 (-0.9 to -0.7) |
| Nauru | 791.8 (728.4 to 870.5) | 711.8 (642.3 to 795.6) | -0.4 (-0.5 to -0.3) | 8.7 (4.1 to 16.4) | 7.8 (3.7 to 14.4) | -0.4 (-0.5 to -0.3) |
| Nepal | 966.5 (853.7 to 1098.1) | 1043.2 (867.9 to 1276.4) | 0.4 (0.2 to 0.5) | 10.5 (4.9 to 19.5) | 11.4 (5.6 to 21.2) | 0.4 (0.3 to 0.5) |
| Netherlands | 1888.7 (1713.7 to 2124.3) | 1714.5 (1544.1 to 1973.4) | -0.2 (-0.5 to 0.0) | 20.9 (9.6 to 39.1) | 18.9 (8.8 to 35.2) | -0.2 (-0.5 to 0.0) |
| New Zealand | 6191.5 (5480.4 to 7012.6) | 5309.4 (4696.6 to 6099.1) | -0.6 (-0.7 to -0.4) | 67.9 (32.0 to 127.3) | 58.4 (26.8 to 109.9) | -0.6 (-0.7 to -0.4) |
| Nicaragua | 2747.6 (2025.6 to 4251.8) | 1970.0 (1541.1 to 2769.8) | -1.1 (-1.1 to -1.0) | 30.4 (14.1 to 61.9) | 21.6 (10.3 to 41.3) | -1.1 (-1.2 to -1.0) |
| Niger | 850.3 (772.3 to 932.7) | 764.0 (691.2 to 854.9) | -0.4 (-0.4 to -0.3) | 9.3 (4.4 to 17.5) | 8.4 (4.0 to 15.3) | -0.4 (-0.4 to -0.3) |
| Nigeria | 825.6 (752.8 to 891.9) | 671.9 (606.6 to 749.7) | -0.8 (-0.9 to -0.6) | 9.0 (4.3 to 16.9) | 7.4 (3.6 to 13.6) | -0.7 (-0.8 to -0.6) |
| Niue | 692.6 (629.1 to 771.7) | 635.3 (565.1 to 722.8) | -0.3 (-0.4 to -0.2) | 7.6 (3.6 to 14.0) | 7.0 (3.3 to 12.9) | -0.3 (-0.4 to -0.2) |
| North Macedonia | 3804.2 (3443.6 to 4214.8) | 3366.3 (3025.4 to 3777.5) | -0.4 (-0.5 to -0.3) | 41.9 (19.8 to 77.7) | 37.1 (17.3 to 69.3) | -0.4 (-0.5 to -0.3) |
| Northern Mariana Islands | 1024.9 (939.5 to 1134.7) | 798.3 (715.2 to 896.6) | -0.9 (-0.9 to -0.9) | 11.3 (5.4 to 21.1) | 8.8 (4.1 to 16.6) | -0.9 (-0.9 to -0.9) |
| Norway | 2755.8 (2489.8 to 3088.9) | 1986.8 (1784.1 to 2263.3) | -1.0 (-1.1 to -0.9) | 30.3 (14.1 to 57.1) | 21.9 (10.4 to 41.4) | -1.0 (-1.1 to -0.9) |
| Oman | 1753.4 (1614.7 to 1911.5) | 1263.6 (1137.6 to 1420.0) | -1.1 (-1.2 to -1.0) | 19.3 (9.2 to 35.6) | 13.9 (6.6 to 25.8) | -1.1 (-1.2 to -1.0) |
| Pakistan | 656.4 (604.4 to 728.4) | 663.7 (578.2 to 794.0) | 0.1 (0.0 to 0.2) | 7.2 (3.4 to 13.6) | 7.3 (3.5 to 13.7) | 0.1 (0.0 to 0.2) |
| Palau | 1036.0 (943.1 to 1150.7) | 1012.2 (906.5 to 1146.3) | -0.1 (-0.1 to 0.0) | 11.4 (5.4 to 21.1) | 11.1 (5.2 to 20.8) | -0.1 (-0.1 to -0.1) |
| Palestine | 2076.0 (1563.5 to 3008.7) | 2389.8 (1592.2 to 4007.7) | 1.0 (0.8 to 1.3) | 23.0 (10.4 to 47.7) | 26.3 (11.7 to 58.6) | 1.0 (0.7 to 1.3) |
| Panama | 1805.3 (1591.5 to 2094.7) | 1580.0 (1388.9 to 1824.7) | -0.5 (-0.6 to -0.4) | 19.9 (9.2 to 37.4) | 17.4 (8.1 to 32.1) | -0.5 (-0.6 to -0.4) |
| Papua New Guinea | 629.4 (574.9 to 704.7) | 733.1 (647.6 to 849.1) | 0.4 (0.3 to 0.6) | 6.9 (3.2 to 12.8) | 8.0 (3.8 to 14.6) | 0.4 (0.3 to 0.6) |
| Paraguay | 1571.3 (1378.8 to 1802.8) | 1337.6 (1189.1 to 1530.8) | -0.6 (-0.7 to -0.5) | 17.3 (8.2 to 32.7) | 14.7 (7.0 to 27.3) | -0.6 (-0.7 to -0.5) |
| Peru | 1676.3 (1491.9 to 1941.3) | 1460.1 (1311.2 to 1692.5) | -0.5 (-0.6 to -0.5) | 18.5 (8.9 to 34.4) | 16.1 (7.6 to 30.4) | -0.5 (-0.6 to -0.5) |
| Philippines | 1086.4 (946.0 to 1361.9) | 814.4 (691.2 to 1020.5) | -0.8 (-0.9 to -0.7) | 12.0 (5.7 to 23.3) | 9.0 (4.3 to 17.2) | -0.8 (-0.9 to -0.7) |
| Poland | 3920.2 (3561.5 to 4353.1) | 2926.1 (2648.7 to 3266.8) | -0.9 (-1.0 to -0.8) | 42.9 (20.2 to 80.2) | 32.1 (15.2 to 60.4) | -0.9 (-1.0 to -0.8) |
| Portugal | 2765.2 (2495.7 to 3098.5) | 1589.4 (1424.0 to 1819.4) | -2.0 (-2.1 to -1.9) | 30.5 (14.3 to 56.7) | 17.5 (8.1 to 33.1) | -2.0 (-2.1 to -1.9) |
| Puerto Rico | 1401.9 (1270.3 to 1548.1) | 1455.8 (1307.7 to 1635.6) | 0.1 (-0.1 to 0.2) | 15.5 (7.2 to 28.8) | 16.0 (7.5 to 29.9) | 0.1 (-0.1 to 0.2) |
| Qatar | 1812.0 (1655.7 to 2011.3) | 1433.5 (1288.2 to 1604.3) | -0.6 (-0.7 to -0.5) | 19.9 (9.5 to 37.0) | 15.7 (7.4 to 29.2) | -0.6 (-0.8 to -0.5) |
| Republic of Korea | 3172.6 (2866.1 to 3527.5) | 2012.9 (1822.1 to 2256.3) | -1.9 (-2.0 to -1.7) | 34.9 (16.3 to 65.3) | 22.2 (10.5 to 41.2) | -1.9 (-2.0 to -1.7) |
| Republic of Moldova | 3127.5 (2842.5 to 3460.1) | 2017.8 (1824.9 to 2240.5) | -1.6 (-1.7 to -1.6) | 34.4 (16.1 to 64.3) | 22.2 (10.4 to 41.4) | -1.6 (-1.7 to -1.6) |
| Romania | 4661.4 (4202.8 to 5176.9) | 3485.9 (3166.8 to 3889.7) | -1.1 (-1.1 to -1.0) | 51.2 (24.2 to 96.2) | 38.4 (18.2 to 71.7) | -1.1 (-1.1 to -1.0) |
| Russian Federation | 3848.8 (3496.7 to 4229.2) | 2527.2 (2301.4 to 2825.2) | -1.7 (-2.0 to -1.5) | 42.2 (19.9 to 79.5) | 27.7 (13.1 to 51.4) | -1.7 (-2.0 to -1.5) |
| Rwanda | 833.2 (761.9 to 908.8) | 1366.6 (921.4 to 2156.7) | 0.7 (-0.2 to 1.6) | 9.1 (4.4 to 17.1) | 15.0 (6.9 to 31.3) | 0.7 (-0.2 to 1.6) |
| Saint Kitts and Nevis | 1774.8 (1620.0 to 1968.3) | 1951.1 (1750.2 to 2190.6) | 0.5 (0.3 to 0.7) | 19.5 (9.2 to 36.4) | 21.4 (10.0 to 40.4) | 0.5 (0.3 to 0.7) |
| Saint Lucia | 1329.4 (1223.8 to 1459.4) | 1451.6 (1321.6 to 1606.3) | 0.3 (0.2 to 0.5) | 14.6 (6.9 to 27.7) | 15.9 (7.6 to 29.9) | 0.3 (0.1 to 0.4) |
| Saint Vincent and the Grenadines | 1410.9 (1285.7 to 1568.8) | 1527.4 (1397.1 to 1677.8) | 0.2 (0.1 to 0.4) | 15.6 (7.4 to 28.7) | 16.8 (7.9 to 31.3) | 0.2 (0.0 to 0.4) |
| Samoa | 682.8 (623.3 to 755.9) | 686.0 (600.9 to 819.7) | 0.1 (0.0 to 0.2) | 7.5 (3.6 to 14.0) | 7.5 (3.7 to 14.0) | 0.1 (0.0 to 0.2) |
| San Marino | 2423.8 (2161.7 to 2740.1) | 2230.7 (1976.2 to 2562.9) | -0.2 (-0.3 to -0.2) | 26.8 (12.5 to 50.2) | 24.6 (11.5 to 46.5) | -0.2 (-0.3 to -0.2) |
| Sao Tome and Principe | 917.3 (820.0 to 1049.7) | 834.9 (745.6 to 936.5) | -0.3 (-0.4 to -0.2) | 10.1 (4.7 to 19.0) | 9.2 (4.3 to 17.2) | -0.3 (-0.4 to -0.2) |
| Saudi Arabia | 2688.4 (2442.8 to 2976.9) | 2402.3 (2126.4 to 2711.3) | -0.1 (-0.4 to 0.3) | 29.5 (13.9 to 54.4) | 26.2 (12.5 to 49.0) | -0.1 (-0.5 to 0.3) |
| Senegal | 747.3 (674.3 to 817.1) | 661.2 (597.8 to 739.2) | -0.4 (-0.5 to -0.3) | 8.2 (3.9 to 15.1) | 7.3 (3.5 to 13.4) | -0.4 (-0.5 to -0.3) |
| Serbia | 3817.6 (3455.5 to 4266.3) | 3319.6 (2945.4 to 3770.9) | -0.4 (-0.5 to -0.3) | 42.1 (19.8 to 78.3) | 36.6 (17.2 to 69.0) | -0.4 (-0.5 to -0.3) |
| Seychelles | 1004.4 (919.6 to 1109.0) | 742.1 (675.6 to 821.2) | -0.9 (-1.0 to -0.8) | 11.1 (5.3 to 20.8) | 8.2 (3.9 to 15.1) | -0.9 (-1.0 to -0.8) |
| Sierra Leone | 790.8 (720.5 to 871.7) | 1020.9 (792.4 to 1464.3) | 0.3 (-0.2 to 0.7) | 8.7 (4.2 to 16.0) | 11.2 (5.3 to 22.3) | 0.3 (-0.2 to 0.8) |
| Singapore | 2520.5 (2287.3 to 2816.7) | 2208.3 (1991.7 to 2468.6) | -0.5 (-0.6 to -0.4) | 27.9 (13.0 to 51.8) | 24.5 (11.4 to 45.2) | -0.5 (-0.6 to -0.4) |
| Slovakia | 4444.7 (4043.8 to 4976.8) | 3691.0 (3326.4 to 4140.3) | -0.6 (-0.6 to -0.6) | 48.8 (22.9 to 93.2) | 40.6 (19.0 to 77.0) | -0.6 (-0.6 to -0.5) |
| Slovenia | 5348.1 (4807.2 to 5948.7) | 4179.0 (3753.0 to 4686.1) | -0.3 (-0.7 to 0.0) | 58.6 (27.7 to 110.1) | 46.0 (21.6 to 86.9) | -0.3 (-0.7 to 0.0) |
| Solomon Islands | 835.5 (764.3 to 916.2) | 845.6 (749.1 to 963.8) | 0.0 (0.0 to 0.0) | 9.2 (4.4 to 16.9) | 9.2 (4.4 to 17.6) | 0.0 (0.0 to 0.0) |
| Somalia | 1011.2 (845.8 to 1558.1) | 1222.9 (937.2 to 1844.5) | 0.4 (0.3 to 0.5) | 11.1 (5.2 to 21.9) | 13.5 (6.6 to 26.6) | 0.4 (0.3 to 0.5) |
| South Africa | 1205.0 (1108.1 to 1335.4) | 793.4 (727.7 to 879.6) | -1.5 (-1.6 to -1.3) | 13.2 (6.2 to 24.9) | 8.6 (4.0 to 16.1) | -1.5 (-1.7 to -1.4) |
| South Sudan | 1053.2 (900.1 to 1318.2) | 1273.0 (953.4 to 1930.7) | 0.9 (0.7 to 1.0) | 11.5 (5.7 to 22.0) | 14.0 (6.5 to 29.0) | 0.9 (0.7 to 1.0) |
| Spain | 2427.1 (2208.7 to 2702.6) | 1969.0 (1736.3 to 2281.5) | -0.7 (-0.7 to -0.6) | 26.8 (12.7 to 50.3) | 21.8 (10.2 to 40.9) | -0.7 (-0.7 to -0.6) |
| Sri Lanka | 1400.8 (1211.7 to 1749.2) | 1712.9 (1224.1 to 2651.9) | 0.5 (0.3 to 0.7) | 15.5 (7.4 to 30.3) | 18.9 (9.0 to 40.4) | 0.5 (0.3 to 0.7) |
| Sudan | 1416.3 (1270.1 to 1623.7) | 1380.4 (1142.6 to 1832.3) | 0.0 (-0.1 to 0.2) | 15.6 (7.4 to 29.1) | 15.2 (7.4 to 29.0) | 0.0 (-0.1 to 0.2) |
| Suriname | 1313.4 (1149.1 to 1703.3) | 1312.9 (1181.4 to 1585.3) | 0.1 (-0.1 to 0.2) | 14.5 (6.7 to 28.8) | 14.4 (6.8 to 27.3) | 0.0 (-0.1 to 0.2) |
| Sweden | 2572.8 (2307.1 to 2897.8) | 1998.8 (1775.2 to 2292.2) | -0.8 (-0.9 to -0.7) | 28.4 (13.2 to 53.4) | 22.1 (10.2 to 42.0) | -0.8 (-0.9 to -0.7) |
| Switzerland | 3712.4 (3304.5 to 4179.5) | 2548.4 (2235.4 to 2932.8) | -1.5 (-1.7 to -1.3) | 40.9 (19.0 to 77.6) | 28.1 (13.1 to 52.6) | -1.5 (-1.7 to -1.3) |
| Syrian Arab Republic | 1234.7 (1079.4 to 1498.9) | 2653.0 (1618.5 to 4860.3) | 2.4 (1.7 to 3.1) | 13.6 (6.3 to 27.0) | 29.4 (12.4 to 69.8) | 2.4 (1.7 to 3.1) |
| Taiwan (Province of China) | 720.9 (643.3 to 812.7) | 425.4 (383.7 to 479.3) | -2.1 (-2.2 to -2.0) | 8.0 (3.8 to 14.9) | 4.7 (2.2 to 8.7) | -2.1 (-2.2 to -2.0) |
| Tajikistan | 1955.1 (1768.1 to 2190.4) | 1758.0 (1516.3 to 2141.9) | -0.7 (-0.9 to -0.5) | 21.6 (10.2 to 40.3) | 19.4 (9.3 to 36.9) | -0.7 (-0.9 to -0.5) |
| Thailand | 1127.2 (1042.6 to 1232.4) | 866.6 (778.9 to 969.3) | -1.0 (-1.1 to -1.0) | 12.4 (5.9 to 22.9) | 9.6 (4.6 to 17.9) | -1.0 (-1.1 to -1.0) |
| Timor-Leste | 1902.3 (1276.2 to 3161.3) | 1824.5 (1101.0 to 3313.7) | -0.3 (-0.6 to 0.1) | 21.1 (9.6 to 47.3) | 20.1 (8.3 to 45.2) | -0.3 (-0.6 to 0.1) |
| Togo | 774.6 (706.3 to 839.1) | 662.3 (606.6 to 732.5) | -0.6 (-0.6 to -0.5) | 8.5 (4.0 to 16.0) | 7.3 (3.5 to 13.5) | -0.5 (-0.6 to -0.5) |
| Tokelau | 663.8 (610.7 to 732.7) | 565.5 (508.4 to 641.6) | -0.5 (-0.6 to -0.5) | 7.3 (3.4 to 13.5) | 6.2 (2.9 to 11.3) | -0.5 (-0.6 to -0.5) |
| Tonga | 595.2 (546.5 to 668.4) | 483.9 (440.0 to 548.5) | -0.7 (-0.7 to -0.6) | 6.6 (3.1 to 12.3) | 5.3 (2.6 to 9.9) | -0.7 (-0.8 to -0.6) |
| Trinidad and Tobago | 1220.9 (1119.2 to 1346.1) | 1242.0 (1127.7 to 1384.5) | 0.2 (0.1 to 0.3) | 13.4 (6.3 to 24.7) | 13.6 (6.5 to 25.5) | 0.2 (0.1 to 0.3) |
| Tunisia | 1865.4 (1704.7 to 2060.2) | 1425.5 (1304.8 to 1576.6) | -0.9 (-0.9 to -0.9) | 20.6 (9.6 to 38.8) | 15.6 (7.3 to 29.0) | -0.9 (-0.9 to -0.9) |
| Turkey | 1328.2 (1215.2 to 1467.7) | 1061.9 (953.4 to 1201.2) | -0.7 (-0.8 to -0.7) | 14.7 (6.9 to 27.0) | 11.7 (5.6 to 21.9) | -0.7 (-0.8 to -0.7) |
| Turkmenistan | 1985.8 (1798.4 to 2222.0) | 1888.6 (1712.8 to 2096.4) | -0.1 (-0.2 to 0.0) | 22.0 (10.4 to 41.1) | 20.9 (9.9 to 39.4) | -0.1 (-0.2 to 0.0) |
| Tuvalu | 699.6 (647.1 to 771.4) | 609.4 (550.8 to 684.5) | -1.1 (-1.4 to -0.8) | 7.7 (3.7 to 14.3) | 6.7 (3.2 to 12.6) | -1.1 (-1.4 to -0.8) |
| Uganda | 1322.1 (922.0 to 2191.2) | 1004.1 (746.9 to 1537.6) | -0.9 (-1.0 to -0.8) | 14.5 (6.7 to 31.1) | 11.0 (5.2 to 22.1) | -0.9 (-1.0 to -0.8) |
| Ukraine | 3139.8 (2787.9 to 3525.2) | 2655.9 (2406.2 to 2949.2) | -0.9 (-1.0 to -0.7) | 34.4 (16.2 to 63.8) | 29.1 (13.9 to 54.6) | -0.8 (-1.0 to -0.7) |
| United Arab Emirates | 1715.1 (1566.5 to 1880.1) | 1488.1 (1331.7 to 1680.5) | -0.4 (-0.5 to -0.4) | 18.9 (9.1 to 35.0) | 16.3 (7.8 to 30.2) | -0.5 (-0.5 to -0.4) |
| United Kingdom | 2179.7 (1961.7 to 2456.8) | 1751.7 (1558.0 to 2008.2) | -0.7 (-0.8 to -0.5) | 24.1 (11.2 to 45.1) | 19.3 (9.0 to 36.2) | -0.7 (-0.8 to -0.5) |
| United Republic of Tanzania | 803.6 (735.8 to 879.1) | 692.3 (627.9 to 759.9) | -0.5 (-0.5 to -0.4) | 8.8 (4.1 to 16.1) | 7.6 (3.7 to 14.2) | -0.4 (-0.5 to -0.4) |
| United States of America | 2225.5 (2038.3 to 2467.0) | 1356.8 (1250.0 to 1482.1) | -1.8 (-2.2 to -1.4) | 24.4 (11.5 to 45.1) | 14.8 (7.0 to 27.2) | -1.9 (-2.2 to -1.5) |
| United States Virgin Islands | 1426.5 (1299.7 to 1583.4) | 1285.2 (1172.6 to 1433.3) | -0.6 (-0.8 to -0.3) | 15.8 (7.4 to 29.4) | 14.1 (6.6 to 26.3) | -0.6 (-0.8 to -0.3) |
| Uruguay | 5447.6 (4783.5 to 6184.6) | 4031.0 (3610.3 to 4524.4) | -1.2 (-1.3 to -1.1) | 60.0 (27.8 to 113.8) | 44.3 (20.6 to 83.7) | -1.2 (-1.3 to -1.1) |
| Uzbekistan | 1967.9 (1778.8 to 2189.7) | 1612.2 (1467.5 to 1772.1) | -0.7 (-0.8 to -0.6) | 21.7 (10.2 to 40.9) | 17.8 (8.4 to 33.1) | -0.7 (-0.8 to -0.6) |
| Vanuatu | 683.6 (626.8 to 760.6) | 598.0 (537.7 to 680.3) | -0.5 (-0.6 to -0.4) | 7.5 (3.6 to 13.8) | 6.6 (3.2 to 12.3) | -0.5 (-0.6 to -0.4) |
| Venezuela (Bolivarian Republic of) | 2365.4 (2114.6 to 2674.0) | 2220.1 (1989.2 to 2480.8) | -0.1 (-0.2 to 0.0) | 26.1 (12.2 to 49.2) | 24.4 (11.4 to 45.5) | -0.1 (-0.2 to 0.0) |
| Viet Nam | 1043.0 (951.0 to 1137.2) | 893.3 (816.1 to 982.1) | -0.4 (-0.5 to -0.3) | 11.5 (5.4 to 21.4) | 9.9 (4.7 to 18.4) | -0.4 (-0.5 to -0.3) |
| Yemen | 1368.1 (1237.6 to 1567.1) | 1581.3 (1233.7 to 2279.7) | 0.2 (0.0 to 0.4) | 15.0 (7.2 to 28.6) | 17.4 (8.4 to 35.7) | 0.2 (0.0 to 0.4) |
| Zambia | 780.3 (713.1 to 848.1) | 675.0 (615.8 to 742.2) | -0.5 (-0.6 to -0.4) | 8.5 (4.1 to 15.8) | 7.4 (3.5 to 13.7) | -0.5 (-0.6 to -0.4) |
| Zimbabwe | 709.5 (654.2 to 769.2) | 651.9 (602.7 to 711.7) | -0.3 (-0.4 to -0.2) | 7.8 (3.8 to 14.6) | 7.1 (3.4 to 13.2) | -0.3 (-0.4 to -0.3) |

**Table S16. ASR of prevalence and YLDs in 1990 and 2021, and their EAPC from 1990 to 2021 for AMP-Toe, by country.**

| **Countries** | **Prevalence** | | | **YLDs** | | |
| --- | --- | --- | --- | --- | --- | --- |
|  | **Age-standardized prevalence rate, 1990** | **Age-standardized prevalence rate, 2021** | **EAPC of prevalence rate No. (95% CI)** | **Age-standardized YLDs rate, 1990** | **Age-standardized YLDs rate, 2021** | **EAPC of YLDs rate No. (95% CI)** |
| Afghanistan | 4142.0 (1738.4 to 10025.5) | 4071.4 (1973.5 to 8504.5) | -0.3 (-0.4 to -0.2) | 23.3 (6.8 to 64.9) | 22.6 (7.1 to 60.8) | -0.3 (-0.4 to -0.2) |
| Albania | 4268.7 (3783.0 to 4845.7) | 3362.5 (2992.2 to 3792.2) | -1.2 (-1.3 to -1.0) | 24.2 (10.1 to 51.0) | 19.0 (7.8 to 39.9) | -1.2 (-1.3 to -1.0) |
| Algeria | 1414.3 (1302.7 to 1538.6) | 1083.4 (971.9 to 1229.2) | -0.9 (-0.9 to -0.8) | 8.0 (3.3 to 16.3) | 6.1 (2.5 to 12.9) | -0.9 (-1.0 to -0.8) |
| American Samoa | 731.0 (666.9 to 815.4) | 694.4 (611.6 to 818.8) | 0.0 (-0.1 to 0.1) | 4.1 (1.7 to 8.6) | 3.9 (1.6 to 8.2) | 0.0 (-0.1 to 0.1) |
| Andorra | 1527.9 (1320.0 to 1825.1) | 1432.3 (1240.8 to 1710.3) | -0.2 (-0.3 to -0.1) | 8.7 (3.6 to 17.9) | 8.1 (3.3 to 17.0) | -0.2 (-0.3 to -0.1) |
| Angola | 1521.1 (1036.0 to 2434.1) | 1237.2 (812.1 to 2032.2) | -0.8 (-1.1 to -0.5) | 8.6 (3.1 to 21.7) | 6.9 (2.5 to 17.9) | -0.8 (-1.1 to -0.6) |
| Antigua and Barbuda | 1515.8 (1367.1 to 1718.6) | 1672.5 (1496.0 to 1923.0) | 0.3 (0.1 to 0.5) | 8.6 (3.5 to 17.8) | 9.4 (3.9 to 19.9) | 0.3 (0.0 to 0.5) |
| Argentina | 1932.2 (1753.9 to 2166.7) | 1716.0 (1559.9 to 1909.0) | -0.3 (-0.6 to 0.0) | 11.0 (4.6 to 23.3) | 9.7 (4.0 to 20.1) | -0.3 (-0.6 to 0.0) |
| Armenia | 2640.1 (2331.1 to 3110.5) | 1728.2 (1493.2 to 2067.1) | -1.7 (-1.9 to -1.5) | 15.0 (6.1 to 31.6) | 9.8 (4.0 to 21.5) | -1.7 (-1.9 to -1.5) |
| Australia | 2769.4 (2472.3 to 3158.6) | 2329.8 (2072.1 to 2682.4) | -0.5 (-0.6 to -0.4) | 15.6 (6.4 to 32.7) | 13.2 (5.4 to 27.7) | -0.5 (-0.6 to -0.4) |
| Austria | 1817.3 (1623.1 to 2081.9) | 1379.4 (1208.5 to 1643.2) | -0.9 (-0.9 to -0.8) | 10.3 (4.3 to 21.3) | 7.8 (3.2 to 16.4) | -0.9 (-0.9 to -0.8) |
| Azerbaijan | 1652.6 (1495.8 to 1830.7) | 1534.3 (1356.0 to 1758.9) | -0.5 (-0.6 to -0.4) | 9.4 (3.9 to 19.6) | 8.7 (3.5 to 18.9) | -0.5 (-0.6 to -0.4) |
| Bahamas | 989.9 (906.5 to 1095.0) | 1041.6 (943.2 to 1166.9) | 0.2 (0.1 to 0.3) | 5.6 (2.3 to 11.7) | 5.9 (2.4 to 12.4) | 0.2 (0.1 to 0.3) |
| Bahrain | 1028.8 (936.5 to 1139.8) | 953.4 (852.1 to 1081.1) | -0.5 (-0.6 to -0.3) | 5.8 (2.4 to 12.2) | 5.4 (2.2 to 11.2) | -0.5 (-0.6 to -0.3) |
| Bangladesh | 583.8 (532.0 to 652.9) | 615.9 (530.9 to 745.3) | -0.1 (-0.2 to 0.1) | 3.3 (1.4 to 6.9) | 3.5 (1.4 to 7.5) | -0.1 (-0.2 to 0.1) |
| Barbados | 1001.0 (910.9 to 1121.3) | 1014.3 (914.8 to 1139.6) | -0.1 (-0.3 to 0.1) | 5.7 (2.4 to 11.9) | 5.7 (2.3 to 12.0) | -0.1 (-0.3 to 0.1) |
| Belarus | 2596.3 (2325.5 to 2917.2) | 2256.9 (2007.5 to 2550.8) | -0.5 (-0.8 to -0.3) | 14.7 (6.1 to 30.4) | 12.7 (5.3 to 26.8) | -0.5 (-0.7 to -0.3) |
| Belgium | 1621.9 (1425.1 to 1941.7) | 1399.0 (1206.1 to 1683.0) | -0.2 (-0.7 to 0.3) | 9.2 (3.8 to 19.1) | 7.9 (3.2 to 16.5) | -0.2 (-0.8 to 0.3) |
| Belize | 1255.2 (1142.2 to 1412.1) | 1404.8 (1277.8 to 1578.2) | 0.2 (0.0 to 0.4) | 7.1 (3.0 to 15.1) | 7.9 (3.3 to 16.6) | 0.2 (-0.1 to 0.4) |
| Benin | 726.7 (659.8 to 788.6) | 648.3 (589.0 to 712.2) | -0.4 (-0.4 to -0.3) | 4.1 (1.7 to 8.3) | 3.7 (1.5 to 7.6) | -0.4 (-0.4 to -0.3) |
| Bermuda | 1068.4 (975.4 to 1195.2) | 1080.9 (971.4 to 1225.7) | 0.0 (-0.1 to 0.2) | 6.1 (2.5 to 12.5) | 6.1 (2.5 to 12.9) | 0.0 (-0.1 to 0.2) |
| Bhutan | 814.8 (748.4 to 889.0) | 860.1 (776.6 to 961.4) | 0.1 (0.0 to 0.2) | 4.6 (1.9 to 9.4) | 4.9 (2.0 to 10.0) | 0.1 (0.0 to 0.2) |
| Bolivia (Plurinational State of) | 1279.9 (1184.8 to 1401.1) | 1055.0 (966.3 to 1172.2) | -0.7 (-0.7 to -0.7) | 7.2 (3.0 to 14.8) | 5.9 (2.5 to 12.3) | -0.7 (-0.7 to -0.7) |
| Bosnia and Herzegovina | 4031.0 (3592.0 to 4559.8) | 3166.7 (2703.6 to 3848.3) | -1.1 (-1.3 to -0.9) | 22.8 (9.3 to 48.9) | 17.9 (7.1 to 40.6) | -1.1 (-1.3 to -0.9) |
| Botswana | 728.2 (669.4 to 793.7) | 736.0 (676.8 to 800.0) | -0.1 (-0.2 to 0.1) | 4.1 (1.7 to 8.3) | 4.1 (1.7 to 8.3) | -0.1 (-0.2 to 0.0) |
| Brazil | 1701.6 (1489.7 to 1957.1) | 1314.3 (1146.6 to 1508.4) | -1.1 (-1.3 to -0.9) | 9.5 (3.9 to 19.9) | 7.4 (3.0 to 15.1) | -1.1 (-1.3 to -0.9) |
| Brunei Darussalam | 1527.6 (1403.5 to 1698.0) | 1251.6 (1134.5 to 1400.7) | -0.7 (-0.7 to -0.6) | 8.6 (3.6 to 17.9) | 7.1 (2.9 to 14.8) | -0.7 (-0.7 to -0.6) |
| Bulgaria | 3749.7 (3344.4 to 4185.5) | 3162.4 (2802.9 to 3562.6) | -0.5 (-0.6 to -0.5) | 21.2 (9.0 to 44.1) | 17.9 (7.4 to 37.9) | -0.5 (-0.6 to -0.4) |
| Burkina Faso | 690.2 (627.9 to 753.0) | 674.6 (608.3 to 749.9) | -0.1 (-0.2 to -0.1) | 3.9 (1.6 to 8.0) | 3.8 (1.6 to 8.0) | -0.1 (-0.1 to 0.0) |
| Burundi | 726.2 (663.6 to 787.8) | 2244.4 (1216.1 to 4187.3) | 3.9 (2.8 to 5.1) | 4.1 (1.7 to 8.5) | 12.7 (4.3 to 34.4) | 3.9 (2.8 to 5.1) |
| Cabo Verde | 770.3 (702.0 to 843.0) | 674.0 (614.2 to 740.4) | -0.4 (-0.5 to -0.4) | 4.4 (1.8 to 9.0) | 3.8 (1.6 to 7.9) | -0.4 (-0.5 to -0.4) |
| Cambodia | 1796.3 (1035.6 to 3603.2) | 1284.9 (858.8 to 2208.7) | -1.0 (-1.2 to -0.9) | 10.2 (3.3 to 24.7) | 7.2 (2.6 to 17.0) | -1.0 (-1.2 to -0.9) |
| Cameroon | 649.2 (587.2 to 713.4) | 653.0 (581.5 to 744.6) | -0.1 (-0.1 to 0.0) | 3.7 (1.5 to 7.5) | 3.7 (1.5 to 7.9) | 0.0 (-0.1 to 0.0) |
| Canada | 1047.0 (966.2 to 1150.3) | 799.6 (732.1 to 876.6) | -0.9 (-0.9 to -0.8) | 5.9 (2.5 to 12.2) | 4.5 (1.9 to 9.3) | -0.9 (-0.9 to -0.9) |
| Central African Republic | 725.6 (670.5 to 781.1) | 971.7 (763.3 to 1337.7) | 1.0 (0.8 to 1.2) | 4.0 (1.7 to 8.2) | 5.5 (2.1 to 13.4) | 1.1 (0.9 to 1.2) |
| Chad | 1027.9 (781.6 to 1477.1) | 1001.0 (781.1 to 1390.3) | -0.1 (-0.2 to 0.0) | 5.8 (2.2 to 13.8) | 5.6 (2.2 to 13.5) | -0.1 (-0.2 to 0.0) |
| Chile | 1691.7 (1532.9 to 1897.3) | 1895.1 (1695.8 to 2193.8) | 0.5 (0.5 to 0.6) | 9.6 (4.0 to 20.0) | 10.7 (4.4 to 22.4) | 0.5 (0.5 to 0.6) |
| China | 797.1 (709.4 to 918.0) | 728.5 (637.5 to 841.7) | -0.7 (-1.0 to -0.4) | 4.5 (1.8 to 9.4) | 4.1 (1.7 to 8.5) | -0.7 (-1.0 to -0.4) |
| Colombia | 2147.3 (1925.3 to 2424.9) | 1543.7 (1338.8 to 1791.6) | -1.0 (-1.0 to -0.9) | 12.1 (5.0 to 25.1) | 8.7 (3.5 to 19.1) | -0.9 (-1.0 to -0.9) |
| Comoros | 736.8 (676.2 to 796.6) | 668.3 (605.8 to 741.0) | -0.4 (-0.5 to -0.4) | 4.2 (1.7 to 8.7) | 3.8 (1.5 to 8.2) | -0.4 (-0.5 to -0.4) |
| Congo | 670.9 (615.5 to 727.7) | 851.1 (630.5 to 1260.3) | 0.8 (0.2 to 1.4) | 3.8 (1.6 to 7.6) | 4.8 (1.8 to 11.5) | 0.8 (0.2 to 1.5) |
| Cook Islands | 632.2 (574.0 to 708.2) | 635.8 (556.0 to 752.7) | 0.0 (-0.1 to 0.2) | 3.6 (1.5 to 7.5) | 3.6 (1.5 to 7.5) | 0.0 (-0.1 to 0.2) |
| Costa Rica | 1494.1 (1305.4 to 1726.8) | 1343.6 (1169.8 to 1552.8) | -0.3 (-0.4 to -0.2) | 8.5 (3.5 to 17.6) | 7.6 (3.1 to 15.7) | -0.3 (-0.4 to -0.2) |
| Coted'Ivoire | 708.5 (644.7 to 770.9) | 674.0 (606.2 to 750.3) | -0.2 (-0.2 to -0.1) | 4.0 (1.6 to 8.1) | 3.8 (1.6 to 8.0) | -0.1 (-0.2 to -0.1) |
| Croatia | 3349.8 (3029.6 to 3758.5) | 2602.1 (2280.5 to 2995.9) | -0.8 (-0.9 to -0.8) | 18.9 (7.9 to 40.1) | 14.7 (6.0 to 31.7) | -0.8 (-0.9 to -0.8) |
| Cuba | 1268.0 (1157.8 to 1405.6) | 1330.2 (1201.0 to 1486.8) | 0.2 (0.1 to 0.4) | 7.2 (3.0 to 14.6) | 7.5 (3.1 to 15.8) | 0.2 (0.1 to 0.4) |
| Cyprus | 1532.1 (1359.2 to 1772.4) | 1329.8 (1163.4 to 1591.2) | -0.5 (-0.6 to -0.3) | 8.7 (3.6 to 17.9) | 7.5 (3.1 to 15.9) | -0.5 (-0.6 to -0.3) |
| Czechia | 4003.8 (3557.5 to 4521.9) | 2941.8 (2614.9 to 3326.3) | -0.6 (-0.9 to -0.4) | 22.6 (9.3 to 47.6) | 16.6 (6.9 to 35.1) | -0.6 (-0.9 to -0.4) |
| Democratic People's Republic of Korea | 535.2 (489.3 to 598.2) | 430.1 (400.9 to 467.7) | -0.6 (-0.7 to -0.6) | 3.0 (1.2 to 6.3) | 2.4 (1.0 to 5.1) | -0.6 (-0.7 to -0.6) |
| Democratic Republic of the Congo | 707.2 (644.8 to 772.1) | 783.6 (630.0 to 1036.7) | 0.4 (0.1 to 0.6) | 3.9 (1.6 to 8.0) | 4.4 (1.7 to 10.6) | 0.4 (0.1 to 0.7) |
| Denmark | 1376.4 (1220.4 to 1615.1) | 1220.1 (1050.3 to 1510.3) | -0.3 (-0.5 to -0.2) | 7.8 (3.2 to 16.1) | 6.9 (2.8 to 14.4) | -0.3 (-0.5 to -0.2) |
| Djibouti | 718.9 (655.7 to 783.6) | 717.0 (633.4 to 824.0) | -0.2 (-0.4 to -0.1) | 4.1 (1.7 to 8.4) | 4.1 (1.7 to 8.9) | -0.2 (-0.4 to -0.1) |
| Dominica | 950.9 (876.9 to 1052.1) | 1044.7 (947.5 to 1179.8) | 0.3 (0.2 to 0.4) | 5.4 (2.2 to 11.4) | 5.9 (2.5 to 12.3) | 0.3 (0.1 to 0.4) |
| Dominican Republic | 1016.9 (941.8 to 1116.1) | 1149.7 (1059.8 to 1271.6) | 0.5 (0.3 to 0.7) | 5.8 (2.4 to 12.0) | 6.5 (2.7 to 13.5) | 0.5 (0.3 to 0.7) |
| Ecuador | 1318.3 (1205.4 to 1456.9) | 1205.4 (1094.1 to 1357.7) | -0.3 (-0.4 to -0.2) | 7.5 (3.1 to 15.5) | 6.8 (2.8 to 14.2) | -0.3 (-0.5 to -0.2) |
| Egypt | 1214.0 (1114.8 to 1333.2) | 909.6 (812.5 to 1040.7) | -0.9 (-1.0 to -0.8) | 6.9 (2.8 to 14.3) | 5.1 (2.1 to 10.6) | -0.9 (-1.0 to -0.8) |
| El Salvador | 2989.0 (2035.6 to 4844.6) | 2051.7 (1541.5 to 3002.0) | -1.3 (-1.4 to -1.2) | 16.9 (6.2 to 41.4) | 11.5 (4.4 to 26.7) | -1.3 (-1.4 to -1.3) |
| Equatorial Guinea | 739.0 (679.4 to 798.8) | 520.7 (472.6 to 577.8) | -1.2 (-1.2 to -1.2) | 4.1 (1.7 to 8.3) | 2.9 (1.2 to 6.2) | -1.2 (-1.2 to -1.2) |
| Eritrea | 3430.3 (1688.3 to 6905.3) | 2344.0 (1246.2 to 4729.8) | -1.4 (-1.6 to -1.3) | 19.4 (6.2 to 53.7) | 13.1 (4.3 to 34.4) | -1.5 (-1.6 to -1.3) |
| Estonia | 3094.1 (2779.4 to 3429.9) | 2005.3 (1795.2 to 2256.1) | -1.7 (-1.8 to -1.6) | 17.5 (7.3 to 36.8) | 11.3 (4.7 to 23.6) | -1.7 (-1.7 to -1.6) |
| Eswatini | 772.0 (713.1 to 827.7) | 727.5 (674.7 to 785.0) | -0.2 (-0.3 to 0.0) | 4.4 (1.8 to 9.0) | 4.1 (1.7 to 8.3) | -0.2 (-0.3 to -0.1) |
| Ethiopia | 1091.6 (952.2 to 1320.2) | 864.4 (675.8 to 1256.6) | -1.0 (-1.1 to -0.8) | 6.1 (2.5 to 13.6) | 4.9 (1.8 to 11.3) | -1.0 (-1.1 to -0.8) |
| Fiji | 579.1 (529.3 to 650.0) | 515.3 (464.4 to 590.8) | -0.4 (-0.5 to -0.4) | 3.3 (1.3 to 6.8) | 2.9 (1.2 to 6.0) | -0.4 (-0.5 to -0.4) |
| Finland | 1894.6 (1635.6 to 2298.1) | 1639.9 (1395.4 to 2037.5) | -0.6 (-1.1 to -0.1) | 10.7 (4.4 to 22.5) | 9.3 (3.8 to 19.4) | -0.6 (-1.1 to -0.1) |
| France | 1654.2 (1472.3 to 1903.8) | 1386.4 (1204.7 to 1658.2) | -0.5 (-0.6 to -0.4) | 9.4 (3.9 to 19.5) | 7.8 (3.2 to 16.2) | -0.5 (-0.6 to -0.4) |
| Gabon | 716.1 (657.3 to 780.0) | 588.9 (539.2 to 650.6) | -0.6 (-0.7 to -0.6) | 4.0 (1.7 to 8.2) | 3.3 (1.4 to 6.9) | -0.6 (-0.7 to -0.6) |
| Gambia | 748.1 (654.5 to 883.9) | 626.4 (555.9 to 710.9) | -0.6 (-0.7 to -0.6) | 4.2 (1.7 to 9.4) | 3.5 (1.4 to 7.5) | -0.7 (-0.7 to -0.6) |
| Georgia | 2260.8 (2031.0 to 2551.8) | 2361.3 (2117.9 to 2665.1) | 0.3 (0.2 to 0.4) | 12.8 (5.4 to 26.9) | 13.3 (5.5 to 27.9) | 0.3 (0.2 to 0.4) |
| Germany | 1396.9 (1241.1 to 1635.7) | 1231.3 (1075.5 to 1501.9) | -0.4 (-0.5 to -0.3) | 7.9 (3.2 to 16.4) | 7.0 (2.8 to 14.6) | -0.4 (-0.5 to -0.3) |
| Ghana | 615.8 (556.4 to 672.9) | 575.5 (521.1 to 640.3) | -0.2 (-0.3 to -0.1) | 3.5 (1.5 to 7.2) | 3.3 (1.3 to 6.7) | -0.2 (-0.3 to -0.1) |
| Greece | 1557.9 (1383.0 to 1807.0) | 1244.7 (1091.5 to 1472.2) | -0.7 (-0.9 to -0.6) | 8.8 (3.7 to 18.2) | 7.1 (2.9 to 14.7) | -0.7 (-0.9 to -0.6) |
| Greenland | 1443.2 (1321.8 to 1578.9) | 1048.2 (951.8 to 1179.3) | -1.2 (-1.4 to -1.1) | 8.1 (3.4 to 16.5) | 5.9 (2.5 to 11.9) | -1.2 (-1.4 to -1.1) |
| Grenada | 1456.7 (1260.6 to 1785.3) | 1435.9 (1284.7 to 1641.3) | 0.0 (-0.1 to 0.2) | 8.3 (3.4 to 18.6) | 8.1 (3.3 to 17.5) | 0.0 (-0.1 to 0.1) |
| Guam | 642.3 (583.6 to 716.2) | 563.7 (498.6 to 639.8) | -0.4 (-0.5 to -0.4) | 3.6 (1.5 to 7.6) | 3.2 (1.3 to 6.6) | -0.4 (-0.5 to -0.4) |
| Guatemala | 2481.0 (1961.6 to 3402.4) | 2221.5 (1897.7 to 2738.0) | -0.4 (-0.4 to -0.4) | 14.0 (5.4 to 32.8) | 12.4 (5.0 to 27.9) | -0.4 (-0.4 to -0.4) |
| Guinea | 754.0 (682.6 to 819.8) | 718.2 (647.8 to 790.7) | -0.1 (-0.1 to 0.0) | 4.3 (1.7 to 8.7) | 4.1 (1.7 to 8.4) | -0.1 (-0.1 to 0.0) |
| Guinea-Bissau | 828.9 (760.7 to 899.0) | 713.4 (637.7 to 812.7) | -0.5 (-0.6 to -0.3) | 4.7 (1.9 to 9.5) | 4.0 (1.6 to 8.5) | -0.5 (-0.6 to -0.3) |
| Guyana | 1148.6 (1055.7 to 1259.0) | 1286.0 (1181.3 to 1411.8) | 0.3 (0.1 to 0.4) | 6.4 (2.7 to 13.5) | 7.2 (3.0 to 14.8) | 0.2 (0.1 to 0.4) |
| Haiti | 1422.3 (1304.3 to 1569.4) | 1598.1 (1346.9 to 2022.1) | 0.7 (0.5 to 0.9) | 8.0 (3.3 to 16.3) | 9.0 (3.5 to 18.6) | 0.7 (0.5 to 0.9) |
| Honduras | 1704.2 (1529.7 to 1913.7) | 1425.8 (1278.2 to 1615.9) | -0.6 (-0.8 to -0.5) | 9.6 (4.0 to 20.1) | 8.0 (3.3 to 16.9) | -0.6 (-0.8 to -0.5) |
| Hungary | 3212.2 (2894.3 to 3599.5) | 2579.7 (2297.8 to 2911.0) | -0.7 (-0.8 to -0.6) | 18.1 (7.6 to 38.3) | 14.6 (6.1 to 30.4) | -0.7 (-0.8 to -0.6) |
| Iceland | 1538.8 (1363.7 to 1788.7) | 1319.4 (1135.8 to 1574.0) | -0.5 (-0.5 to -0.4) | 8.7 (3.7 to 18.1) | 7.5 (3.1 to 15.8) | -0.5 (-0.5 to -0.4) |
| India | 1017.0 (895.6 to 1152.6) | 783.5 (687.9 to 893.6) | -1.0 (-1.1 to -0.9) | 5.7 (2.3 to 11.7) | 4.4 (1.8 to 9.1) | -1.0 (-1.1 to -0.9) |
| Indonesia | 982.8 (882.1 to 1157.8) | 680.5 (608.9 to 788.7) | -1.2 (-1.2 to -1.1) | 5.6 (2.3 to 12.0) | 3.9 (1.6 to 8.2) | -1.1 (-1.2 to -1.1) |
| Iran (Islamic Republic of) | 1948.2 (1667.5 to 2429.1) | 1294.8 (1124.0 to 1534.9) | -1.4 (-1.5 to -1.3) | 11.0 (4.4 to 25.1) | 7.3 (2.9 to 15.8) | -1.4 (-1.5 to -1.3) |
| Iraq | 3523.5 (2633.1 to 5083.9) | 3364.3 (2245.1 to 5466.2) | 0.1 (0.0 to 0.2) | 19.8 (7.4 to 47.1) | 18.7 (6.7 to 46.3) | 0.0 (-0.1 to 0.1) |
| Ireland | 1477.8 (1311.2 to 1717.6) | 1309.5 (1132.1 to 1578.7) | -0.4 (-0.5 to -0.3) | 8.4 (3.5 to 17.3) | 7.4 (3.0 to 15.6) | -0.4 (-0.5 to -0.3) |
| Israel | 1430.6 (1274.3 to 1669.5) | 1311.5 (1106.5 to 1575.1) | -0.1 (-0.3 to 0.0) | 8.1 (3.3 to 16.9) | 7.4 (3.0 to 16.5) | -0.1 (-0.3 to 0.0) |
| Italy | 1714.2 (1501.5 to 1993.0) | 1315.8 (1107.5 to 1655.3) | -1.0 (-1.2 to -0.9) | 9.7 (4.1 to 20.1) | 7.4 (3.0 to 15.6) | -1.0 (-1.1 to -0.9) |
| Jamaica | 1421.6 (1296.2 to 1589.4) | 1280.3 (1166.1 to 1424.0) | -0.7 (-0.8 to -0.5) | 8.1 (3.4 to 16.9) | 7.2 (3.0 to 15.0) | -0.7 (-0.8 to -0.5) |
| Japan | 1389.5 (1268.8 to 1545.7) | 1036.0 (940.9 to 1158.8) | -1.0 (-1.1 to -0.9) | 7.9 (3.3 to 16.4) | 5.9 (2.4 to 12.3) | -1.0 (-1.1 to -0.8) |
| Jordan | 1080.0 (982.6 to 1196.0) | 847.0 (752.2 to 956.0) | -0.8 (-0.9 to -0.7) | 6.1 (2.5 to 12.7) | 4.8 (1.9 to 9.8) | -0.8 (-0.9 to -0.7) |
| Kazakhstan | 2289.0 (2070.5 to 2570.3) | 1838.6 (1667.4 to 2056.2) | -0.8 (-0.8 to -0.7) | 13.0 (5.5 to 26.7) | 10.4 (4.3 to 21.8) | -0.8 (-0.8 to -0.7) |
| Kenya | 709.5 (648.5 to 771.2) | 631.7 (574.4 to 690.3) | -0.5 (-0.7 to -0.4) | 4.0 (1.7 to 8.2) | 3.6 (1.5 to 7.4) | -0.5 (-0.7 to -0.3) |
| Kiribati | 504.8 (466.2 to 551.5) | 432.8 (391.3 to 484.4) | -0.5 (-0.6 to -0.3) | 2.8 (1.2 to 6.0) | 2.4 (1.0 to 5.1) | -0.4 (-0.6 to -0.3) |
| Kuwait | 1269.0 (1138.0 to 1428.4) | 1187.9 (1014.9 to 1425.2) | -0.6 (-0.8 to -0.4) | 7.2 (2.9 to 15.1) | 6.7 (2.7 to 14.6) | -0.6 (-0.8 to -0.4) |
| Kyrgyzstan | 1871.0 (1676.3 to 2118.2) | 1311.4 (1188.0 to 1460.6) | -1.2 (-1.3 to -1.2) | 10.6 (4.4 to 22.0) | 7.4 (3.1 to 15.6) | -1.2 (-1.3 to -1.2) |
| Lao People's Democratic Republic | 881.2 (817.6 to 947.7) | 650.0 (595.5 to 716.7) | -1.1 (-1.1 to -1.0) | 5.0 (2.0 to 10.3) | 3.7 (1.5 to 7.7) | -1.1 (-1.1 to -1.0) |
| Latvia | 3254.6 (2922.9 to 3650.8) | 2064.0 (1861.1 to 2294.1) | -1.8 (-1.9 to -1.7) | 18.3 (7.8 to 38.2) | 11.6 (4.8 to 24.0) | -1.8 (-1.9 to -1.7) |
| Lebanon | 2732.3 (1606.1 to 5060.3) | 1635.4 (1085.1 to 2728.1) | -1.7 (-1.8 to -1.6) | 15.4 (5.3 to 38.6) | 9.1 (3.3 to 21.8) | -1.7 (-1.8 to -1.7) |
| Lesotho | 673.5 (620.9 to 731.2) | 783.1 (724.9 to 844.4) | 0.6 (0.5 to 0.7) | 3.8 (1.6 to 7.8) | 4.4 (1.8 to 9.0) | 0.5 (0.4 to 0.7) |
| Liberia | 792.0 (700.3 to 907.7) | 1163.7 (781.3 to 1865.2) | 0.7 (0.2 to 1.2) | 4.4 (1.8 to 9.4) | 6.5 (2.4 to 16.2) | 0.7 (0.2 to 1.2) |
| Libya | 1340.4 (1215.9 to 1513.8) | 1689.3 (1336.5 to 2402.5) | 1.0 (0.8 to 1.2) | 7.6 (3.1 to 16.1) | 9.5 (3.7 to 22.9) | 1.0 (0.7 to 1.2) |
| Lithuania | 2875.5 (2599.4 to 3208.9) | 2022.0 (1817.3 to 2274.4) | -1.3 (-1.4 to -1.1) | 16.2 (6.8 to 33.8) | 11.4 (4.7 to 23.6) | -1.3 (-1.4 to -1.1) |
| Luxembourg | 1751.1 (1551.0 to 2017.7) | 1381.3 (1198.1 to 1644.0) | -0.8 (-0.9 to -0.8) | 9.9 (4.1 to 20.4) | 7.8 (3.3 to 16.4) | -0.8 (-0.9 to -0.8) |
| Madagascar | 732.5 (669.7 to 796.2) | 572.3 (523.8 to 625.2) | -0.8 (-0.8 to -0.7) | 4.1 (1.7 to 8.5) | 3.2 (1.3 to 6.8) | -0.7 (-0.8 to -0.7) |
| Malawi | 669.0 (605.6 to 728.7) | 545.5 (495.1 to 604.4) | -0.7 (-0.8 to -0.6) | 3.8 (1.5 to 7.8) | 3.1 (1.3 to 6.4) | -0.7 (-0.8 to -0.6) |
| Malaysia | 720.6 (666.4 to 790.0) | 631.8 (578.0 to 700.1) | -0.5 (-0.5 to -0.4) | 4.1 (1.7 to 8.4) | 3.6 (1.5 to 7.3) | -0.5 (-0.5 to -0.4) |
| Maldives | 955.4 (873.5 to 1055.9) | 750.5 (673.5 to 843.6) | -0.7 (-0.8 to -0.6) | 5.4 (2.2 to 11.2) | 4.3 (1.8 to 8.9) | -0.7 (-0.8 to -0.6) |
| Mali | 768.1 (694.4 to 831.8) | 976.3 (779.9 to 1294.9) | 0.9 (0.7 to 1.2) | 4.3 (1.8 to 8.8) | 5.5 (2.1 to 13.2) | 0.9 (0.7 to 1.2) |
| Malta | 1504.8 (1312.6 to 1793.3) | 1412.1 (1213.4 to 1715.1) | -0.1 (-0.3 to 0.1) | 8.5 (3.5 to 18.1) | 8.0 (3.3 to 16.8) | -0.1 (-0.3 to 0.1) |
| Marshall Islands | 659.2 (607.4 to 720.6) | 584.1 (531.8 to 657.2) | -0.4 (-0.5 to -0.4) | 3.7 (1.5 to 7.8) | 3.3 (1.4 to 6.8) | -0.4 (-0.5 to -0.4) |
| Mauritania | 716.2 (657.4 to 773.5) | 588.9 (536.7 to 652.6) | -0.7 (-0.7 to -0.6) | 4.0 (1.7 to 8.3) | 3.3 (1.4 to 7.0) | -0.7 (-0.7 to -0.6) |
| Mauritius | 680.2 (621.6 to 750.1) | 591.9 (531.7 to 662.7) | -0.3 (-0.4 to -0.2) | 3.8 (1.6 to 8.0) | 3.3 (1.4 to 6.9) | -0.3 (-0.4 to -0.2) |
| Mexico | 2305.8 (2061.9 to 2617.5) | 1424.8 (1268.9 to 1609.7) | -0.1 (-0.7 to 0.5) | 13.0 (5.4 to 27.1) | 8.0 (3.3 to 16.6) | -0.1 (-0.7 to 0.5) |
| Micronesia (Federated States of) | 672.0 (618.3 to 736.9) | 639.0 (578.8 to 729.1) | -0.1 (-0.2 to -0.1) | 3.8 (1.6 to 7.9) | 3.6 (1.5 to 7.4) | -0.1 (-0.2 to -0.1) |
| Monaco | 1282.0 (1119.9 to 1531.2) | 1241.8 (1075.4 to 1484.7) | 0.0 (-0.2 to 0.1) | 7.3 (3.0 to 15.1) | 7.0 (2.9 to 14.8) | 0.0 (-0.2 to 0.1) |
| Mongolia | 1953.3 (1788.0 to 2167.9) | 1981.3 (1797.6 to 2213.4) | 0.2 (0.1 to 0.3) | 11.1 (4.6 to 23.1) | 11.2 (4.7 to 23.2) | 0.2 (0.1 to 0.3) |
| Montenegro | 3157.4 (2817.8 to 3530.7) | 2716.2 (2420.9 to 3069.5) | -0.5 (-0.6 to -0.4) | 17.9 (7.5 to 37.1) | 15.4 (6.3 to 32.4) | -0.5 (-0.6 to -0.4) |
| Morocco | 1443.1 (1326.9 to 1583.5) | 1077.6 (974.4 to 1185.5) | -1.1 (-1.2 to -1.0) | 8.1 (3.3 to 16.9) | 6.0 (2.5 to 12.4) | -1.1 (-1.2 to -1.0) |
| Mozambique | 1562.1 (943.1 to 2865.3) | 1096.1 (760.7 to 1803.5) | -1.2 (-1.3 to -1.1) | 8.8 (3.1 to 22.2) | 6.1 (2.3 to 14.5) | -1.2 (-1.3 to -1.2) |
| Myanmar | 1240.2 (1029.1 to 1757.5) | 1120.1 (897.5 to 1512.6) | -0.4 (-0.5 to -0.3) | 7.0 (2.7 to 16.2) | 6.4 (2.5 to 14.2) | -0.3 (-0.4 to -0.2) |
| Namibia | 1150.3 (855.0 to 1695.3) | 893.4 (728.3 to 1161.9) | -0.9 (-1.0 to -0.8) | 6.5 (2.4 to 15.9) | 5.0 (2.0 to 12.0) | -0.9 (-1.0 to -0.8) |
| Nauru | 728.4 (671.0 to 806.7) | 690.5 (626.3 to 769.1) | -0.3 (-0.4 to -0.2) | 4.1 (1.7 to 8.5) | 3.9 (1.6 to 7.9) | -0.3 (-0.4 to -0.2) |
| Nepal | 919.4 (808.8 to 1053.1) | 1034.9 (854.1 to 1278.4) | 0.6 (0.4 to 0.7) | 5.1 (2.1 to 10.7) | 5.8 (2.3 to 13.0) | 0.6 (0.4 to 0.8) |
| Netherlands | 1123.0 (1004.9 to 1282.3) | 1025.0 (908.6 to 1189.1) | -0.3 (-0.5 to 0.0) | 6.4 (2.6 to 13.2) | 5.8 (2.4 to 12.1) | -0.3 (-0.5 to 0.0) |
| New Zealand | 3206.0 (2868.1 to 3620.5) | 2814.5 (2484.4 to 3278.2) | -0.5 (-0.7 to -0.3) | 18.0 (7.5 to 38.1) | 15.9 (6.6 to 33.4) | -0.5 (-0.7 to -0.3) |
| Nicaragua | 2778.2 (1828.9 to 4642.9) | 1889.8 (1394.0 to 2778.2) | -1.2 (-1.3 to -1.2) | 15.8 (5.8 to 38.9) | 10.6 (4.0 to 24.5) | -1.3 (-1.3 to -1.2) |
| Niger | 764.3 (689.5 to 845.4) | 702.0 (624.6 to 793.0) | -0.4 (-0.4 to -0.3) | 4.3 (1.8 to 8.9) | 4.0 (1.6 to 8.4) | -0.3 (-0.4 to -0.3) |
| Nigeria | 736.0 (667.6 to 802.5) | 624.1 (560.3 to 701.7) | -0.6 (-0.7 to -0.5) | 4.1 (1.7 to 8.5) | 3.5 (1.4 to 7.4) | -0.6 (-0.7 to -0.5) |
| Niue | 637.6 (581.7 to 711.4) | 603.0 (538.5 to 692.0) | -0.2 (-0.3 to -0.1) | 3.6 (1.5 to 7.4) | 3.4 (1.4 to 7.1) | -0.2 (-0.3 to -0.1) |
| North Macedonia | 3049.5 (2740.3 to 3410.4) | 2671.6 (2387.4 to 3011.4) | -0.4 (-0.6 to -0.3) | 17.3 (7.2 to 36.1) | 15.1 (6.2 to 31.8) | -0.4 (-0.6 to -0.3) |
| Northern Mariana Islands | 899.9 (824.0 to 996.1) | 755.4 (675.2 to 853.1) | -0.7 (-0.7 to -0.6) | 5.1 (2.1 to 10.5) | 4.3 (1.7 to 8.7) | -0.7 (-0.7 to -0.6) |
| Norway | 1566.9 (1395.9 to 1796.0) | 1185.8 (1042.8 to 1386.0) | -0.8 (-1.0 to -0.7) | 8.8 (3.7 to 18.4) | 6.7 (2.7 to 14.0) | -0.8 (-1.0 to -0.7) |
| Oman | 1646.3 (1523.6 to 1785.9) | 1197.2 (1083.2 to 1339.6) | -1.1 (-1.2 to -1.0) | 9.3 (3.8 to 19.0) | 6.7 (2.8 to 14.0) | -1.1 (-1.2 to -1.0) |
| Pakistan | 603.4 (553.2 to 674.8) | 640.6 (546.5 to 792.2) | 0.3 (0.2 to 0.4) | 3.4 (1.4 to 7.0) | 3.6 (1.4 to 8.2) | 0.3 (0.2 to 0.4) |
| Palau | 941.1 (850.8 to 1056.2) | 941.5 (842.2 to 1074.1) | 0.0 (0.0 to 0.1) | 5.3 (2.2 to 10.9) | 5.3 (2.2 to 10.9) | 0.0 (0.0 to 0.0) |
| Palestine | 2199.1 (1528.6 to 3381.8) | 2628.5 (1605.8 to 4614.3) | 1.2 (0.9 to 1.6) | 12.5 (4.5 to 29.4) | 14.8 (5.1 to 37.1) | 1.2 (0.9 to 1.5) |
| Panama | 1593.1 (1392.4 to 1862.7) | 1375.3 (1206.8 to 1588.1) | -0.5 (-0.6 to -0.4) | 9.0 (3.6 to 19.2) | 7.8 (3.1 to 16.7) | -0.5 (-0.6 to -0.4) |
| Papua New Guinea | 601.1 (545.8 to 676.7) | 701.4 (618.3 to 802.0) | 0.4 (0.3 to 0.6) | 3.4 (1.4 to 7.1) | 3.9 (1.6 to 8.4) | 0.4 (0.3 to 0.6) |
| Paraguay | 1379.7 (1213.2 to 1587.7) | 1183.8 (1048.7 to 1364.1) | -0.5 (-0.6 to -0.4) | 7.8 (3.2 to 16.3) | 6.7 (2.8 to 13.8) | -0.6 (-0.6 to -0.5) |
| Peru | 1465.0 (1282.3 to 1757.0) | 1270.0 (1108.9 to 1498.6) | -0.6 (-0.6 to -0.5) | 8.3 (3.3 to 18.5) | 7.2 (2.9 to 15.5) | -0.6 (-0.6 to -0.5) |
| Philippines | 1031.0 (867.5 to 1338.8) | 787.9 (655.9 to 1019.9) | -0.8 (-0.9 to -0.7) | 5.8 (2.3 to 13.3) | 4.5 (1.7 to 10.2) | -0.8 (-0.9 to -0.7) |
| Poland | 3241.2 (2924.4 to 3621.5) | 2414.8 (2172.9 to 2721.8) | -1.0 (-1.1 to -0.9) | 18.2 (7.6 to 38.1) | 13.6 (5.6 to 28.4) | -0.9 (-1.0 to -0.8) |
| Portugal | 1690.0 (1515.3 to 1923.0) | 987.7 (876.1 to 1139.7) | -1.9 (-2.0 to -1.9) | 9.6 (3.9 to 20.1) | 5.6 (2.3 to 11.7) | -1.9 (-2.0 to -1.9) |
| Puerto Rico | 1217.3 (1097.0 to 1356.9) | 1254.2 (1120.6 to 1415.3) | 0.1 (-0.1 to 0.2) | 6.9 (2.9 to 14.1) | 7.1 (2.9 to 14.8) | 0.1 (-0.1 to 0.2) |
| Qatar | 1550.9 (1405.9 to 1726.8) | 1268.8 (1123.2 to 1431.1) | -0.6 (-0.7 to -0.4) | 8.8 (3.7 to 18.0) | 7.1 (3.0 to 14.7) | -0.6 (-0.7 to -0.4) |
| Republic of Korea | 1888.1 (1708.7 to 2113.2) | 1188.0 (1056.8 to 1355.9) | -1.8 (-2.0 to -1.7) | 10.7 (4.5 to 21.9) | 6.7 (2.8 to 13.9) | -1.8 (-2.0 to -1.7) |
| Republic of Moldova | 2721.7 (2462.4 to 3032.8) | 1755.7 (1576.6 to 1956.2) | -1.6 (-1.7 to -1.6) | 15.4 (6.5 to 32.1) | 9.9 (4.2 to 20.5) | -1.6 (-1.7 to -1.5) |
| Romania | 3834.0 (3462.8 to 4306.2) | 2832.4 (2545.2 to 3184.2) | -1.1 (-1.2 to -1.1) | 21.6 (9.0 to 45.1) | 16.0 (6.6 to 33.2) | -1.1 (-1.1 to -1.1) |
| Russian Federation | 3269.7 (2951.2 to 3633.7) | 2241.7 (2011.5 to 2521.3) | -1.6 (-1.8 to -1.3) | 18.4 (7.7 to 38.3) | 12.6 (5.3 to 25.8) | -1.6 (-1.8 to -1.3) |
| Rwanda | 759.6 (690.1 to 827.0) | 1436.9 (895.1 to 2371.7) | 0.9 (-0.2 to 2.0) | 4.3 (1.8 to 8.8) | 8.1 (2.9 to 19.6) | 0.9 (-0.2 to 2.0) |
| Saint Kitts and Nevis | 1385.7 (1263.0 to 1534.6) | 1525.8 (1371.4 to 1717.2) | 0.5 (0.3 to 0.7) | 7.8 (3.3 to 16.1) | 8.6 (3.6 to 17.9) | 0.5 (0.3 to 0.7) |
| Saint Lucia | 1098.4 (1008.8 to 1215.9) | 1187.2 (1083.3 to 1333.6) | 0.3 (0.1 to 0.4) | 6.2 (2.6 to 12.9) | 6.7 (2.8 to 13.8) | 0.3 (0.1 to 0.4) |
| Saint Vincent and the Grenadines | 1150.2 (1057.3 to 1277.0) | 1237.4 (1130.7 to 1378.0) | 0.2 (0.1 to 0.4) | 6.5 (2.7 to 13.4) | 7.0 (2.9 to 14.4) | 0.2 (0.0 to 0.3) |
| Samoa | 620.7 (568.6 to 694.3) | 637.5 (561.4 to 758.7) | 0.2 (0.1 to 0.3) | 3.5 (1.4 to 7.2) | 3.6 (1.5 to 7.5) | 0.2 (0.1 to 0.3) |
| San Marino | 1381.2 (1210.2 to 1621.5) | 1291.6 (1125.7 to 1540.4) | -0.2 (-0.3 to -0.1) | 7.8 (3.2 to 16.3) | 7.3 (3.0 to 15.3) | -0.2 (-0.3 to -0.1) |
| Sao Tome and Principe | 819.5 (718.3 to 957.3) | 763.3 (680.3 to 862.6) | -0.2 (-0.3 to -0.2) | 4.6 (1.9 to 9.8) | 4.3 (1.8 to 9.2) | -0.2 (-0.3 to -0.2) |
| Saudi Arabia | 2316.2 (2075.2 to 2570.6) | 2170.3 (1906.9 to 2500.8) | 0.0 (-0.3 to 0.3) | 13.0 (5.5 to 27.0) | 12.1 (5.1 to 25.1) | 0.0 (-0.3 to 0.3) |
| Senegal | 665.8 (601.8 to 728.5) | 597.4 (538.7 to 663.7) | -0.4 (-0.5 to -0.3) | 3.8 (1.5 to 7.7) | 3.4 (1.4 to 7.1) | -0.4 (-0.4 to -0.3) |
| Serbia | 3095.0 (2793.2 to 3458.5) | 2734.7 (2422.9 to 3110.6) | -0.3 (-0.5 to -0.2) | 17.5 (7.2 to 36.7) | 15.5 (6.3 to 33.1) | -0.3 (-0.5 to -0.2) |
| Seychelles | 852.7 (776.9 to 944.1) | 667.4 (607.8 to 738.4) | -0.8 (-0.8 to -0.7) | 4.8 (2.0 to 10.2) | 3.8 (1.5 to 8.0) | -0.8 (-0.8 to -0.7) |
| Sierra Leone | 705.5 (640.9 to 771.6) | 1026.9 (746.0 to 1521.7) | 0.7 (0.1 to 1.3) | 4.0 (1.6 to 8.2) | 5.8 (2.1 to 14.1) | 0.7 (0.1 to 1.3) |
| Singapore | 1402.7 (1270.2 to 1572.1) | 1222.2 (1093.0 to 1382.2) | -0.5 (-0.6 to -0.4) | 8.0 (3.4 to 16.6) | 7.0 (2.9 to 14.7) | -0.5 (-0.6 to -0.4) |
| Slovakia | 3598.3 (3233.9 to 4046.1) | 3002.9 (2676.7 to 3409.3) | -0.6 (-0.6 to -0.6) | 20.3 (8.5 to 42.4) | 17.0 (7.0 to 35.6) | -0.6 (-0.6 to -0.5) |
| Slovenia | 4296.5 (3870.7 to 4810.9) | 3367.3 (2989.0 to 3795.7) | -0.4 (-0.7 to 0.0) | 24.2 (10.0 to 51.2) | 19.0 (7.8 to 40.3) | -0.4 (-0.7 to 0.0) |
| Solomon Islands | 786.6 (722.7 to 871.4) | 824.0 (733.9 to 952.7) | 0.1 (0.1 to 0.1) | 4.4 (1.9 to 9.2) | 4.6 (2.0 to 9.6) | 0.1 (0.1 to 0.1) |
| Somalia | 948.2 (750.1 to 1634.0) | 1221.7 (871.9 to 1949.0) | 0.5 (0.4 to 0.6) | 5.3 (2.0 to 12.3) | 6.9 (2.5 to 16.6) | 0.5 (0.4 to 0.6) |
| South Africa | 1140.2 (1038.7 to 1277.9) | 743.4 (677.5 to 835.8) | -1.5 (-1.7 to -1.4) | 6.4 (2.7 to 13.3) | 4.2 (1.7 to 8.6) | -1.6 (-1.7 to -1.4) |
| South Sudan | 990.0 (806.6 to 1302.7) | 1283.6 (882.5 to 2027.0) | 1.1 (0.9 to 1.3) | 5.6 (2.1 to 13.5) | 7.2 (2.6 to 18.2) | 1.1 (0.9 to 1.3) |
| Spain | 1430.9 (1289.7 to 1617.3) | 1215.1 (1044.3 to 1455.6) | -0.5 (-0.6 to -0.4) | 8.1 (3.4 to 16.8) | 6.9 (2.8 to 14.5) | -0.5 (-0.6 to -0.4) |
| Sri Lanka | 1289.9 (1058.6 to 1697.1) | 1783.2 (1197.2 to 2885.4) | 0.9 (0.6 to 1.1) | 7.3 (2.9 to 17.2) | 10.1 (3.6 to 25.3) | 0.9 (0.6 to 1.1) |
| Sudan | 1310.0 (1160.2 to 1556.1) | 1350.8 (1064.8 to 1874.9) | 0.2 (0.0 to 0.4) | 7.4 (3.0 to 16.1) | 7.6 (2.9 to 18.6) | 0.2 (0.0 to 0.4) |
| Suriname | 1149.0 (976.7 to 1628.5) | 1121.9 (994.2 to 1403.5) | 0.0 (-0.1 to 0.1) | 6.5 (2.6 to 15.0) | 6.3 (2.6 to 13.7) | -0.1 (-0.2 to 0.1) |
| Sweden | 1502.0 (1324.4 to 1726.2) | 1195.2 (1043.6 to 1405.6) | -0.7 (-0.8 to -0.6) | 8.5 (3.5 to 17.6) | 6.8 (2.8 to 14.3) | -0.7 (-0.8 to -0.6) |
| Switzerland | 2098.7 (1843.6 to 2417.3) | 1524.1 (1314.3 to 1822.1) | -1.3 (-1.6 to -1.1) | 11.8 (4.9 to 24.5) | 8.6 (3.6 to 18.1) | -1.3 (-1.5 to -1.1) |
| Syrian Arab Republic | 1167.5 (980.6 to 1517.8) | 3095.5 (1713.1 to 5901.7) | 3.2 (2.3 to 4.0) | 6.6 (2.6 to 14.9) | 17.5 (5.8 to 48.0) | 3.2 (2.3 to 4.0) |
| Taiwan (Province of China) | 731.8 (653.0 to 824.3) | 417.1 (377.3 to 472.6) | -2.2 (-2.4 to -2.1) | 4.1 (1.7 to 8.5) | 2.4 (1.0 to 4.9) | -2.2 (-2.3 to -2.1) |
| Tajikistan | 1681.7 (1502.5 to 1904.2) | 1572.0 (1321.4 to 1974.4) | -0.7 (-1.0 to -0.5) | 9.5 (4.0 to 19.7) | 8.9 (3.6 to 20.7) | -0.7 (-1.0 to -0.5) |
| Thailand | 1034.5 (948.8 to 1115.6) | 823.8 (737.7 to 917.4) | -0.9 (-1.0 to -0.9) | 5.9 (2.5 to 11.9) | 4.7 (1.9 to 9.8) | -0.9 (-1.0 to -0.9) |
| Timor-Leste | 2040.6 (1227.5 to 3544.9) | 1999.7 (1096.1 to 3644.3) | -0.2 (-0.6 to 0.2) | 11.6 (4.1 to 30.6) | 11.2 (3.8 to 30.3) | -0.2 (-0.6 to 0.2) |
| Togo | 704.6 (638.2 to 770.3) | 613.3 (556.0 to 676.2) | -0.5 (-0.5 to -0.4) | 4.0 (1.7 to 8.0) | 3.5 (1.4 to 7.3) | -0.5 (-0.5 to -0.4) |
| Tokelau | 607.0 (557.4 to 672.3) | 530.2 (476.4 to 604.5) | -0.5 (-0.6 to -0.4) | 3.4 (1.4 to 7.1) | 3.0 (1.2 to 6.1) | -0.5 (-0.6 to -0.4) |
| Tonga | 549.6 (503.9 to 619.6) | 455.7 (411.6 to 523.5) | -0.6 (-0.7 to -0.5) | 3.1 (1.3 to 6.5) | 2.6 (1.1 to 5.4) | -0.6 (-0.7 to -0.5) |
| Trinidad and Tobago | 1041.8 (956.7 to 1154.2) | 1098.9 (990.3 to 1247.2) | 0.3 (0.1 to 0.4) | 5.9 (2.4 to 12.4) | 6.2 (2.5 to 13.2) | 0.3 (0.1 to 0.4) |
| Tunisia | 1540.3 (1407.3 to 1708.4) | 1201.7 (1091.8 to 1342.3) | -0.8 (-0.8 to -0.8) | 8.7 (3.6 to 18.0) | 6.8 (2.8 to 13.9) | -0.8 (-0.9 to -0.8) |
| Turkey | 1154.0 (1056.4 to 1278.6) | 961.9 (858.0 to 1091.4) | -0.6 (-0.7 to -0.6) | 6.5 (2.8 to 13.5) | 5.4 (2.2 to 11.6) | -0.6 (-0.7 to -0.6) |
| Turkmenistan | 1698.9 (1524.8 to 1932.4) | 1522.4 (1377.5 to 1708.8) | -0.3 (-0.4 to -0.3) | 9.7 (4.0 to 20.0) | 8.7 (3.6 to 18.3) | -0.3 (-0.4 to -0.3) |
| Tuvalu | 655.5 (605.9 to 719.6) | 577.3 (523.5 to 648.9) | -1.1 (-1.4 to -0.8) | 3.7 (1.5 to 7.7) | 3.3 (1.3 to 6.7) | -1.1 (-1.4 to -0.8) |
| Uganda | 1402.7 (887.6 to 2498.2) | 1022.4 (714.4 to 1671.0) | -1.0 (-1.1 to -0.9) | 7.9 (2.9 to 19.9) | 5.7 (2.2 to 13.8) | -1.0 (-1.2 to -0.9) |
| Ukraine | 2834.0 (2521.5 to 3215.7) | 2352.9 (2110.3 to 2641.9) | -0.9 (-1.1 to -0.8) | 16.0 (6.7 to 33.0) | 13.2 (5.6 to 27.4) | -0.9 (-1.1 to -0.8) |
| United Arab Emirates | 1499.0 (1364.7 to 1649.0) | 1318.6 (1178.1 to 1518.2) | -0.4 (-0.5 to -0.4) | 8.5 (3.6 to 17.5) | 7.4 (3.1 to 15.1) | -0.4 (-0.5 to -0.4) |
| United Kingdom | 1305.3 (1156.6 to 1505.3) | 1071.0 (934.8 to 1243.9) | -0.6 (-0.8 to -0.5) | 7.4 (3.0 to 15.3) | 6.0 (2.4 to 12.8) | -0.6 (-0.8 to -0.5) |
| United Republic of Tanzania | 714.2 (644.5 to 780.1) | 620.5 (559.0 to 683.1) | -0.5 (-0.5 to -0.4) | 4.0 (1.7 to 8.3) | 3.5 (1.4 to 7.3) | -0.4 (-0.5 to -0.4) |
| United States of America | 1297.3 (1189.2 to 1435.9) | 850.8 (787.8 to 925.3) | -1.6 (-1.9 to -1.2) | 7.3 (3.1 to 14.9) | 4.8 (2.0 to 9.7) | -1.6 (-2.0 to -1.2) |
| United States Virgin Islands | 1195.2 (1101.6 to 1321.9) | 1074.7 (973.7 to 1207.7) | -0.6 (-0.8 to -0.4) | 6.8 (2.9 to 14.2) | 6.1 (2.5 to 12.8) | -0.6 (-0.8 to -0.4) |
| Uruguay | 2421.3 (2166.4 to 2739.8) | 1917.9 (1726.0 to 2164.7) | -0.9 (-1.0 to -0.9) | 13.7 (5.7 to 28.4) | 10.8 (4.5 to 22.4) | -0.9 (-1.0 to -0.9) |
| Uzbekistan | 1684.4 (1511.9 to 1903.8) | 1365.0 (1234.8 to 1518.5) | -0.8 (-0.9 to -0.7) | 9.6 (4.0 to 19.8) | 7.7 (3.2 to 16.1) | -0.8 (-0.9 to -0.7) |
| Vanuatu | 615.3 (565.4 to 695.5) | 560.8 (506.2 to 647.0) | -0.3 (-0.4 to -0.3) | 3.5 (1.4 to 7.2) | 3.2 (1.3 to 6.5) | -0.3 (-0.4 to -0.3) |
| Venezuela (Bolivarian Republic of) | 2013.2 (1786.4 to 2308.0) | 1881.4 (1680.1 to 2113.7) | -0.2 (-0.3 to -0.1) | 11.4 (4.6 to 24.0) | 10.6 (4.4 to 22.3) | -0.2 (-0.3 to -0.1) |
| Viet Nam | 888.3 (817.2 to 971.3) | 803.5 (730.7 to 888.0) | -0.2 (-0.3 to -0.2) | 5.0 (2.1 to 10.4) | 4.6 (1.9 to 9.4) | -0.2 (-0.3 to -0.1) |
| Yemen | 1316.8 (1174.8 to 1554.0) | 1650.7 (1193.7 to 2515.8) | 0.3 (0.1 to 0.6) | 7.4 (3.1 to 16.3) | 9.3 (3.5 to 22.6) | 0.3 (0.1 to 0.6) |
| Zambia | 690.9 (628.3 to 751.7) | 605.9 (548.4 to 671.4) | -0.5 (-0.6 to -0.4) | 3.9 (1.6 to 7.9) | 3.4 (1.4 to 7.0) | -0.5 (-0.6 to -0.4) |
| Zimbabwe | 644.5 (593.3 to 698.1) | 589.9 (544.2 to 641.3) | -0.3 (-0.4 to -0.2) | 3.6 (1.5 to 7.5) | 3.3 (1.4 to 6.9) | -0.3 (-0.4 to -0.3) |

**Table S17. ASR of prevalence and YLDs in 1990 and 2021, and their EAPC from 1990 to 2021 for AMP-UL1, by country.**

| **Countries** | **Prevalence** | | | **YLDs** | | |
| --- | --- | --- | --- | --- | --- | --- |
|  | **Age-standardized prevalence rate, 1990** | **Age-standardized prevalence rate, 2021** | **EAPC of prevalence rate No. (95% CI)** | **Age-standardized YLDs rate, 1990** | **Age-standardized YLDs rate, 2021** | **EAPC of YLDs rate No. (95% CI)** |
| Afghanistan | 662.1 (173.4 to 2144.8) | 591.9 (198.7 to 1595.2) | -0.6 (-0.6 to -0.5) | 61.5 (15.3 to 198.1) | 46.5 (15.9 to 129.5) | -1.1 (-1.2 to -1.0) |
| Albania | 234.3 (213.1 to 262.0) | 196.9 (179.4 to 221.8) | -0.9 (-1.1 to -0.7) | 15.2 (10.2 to 21.6) | 8.1 (5.2 to 12.2) | -2.5 (-2.7 to -2.3) |
| Algeria | 114.6 (106.7 to 125.4) | 88.1 (77.8 to 104.5) | -0.9 (-1.0 to -0.8) | 8.2 (5.6 to 11.2) | 4.5 (2.9 to 6.5) | -2.0 (-2.1 to -1.9) |
| American Samoa | 59.4 (55.4 to 63.7) | 65.9 (56.1 to 86.2) | 0.7 (0.5 to 0.9) | 4.2 (2.9 to 5.9) | 4.2 (2.7 to 6.1) | 0.4 (0.2 to 0.7) |
| Andorra | 85.3 (76.4 to 97.7) | 80.7 (71.7 to 92.7) | -0.2 (-0.3 to -0.1) | 3.2 (1.9 to 4.9) | 3.0 (1.8 to 4.6) | -0.2 (-0.3 to -0.1) |
| Angola | 152.5 (89.5 to 282.8) | 134.1 (74.4 to 257.1) | -0.6 (-0.9 to -0.3) | 14.5 (7.6 to 28.4) | 10.5 (5.4 to 21.6) | -1.2 (-1.6 to -0.9) |
| Antigua and Barbuda | 89.2 (81.2 to 97.3) | 95.9 (86.3 to 107.9) | 0.2 (-0.1 to 0.4) | 5.7 (3.8 to 7.8) | 5.0 (3.2 to 7.1) | -0.5 (-0.7 to -0.3) |
| Argentina | 81.3 (74.7 to 88.2) | 74.9 (68.8 to 81.8) | -0.3 (-0.5 to 0.0) | 5.3 (3.6 to 7.4) | 3.8 (2.5 to 5.4) | -1.0 (-1.2 to -0.8) |
| Armenia | 234.4 (168.3 to 376.8) | 164.3 (119.8 to 243.6) | -1.7 (-2.0 to -1.5) | 14.6 (8.2 to 25.8) | 7.9 (4.6 to 12.8) | -2.7 (-2.9 to -2.5) |
| Australia | 117.6 (105.4 to 133.5) | 97.9 (86.6 to 112.0) | -0.5 (-0.6 to -0.5) | 4.3 (2.7 to 6.5) | 3.6 (2.2 to 5.5) | -0.5 (-0.6 to -0.5) |
| Austria | 98.1 (87.7 to 112.5) | 73.1 (65.9 to 84.7) | -0.9 (-1.0 to -0.8) | 3.8 (2.4 to 5.9) | 2.7 (1.6 to 4.2) | -1.0 (-1.1 to -0.9) |
| Azerbaijan | 110.5 (102.4 to 117.9) | 103.7 (89.9 to 130.9) | -0.7 (-0.8 to -0.5) | 7.9 (5.3 to 11.1) | 5.9 (3.9 to 8.5) | -1.5 (-1.7 to -1.4) |
| Bahamas | 73.6 (69.0 to 79.1) | 82.0 (74.2 to 96.2) | 0.3 (0.2 to 0.3) | 5.0 (3.4 to 6.9) | 4.7 (3.2 to 6.6) | -0.3 (-0.4 to -0.2) |
| Bahrain | 78.0 (71.9 to 86.6) | 68.2 (62.4 to 75.1) | -1.5 (-2.0 to -1.1) | 5.1 (3.4 to 7.2) | 2.8 (1.7 to 4.1) | -3.1 (-3.6 to -2.6) |
| Bangladesh | 48.8 (44.5 to 55.3) | 53.1 (44.0 to 71.8) | -0.4 (-0.6 to -0.2) | 4.3 (2.9 to 6.1) | 3.4 (2.2 to 5.2) | -1.5 (-1.7 to -1.3) |
| Barbados | 65.4 (61.6 to 70.8) | 64.7 (60.3 to 70.2) | -0.2 (-0.3 to 0.0) | 4.1 (2.7 to 5.9) | 3.3 (2.1 to 4.7) | -0.8 (-0.9 to -0.7) |
| Belarus | 177.5 (162.9 to 193.8) | 150.8 (136.7 to 165.8) | -0.7 (-0.9 to -0.4) | 9.7 (6.6 to 13.6) | 5.8 (3.6 to 8.7) | -2.0 (-2.4 to -1.6) |
| Belgium | 90.4 (81.5 to 102.5) | 76.9 (68.1 to 88.9) | -0.3 (-0.8 to 0.1) | 3.5 (2.2 to 5.2) | 2.8 (1.7 to 4.3) | -0.4 (-0.8 to 0.1) |
| Belize | 84.5 (79.1 to 91.2) | 100.0 (92.2 to 110.9) | 0.3 (0.1 to 0.5) | 6.1 (4.0 to 8.4) | 6.1 (4.3 to 8.6) | -0.3 (-0.5 to -0.1) |
| Benin | 53.4 (50.0 to 57.1) | 49.2 (46.0 to 52.9) | -0.3 (-0.4 to -0.3) | 4.7 (3.1 to 6.5) | 3.8 (2.6 to 5.2) | -0.7 (-0.8 to -0.7) |
| Bermuda | 70.3 (65.5 to 76.3) | 65.7 (60.7 to 71.7) | -0.6 (-0.8 to -0.4) | 3.9 (2.5 to 5.4) | 2.4 (1.5 to 3.7) | -1.9 (-2.0 to -1.8) |
| Bhutan | 55.2 (51.5 to 59.5) | 59.9 (54.0 to 67.0) | 0.3 (0.1 to 0.4) | 4.9 (3.2 to 6.8) | 4.0 (2.6 to 5.5) | -0.7 (-0.9 to -0.6) |
| Bolivia (Plurinational State of) | 87.9 (82.7 to 94.3) | 71.6 (66.8 to 77.2) | -0.7 (-0.8 to -0.7) | 7.9 (5.3 to 10.9) | 5.0 (3.3 to 6.9) | -1.6 (-1.6 to -1.6) |
| Bosnia and Herzegovina | 225.7 (202.9 to 256.2) | 242.8 (178.4 to 371.4) | -0.4 (-0.8 to 0.0) | 13.5 (8.9 to 19.0) | 10.0 (5.7 to 16.9) | -1.9 (-2.3 to -1.5) |
| Botswana | 56.3 (53.0 to 60.2) | 63.3 (59.0 to 67.5) | 0.3 (0.0 to 0.5) | 4.7 (3.1 to 6.5) | 4.5 (3.1 to 6.2) | -0.3 (-0.6 to 0.0) |
| Brazil | 123.0 (113.3 to 135.3) | 99.3 (90.6 to 110.3) | -0.9 (-1.0 to -0.8) | 8.9 (6.2 to 12.3) | 5.6 (3.8 to 7.9) | -1.7 (-1.7 to -1.6) |
| Brunei Darussalam | 82.7 (76.0 to 91.5) | 62.5 (56.8 to 68.7) | -1.0 (-1.0 to -0.9) | 5.5 (3.8 to 7.7) | 3.3 (2.2 to 4.7) | -1.7 (-1.8 to -1.6) |
| Bulgaria | 226.3 (207.8 to 247.8) | 185.5 (169.4 to 205.7) | -0.6 (-0.7 to -0.6) | 12.7 (8.7 to 17.8) | 8.5 (5.5 to 12.3) | -1.4 (-1.5 to -1.3) |
| Burkina Faso | 54.1 (50.6 to 58.7) | 54.9 (50.5 to 60.1) | 0.0 (-0.1 to 0.0) | 4.7 (3.2 to 6.5) | 4.5 (3.0 to 6.2) | -0.3 (-0.4 to -0.2) |
| Burundi | 52.9 (49.7 to 56.3) | 326.2 (130.5 to 713.3) | 6.1 (4.4 to 7.8) | 5.0 (3.3 to 6.9) | 27.8 (10.7 to 66.3) | 5.7 (4.0 to 7.4) |
| Cabo Verde | 59.7 (55.8 to 63.4) | 53.8 (49.7 to 57.5) | -0.3 (-0.3 to -0.2) | 4.5 (3.0 to 6.1) | 3.2 (2.1 to 4.5) | -1.2 (-1.3 to -1.1) |
| Cambodia | 224.1 (90.4 to 612.4) | 150.7 (74.5 to 367.2) | -1.3 (-1.4 to -1.2) | 20.5 (7.8 to 53.9) | 10.6 (4.9 to 24.8) | -2.2 (-2.4 to -2.1) |
| Cameroon | 50.7 (47.1 to 55.5) | 53.2 (47.8 to 61.9) | 0.0 (0.0 to 0.1) | 4.3 (2.9 to 6.0) | 4.0 (2.8 to 5.7) | -0.4 (-0.4 to -0.3) |
| Canada | 60.3 (55.9 to 66.1) | 46.6 (42.6 to 50.6) | -0.8 (-0.8 to -0.8) | 2.2 (1.4 to 3.5) | 1.7 (1.0 to 2.6) | -0.8 (-0.8 to -0.8) |
| Central African Republic | 60.6 (57.0 to 64.9) | 96.6 (71.1 to 149.1) | 1.6 (1.4 to 1.8) | 5.8 (4.0 to 8.1) | 9.1 (5.4 to 14.8) | 1.5 (1.3 to 1.8) |
| Chad | 93.3 (62.7 to 160.3) | 92.7 (64.5 to 150.1) | 0.0 (-0.2 to 0.1) | 8.6 (4.9 to 15.5) | 7.9 (4.6 to 13.4) | -0.3 (-0.5 to -0.2) |
| Chile | 78.5 (71.9 to 85.8) | 82.0 (74.2 to 92.4) | 0.3 (0.2 to 0.3) | 4.9 (3.2 to 6.8) | 3.2 (2.0 to 4.9) | -1.3 (-1.3 to -1.3) |
| China | 77.0 (72.2 to 83.6) | 66.9 (61.9 to 73.0) | -0.7 (-0.9 to -0.5) | 5.5 (3.7 to 7.6) | 2.7 (1.7 to 4.0) | -2.9 (-3.2 to -2.6) |
| Colombia | 160.4 (145.7 to 182.6) | 117.1 (100.2 to 144.6) | -1.0 (-1.1 to -1.0) | 11.5 (7.8 to 15.9) | 5.8 (3.7 to 8.5) | -2.2 (-2.3 to -2.2) |
| Comoros | 56.0 (52.6 to 59.6) | 53.0 (47.8 to 61.6) | -0.4 (-0.4 to -0.3) | 5.0 (3.4 to 6.8) | 4.1 (2.8 to 5.7) | -0.8 (-0.9 to -0.7) |
| Congo | 56.1 (52.9 to 60.2) | 97.9 (60.3 to 177.6) | 1.9 (1.0 to 2.9) | 5.1 (3.4 to 7.0) | 7.5 (4.1 to 14.7) | 1.3 (0.4 to 2.3) |
| Cook Islands | 62.3 (57.1 to 69.4) | 60.9 (52.1 to 76.3) | 0.0 (-0.2 to 0.2) | 4.0 (2.7 to 5.6) | 2.8 (1.8 to 4.3) | -1.1 (-1.3 to -0.9) |
| Costa Rica | 96.4 (88.9 to 106.3) | 89.1 (82.1 to 97.9) | -0.3 (-0.4 to -0.2) | 5.6 (3.7 to 7.9) | 4.0 (2.5 to 5.8) | -1.2 (-1.2 to -1.1) |
| Coted'Ivoire | 54.1 (50.7 to 57.8) | 53.5 (49.2 to 59.2) | -0.1 (-0.1 to 0.0) | 4.7 (3.1 to 6.5) | 4.0 (2.6 to 5.5) | -0.5 (-0.6 to -0.4) |
| Croatia | 207.0 (190.4 to 228.4) | 172.2 (151.7 to 208.8) | -0.8 (-1.0 to -0.7) | 9.5 (6.2 to 13.5) | 6.4 (3.9 to 9.8) | -1.6 (-1.7 to -1.5) |
| Cuba | 89.4 (83.8 to 96.6) | 85.6 (79.4 to 93.1) | -0.1 (-0.2 to 0.0) | 5.1 (3.4 to 7.0) | 3.7 (2.4 to 5.4) | -0.9 (-1.0 to -0.8) |
| Cyprus | 83.1 (75.6 to 94.3) | 70.3 (62.6 to 81.4) | -0.6 (-0.7 to -0.4) | 3.9 (2.6 to 5.7) | 2.6 (1.6 to 4.1) | -1.3 (-1.3 to -1.2) |
| Czechia | 243.4 (225.3 to 267.8) | 175.7 (160.2 to 196.4) | -0.8 (-1.0 to -0.6) | 12.2 (8.2 to 17.4) | 6.5 (4.0 to 10.1) | -1.5 (-1.8 to -1.3) |
| Democratic People's Republic of Korea | 59.0 (55.4 to 64.4) | 51.3 (48.0 to 55.4) | -0.4 (-0.5 to -0.4) | 4.3 (2.9 to 6.1) | 3.0 (2.0 to 4.3) | -1.1 (-1.2 to -1.1) |
| Democratic Republic of the Congo | 56.7 (53.0 to 61.5) | 74.5 (56.2 to 111.6) | 0.9 (0.6 to 1.3) | 5.1 (3.4 to 7.0) | 6.0 (3.8 to 9.6) | 0.6 (0.2 to 1.0) |
| Denmark | 74.9 (67.5 to 86.5) | 61.5 (54.3 to 73.0) | -0.6 (-0.8 to -0.5) | 3.1 (1.9 to 4.5) | 2.3 (1.4 to 3.5) | -0.8 (-0.9 to -0.7) |
| Djibouti | 53.9 (50.4 to 57.7) | 58.1 (50.8 to 70.9) | -0.1 (-0.4 to 0.1) | 4.7 (3.2 to 6.5) | 4.4 (3.0 to 6.5) | -0.6 (-0.9 to -0.3) |
| Dominica | 67.0 (62.8 to 72.3) | 77.5 (68.9 to 93.6) | 0.4 (0.3 to 0.5) | 4.8 (3.1 to 6.7) | 5.0 (3.2 to 7.3) | 0.1 (0.0 to 0.2) |
| Dominican Republic | 71.6 (67.3 to 76.9) | 81.1 (76.3 to 86.9) | 0.5 (0.3 to 0.7) | 5.6 (3.7 to 7.8) | 5.2 (3.4 to 7.3) | -0.1 (-0.2 to 0.1) |
| Ecuador | 97.2 (88.6 to 110.0) | 89.0 (82.3 to 97.5) | -0.3 (-0.4 to -0.1) | 7.6 (5.0 to 10.6) | 5.1 (3.4 to 7.3) | -1.2 (-1.4 to -1.1) |
| Egypt | 97.6 (90.4 to 106.1) | 77.7 (70.3 to 89.6) | -0.7 (-0.8 to -0.6) | 7.7 (5.0 to 10.5) | 4.5 (2.9 to 6.3) | -1.6 (-1.7 to -1.5) |
| El Salvador | 368.8 (194.7 to 745.0) | 237.6 (143.9 to 428.5) | -1.5 (-1.6 to -1.4) | 28.9 (13.9 to 61.3) | 12.9 (7.0 to 25.5) | -2.7 (-2.7 to -2.6) |
| Equatorial Guinea | 58.9 (55.5 to 63.1) | 42.5 (39.8 to 46.1) | -1.2 (-1.2 to -1.1) | 5.6 (3.8 to 7.6) | 2.8 (1.9 to 4.0) | -2.4 (-2.5 to -2.3) |
| Eritrea | 544.3 (201.6 to 1286.3) | 377.6 (144.2 to 872.5) | -1.4 (-1.6 to -1.2) | 51.7 (18.5 to 125.9) | 31.3 (12.1 to 76.8) | -1.8 (-2.0 to -1.6) |
| Estonia | 216.5 (200.2 to 234.9) | 129.0 (118.0 to 143.3) | -2.1 (-2.2 to -2.0) | 11.5 (7.6 to 16.1) | 4.8 (3.0 to 7.3) | -3.5 (-3.7 to -3.2) |
| Eswatini | 70.3 (65.7 to 75.0) | 67.7 (63.4 to 72.1) | -0.1 (-0.2 to 0.1) | 5.8 (4.1 to 8.2) | 5.1 (3.4 to 7.0) | -0.4 (-0.6 to -0.1) |
| Ethiopia | 92.5 (77.2 to 123.5) | 80.2 (55.7 to 140.9) | -0.7 (-0.9 to -0.5) | 9.0 (5.9 to 13.7) | 6.1 (3.6 to 11.6) | -1.5 (-1.8 to -1.3) |
| Fiji | 50.0 (46.5 to 54.7) | 46.0 (41.8 to 52.0) | -0.3 (-0.4 to -0.3) | 3.8 (2.6 to 5.3) | 3.3 (2.2 to 4.6) | -0.6 (-0.7 to -0.5) |
| Finland | 106.0 (95.0 to 120.7) | 88.2 (77.0 to 103.0) | -0.7 (-1.2 to -0.2) | 4.4 (2.8 to 6.6) | 3.3 (1.9 to 5.1) | -0.9 (-1.3 to -0.5) |
| France | 93.0 (84.4 to 105.6) | 74.6 (66.5 to 87.0) | -0.7 (-0.7 to -0.6) | 3.8 (2.4 to 5.7) | 2.8 (1.7 to 4.1) | -0.8 (-0.9 to -0.7) |
| Gabon | 56.8 (52.9 to 61.5) | 48.5 (45.4 to 52.5) | -0.5 (-0.6 to -0.5) | 4.8 (3.3 to 6.7) | 3.4 (2.3 to 4.6) | -1.2 (-1.3 to -1.1) |
| Gambia | 57.6 (47.9 to 76.4) | 49.1 (42.9 to 59.7) | -0.6 (-0.6 to -0.5) | 4.8 (3.1 to 7.5) | 3.7 (2.4 to 5.4) | -1.0 (-1.1 to -0.9) |
| Georgia | 139.4 (127.9 to 154.8) | 152.4 (137.0 to 175.7) | 0.4 (0.3 to 0.5) | 8.3 (5.7 to 11.3) | 8.1 (5.3 to 11.9) | 0.2 (0.0 to 0.3) |
| Germany | 75.9 (69.0 to 87.0) | 64.7 (57.8 to 74.7) | -0.6 (-0.7 to -0.5) | 3.2 (2.0 to 4.8) | 2.4 (1.5 to 3.7) | -0.8 (-0.9 to -0.7) |
| Ghana | 46.2 (43.2 to 49.5) | 43.8 (40.6 to 47.7) | -0.2 (-0.3 to -0.1) | 3.9 (2.6 to 5.5) | 3.2 (2.1 to 4.5) | -0.6 (-0.7 to -0.5) |
| Greece | 86.0 (78.2 to 97.3) | 64.9 (57.6 to 74.5) | -1.0 (-1.1 to -0.8) | 3.2 (2.0 to 4.9) | 2.4 (1.5 to 3.7) | -1.0 (-1.1 to -0.8) |
| Greenland | 93.8 (86.8 to 102.2) | 67.8 (61.5 to 74.6) | -1.3 (-1.4 to -1.2) | 6.0 (4.1 to 8.2) | 3.3 (2.1 to 4.7) | -2.2 (-2.3 to -2.1) |
| Grenada | 118.2 (88.1 to 185.2) | 101.3 (83.1 to 139.7) | -0.5 (-0.6 to -0.4) | 8.8 (5.4 to 14.7) | 6.0 (3.9 to 9.3) | -1.1 (-1.2 to -1.0) |
| Guam | 56.2 (51.9 to 61.2) | 52.1 (47.4 to 57.6) | -0.2 (-0.3 to -0.2) | 3.3 (2.2 to 4.6) | 2.8 (1.8 to 3.9) | -0.5 (-0.6 to -0.4) |
| Guatemala | 223.3 (149.1 to 390.8) | 176.0 (133.2 to 273.1) | -0.8 (-0.9 to -0.8) | 19.4 (11.4 to 36.4) | 11.6 (7.3 to 19.6) | -1.7 (-1.7 to -1.7) |
| Guinea | 54.3 (50.7 to 58.3) | 57.1 (52.5 to 62.8) | 0.3 (0.2 to 0.3) | 5.0 (3.3 to 6.9) | 4.7 (3.2 to 6.4) | 0.0 (-0.1 to 0.1) |
| Guinea-Bissau | 65.8 (61.9 to 70.1) | 62.5 (56.0 to 74.2) | -0.1 (-0.3 to 0.1) | 6.2 (4.2 to 8.5) | 5.3 (3.6 to 7.7) | -0.5 (-0.7 to -0.2) |
| Guyana | 83.4 (78.6 to 89.3) | 93.9 (87.2 to 101.0) | 0.3 (0.2 to 0.4) | 7.0 (4.7 to 9.7) | 6.8 (4.6 to 9.3) | -0.1 (-0.2 to 0.0) |
| Haiti | 96.7 (90.7 to 103.5) | 220.8 (125.8 to 426.2) | 4.0 (3.0 to 5.0) | 9.3 (6.4 to 13.0) | 18.9 (8.9 to 37.6) | 3.6 (2.6 to 4.6) |
| Honduras | 114.5 (105.5 to 125.2) | 110.8 (99.0 to 131.7) | -0.1 (-0.3 to 0.1) | 9.6 (6.5 to 13.6) | 7.8 (5.3 to 11.3) | -0.7 (-0.9 to -0.5) |
| Hungary | 211.2 (195.6 to 229.9) | 155.4 (142.1 to 172.8) | -1.1 (-1.2 to -1.0) | 11.5 (7.6 to 16.2) | 5.8 (3.5 to 8.9) | -2.3 (-2.4 to -2.2) |
| Iceland | 83.0 (74.3 to 94.1) | 68.5 (61.1 to 79.9) | -0.6 (-0.7 to -0.5) | 3.1 (1.8 to 4.7) | 2.6 (1.6 to 3.9) | -0.6 (-0.7 to -0.5) |
| India | 81.5 (75.8 to 89.2) | 68.1 (62.4 to 75.9) | -0.7 (-0.8 to -0.6) | 6.9 (4.7 to 9.4) | 4.5 (3.1 to 6.2) | -1.6 (-1.7 to -1.4) |
| Indonesia | 75.9 (67.8 to 95.5) | 57.5 (50.1 to 73.0) | -0.8 (-1.0 to -0.7) | 6.3 (4.3 to 9.0) | 3.9 (2.6 to 5.7) | -1.4 (-1.6 to -1.3) |
| Iran (Islamic Republic of) | 180.8 (144.7 to 250.7) | 119.8 (98.9 to 156.5) | -1.5 (-1.6 to -1.3) | 12.2 (7.8 to 18.7) | 5.3 (3.3 to 7.9) | -2.7 (-2.9 to -2.6) |
| Iraq | 415.1 (219.5 to 784.7) | 393.8 (199.6 to 776.9) | 0.0 (-0.1 to 0.2) | 29.7 (14.5 to 61.3) | 20.1 (9.2 to 42.6) | -1.2 (-1.3 to -1.1) |
| Ireland | 76.2 (69.4 to 87.1) | 66.3 (57.6 to 79.7) | -0.5 (-0.6 to -0.3) | 3.1 (1.9 to 4.6) | 2.5 (1.5 to 3.8) | -0.6 (-0.7 to -0.5) |
| Israel | 70.3 (63.7 to 80.3) | 71.9 (59.7 to 92.6) | 0.2 (0.1 to 0.4) | 3.2 (2.1 to 4.7) | 2.7 (1.6 to 4.2) | -0.3 (-0.4 to -0.1) |
| Italy | 93.1 (84.3 to 104.6) | 70.2 (61.7 to 81.3) | -1.1 (-1.2 to -1.0) | 3.6 (2.2 to 5.4) | 2.6 (1.6 to 4.0) | -1.1 (-1.2 to -1.0) |
| Jamaica | 85.0 (78.5 to 92.3) | 79.5 (72.9 to 87.7) | -0.5 (-0.7 to -0.4) | 5.5 (3.7 to 7.7) | 4.4 (2.9 to 6.3) | -1.0 (-1.2 to -0.9) |
| Japan | 69.5 (64.3 to 76.4) | 50.5 (46.4 to 56.5) | -1.1 (-1.2 to -0.9) | 2.6 (1.6 to 4.0) | 1.9 (1.2 to 2.9) | -1.1 (-1.2 to -1.0) |
| Jordan | 79.8 (74.8 to 86.6) | 61.4 (56.9 to 67.1) | -0.9 (-1.0 to -0.8) | 5.3 (3.5 to 7.3) | 2.8 (1.7 to 4.1) | -2.4 (-2.5 to -2.3) |
| Kazakhstan | 153.3 (142.2 to 168.3) | 123.9 (114.2 to 135.6) | -0.8 (-0.9 to -0.6) | 10.2 (7.0 to 14.0) | 6.3 (4.1 to 9.1) | -1.9 (-2.2 to -1.5) |
| Kenya | 53.6 (50.5 to 56.8) | 51.3 (47.1 to 56.0) | -0.3 (-0.4 to -0.1) | 4.3 (2.9 to 5.9) | 3.8 (2.5 to 5.2) | -0.4 (-0.6 to -0.2) |
| Kiribati | 53.5 (49.3 to 58.6) | 44.0 (39.8 to 49.7) | -0.5 (-0.6 to -0.5) | 5.0 (3.3 to 7.0) | 3.7 (2.5 to 5.2) | -0.8 (-0.9 to -0.7) |
| Kuwait | 94.6 (86.3 to 105.5) | 92.1 (75.5 to 123.2) | -0.7 (-1.0 to -0.5) | 4.7 (3.1 to 6.7) | 3.4 (2.0 to 5.4) | -2.0 (-2.3 to -1.7) |
| Kyrgyzstan | 129.8 (120.2 to 142.9) | 90.9 (84.1 to 99.8) | -1.2 (-1.3 to -1.2) | 9.1 (6.1 to 12.5) | 5.1 (3.4 to 7.2) | -2.0 (-2.0 to -1.9) |
| Lao People's Democratic Republic | 74.6 (69.7 to 79.4) | 53.9 (49.6 to 59.1) | -1.2 (-1.3 to -1.1) | 7.1 (4.7 to 9.9) | 4.1 (2.7 to 5.8) | -1.9 (-2.0 to -1.8) |
| Latvia | 231.6 (214.7 to 250.8) | 137.3 (126.1 to 149.9) | -2.1 (-2.3 to -2.0) | 12.5 (8.5 to 17.1) | 5.6 (3.6 to 8.3) | -3.0 (-3.2 to -2.8) |
| Lebanon | 373.5 (154.9 to 962.7) | 211.7 (96.0 to 511.8) | -1.9 (-1.9 to -1.8) | 23.9 (9.7 to 62.8) | 8.3 (3.5 to 20.5) | -3.5 (-3.6 to -3.4) |
| Lesotho | 59.3 (55.8 to 63.1) | 78.7 (73.2 to 84.2) | 1.1 (0.9 to 1.2) | 5.1 (3.4 to 7.1) | 6.5 (4.5 to 8.9) | 1.0 (0.7 to 1.2) |
| Liberia | 62.6 (53.5 to 79.9) | 132.9 (69.8 to 260.3) | 1.5 (0.8 to 2.3) | 5.6 (3.7 to 8.1) | 9.9 (4.8 to 20.8) | 0.9 (0.1 to 1.6) |
| Libya | 108.3 (96.4 to 128.5) | 161.5 (119.0 to 246.6) | 1.6 (1.3 to 1.9) | 7.2 (4.8 to 10.1) | 8.2 (4.7 to 14.0) | 0.7 (0.3 to 1.0) |
| Lithuania | 206.1 (191.0 to 224.4) | 137.4 (126.5 to 149.9) | -1.5 (-1.7 to -1.3) | 10.7 (7.3 to 15.0) | 5.7 (3.6 to 8.4) | -2.1 (-2.3 to -1.9) |
| Luxembourg | 94.9 (85.8 to 107.6) | 72.9 (65.1 to 85.8) | -0.9 (-1.0 to -0.9) | 4.0 (2.5 to 5.9) | 2.7 (1.6 to 4.2) | -1.1 (-1.2 to -1.0) |
| Madagascar | 51.4 (48.6 to 55.0) | 42.1 (39.1 to 45.2) | -0.6 (-0.7 to -0.6) | 4.6 (3.1 to 6.4) | 3.4 (2.3 to 4.8) | -0.9 (-1.0 to -0.9) |
| Malawi | 47.2 (44.0 to 50.9) | 40.5 (37.6 to 43.8) | -0.6 (-0.7 to -0.5) | 4.3 (2.9 to 6.0) | 3.2 (2.2 to 4.6) | -1.0 (-1.1 to -0.9) |
| Malaysia | 57.3 (53.7 to 61.4) | 51.5 (48.0 to 55.7) | -0.4 (-0.4 to -0.4) | 4.1 (2.8 to 5.7) | 2.8 (1.8 to 3.9) | -1.4 (-1.4 to -1.4) |
| Maldives | 64.6 (60.1 to 70.4) | 55.8 (50.0 to 64.3) | -0.3 (-0.4 to -0.1) | 5.2 (3.4 to 7.3) | 2.7 (1.8 to 4.1) | -2.0 (-2.1 to -1.8) |
| Mali | 58.5 (54.2 to 63.1) | 86.7 (63.7 to 134.1) | 1.4 (1.0 to 1.8) | 5.2 (3.6 to 7.3) | 6.9 (4.2 to 11.3) | 1.0 (0.6 to 1.4) |
| Malta | 76.8 (68.8 to 88.1) | 71.6 (63.2 to 84.5) | -0.1 (-0.3 to 0.1) | 3.3 (2.0 to 4.9) | 2.7 (1.6 to 4.1) | -0.3 (-0.5 to -0.1) |
| Marshall Islands | 63.3 (58.9 to 69.0) | 56.0 (51.8 to 61.4) | -0.4 (-0.4 to -0.3) | 5.4 (3.7 to 7.5) | 4.3 (2.9 to 5.9) | -0.7 (-0.8 to -0.6) |
| Mauritania | 58.5 (54.9 to 63.2) | 47.4 (44.0 to 51.4) | -0.7 (-0.8 to -0.7) | 5.1 (3.4 to 7.0) | 3.2 (2.1 to 4.5) | -1.5 (-1.6 to -1.4) |
| Mauritius | 51.8 (48.3 to 56.2) | 45.5 (42.3 to 49.4) | -0.3 (-0.4 to -0.3) | 3.4 (2.3 to 4.8) | 2.4 (1.6 to 3.4) | -0.9 (-1.0 to -0.8) |
| Mexico | 150.7 (140.1 to 164.8) | 98.0 (91.6 to 106.0) | -0.3 (-0.7 to 0.2) | 11.1 (7.6 to 15.1) | 5.5 (3.8 to 7.6) | -1.0 (-1.5 to -0.6) |
| Micronesia (Federated States of) | 65.7 (61.3 to 71.0) | 65.1 (59.4 to 73.4) | 0.1 (0.0 to 0.2) | 5.7 (3.8 to 7.9) | 4.8 (3.2 to 6.6) | -0.5 (-0.6 to -0.4) |
| Monaco | 66.1 (58.3 to 77.6) | 63.6 (56.7 to 73.6) | -0.1 (-0.2 to 0.1) | 2.5 (1.5 to 3.8) | 2.4 (1.4 to 3.6) | -0.1 (-0.2 to 0.0) |
| Mongolia | 132.3 (123.4 to 143.3) | 131.8 (121.5 to 143.9) | 0.1 (0.0 to 0.1) | 11.1 (7.6 to 15.3) | 8.3 (5.7 to 11.6) | -1.0 (-1.1 to -0.9) |
| Montenegro | 191.2 (175.0 to 211.1) | 162.5 (147.5 to 181.5) | -0.5 (-0.6 to -0.5) | 8.4 (5.4 to 12.3) | 6.0 (3.7 to 9.2) | -1.4 (-1.6 to -1.2) |
| Morocco | 104.5 (97.0 to 114.5) | 79.7 (73.8 to 88.1) | -1.0 (-1.1 to -0.9) | 8.2 (5.4 to 11.3) | 4.8 (3.2 to 6.6) | -1.8 (-2.0 to -1.7) |
| Mozambique | 181.3 (85.4 to 400.8) | 126.2 (69.6 to 257.9) | -1.2 (-1.3 to -1.1) | 16.8 (7.6 to 38.4) | 10.5 (5.5 to 21.7) | -1.5 (-1.6 to -1.4) |
| Myanmar | 99.2 (78.3 to 166.9) | 108.8 (77.9 to 176.5) | 0.6 (0.3 to 0.9) | 9.0 (5.7 to 15.3) | 7.8 (4.7 to 12.6) | -0.3 (-0.5 to 0.0) |
| Namibia | 114.6 (77.9 to 190.7) | 87.7 (67.4 to 129.9) | -0.9 (-1.0 to -0.8) | 9.7 (5.6 to 17.7) | 6.1 (3.8 to 9.6) | -1.6 (-1.7 to -1.4) |
| Nauru | 71.3 (65.8 to 76.7) | 67.9 (62.5 to 74.2) | -0.2 (-0.4 to -0.1) | 5.8 (4.0 to 8.0) | 4.9 (3.2 to 7.0) | -0.6 (-0.8 to -0.3) |
| Nepal | 68.2 (62.5 to 75.9) | 90.8 (72.8 to 121.6) | 1.2 (1.0 to 1.5) | 6.0 (4.1 to 8.2) | 6.3 (4.0 to 9.3) | 0.5 (0.2 to 0.7) |
| Netherlands | 58.0 (52.6 to 65.7) | 52.8 (47.3 to 60.3) | -0.3 (-0.5 to -0.1) | 2.2 (1.3 to 3.3) | 2.0 (1.2 to 3.0) | -0.3 (-0.5 to -0.1) |
| New Zealand | 139.8 (127.0 to 158.2) | 117.7 (105.3 to 135.3) | -0.7 (-0.8 to -0.5) | 5.5 (3.5 to 8.1) | 4.4 (2.7 to 6.7) | -0.8 (-1.0 to -0.6) |
| Nicaragua | 338.8 (157.4 to 721.1) | 216.6 (114.6 to 424.6) | -1.5 (-1.5 to -1.4) | 25.5 (11.7 to 56.1) | 12.4 (6.2 to 26.0) | -2.4 (-2.5 to -2.3) |
| Niger | 57.8 (53.6 to 62.4) | 55.9 (50.7 to 64.0) | -0.2 (-0.3 to -0.2) | 5.4 (3.6 to 7.6) | 4.7 (3.1 to 6.6) | -0.6 (-0.7 to -0.5) |
| Nigeria | 55.2 (51.6 to 59.2) | 51.6 (46.5 to 59.5) | -0.3 (-0.4 to -0.2) | 4.7 (3.2 to 6.4) | 3.8 (2.5 to 5.2) | -0.8 (-0.9 to -0.7) |
| Niue | 57.5 (53.5 to 62.4) | 54.7 (49.7 to 62.3) | -0.1 (-0.3 to 0.0) | 4.2 (2.8 to 5.7) | 3.3 (2.2 to 4.8) | -0.7 (-0.8 to -0.6) |
| North Macedonia | 170.5 (156.9 to 191.4) | 154.7 (141.5 to 173.7) | -0.3 (-0.5 to -0.2) | 10.4 (7.1 to 14.8) | 6.7 (4.3 to 9.7) | -1.7 (-1.8 to -1.6) |
| Northern Mariana Islands | 75.9 (71.0 to 81.6) | 65.9 (60.4 to 71.9) | -0.5 (-0.5 to -0.5) | 4.6 (3.1 to 6.4) | 3.5 (2.3 to 5.0) | -0.7 (-0.8 to -0.6) |
| Norway | 86.3 (78.6 to 96.6) | 61.2 (55.6 to 70.9) | -1.1 (-1.2 to -1.0) | 3.3 (2.1 to 5.1) | 2.3 (1.4 to 3.5) | -1.1 (-1.3 to -1.0) |
| Oman | 143.5 (134.3 to 156.6) | 97.6 (89.9 to 107.4) | -1.4 (-1.5 to -1.3) | 9.4 (6.4 to 13.1) | 4.1 (2.6 to 6.0) | -2.7 (-2.9 to -2.6) |
| Pakistan | 50.2 (47.2 to 53.9) | 63.0 (51.8 to 84.1) | 1.0 (0.9 to 1.1) | 4.3 (2.9 to 5.9) | 4.8 (3.1 to 7.1) | 0.5 (0.4 to 0.7) |
| Palau | 79.8 (73.7 to 86.4) | 78.0 (72.0 to 85.4) | -0.1 (-0.1 to 0.0) | 5.6 (3.8 to 7.8) | 4.7 (3.1 to 6.6) | -0.5 (-0.5 to -0.5) |
| Palestine | 272.7 (140.5 to 529.1) | 322.6 (149.3 to 673.4) | 1.2 (0.9 to 1.5) | 19.0 (9.2 to 39.2) | 16.5 (7.3 to 36.8) | 0.2 (-0.1 to 0.6) |
| Panama | 108.9 (96.3 to 139.7) | 92.1 (82.1 to 110.9) | -0.6 (-0.6 to -0.5) | 7.2 (4.7 to 10.7) | 4.6 (3.0 to 6.7) | -1.4 (-1.5 to -1.3) |
| Papua New Guinea | 56.9 (53.2 to 61.7) | 71.2 (63.7 to 83.9) | 0.7 (0.5 to 0.9) | 4.7 (3.2 to 6.6) | 5.6 (3.7 to 8.1) | 0.5 (0.3 to 0.7) |
| Paraguay | 87.2 (78.9 to 96.2) | 82.0 (75.7 to 90.7) | -0.3 (-0.4 to -0.2) | 6.2 (4.2 to 8.6) | 4.7 (3.2 to 6.7) | -0.9 (-1.0 to -0.8) |
| Peru | 107.5 (89.6 to 145.3) | 90.3 (75.0 to 118.4) | -0.7 (-0.8 to -0.6) | 8.4 (5.6 to 12.7) | 4.6 (2.9 to 6.9) | -2.2 (-2.3 to -2.1) |
| Philippines | 94.4 (76.5 to 132.6) | 74.7 (59.5 to 103.8) | -0.8 (-0.8 to -0.8) | 7.1 (4.6 to 11.0) | 5.1 (3.3 to 7.8) | -1.1 (-1.1 to -1.0) |
| Poland | 208.1 (192.4 to 228.5) | 151.4 (138.8 to 168.9) | -1.1 (-1.2 to -1.0) | 12.1 (8.3 to 16.7) | 5.7 (3.6 to 8.8) | -2.5 (-2.6 to -2.5) |
| Portugal | 96.6 (87.9 to 109.0) | 54.7 (48.9 to 62.3) | -2.1 (-2.2 to -2.0) | 4.8 (3.1 to 6.8) | 2.0 (1.2 to 3.1) | -2.9 (-3.0 to -2.7) |
| Puerto Rico | 86.7 (80.9 to 93.9) | 87.0 (78.9 to 101.8) | -0.1 (-0.2 to 0.0) | 4.9 (3.3 to 6.8) | 3.4 (2.1 to 5.2) | -1.4 (-1.5 to -1.3) |
| Qatar | 114.0 (105.8 to 124.9) | 88.9 (80.1 to 98.3) | -0.8 (-1.0 to -0.6) | 6.9 (4.6 to 9.9) | 3.3 (1.9 to 5.0) | -2.6 (-2.8 to -2.4) |
| Republic of Korea | 102.3 (94.2 to 113.2) | 62.7 (56.1 to 70.0) | -1.9 (-2.0 to -1.8) | 6.0 (4.0 to 8.4) | 2.3 (1.4 to 3.6) | -3.2 (-3.5 to -3.0) |
| Republic of Moldova | 185.8 (174.0 to 200.6) | 119.4 (110.6 to 130.5) | -1.6 (-1.7 to -1.6) | 11.4 (7.7 to 15.6) | 5.6 (3.6 to 8.0) | -2.5 (-2.6 to -2.3) |
| Romania | 235.5 (215.5 to 259.8) | 172.7 (159.1 to 190.7) | -1.1 (-1.2 to -1.1) | 14.7 (10.1 to 20.1) | 7.0 (4.5 to 10.5) | -2.6 (-2.6 to -2.5) |
| Russian Federation | 228.3 (213.5 to 245.7) | 166.4 (152.4 to 181.9) | -1.3 (-1.6 to -1.0) | 12.9 (8.8 to 17.7) | 6.8 (4.4 to 10.1) | -2.6 (-3.0 to -2.1) |
| Rwanda | 62.3 (58.0 to 67.3) | 359.7 (131.3 to 860.3) | 3.2 (0.9 to 5.5) | 6.0 (4.1 to 8.3) | 27.8 (9.8 to 68.0) | 2.3 (0.0 to 4.7) |
| Saint Kitts and Nevis | 91.1 (84.4 to 98.0) | 94.1 (86.0 to 102.3) | 0.3 (0.1 to 0.5) | 7.1 (4.7 to 10.0) | 5.5 (3.7 to 7.7) | -0.7 (-0.7 to -0.6) |
| Saint Lucia | 75.9 (71.3 to 81.6) | 78.1 (73.1 to 84.1) | 0.1 (0.0 to 0.2) | 5.5 (3.7 to 7.7) | 4.5 (3.0 to 6.3) | -0.7 (-0.7 to -0.6) |
| Saint Vincent and the Grenadines | 76.0 (71.1 to 81.8) | 80.4 (74.8 to 86.6) | 0.2 (0.0 to 0.3) | 5.5 (3.7 to 7.5) | 5.0 (3.3 to 7.0) | -0.4 (-0.5 to -0.3) |
| Samoa | 57.3 (53.2 to 61.9) | 67.4 (56.3 to 90.4) | 0.8 (0.6 to 1.0) | 4.4 (2.9 to 6.0) | 4.4 (2.8 to 6.6) | 0.4 (0.1 to 0.6) |
| San Marino | 70.3 (63.6 to 82.3) | 65.5 (58.9 to 75.6) | -0.2 (-0.3 to -0.1) | 2.6 (1.6 to 4.1) | 2.4 (1.5 to 3.7) | -0.2 (-0.3 to -0.1) |
| Sao Tome and Principe | 54.0 (49.6 to 59.7) | 54.1 (49.7 to 59.5) | 0.0 (-0.1 to 0.0) | 4.5 (2.9 to 6.2) | 3.7 (2.4 to 5.1) | -0.7 (-0.7 to -0.6) |
| Saudi Arabia | 155.8 (143.2 to 170.3) | 161.1 (147.8 to 177.9) | 0.4 (0.2 to 0.5) | 11.2 (7.6 to 15.3) | 7.2 (4.7 to 10.6) | -1.3 (-1.4 to -1.2) |
| Senegal | 46.6 (43.3 to 50.6) | 44.5 (40.4 to 49.8) | -0.2 (-0.3 to -0.1) | 4.0 (2.7 to 5.6) | 3.4 (2.2 to 4.8) | -0.5 (-0.6 to -0.5) |
| Serbia | 188.3 (174.1 to 208.7) | 173.6 (152.6 to 211.4) | -0.2 (-0.4 to -0.1) | 10.8 (7.3 to 15.0) | 6.6 (4.0 to 10.2) | -1.8 (-2.0 to -1.6) |
| Seychelles | 63.4 (58.5 to 70.0) | 52.6 (48.1 to 57.3) | -0.6 (-0.6 to -0.6) | 4.5 (3.0 to 6.4) | 3.0 (2.0 to 4.3) | -1.4 (-1.4 to -1.3) |
| Sierra Leone | 50.1 (46.8 to 54.0) | 102.0 (63.2 to 179.9) | 1.8 (1.0 to 2.6) | 4.4 (3.0 to 6.1) | 8.0 (4.4 to 15.4) | 1.5 (0.6 to 2.3) |
| Singapore | 68.4 (62.3 to 75.7) | 57.8 (51.9 to 65.4) | -0.6 (-0.7 to -0.5) | 3.4 (2.2 to 4.9) | 2.2 (1.4 to 3.4) | -1.4 (-1.5 to -1.3) |
| Slovakia | 222.8 (204.8 to 245.0) | 178.6 (162.8 to 198.7) | -0.7 (-0.8 to -0.7) | 11.9 (8.0 to 16.8) | 6.6 (4.1 to 10.3) | -2.0 (-2.0 to -1.9) |
| Slovenia | 265.8 (244.9 to 291.9) | 199.0 (181.3 to 221.5) | -0.6 (-0.9 to -0.3) | 11.7 (7.5 to 17.0) | 7.4 (4.6 to 11.2) | -1.1 (-1.3 to -0.9) |
| Solomon Islands | 76.4 (70.8 to 84.2) | 77.2 (69.9 to 87.9) | 0.0 (0.0 to 0.0) | 6.7 (4.5 to 9.2) | 6.1 (4.1 to 8.5) | -0.3 (-0.4 to -0.3) |
| Somalia | 85.7 (60.3 to 202.0) | 123.7 (76.0 to 238.2) | 0.7 (0.6 to 0.8) | 8.2 (4.6 to 20.1) | 11.3 (6.3 to 22.7) | 0.6 (0.5 to 0.7) |
| South Africa | 117.8 (106.8 to 131.8) | 75.6 (68.8 to 85.4) | -1.6 (-1.8 to -1.5) | 8.8 (5.9 to 12.3) | 4.9 (3.4 to 6.9) | -2.1 (-2.4 to -1.9) |
| South Sudan | 82.2 (61.6 to 124.2) | 126.5 (73.9 to 234.9) | 1.6 (1.4 to 1.9) | 7.4 (4.5 to 12.2) | 10.2 (5.4 to 19.8) | 1.3 (1.1 to 1.6) |
| Spain | 76.9 (69.9 to 85.0) | 62.7 (55.4 to 73.6) | -0.7 (-0.8 to -0.6) | 3.0 (1.8 to 4.7) | 2.3 (1.4 to 3.6) | -0.8 (-0.9 to -0.7) |
| Sri Lanka | 115.8 (90.8 to 168.8) | 195.7 (111.9 to 350.7) | 1.6 (1.3 to 1.9) | 8.2 (5.1 to 13.2) | 9.6 (4.7 to 19.6) | 0.3 (-0.1 to 0.6) |
| Sudan | 102.1 (88.0 to 128.9) | 119.4 (86.4 to 187.4) | 0.6 (0.4 to 0.8) | 8.6 (5.8 to 12.2) | 7.8 (4.5 to 13.5) | -0.2 (-0.4 to 0.0) |
| Suriname | 89.2 (73.0 to 138.1) | 82.0 (71.0 to 110.4) | -0.2 (-0.3 to -0.1) | 7.0 (4.4 to 11.5) | 5.5 (3.5 to 8.2) | -0.7 (-0.8 to -0.7) |
| Sweden | 77.7 (70.7 to 88.3) | 59.9 (53.6 to 69.1) | -0.8 (-0.9 to -0.7) | 2.9 (1.7 to 4.4) | 2.2 (1.3 to 3.5) | -0.8 (-0.9 to -0.7) |
| Switzerland | 110.3 (97.8 to 126.0) | 78.3 (68.8 to 92.2) | -1.4 (-1.6 to -1.2) | 4.1 (2.5 to 6.2) | 2.9 (1.7 to 4.5) | -1.4 (-1.6 to -1.2) |
| Syrian Arab Republic | 93.5 (73.8 to 147.0) | 436.7 (173.4 to 1007.1) | 5.1 (3.6 to 6.7) | 6.7 (4.3 to 11.1) | 21.7 (8.2 to 53.6) | 3.8 (2.3 to 5.4) |
| Taiwan (Province of China) | 72.4 (66.3 to 79.8) | 44.1 (40.8 to 48.1) | -2.0 (-2.1 to -1.8) | 3.7 (2.4 to 5.1) | 1.6 (1.0 to 2.5) | -2.9 (-3.1 to -2.8) |
| Tajikistan | 110.2 (102.2 to 120.5) | 134.3 (97.0 to 208.8) | -0.3 (-0.8 to 0.3) | 8.3 (5.6 to 11.3) | 9.2 (5.4 to 15.4) | -0.6 (-1.1 to 0.0) |
| Thailand | 92.0 (85.9 to 98.3) | 71.4 (65.0 to 78.4) | -1.1 (-1.2 to -1.0) | 6.2 (4.1 to 8.7) | 3.3 (2.2 to 5.0) | -2.3 (-2.4 to -2.2) |
| Timor-Leste | 257.3 (116.4 to 533.7) | 284.7 (117.8 to 611.7) | 0.2 (-0.3 to 0.7) | 22.9 (10.0 to 50.5) | 21.0 (8.6 to 49.4) | -0.5 (-0.9 to 0.0) |
| Togo | 53.9 (50.3 to 58.1) | 50.5 (46.9 to 54.7) | -0.2 (-0.3 to -0.2) | 4.6 (3.1 to 6.3) | 3.9 (2.6 to 5.5) | -0.6 (-0.7 to -0.5) |
| Tokelau | 54.2 (50.6 to 58.6) | 47.0 (43.1 to 51.6) | -0.5 (-0.6 to -0.4) | 4.3 (2.9 to 6.0) | 2.9 (1.9 to 4.0) | -1.3 (-1.3 to -1.2) |
| Tonga | 48.1 (45.0 to 51.8) | 39.2 (36.0 to 43.5) | -0.6 (-0.7 to -0.5) | 3.5 (2.3 to 4.9) | 2.5 (1.7 to 3.4) | -1.0 (-1.1 to -0.9) |
| Trinidad and Tobago | 72.9 (68.1 to 78.0) | 82.0 (73.4 to 93.5) | 0.5 (0.4 to 0.7) | 5.2 (3.5 to 7.2) | 4.8 (3.2 to 6.9) | -0.3 (-0.4 to -0.2) |
| Tunisia | 104.6 (96.5 to 115.4) | 83.8 (77.3 to 92.3) | -0.7 (-0.8 to -0.7) | 6.8 (4.6 to 9.5) | 3.8 (2.5 to 5.5) | -1.9 (-1.9 to -1.9) |
| Turkey | 78.6 (73.8 to 85.8) | 69.7 (62.5 to 81.6) | -0.4 (-0.5 to -0.4) | 5.8 (3.9 to 8.0) | 3.1 (2.0 to 4.6) | -2.2 (-2.2 to -2.1) |
| Turkmenistan | 109.5 (101.8 to 119.9) | 94.7 (86.7 to 104.4) | -0.5 (-0.6 to -0.5) | 8.0 (5.5 to 11.3) | 5.8 (3.8 to 8.3) | -1.1 (-1.2 to -1.1) |
| Tuvalu | 64.5 (60.3 to 69.7) | 56.9 (52.5 to 61.8) | -0.9 (-1.2 to -0.7) | 5.6 (3.8 to 7.8) | 4.0 (2.7 to 5.7) | -1.5 (-1.7 to -1.3) |
| Uganda | 148.9 (77.3 to 307.9) | 107.0 (62.1 to 201.7) | -1.1 (-1.2 to -1.0) | 13.0 (6.4 to 27.3) | 8.2 (4.4 to 16.1) | -1.6 (-1.7 to -1.4) |
| Ukraine | 197.8 (180.6 to 216.5) | 171.0 (157.2 to 189.0) | -0.9 (-1.0 to -0.7) | 10.6 (7.2 to 14.7) | 8.1 (5.3 to 11.6) | -1.4 (-1.6 to -1.2) |
| United Arab Emirates | 111.3 (103.1 to 121.5) | 99.0 (91.1 to 109.4) | -0.4 (-0.4 to -0.4) | 7.8 (5.3 to 10.8) | 5.0 (3.3 to 7.1) | -1.4 (-1.5 to -1.3) |
| United Kingdom | 66.9 (61.2 to 75.3) | 54.9 (49.4 to 63.0) | -0.6 (-0.8 to -0.5) | 2.7 (1.7 to 4.1) | 2.0 (1.2 to 3.2) | -0.9 (-1.0 to -0.8) |
| United Republic of Tanzania | 52.7 (49.4 to 56.6) | 46.6 (43.4 to 50.0) | -0.4 (-0.5 to -0.4) | 4.5 (3.1 to 6.4) | 3.6 (2.4 to 5.0) | -0.7 (-0.8 to -0.7) |
| United States of America | 73.5 (68.4 to 80.9) | 53.8 (49.8 to 57.7) | -1.2 (-1.4 to -1.0) | 2.8 (1.8 to 4.2) | 2.0 (1.2 to 2.9) | -1.3 (-1.6 to -1.1) |
| United States Virgin Islands | 87.2 (81.9 to 93.2) | 74.2 (69.3 to 80.0) | -0.8 (-1.0 to -0.6) | 5.6 (3.8 to 7.7) | 3.9 (2.6 to 5.4) | -1.5 (-1.6 to -1.3) |
| Uruguay | 96.9 (87.8 to 106.0) | 83.0 (76.3 to 91.5) | -0.6 (-0.7 to -0.6) | 5.8 (3.8 to 8.1) | 3.8 (2.4 to 5.5) | -1.5 (-1.5 to -1.4) |
| Uzbekistan | 108.3 (100.8 to 117.9) | 91.1 (84.3 to 99.7) | -0.6 (-0.7 to -0.5) | 7.6 (5.1 to 10.6) | 5.6 (3.8 to 7.7) | -1.1 (-1.3 to -1.0) |
| Vanuatu | 58.3 (53.0 to 66.5) | 54.9 (49.1 to 64.1) | -0.2 (-0.3 to -0.1) | 4.9 (3.2 to 6.9) | 4.3 (2.8 to 6.1) | -0.4 (-0.5 to -0.3) |
| Venezuela (Bolivarian Republic of) | 131.0 (121.3 to 144.3) | 133.2 (122.3 to 147.2) | 0.1 (0.0 to 0.2) | 9.5 (6.4 to 13.0) | 7.6 (5.0 to 10.8) | -0.7 (-0.8 to -0.5) |
| Viet Nam | 67.1 (62.8 to 71.3) | 63.8 (59.1 to 68.7) | 0.0 (-0.1 to 0.1) | 5.0 (3.3 to 7.0) | 3.4 (2.2 to 4.9) | -1.1 (-1.2 to -1.1) |
| Yemen | 113.2 (98.3 to 143.2) | 164.4 (107.1 to 280.8) | 0.7 (0.3 to 1.1) | 9.5 (6.2 to 13.9) | 11.7 (6.5 to 21.2) | 0.1 (-0.4 to 0.5) |
| Zambia | 52.6 (49.2 to 56.0) | 48.6 (45.2 to 52.3) | -0.3 (-0.4 to -0.2) | 4.7 (3.2 to 6.6) | 3.7 (2.5 to 5.3) | -0.9 (-1.0 to -0.7) |
| Zimbabwe | 56.1 (52.7 to 60.0) | 53.2 (49.8 to 56.7) | -0.2 (-0.3 to -0.1) | 4.4 (2.9 to 6.2) | 4.3 (2.9 to 6.0) | 0.0 (-0.1 to 0.2) |

**Table S18. ASR of prevalence and YLDs in 1990 and 2021, and their EAPC from 1990 to 2021 for AMP-UL2, by country.**

| **Countries** | **Prevalence** | | | **YLDs** | | |
| --- | --- | --- | --- | --- | --- | --- |
|  | **Age-standardized prevalence rate, 1990** | **Age-standardized prevalence rate, 2021** | **EAPC of prevalence rate No. (95% CI)** | **Age-standardized YLDs rate, 1990** | **Age-standardized YLDs rate, 2021** | **EAPC of YLDs rate No. (95% CI)** |
| Afghanistan | 741.6 (171.2 to 2383.5) | 656.1 (215.9 to 1802.3) | -0.6 (-0.6 to -0.5) | 241.4 (53.7 to 816.4) | 173.6 (51.7 to 450.3) | -1.3 (-1.4 to -1.2) |
| Albania | 310.6 (279.4 to 349.3) | 250.0 (225.2 to 283.3) | -1.1 (-1.3 to -0.9) | 63.2 (44.6 to 83.8) | 25.0 (17.2 to 34.1) | -3.6 (-3.9 to -3.4) |
| Algeria | 112.3 (104.4 to 121.9) | 89.7 (79.2 to 106.7) | -0.7 (-0.8 to -0.6) | 26.3 (17.9 to 34.3) | 12.7 (8.5 to 17.1) | -2.3 (-2.5 to -2.2) |
| American Samoa | 68.3 (63.7 to 74.3) | 70.9 (60.3 to 87.5) | 0.4 (0.2 to 0.6) | 15.6 (11.0 to 20.7) | 14.1 (9.4 to 19.3) | 0.1 (-0.2 to 0.3) |
| Andorra | 79.9 (71.2 to 92.2) | 75.9 (67.4 to 86.8) | -0.2 (-0.3 to -0.1) | 6.6 (4.1 to 9.5) | 6.2 (3.9 to 8.9) | -0.2 (-0.3 to -0.1) |
| Angola | 161.5 (92.0 to 307.9) | 141.9 (76.3 to 279.7) | -0.6 (-0.9 to -0.3) | 54.5 (27.7 to 103.4) | 37.4 (18.8 to 73.5) | -1.4 (-1.8 to -1.0) |
| Antigua and Barbuda | 117.6 (105.7 to 130.6) | 127.5 (114.4 to 141.8) | 0.2 (0.0 to 0.4) | 23.5 (16.1 to 31.4) | 18.7 (13.0 to 25.2) | -0.8 (-1.0 to -0.6) |
| Argentina | 68.7 (63.7 to 75.0) | 63.6 (58.3 to 70.6) | -0.2 (-0.4 to -0.1) | 14.1 (9.7 to 18.7) | 9.1 (6.3 to 12.2) | -1.4 (-1.5 to -1.2) |
| Armenia | 262.3 (198.0 to 406.8) | 182.9 (136.6 to 259.6) | -1.7 (-1.9 to -1.5) | 50.6 (31.1 to 78.1) | 23.7 (14.6 to 35.9) | -3.2 (-3.4 to -2.9) |
| Australia | 100.5 (89.6 to 117.4) | 86.2 (75.9 to 101.8) | -0.5 (-0.6 to -0.4) | 8.3 (5.4 to 11.8) | 7.1 (4.6 to 10.4) | -0.5 (-0.6 to -0.4) |
| Austria | 89.6 (79.3 to 102.3) | 69.5 (61.4 to 80.6) | -0.8 (-0.9 to -0.7) | 8.2 (5.4 to 11.5) | 5.7 (3.7 to 8.2) | -0.9 (-1.0 to -0.8) |
| Azerbaijan | 135.0 (124.6 to 146.6) | 124.1 (107.9 to 153.9) | -0.7 (-0.9 to -0.6) | 31.5 (21.9 to 41.7) | 21.2 (14.2 to 29.6) | -1.9 (-2.1 to -1.7) |
| Bahamas | 83.7 (78.6 to 90.8) | 95.1 (86.2 to 109.2) | 0.4 (0.3 to 0.4) | 18.4 (12.6 to 24.3) | 16.4 (11.2 to 22.1) | -0.4 (-0.5 to -0.4) |
| Bahrain | 81.4 (74.5 to 90.2) | 75.2 (68.5 to 83.0) | -0.8 (-1.1 to -0.5) | 16.9 (12.0 to 22.7) | 7.6 (5.2 to 10.6) | -3.2 (-3.5 to -2.9) |
| Bangladesh | 51.9 (47.3 to 58.1) | 55.5 (46.2 to 73.9) | -0.4 (-0.6 to -0.2) | 15.9 (11.0 to 22.2) | 11.3 (7.5 to 16.2) | -1.8 (-2.0 to -1.6) |
| Barbados | 78.1 (73.1 to 84.8) | 78.7 (72.3 to 86.1) | -0.1 (-0.3 to 0.1) | 15.4 (10.6 to 20.4) | 11.2 (7.8 to 14.8) | -1.1 (-1.2 to -1.0) |
| Belarus | 201.0 (185.0 to 221.4) | 172.8 (157.5 to 190.6) | -0.6 (-0.9 to -0.4) | 32.2 (22.6 to 42.7) | 15.1 (10.0 to 21.0) | -2.8 (-3.3 to -2.3) |
| Belgium | 83.8 (74.6 to 96.4) | 72.8 (64.3 to 83.3) | -0.2 (-0.7 to 0.2) | 7.4 (4.8 to 10.4) | 6.0 (3.9 to 8.5) | -0.3 (-0.8 to 0.1) |
| Belize | 101.3 (93.5 to 110.0) | 121.9 (111.6 to 134.2) | 0.4 (0.1 to 0.6) | 23.9 (16.3 to 31.7) | 23.0 (15.9 to 30.1) | -0.4 (-0.7 to -0.2) |
| Benin | 59.3 (55.5 to 63.7) | 55.3 (51.8 to 59.5) | -0.3 (-0.3 to -0.2) | 18.2 (12.3 to 24.4) | 14.5 (10.2 to 19.0) | -0.8 (-0.8 to -0.7) |
| Bermuda | 81.8 (75.7 to 89.9) | 79.3 (73.0 to 88.0) | -0.2 (-0.3 to -0.1) | 13.2 (9.0 to 18.0) | 6.6 (4.2 to 9.2) | -2.4 (-2.6 to -2.2) |
| Bhutan | 63.4 (58.9 to 68.6) | 68.1 (61.4 to 76.1) | 0.2 (0.1 to 0.3) | 19.4 (13.4 to 26.2) | 14.3 (9.6 to 19.4) | -1.1 (-1.2 to -0.9) |
| Bolivia (Plurinational State of) | 98.4 (92.0 to 105.9) | 82.0 (75.7 to 90.0) | -0.7 (-0.7 to -0.6) | 30.5 (21.4 to 40.1) | 18.3 (12.7 to 24.2) | -1.7 (-1.8 to -1.7) |
| Bosnia and Herzegovina | 299.2 (265.8 to 336.9) | 287.7 (218.1 to 437.6) | -0.7 (-1.0 to -0.4) | 54.3 (37.3 to 72.2) | 28.9 (17.9 to 45.2) | -3.0 (-3.4 to -2.6) |
| Botswana | 61.0 (57.0 to 65.3) | 70.2 (65.2 to 75.5) | 0.4 (0.1 to 0.6) | 17.3 (11.8 to 22.5) | 16.4 (11.7 to 21.4) | -0.3 (-0.7 to 0.0) |
| Brazil | 135.1 (124.6 to 150.3) | 110.9 (100.2 to 126.6) | -0.9 (-1.0 to -0.8) | 32.3 (22.5 to 42.3) | 18.6 (12.9 to 24.6) | -2.0 (-2.1 to -1.9) |
| Brunei Darussalam | 71.4 (65.3 to 78.9) | 54.7 (49.0 to 61.0) | -0.9 (-1.0 to -0.9) | 15.1 (10.3 to 19.9) | 8.2 (5.7 to 11.2) | -2.0 (-2.1 to -1.9) |
| Bulgaria | 272.0 (249.7 to 302.2) | 230.2 (208.1 to 258.0) | -0.5 (-0.6 to -0.4) | 45.3 (32.1 to 59.9) | 27.6 (18.9 to 37.4) | -1.6 (-1.8 to -1.5) |
| Burkina Faso | 58.7 (54.9 to 63.1) | 60.0 (54.9 to 65.8) | 0.0 (-0.1 to 0.1) | 17.8 (12.2 to 23.0) | 16.6 (11.4 to 21.8) | -0.3 (-0.4 to -0.2) |
| Burundi | 56.4 (53.2 to 60.6) | 352.9 (140.9 to 798.2) | 6.1 (4.4 to 7.8) | 18.7 (12.7 to 25.2) | 103.6 (39.1 to 230.4) | 5.6 (3.9 to 7.4) |
| Cabo Verde | 69.7 (64.9 to 74.6) | 63.4 (58.7 to 68.9) | -0.2 (-0.3 to -0.2) | 17.3 (12.1 to 22.9) | 11.5 (8.0 to 15.6) | -1.5 (-1.6 to -1.4) |
| Cambodia | 248.9 (93.5 to 675.4) | 166.2 (78.1 to 394.6) | -1.3 (-1.4 to -1.2) | 79.6 (27.6 to 224.0) | 38.2 (16.0 to 96.1) | -2.5 (-2.6 to -2.3) |
| Cameroon | 54.2 (50.2 to 59.7) | 56.9 (50.8 to 66.9) | 0.0 (0.0 to 0.1) | 16.0 (11.2 to 21.5) | 14.5 (10.0 to 19.7) | -0.4 (-0.5 to -0.4) |
| Canada | 52.5 (48.5 to 57.8) | 41.9 (38.0 to 46.0) | -0.7 (-0.7 to -0.7) | 4.3 (2.8 to 6.1) | 3.4 (2.2 to 4.8) | -0.7 (-0.8 to -0.7) |
| Central African Republic | 62.5 (58.8 to 66.5) | 100.6 (72.8 to 157.0) | 1.7 (1.4 to 1.9) | 21.3 (14.9 to 27.7) | 33.3 (20.1 to 53.4) | 1.6 (1.3 to 1.8) |
| Chad | 102.9 (69.1 to 175.1) | 103.0 (71.9 to 168.2) | 0.0 (-0.1 to 0.1) | 33.1 (19.7 to 58.6) | 30.1 (18.1 to 49.5) | -0.3 (-0.5 to -0.2) |
| Chile | 67.8 (62.3 to 74.9) | 71.5 (64.2 to 81.3) | 0.3 (0.1 to 0.4) | 13.1 (9.1 to 17.4) | 6.5 (4.3 to 9.2) | -2.3 (-2.4 to -2.2) |
| China | 77.6 (72.7 to 84.0) | 67.2 (62.0 to 74.0) | -0.8 (-1.0 to -0.6) | 18.3 (12.8 to 24.0) | 6.4 (4.4 to 8.8) | -4.1 (-4.5 to -3.8) |
| Colombia | 192.1 (173.6 to 217.9) | 134.6 (116.2 to 165.2) | -1.2 (-1.2 to -1.1) | 45.0 (31.2 to 60.9) | 18.2 (12.3 to 25.0) | -2.9 (-3.0 to -2.8) |
| Comoros | 61.9 (58.1 to 66.0) | 59.3 (53.7 to 67.9) | -0.3 (-0.4 to -0.2) | 19.1 (13.3 to 25.2) | 15.5 (10.7 to 20.9) | -0.9 (-1.0 to -0.8) |
| Congo | 58.4 (54.9 to 62.4) | 104.2 (62.7 to 197.0) | 2.0 (1.1 to 3.0) | 18.4 (12.8 to 24.6) | 26.3 (13.9 to 50.3) | 1.3 (0.2 to 2.3) |
| Cook Islands | 61.3 (56.1 to 68.6) | 60.3 (51.9 to 74.5) | 0.0 (-0.2 to 0.2) | 12.3 (8.4 to 16.6) | 7.3 (4.9 to 10.2) | -1.7 (-1.8 to -1.5) |
| Costa Rica | 108.6 (99.1 to 120.6) | 99.8 (91.5 to 112.7) | -0.3 (-0.4 to -0.2) | 19.1 (13.4 to 25.8) | 11.7 (8.2 to 15.8) | -1.7 (-1.7 to -1.6) |
| Coted'Ivoire | 62.3 (58.3 to 66.5) | 60.9 (55.9 to 67.4) | -0.1 (-0.2 to -0.1) | 18.3 (12.7 to 24.2) | 15.2 (10.5 to 19.8) | -0.7 (-0.7 to -0.6) |
| Croatia | 247.2 (225.6 to 273.9) | 206.4 (180.6 to 244.0) | -0.8 (-0.9 to -0.7) | 29.9 (21.1 to 40.1) | 17.0 (10.9 to 24.3) | -2.1 (-2.3 to -2.0) |
| Cuba | 100.7 (93.6 to 110.3) | 100.0 (92.7 to 111.1) | 0.0 (-0.1 to 0.2) | 17.2 (12.0 to 23.1) | 11.1 (7.6 to 14.9) | -1.3 (-1.4 to -1.2) |
| Cyprus | 74.3 (66.7 to 86.1) | 66.2 (58.2 to 77.0) | -0.4 (-0.6 to -0.2) | 9.4 (6.3 to 12.7) | 5.5 (3.5 to 7.8) | -1.7 (-1.8 to -1.5) |
| Czechia | 296.0 (269.6 to 328.5) | 215.5 (196.4 to 237.8) | -0.7 (-0.9 to -0.4) | 41.4 (29.1 to 55.7) | 17.7 (11.6 to 25.2) | -2.0 (-2.3 to -1.6) |
| Democratic People's Republic of Korea | 56.8 (52.8 to 61.7) | 47.5 (44.1 to 51.0) | -0.5 (-0.6 to -0.5) | 13.4 (9.5 to 17.6) | 8.4 (5.8 to 11.2) | -1.5 (-1.6 to -1.5) |
| Democratic Republic of the Congo | 57.6 (53.9 to 61.8) | 76.7 (57.0 to 119.6) | 1.0 (0.6 to 1.3) | 18.0 (12.5 to 23.5) | 21.0 (13.1 to 33.7) | 0.5 (0.1 to 1.0) |
| Denmark | 68.1 (60.8 to 81.0) | 58.0 (50.9 to 68.8) | -0.5 (-0.7 to -0.4) | 6.8 (4.6 to 9.6) | 4.8 (3.0 to 7.0) | -0.9 (-1.0 to -0.8) |
| Djibouti | 62.7 (58.6 to 67.5) | 67.0 (58.6 to 82.2) | -0.2 (-0.4 to 0.1) | 19.0 (13.3 to 25.5) | 17.1 (11.5 to 24.1) | -0.7 (-1.0 to -0.5) |
| Dominica | 76.2 (70.9 to 83.3) | 88.9 (79.7 to 103.9) | 0.5 (0.4 to 0.6) | 18.0 (12.4 to 24.3) | 18.0 (12.3 to 24.6) | 0.0 (0.0 to 0.1) |
| Dominican Republic | 81.7 (76.5 to 88.6) | 95.6 (88.4 to 104.1) | 0.6 (0.4 to 0.8) | 21.4 (14.8 to 28.2) | 19.1 (13.4 to 25.3) | -0.2 (-0.3 to 0.0) |
| Ecuador | 107.9 (98.0 to 120.1) | 97.1 (88.5 to 107.3) | -0.3 (-0.5 to -0.2) | 28.3 (19.4 to 37.8) | 16.6 (11.6 to 22.0) | -1.7 (-1.8 to -1.5) |
| Egypt | 90.3 (84.1 to 98.9) | 74.5 (67.3 to 89.3) | -0.6 (-0.6 to -0.5) | 23.8 (16.0 to 31.6) | 12.9 (8.7 to 17.3) | -1.8 (-1.9 to -1.7) |
| El Salvador | 404.7 (212.5 to 816.5) | 267.8 (163.1 to 489.0) | -1.4 (-1.5 to -1.3) | 106.5 (51.8 to 214.9) | 42.3 (23.7 to 77.6) | -3.0 (-3.1 to -3.0) |
| Equatorial Guinea | 61.4 (57.6 to 65.6) | 44.5 (41.5 to 48.3) | -1.1 (-1.2 to -1.0) | 20.4 (14.2 to 27.1) | 9.5 (6.5 to 12.6) | -2.7 (-2.8 to -2.6) |
| Eritrea | 588.9 (210.4 to 1417.6) | 414.0 (155.0 to 985.4) | -1.4 (-1.5 to -1.2) | 198.2 (68.8 to 466.4) | 117.3 (42.7 to 274.9) | -1.9 (-2.1 to -1.7) |
| Estonia | 248.5 (229.2 to 271.6) | 152.3 (138.7 to 169.3) | -2.0 (-2.1 to -1.8) | 38.0 (26.7 to 50.5) | 12.5 (8.2 to 17.6) | -4.4 (-4.7 to -4.1) |
| Eswatini | 79.6 (74.1 to 86.3) | 73.9 (68.4 to 80.1) | -0.2 (-0.3 to -0.1) | 22.5 (15.6 to 30.1) | 18.5 (12.8 to 24.5) | -0.6 (-0.8 to -0.4) |
| Ethiopia | 104.7 (87.8 to 141.3) | 91.2 (63.2 to 159.8) | -0.7 (-0.9 to -0.5) | 36.1 (23.9 to 51.6) | 23.3 (14.0 to 43.1) | -1.7 (-2.0 to -1.5) |
| Fiji | 52.9 (49.2 to 57.6) | 48.4 (43.8 to 54.8) | -0.4 (-0.4 to -0.3) | 13.6 (9.3 to 17.7) | 11.2 (7.7 to 14.9) | -0.7 (-0.8 to -0.6) |
| Finland | 100.3 (88.2 to 115.0) | 84.7 (73.7 to 102.7) | -0.6 (-1.1 to -0.1) | 10.4 (7.1 to 14.4) | 7.0 (4.5 to 10.0) | -1.0 (-1.4 to -0.6) |
| France | 84.9 (76.1 to 97.2) | 70.1 (62.3 to 80.8) | -0.6 (-0.7 to -0.5) | 8.5 (5.8 to 11.8) | 5.8 (3.7 to 8.3) | -0.9 (-1.0 to -0.7) |
| Gabon | 60.4 (56.6 to 65.0) | 50.6 (47.2 to 54.9) | -0.6 (-0.6 to -0.5) | 17.7 (12.2 to 23.2) | 11.4 (8.0 to 15.5) | -1.4 (-1.5 to -1.3) |
| Gambia | 63.9 (53.5 to 84.9) | 53.5 (46.7 to 65.2) | -0.6 (-0.7 to -0.5) | 18.6 (12.2 to 26.1) | 13.4 (9.2 to 18.2) | -1.1 (-1.3 to -1.0) |
| Georgia | 166.6 (153.1 to 183.3) | 181.5 (163.4 to 205.4) | 0.4 (0.3 to 0.5) | 30.1 (21.3 to 40.6) | 27.9 (19.4 to 37.1) | 0.0 (-0.1 to 0.2) |
| Germany | 70.7 (63.8 to 83.5) | 61.1 (54.2 to 72.1) | -0.5 (-0.6 to -0.5) | 7.3 (4.9 to 10.1) | 5.0 (3.1 to 7.2) | -0.8 (-1.0 to -0.7) |
| Ghana | 49.8 (46.2 to 54.0) | 47.0 (43.7 to 51.1) | -0.2 (-0.3 to -0.1) | 14.4 (10.0 to 19.1) | 11.4 (7.9 to 15.3) | -0.7 (-0.8 to -0.6) |
| Greece | 79.8 (71.2 to 92.6) | 59.9 (52.6 to 70.0) | -1.0 (-1.1 to -0.8) | 6.6 (4.3 to 9.4) | 5.0 (3.1 to 7.1) | -0.9 (-1.1 to -0.8) |
| Greenland | 82.2 (76.1 to 89.6) | 60.8 (54.9 to 67.9) | -1.2 (-1.3 to -1.1) | 16.4 (11.4 to 21.7) | 8.0 (5.5 to 10.9) | -2.6 (-2.7 to -2.5) |
| Grenada | 140.0 (105.8 to 216.0) | 125.3 (103.8 to 167.7) | -0.4 (-0.5 to -0.2) | 34.3 (21.7 to 55.6) | 22.7 (15.1 to 33.0) | -1.2 (-1.3 to -1.1) |
| Guam | 62.2 (57.5 to 68.6) | 55.5 (50.8 to 61.7) | -0.3 (-0.4 to -0.3) | 11.0 (7.5 to 14.4) | 8.5 (6.0 to 11.6) | -0.7 (-0.9 to -0.6) |
| Guatemala | 258.0 (172.2 to 449.4) | 208.9 (159.9 to 317.9) | -0.7 (-0.8 to -0.7) | 77.4 (45.4 to 136.0) | 43.7 (27.9 to 71.4) | -1.9 (-1.9 to -1.8) |
| Guinea | 60.7 (56.8 to 65.0) | 63.8 (58.7 to 70.2) | 0.3 (0.2 to 0.3) | 19.3 (13.2 to 25.5) | 17.9 (12.1 to 24.1) | -0.1 (-0.2 to 0.0) |
| Guinea-Bissau | 71.3 (67.2 to 75.9) | 68.1 (60.8 to 80.7) | -0.1 (-0.3 to 0.1) | 23.9 (16.3 to 31.9) | 20.0 (13.9 to 26.8) | -0.6 (-0.8 to -0.3) |
| Guyana | 95.3 (88.6 to 102.2) | 108.7 (100.8 to 117.3) | 0.3 (0.2 to 0.5) | 27.2 (18.7 to 36.0) | 25.5 (17.2 to 33.5) | -0.2 (-0.3 to 0.0) |
| Haiti | 116.8 (108.3 to 126.4) | 228.7 (141.5 to 424.3) | 3.3 (2.5 to 4.1) | 40.0 (27.0 to 52.7) | 67.6 (34.7 to 128.3) | 2.8 (2.0 to 3.6) |
| Honduras | 136.2 (125.5 to 148.3) | 130.1 (116.4 to 151.0) | -0.1 (-0.3 to 0.0) | 39.1 (27.2 to 52.7) | 29.9 (20.7 to 39.5) | -0.9 (-1.0 to -0.7) |
| Hungary | 249.3 (228.7 to 276.4) | 190.2 (173.2 to 213.1) | -0.9 (-1.0 to -0.8) | 39.6 (27.1 to 52.1) | 15.7 (10.3 to 22.0) | -3.0 (-3.1 to -2.9) |
| Iceland | 76.6 (68.6 to 87.2) | 65.0 (57.2 to 75.3) | -0.5 (-0.6 to -0.4) | 6.3 (4.1 to 8.9) | 5.4 (3.5 to 7.7) | -0.5 (-0.6 to -0.4) |
| India | 86.3 (79.9 to 94.7) | 70.1 (63.9 to 77.8) | -0.8 (-0.9 to -0.7) | 25.1 (17.4 to 32.8) | 14.8 (10.4 to 19.6) | -1.9 (-2.1 to -1.8) |
| Indonesia | 78.6 (69.4 to 100.5) | 60.3 (52.1 to 76.0) | -0.8 (-0.9 to -0.7) | 22.2 (14.8 to 31.6) | 13.3 (9.1 to 18.7) | -1.6 (-1.7 to -1.4) |
| Iran (Islamic Republic of) | 182.1 (143.7 to 254.7) | 124.7 (102.3 to 163.9) | -1.3 (-1.4 to -1.2) | 39.4 (25.4 to 58.2) | 14.3 (9.5 to 20.5) | -3.3 (-3.4 to -3.1) |
| Iraq | 467.7 (257.2 to 866.3) | 439.3 (225.9 to 860.9) | 0.0 (-0.1 to 0.1) | 109.6 (56.2 to 208.9) | 63.4 (31.3 to 125.1) | -1.7 (-1.8 to -1.6) |
| Ireland | 69.0 (61.9 to 79.8) | 63.2 (55.1 to 76.6) | -0.3 (-0.5 to -0.1) | 6.7 (4.5 to 9.4) | 5.2 (3.4 to 7.6) | -0.5 (-0.7 to -0.4) |
| Israel | 64.6 (58.0 to 75.9) | 69.7 (56.5 to 93.5) | 0.4 (0.2 to 0.6) | 7.6 (5.3 to 10.6) | 5.8 (3.6 to 8.8) | -0.5 (-0.7 to -0.3) |
| Italy | 87.3 (78.7 to 98.4) | 68.5 (59.7 to 80.5) | -0.9 (-1.0 to -0.8) | 7.7 (5.1 to 10.7) | 5.7 (3.7 to 8.1) | -1.0 (-1.1 to -0.9) |
| Jamaica | 108.4 (99.9 to 118.3) | 100.8 (92.1 to 111.2) | -0.6 (-0.8 to -0.5) | 22.0 (15.2 to 29.1) | 16.5 (11.3 to 22.0) | -1.3 (-1.5 to -1.2) |
| Japan | 61.5 (56.7 to 68.4) | 45.6 (41.5 to 51.4) | -1.0 (-1.2 to -0.9) | 5.2 (3.5 to 7.3) | 3.8 (2.5 to 5.4) | -1.0 (-1.2 to -0.9) |
| Jordan | 82.8 (77.3 to 89.8) | 65.5 (60.2 to 72.0) | -0.8 (-0.8 to -0.7) | 17.5 (12.2 to 23.1) | 7.7 (5.1 to 10.5) | -3.0 (-3.2 to -2.8) |
| Kazakhstan | 187.3 (172.9 to 204.4) | 148.1 (136.6 to 162.5) | -0.9 (-1.0 to -0.7) | 39.4 (28.0 to 51.5) | 21.1 (15.0 to 28.5) | -2.4 (-2.8 to -2.0) |
| Kenya | 61.4 (57.8 to 65.2) | 58.8 (54.1 to 64.1) | -0.3 (-0.4 to -0.1) | 16.7 (11.8 to 21.5) | 14.3 (10.1 to 18.5) | -0.4 (-0.7 to -0.2) |
| Kiribati | 59.5 (54.3 to 65.7) | 45.9 (41.7 to 51.8) | -0.7 (-0.8 to -0.6) | 19.4 (13.1 to 25.8) | 13.5 (9.0 to 18.1) | -1.0 (-1.1 to -0.9) |
| Kuwait | 100.5 (90.8 to 112.7) | 99.6 (81.7 to 133.4) | -0.7 (-0.9 to -0.4) | 13.9 (9.6 to 18.2) | 8.1 (5.2 to 12.3) | -2.9 (-3.2 to -2.5) |
| Kyrgyzstan | 150.3 (139.7 to 164.5) | 104.4 (96.0 to 114.7) | -1.3 (-1.3 to -1.2) | 34.2 (24.2 to 45.5) | 17.5 (12.1 to 23.3) | -2.3 (-2.4 to -2.2) |
| Lao People's Democratic Republic | 83.8 (77.9 to 91.3) | 59.4 (54.3 to 64.9) | -1.3 (-1.3 to -1.2) | 28.1 (19.4 to 37.0) | 15.2 (10.7 to 20.5) | -2.2 (-2.3 to -2.1) |
| Latvia | 262.0 (241.3 to 283.9) | 161.7 (148.0 to 179.3) | -2.0 (-2.1 to -1.8) | 41.0 (28.6 to 53.5) | 15.9 (10.8 to 22.0) | -3.4 (-3.6 to -3.1) |
| Lebanon | 423.2 (177.3 to 1083.0) | 235.0 (102.3 to 579.9) | -1.9 (-2.0 to -1.9) | 85.2 (31.4 to 240.9) | 21.8 (8.4 to 57.0) | -4.5 (-4.6 to -4.4) |
| Lesotho | 65.2 (61.2 to 69.5) | 88.0 (81.1 to 97.1) | 1.1 (0.8 to 1.3) | 19.3 (13.1 to 25.8) | 24.6 (17.1 to 32.7) | 0.9 (0.7 to 1.2) |
| Liberia | 69.8 (59.9 to 88.0) | 144.2 (75.4 to 286.2) | 1.5 (0.8 to 2.2) | 21.7 (14.6 to 29.9) | 35.6 (17.3 to 70.4) | 0.6 (-0.1 to 1.4) |
| Libya | 111.4 (98.4 to 135.1) | 175.2 (130.2 to 271.0) | 1.9 (1.5 to 2.2) | 23.5 (16.2 to 31.8) | 25.0 (14.9 to 39.5) | 0.5 (0.1 to 0.9) |
| Lithuania | 231.3 (212.6 to 254.7) | 160.5 (146.5 to 175.8) | -1.4 (-1.6 to -1.2) | 34.4 (24.0 to 45.8) | 16.5 (11.2 to 22.7) | -2.4 (-2.6 to -2.2) |
| Luxembourg | 88.0 (78.5 to 100.1) | 69.5 (61.5 to 79.4) | -0.8 (-0.9 to -0.8) | 9.2 (6.2 to 12.5) | 5.7 (3.6 to 8.0) | -1.2 (-1.3 to -1.0) |
| Madagascar | 56.6 (53.1 to 61.0) | 46.0 (42.9 to 49.7) | -0.7 (-0.7 to -0.6) | 17.7 (12.1 to 23.3) | 12.7 (8.6 to 17.1) | -1.0 (-1.1 to -1.0) |
| Malawi | 51.4 (47.9 to 55.6) | 44.1 (41.1 to 47.6) | -0.6 (-0.7 to -0.5) | 16.2 (11.1 to 21.6) | 11.9 (8.4 to 15.6) | -1.1 (-1.2 to -1.0) |
| Malaysia | 60.2 (56.4 to 64.6) | 53.2 (49.4 to 57.2) | -0.5 (-0.5 to -0.4) | 14.3 (9.6 to 19.1) | 8.3 (5.9 to 10.9) | -1.9 (-1.9 to -1.8) |
| Maldives | 73.5 (67.3 to 80.4) | 62.3 (56.1 to 70.4) | -0.4 (-0.5 to -0.2) | 19.7 (13.6 to 26.3) | 8.4 (5.7 to 11.4) | -2.7 (-2.9 to -2.6) |
| Mali | 67.1 (62.6 to 72.1) | 98.5 (73.0 to 150.6) | 1.4 (1.0 to 1.8) | 21.0 (14.3 to 28.0) | 26.6 (16.9 to 42.2) | 0.9 (0.6 to 1.3) |
| Malta | 71.9 (63.7 to 83.2) | 68.2 (59.6 to 80.2) | 0.0 (-0.3 to 0.2) | 7.6 (5.1 to 10.5) | 5.6 (3.7 to 8.1) | -0.5 (-0.7 to -0.2) |
| Marshall Islands | 69.9 (64.1 to 75.7) | 60.6 (55.6 to 66.6) | -0.5 (-0.5 to -0.4) | 20.5 (14.5 to 27.5) | 15.5 (10.8 to 20.5) | -0.9 (-1.0 to -0.8) |
| Mauritania | 60.5 (56.8 to 64.2) | 50.4 (46.8 to 54.7) | -0.6 (-0.7 to -0.6) | 18.4 (12.5 to 23.8) | 11.1 (7.6 to 15.0) | -1.6 (-1.7 to -1.5) |
| Mauritius | 56.8 (53.0 to 61.6) | 49.3 (45.1 to 53.6) | -0.4 (-0.4 to -0.3) | 11.8 (8.3 to 15.5) | 7.6 (5.3 to 10.3) | -1.2 (-1.3 to -1.1) |
| Mexico | 175.6 (161.3 to 194.3) | 113.2 (105.0 to 123.8) | -0.1 (-0.6 to 0.4) | 42.6 (29.0 to 55.6) | 19.0 (13.5 to 25.3) | -1.2 (-1.7 to -0.7) |
| Micronesia (Federated States of) | 71.0 (65.6 to 76.9) | 68.7 (61.7 to 77.1) | 0.0 (-0.1 to 0.1) | 21.1 (14.5 to 28.4) | 16.7 (11.5 to 22.6) | -0.7 (-0.8 to -0.6) |
| Monaco | 60.8 (54.2 to 70.9) | 59.6 (51.8 to 72.1) | 0.0 (-0.1 to 0.1) | 5.0 (3.2 to 7.3) | 4.9 (3.2 to 7.1) | 0.0 (-0.1 to 0.1) |
| Mongolia | 164.4 (151.8 to 179.3) | 159.5 (145.9 to 178.7) | 0.0 (-0.1 to 0.0) | 47.2 (32.4 to 62.1) | 31.4 (21.8 to 41.2) | -1.4 (-1.5 to -1.3) |
| Montenegro | 231.8 (211.7 to 258.5) | 198.1 (179.1 to 222.3) | -0.5 (-0.6 to -0.4) | 26.0 (18.2 to 35.5) | 16.3 (10.6 to 22.9) | -2.0 (-2.3 to -1.8) |
| Morocco | 107.8 (99.0 to 117.3) | 83.5 (76.5 to 91.9) | -0.9 (-1.0 to -0.8) | 28.2 (19.0 to 36.8) | 15.2 (10.6 to 20.0) | -2.1 (-2.2 to -1.9) |
| Mozambique | 198.2 (91.3 to 446.8) | 138.6 (76.1 to 288.0) | -1.2 (-1.3 to -1.1) | 64.7 (27.6 to 144.5) | 39.2 (19.7 to 80.5) | -1.6 (-1.7 to -1.5) |
| Myanmar | 109.2 (84.0 to 181.4) | 114.7 (81.1 to 182.0) | 0.4 (0.1 to 0.6) | 34.5 (21.3 to 64.8) | 27.0 (15.7 to 44.7) | -0.7 (-0.9 to -0.5) |
| Namibia | 126.4 (86.4 to 210.5) | 97.2 (75.1 to 143.5) | -0.9 (-1.0 to -0.8) | 36.7 (21.3 to 63.3) | 21.7 (14.0 to 33.1) | -1.8 (-2.0 to -1.6) |
| Nauru | 75.9 (69.9 to 81.7) | 70.3 (64.6 to 76.6) | -0.3 (-0.4 to -0.2) | 20.9 (14.8 to 27.5) | 16.7 (11.6 to 22.1) | -0.8 (-1.0 to -0.5) |
| Nepal | 71.4 (64.7 to 80.2) | 94.2 (74.1 to 127.2) | 1.2 (1.0 to 1.4) | 21.9 (14.7 to 29.7) | 21.3 (13.6 to 30.8) | 0.2 (0.0 to 0.4) |
| Netherlands | 52.5 (47.1 to 59.4) | 48.8 (42.7 to 56.3) | -0.2 (-0.5 to 0.0) | 4.4 (2.8 to 6.2) | 4.0 (2.6 to 5.8) | -0.3 (-0.5 to 0.0) |
| New Zealand | 118.9 (106.5 to 135.4) | 103.4 (91.2 to 121.2) | -0.6 (-0.8 to -0.3) | 11.0 (7.3 to 15.1) | 8.7 (5.8 to 12.3) | -0.8 (-1.1 to -0.6) |
| Nicaragua | 377.2 (173.8 to 838.4) | 240.8 (127.9 to 497.1) | -1.5 (-1.5 to -1.4) | 94.3 (40.8 to 212.0) | 41.2 (20.5 to 85.2) | -2.8 (-2.9 to -2.7) |
| Niger | 65.8 (61.4 to 70.5) | 63.1 (57.1 to 71.1) | -0.3 (-0.3 to -0.2) | 21.4 (14.5 to 28.7) | 18.1 (12.7 to 24.3) | -0.7 (-0.8 to -0.7) |
| Nigeria | 63.9 (59.8 to 68.4) | 58.2 (52.5 to 65.7) | -0.4 (-0.5 to -0.3) | 18.6 (13.2 to 24.1) | 13.9 (9.7 to 18.3) | -1.0 (-1.1 to -0.9) |
| Niue | 61.8 (57.1 to 67.4) | 57.6 (51.7 to 65.9) | -0.2 (-0.3 to -0.1) | 14.7 (10.2 to 19.6) | 10.7 (7.3 to 14.6) | -1.0 (-1.1 to -0.9) |
| North Macedonia | 207.2 (189.0 to 230.1) | 192.5 (173.3 to 215.6) | -0.2 (-0.4 to -0.1) | 38.9 (27.3 to 50.7) | 20.8 (14.4 to 28.4) | -2.4 (-2.5 to -2.3) |
| Northern Mariana Islands | 84.0 (78.2 to 90.6) | 70.5 (64.7 to 77.8) | -0.6 (-0.7 to -0.6) | 15.5 (11.1 to 21.0) | 11.0 (7.7 to 14.8) | -0.9 (-1.0 to -0.8) |
| Norway | 77.5 (70.2 to 86.5) | 57.3 (51.3 to 66.7) | -1.0 (-1.1 to -0.8) | 7.0 (4.6 to 9.7) | 4.7 (3.1 to 6.8) | -1.1 (-1.2 to -0.9) |
| Oman | 135.5 (126.0 to 145.8) | 96.8 (88.5 to 106.0) | -1.2 (-1.3 to -1.1) | 28.2 (19.6 to 36.7) | 10.2 (6.9 to 13.7) | -3.3 (-3.4 to -3.1) |
| Pakistan | 56.1 (52.3 to 60.7) | 68.2 (55.7 to 89.8) | 0.9 (0.7 to 1.0) | 16.5 (11.8 to 21.9) | 17.1 (11.5 to 24.5) | 0.3 (0.2 to 0.4) |
| Palau | 88.0 (81.4 to 95.0) | 85.9 (78.7 to 93.5) | -0.1 (-0.1 to -0.1) | 20.0 (14.4 to 26.3) | 15.8 (11.0 to 20.9) | -0.7 (-0.7 to -0.7) |
| Palestine | 293.5 (157.5 to 548.6) | 348.5 (166.5 to 722.7) | 1.2 (0.9 to 1.5) | 66.2 (31.4 to 128.3) | 50.1 (22.2 to 105.9) | -0.2 (-0.6 to 0.2) |
| Panama | 123.5 (108.1 to 158.8) | 107.9 (96.5 to 128.1) | -0.4 (-0.5 to -0.4) | 26.0 (18.4 to 37.7) | 15.0 (10.4 to 21.0) | -1.6 (-1.7 to -1.5) |
| Papua New Guinea | 58.3 (54.2 to 63.1) | 75.1 (66.3 to 88.4) | 0.8 (0.6 to 0.9) | 16.6 (11.3 to 21.7) | 20.0 (13.6 to 26.6) | 0.6 (0.5 to 0.8) |
| Paraguay | 100.0 (90.6 to 112.1) | 90.7 (82.7 to 100.6) | -0.4 (-0.5 to -0.3) | 23.2 (15.8 to 31.5) | 15.8 (11.4 to 21.6) | -1.3 (-1.4 to -1.2) |
| Peru | 121.4 (101.3 to 161.4) | 104.0 (87.7 to 135.9) | -0.6 (-0.7 to -0.6) | 31.8 (21.2 to 46.3) | 14.7 (9.9 to 21.2) | -2.7 (-2.9 to -2.6) |
| Philippines | 108.2 (87.3 to 151.1) | 82.4 (64.6 to 113.1) | -0.9 (-1.0 to -0.9) | 27.0 (17.7 to 41.4) | 18.1 (11.8 to 27.4) | -1.3 (-1.3 to -1.2) |
| Poland | 242.0 (222.3 to 266.1) | 180.2 (164.4 to 202.3) | -1.0 (-1.1 to -0.9) | 42.6 (30.3 to 55.8) | 15.4 (10.1 to 21.4) | -3.4 (-3.5 to -3.3) |
| Portugal | 88.6 (78.7 to 99.5) | 51.0 (45.1 to 58.3) | -2.0 (-2.1 to -1.9) | 12.0 (8.3 to 16.0) | 4.2 (2.7 to 6.0) | -3.4 (-3.7 to -3.1) |
| Puerto Rico | 101.4 (94.3 to 110.3) | 103.7 (94.4 to 117.8) | 0.0 (-0.1 to 0.1) | 16.8 (12.0 to 22.6) | 9.4 (6.1 to 13.3) | -2.2 (-2.3 to -2.1) |
| Qatar | 124.4 (113.6 to 137.6) | 99.1 (89.1 to 109.6) | -0.6 (-0.8 to -0.5) | 22.9 (15.6 to 30.0) | 8.1 (5.2 to 11.3) | -3.6 (-3.9 to -3.3) |
| Republic of Korea | 90.1 (82.3 to 100.3) | 56.4 (50.7 to 63.8) | -1.8 (-1.9 to -1.7) | 15.9 (11.2 to 21.3) | 4.7 (3.1 to 6.6) | -4.1 (-4.5 to -3.7) |
| Republic of Moldova | 209.9 (194.1 to 228.9) | 136.2 (125.3 to 148.7) | -1.6 (-1.7 to -1.5) | 39.7 (27.7 to 52.1) | 17.2 (12.0 to 23.2) | -2.9 (-3.1 to -2.7) |
| Romania | 283.4 (258.9 to 316.1) | 211.2 (193.0 to 236.3) | -1.1 (-1.1 to -1.0) | 55.0 (38.0 to 72.8) | 20.8 (13.9 to 28.3) | -3.4 (-3.5 to -3.3) |
| Russian Federation | 269.1 (249.4 to 292.0) | 187.3 (172.0 to 206.7) | -1.5 (-1.8 to -1.3) | 45.0 (32.0 to 59.0) | 18.5 (12.6 to 25.1) | -3.5 (-4.1 to -3.0) |
| Rwanda | 66.6 (62.0 to 71.8) | 382.2 (138.7 to 914.4) | 3.2 (0.9 to 5.5) | 22.6 (15.5 to 29.9) | 99.1 (33.8 to 238.3) | 2.1 (-0.2 to 4.5) |
| Saint Kitts and Nevis | 114.3 (105.0 to 124.7) | 123.1 (111.6 to 134.6) | 0.5 (0.3 to 0.6) | 30.1 (20.2 to 39.8) | 21.6 (15.1 to 28.7) | -0.9 (-0.9 to -0.8) |
| Saint Lucia | 89.1 (83.3 to 96.2) | 95.7 (88.5 to 104.1) | 0.3 (0.1 to 0.4) | 21.2 (14.8 to 27.9) | 16.4 (11.5 to 21.9) | -0.8 (-0.8 to -0.7) |
| Saint Vincent and the Grenadines | 93.2 (86.6 to 100.7) | 101.4 (93.9 to 110.3) | 0.3 (0.1 to 0.4) | 22.1 (15.0 to 29.4) | 19.4 (13.8 to 25.8) | -0.4 (-0.5 to -0.3) |
| Samoa | 62.0 (57.4 to 66.9) | 69.7 (58.0 to 93.3) | 0.6 (0.4 to 0.8) | 15.7 (10.6 to 20.7) | 14.5 (9.5 to 20.9) | 0.0 (-0.2 to 0.2) |
| San Marino | 63.7 (56.6 to 75.1) | 60.6 (53.3 to 72.3) | -0.1 (-0.3 to 0.0) | 5.3 (3.4 to 7.6) | 5.0 (3.2 to 7.2) | -0.1 (-0.3 to 0.0) |
| Sao Tome and Principe | 61.6 (56.9 to 67.7) | 61.2 (56.3 to 67.1) | 0.0 (-0.1 to 0.0) | 17.4 (12.1 to 23.6) | 13.3 (9.4 to 17.6) | -0.9 (-0.9 to -0.8) |
| Saudi Arabia | 172.4 (157.9 to 188.0) | 174.3 (157.0 to 192.7) | 0.3 (0.1 to 0.6) | 40.9 (27.9 to 54.3) | 20.5 (14.1 to 27.4) | -2.1 (-2.3 to -1.9) |
| Senegal | 50.9 (47.3 to 55.1) | 48.6 (44.3 to 54.1) | -0.2 (-0.3 to -0.1) | 15.2 (10.4 to 20.2) | 12.2 (8.4 to 16.3) | -0.7 (-0.7 to -0.6) |
| Serbia | 226.9 (208.0 to 251.5) | 210.7 (183.3 to 251.2) | -0.2 (-0.3 to 0.0) | 39.1 (27.4 to 51.4) | 18.0 (11.6 to 25.3) | -2.8 (-3.0 to -2.5) |
| Seychelles | 74.8 (68.8 to 82.4) | 58.4 (53.7 to 63.5) | -0.8 (-0.9 to -0.7) | 17.3 (11.8 to 23.0) | 9.7 (6.7 to 12.9) | -1.8 (-1.9 to -1.7) |
| Sierra Leone | 55.1 (51.3 to 59.6) | 109.8 (67.5 to 197.2) | 1.6 (0.8 to 2.4) | 16.8 (11.5 to 22.0) | 29.2 (15.9 to 52.4) | 1.2 (0.4 to 2.1) |
| Singapore | 61.2 (54.8 to 68.4) | 52.5 (46.6 to 60.2) | -0.6 (-0.7 to -0.5) | 8.4 (5.8 to 11.3) | 4.4 (2.8 to 6.2) | -2.0 (-2.2 to -1.7) |
| Slovakia | 269.8 (245.1 to 300.1) | 220.2 (199.2 to 246.0) | -0.7 (-0.7 to -0.6) | 41.7 (28.9 to 55.4) | 18.1 (11.6 to 25.3) | -2.8 (-2.8 to -2.7) |
| Slovenia | 325.6 (293.4 to 363.0) | 247.2 (225.1 to 277.1) | -0.5 (-0.8 to -0.2) | 37.1 (25.7 to 50.1) | 20.3 (13.2 to 28.5) | -1.4 (-1.7 to -1.2) |
| Solomon Islands | 80.9 (73.7 to 89.4) | 77.3 (69.7 to 89.1) | -0.2 (-0.3 to -0.2) | 24.7 (17.2 to 32.2) | 20.5 (13.8 to 27.6) | -0.6 (-0.6 to -0.5) |
| Somalia | 97.8 (67.8 to 250.3) | 138.3 (85.3 to 263.8) | 0.7 (0.6 to 0.8) | 33.2 (18.1 to 93.6) | 44.4 (23.7 to 88.7) | 0.5 (0.4 to 0.6) |
| South Africa | 130.1 (117.5 to 146.7) | 83.5 (75.7 to 94.6) | -1.6 (-1.7 to -1.5) | 32.0 (22.6 to 42.7) | 16.9 (11.9 to 22.5) | -2.3 (-2.5 to -2.1) |
| South Sudan | 92.8 (70.2 to 138.8) | 141.2 (84.0 to 263.4) | 1.6 (1.4 to 1.9) | 28.8 (18.0 to 45.2) | 38.4 (20.5 to 70.6) | 1.2 (0.9 to 1.5) |
| Spain | 69.9 (62.8 to 78.4) | 59.5 (51.7 to 71.0) | -0.6 (-0.7 to -0.5) | 6.5 (4.3 to 9.2) | 4.9 (3.1 to 7.1) | -0.7 (-0.8 to -0.6) |
| Sri Lanka | 130.7 (101.3 to 188.9) | 210.5 (119.9 to 395.4) | 1.4 (1.1 to 1.7) | 30.1 (19.6 to 47.5) | 28.3 (14.5 to 53.9) | -0.5 (-0.8 to -0.1) |
| Sudan | 106.6 (91.7 to 136.9) | 127.6 (91.7 to 202.0) | 0.7 (0.5 to 0.9) | 30.7 (20.2 to 42.3) | 26.6 (16.0 to 42.7) | -0.4 (-0.6 to -0.1) |
| Suriname | 102.6 (83.3 to 172.8) | 95.2 (81.9 to 134.1) | -0.2 (-0.3 to -0.1) | 27.0 (17.2 to 47.5) | 20.3 (13.4 to 29.4) | -0.9 (-1.0 to -0.8) |
| Sweden | 70.6 (63.1 to 81.0) | 56.2 (49.8 to 65.2) | -0.7 (-0.9 to -0.6) | 5.8 (3.8 to 8.4) | 4.6 (3.0 to 6.7) | -0.7 (-0.9 to -0.6) |
| Switzerland | 101.0 (88.7 to 117.3) | 74.2 (64.6 to 88.2) | -1.3 (-1.5 to -1.1) | 8.3 (5.4 to 12.0) | 6.1 (3.9 to 8.8) | -1.3 (-1.5 to -1.1) |
| Syrian Arab Republic | 99.6 (77.3 to 162.2) | 466.4 (181.3 to 1065.6) | 5.1 (3.7 to 6.6) | 23.2 (14.5 to 40.0) | 64.7 (24.3 to 148.5) | 3.3 (1.7 to 4.9) |
| Taiwan (Province of China) | 71.3 (66.3 to 77.4) | 44.1 (40.6 to 48.6) | -1.9 (-2.1 to -1.7) | 10.1 (7.0 to 13.6) | 3.6 (2.4 to 5.2) | -3.7 (-3.9 to -3.5) |
| Tajikistan | 130.6 (119.7 to 143.3) | 153.4 (113.3 to 239.5) | -0.4 (-0.8 to 0.2) | 32.5 (22.7 to 42.6) | 33.8 (21.0 to 53.5) | -0.7 (-1.3 to -0.2) |
| Thailand | 98.0 (91.4 to 105.7) | 74.2 (67.9 to 81.7) | -1.2 (-1.2 to -1.1) | 21.0 (14.8 to 28.1) | 9.2 (6.4 to 12.5) | -3.0 (-3.1 to -2.9) |
| Timor-Leste | 280.7 (128.1 to 594.8) | 309.2 (128.0 to 679.3) | 0.2 (-0.3 to 0.7) | 87.6 (38.0 to 183.6) | 75.7 (30.9 to 165.7) | -0.7 (-1.1 to -0.2) |
| Togo | 58.4 (54.6 to 63.4) | 54.5 (50.8 to 59.0) | -0.3 (-0.3 to -0.2) | 17.1 (11.9 to 22.8) | 14.0 (9.8 to 18.5) | -0.7 (-0.8 to -0.6) |
| Tokelau | 58.1 (53.9 to 63.0) | 50.0 (45.9 to 55.6) | -0.5 (-0.6 to -0.4) | 15.4 (10.6 to 20.5) | 9.6 (6.7 to 12.8) | -1.5 (-1.6 to -1.4) |
| Tonga | 52.2 (48.2 to 56.8) | 39.7 (36.4 to 44.0) | -0.8 (-0.9 to -0.8) | 12.3 (8.7 to 16.3) | 7.9 (5.5 to 10.8) | -1.3 (-1.4 to -1.3) |
| Trinidad and Tobago | 85.2 (79.1 to 92.4) | 97.3 (87.8 to 110.0) | 0.6 (0.4 to 0.8) | 20.0 (13.8 to 26.7) | 16.9 (11.7 to 22.8) | -0.6 (-0.7 to -0.4) |
| Tunisia | 117.2 (107.9 to 131.5) | 93.9 (85.4 to 104.7) | -0.7 (-0.8 to -0.7) | 23.8 (16.5 to 31.6) | 11.2 (7.7 to 15.1) | -2.5 (-2.5 to -2.4) |
| Turkey | 86.9 (80.8 to 94.0) | 76.2 (68.0 to 88.3) | -0.5 (-0.5 to -0.4) | 20.9 (14.3 to 27.1) | 8.7 (5.8 to 12.1) | -3.0 (-3.1 to -2.9) |
| Turkmenistan | 128.8 (118.2 to 142.7) | 118.5 (107.9 to 130.8) | -0.3 (-0.4 to -0.3) | 30.8 (21.7 to 41.6) | 22.4 (15.7 to 29.8) | -1.1 (-1.2 to -1.1) |
| Tuvalu | 67.1 (61.7 to 72.8) | 60.6 (55.1 to 66.3) | -0.9 (-1.2 to -0.7) | 20.2 (13.9 to 26.6) | 14.1 (9.8 to 18.7) | -1.7 (-1.9 to -1.5) |
| Uganda | 163.7 (84.1 to 340.6) | 117.9 (68.0 to 223.4) | -1.1 (-1.2 to -1.0) | 49.6 (23.3 to 108.1) | 30.0 (15.7 to 59.1) | -1.7 (-1.9 to -1.5) |
| Ukraine | 221.0 (201.1 to 244.7) | 195.5 (177.9 to 215.1) | -0.8 (-1.0 to -0.7) | 34.3 (24.3 to 45.5) | 24.9 (17.2 to 33.5) | -1.6 (-1.9 to -1.4) |
| United Arab Emirates | 118.2 (108.2 to 128.7) | 107.2 (97.6 to 117.9) | -0.3 (-0.4 to -0.3) | 26.6 (17.8 to 35.2) | 15.3 (10.5 to 20.0) | -1.8 (-1.9 to -1.7) |
| United Kingdom | 62.1 (56.2 to 71.2) | 51.7 (46.1 to 59.5) | -0.6 (-0.8 to -0.4) | 6.1 (4.1 to 8.4) | 4.3 (2.8 to 6.1) | -1.1 (-1.2 to -1.0) |
| United Republic of Tanzania | 59.5 (55.5 to 63.9) | 53.2 (49.4 to 57.2) | -0.4 (-0.4 to -0.3) | 17.6 (12.0 to 23.0) | 13.7 (9.2 to 18.2) | -0.8 (-0.9 to -0.7) |
| United States of America | 64.2 (59.3 to 71.1) | 47.7 (43.9 to 51.7) | -1.2 (-1.4 to -0.9) | 5.6 (3.8 to 7.8) | 3.9 (2.5 to 5.4) | -1.3 (-1.6 to -1.1) |
| United States Virgin Islands | 105.2 (97.2 to 114.0) | 90.9 (83.6 to 99.3) | -0.8 (-1.0 to -0.5) | 21.0 (15.0 to 28.0) | 13.6 (9.6 to 18.4) | -1.7 (-1.9 to -1.5) |
| Uruguay | 76.8 (70.0 to 84.4) | 69.1 (63.7 to 76.6) | -0.5 (-0.5 to -0.4) | 13.9 (9.5 to 18.4) | 8.2 (5.6 to 11.0) | -1.8 (-1.8 to -1.7) |
| Uzbekistan | 125.6 (115.9 to 137.7) | 104.9 (96.7 to 115.8) | -0.6 (-0.7 to -0.5) | 28.4 (19.7 to 38.3) | 19.8 (13.9 to 26.4) | -1.4 (-1.5 to -1.2) |
| Vanuatu | 58.8 (53.9 to 67.1) | 53.9 (48.6 to 63.6) | -0.3 (-0.4 to -0.2) | 16.8 (11.3 to 22.5) | 14.2 (9.5 to 19.4) | -0.5 (-0.7 to -0.4) |
| Venezuela (Bolivarian Republic of) | 151.8 (139.2 to 169.8) | 156.4 (143.5 to 173.0) | 0.2 (0.0 to 0.3) | 35.7 (24.8 to 46.8) | 26.7 (18.6 to 35.8) | -0.9 (-1.1 to -0.8) |
| Viet Nam | 73.8 (68.7 to 79.1) | 67.5 (62.7 to 73.0) | -0.2 (-0.2 to -0.1) | 18.2 (12.5 to 24.5) | 10.3 (7.0 to 14.0) | -1.8 (-1.8 to -1.7) |
| Yemen | 112.1 (95.7 to 144.8) | 172.3 (110.4 to 303.0) | 0.9 (0.5 to 1.3) | 32.1 (20.5 to 45.3) | 40.0 (22.7 to 71.4) | 0.1 (-0.3 to 0.5) |
| Zambia | 58.7 (54.8 to 62.8) | 55.5 (51.7 to 59.5) | -0.2 (-0.4 to -0.1) | 18.5 (13.0 to 24.8) | 14.4 (9.8 to 18.8) | -0.9 (-1.1 to -0.7) |
| Zimbabwe | 60.4 (56.6 to 64.0) | 58.0 (53.9 to 61.8) | -0.2 (-0.3 to -0.1) | 16.1 (11.3 to 21.4) | 15.8 (11.1 to 20.7) | 0.2 (0.0 to 0.3) |

**Table S19. Global cases and ASR of prevalence and YLDs in 2021, and their EAPC from 1990 to 2021 for 7 amputation subtypes.**

| **Characteristics** | **Prevalence** | | | **YLDs** | | |
| --- | --- | --- | --- | --- | --- | --- |
|  | **Cases No. (95% UI)** | **ASR per 100,000 No. (95% UI)** | **EAPC No. (95% CI)** | **Cases No. (95% UI)** | **ASR per 100,000 No. (95% UI)** | **EAPC No. (95% CI)** |
| AMP-F | 223793376.6 (204337382.6 to 246011335.7) | 2681.1 (2446.5 to 2946.1) | -1.0 (-1.0 to -1.0) | 1019501.6 (369661.0 to 2253382.8) | 12.2 (4.4 to 27.0) | 1.0 (-1.0 to -0.9) |
| AMP-LL1 | 19271753.1 (17546973.8 to 21791173.5) | 227.7 (206.9 to 257.2) | -0.4 (-0.4 to -0.3) | 1231468.3 (845240.7 to 1675869.5) | 14.6 (10.0 to 19.9) | -1.6 (-1.8 to -1.5) |
| AMP-LL2 | 3795491.8 (3169630.2 to 4964859.5) | 45.4 (37.9 to 59.4) | -0.8 (-0.8 to -0.7) | 628584.6 (409123.0 to 966723.6) | 7.6 (4.9 to 11.6) | -1.4 (-1.5 to -1.3) |
| AMP-Th | 99830660.7 (90285572.0 to 112852542.0) | 1197.2 (1081.7 to 1355.4) | -1.2 (-1.2 to -1.1) | 1093176.4 (520575.9 to 2020114.6) | 13.1 (6.2 to 24.3) | -1.2 (-1.2 to -1.1) |
| AMP-Toe | 84609371.9 (75613661.6 to 96295253.5) | 1015.5 (907.0 to 1156.8) | -1.0 (-1.0 to -1.0) | 475576.6 (190824.6 to 998735.3) | 5.7 (2.3 to 12.0) | -1.0 (-1.0 to -0.9) |
| AMP-UL1 | 6799995.8 (6034872.3 to 8169092.7) | 81.2 (72.0 to 97.4) | -0.7 (-0.8 to -0.7) | 364149.0 (239439.6 to 525869.3) | 4.4 (2.9 to 6.3) | -1.5 (-1.6 to -1.4) |
| AMP-UL2 | 7139997.2 (6309192.6 to 8588696.2) | 85.4 (75.4 to 102.9) | -0.8 (-0.8 to -0.7) | 1123374.3 (779923.9 to 1558792.5) | 13.5 (9.4 to 18.8) | -1.8 (-1.9 to -1.7) |

# Table S20. Projected ASR of prevalence and YLDs from 2022 to 2050 for total amputations.

| **Year** | **Prevalence** | | **YLDs** | |
| --- | --- | --- | --- | --- |
|  | **Age-standardized prevalence rate per 100,000** | **95%UI** | **Age-standardized YLDs rate per 100,000** | **95%UI** |
| 2022 | 5330.3 | 5201.4 to 5459.1 | 71.1 | 68.7 to 73.4 |
| 2023 | 5317.8 | 5152.6 to 5483 | 70.9 | 68.2 to 73.7 |
| 2024 | 5306.6 | 5085.3 to 5527.9 | 70.8 | 67.3 to 74.4 |
| 2025 | 5296.6 | 5004.2 to 5588.9 | 70.8 | 66.3 to 75.2 |
| 2026 | 5285.3 | 4910.5 to 5660.1 | 70.6 | 65 to 76.3 |
| 2027 | 5273.0 | 4805.7 to 5740.2 | 70.5 | 63.6 to 77.4 |
| 2028 | 5260.4 | 4692.7 to 5828.1 | 70.4 | 62 to 78.7 |
| 2029 | 5249.9 | 4574.4 to 5925.5 | 70.3 | 60.4 to 80.1 |
| 2030 | 5241.2 | 4451.2 to 6031.3 | 70.2 | 58.7 to 81.7 |
| 2031 | 5231.7 | 4321 to 6142.3 | 70.1 | 56.8 to 83.3 |
| 2032 | 5221.2 | 4183.7 to 6258.6 | 70.0 | 54.9 to 85 |
| 2033 | 5211.0 | 4041 to 6381 | 69.8 | 52.9 to 86.8 |
| 2034 | 5203.6 | 3894.9 to 6512.2 | 69.8 | 50.8 to 88.7 |
| 2035 | 5198.5 | 3745.3 to 6651.7 | 69.7 | 48.7 to 90.8 |
| 2036 | 5192.9 | 3589.7 to 6796 | 69.7 | 46.5 to 92.9 |
| 2037 | 5186.5 | 3427.9 to 6945.1 | 69.6 | 44.2 to 95 |
| 2038 | 5180.9 | 3261.1 to 7100.8 | 69.5 | 41.8 to 97.3 |
| 2039 | 5178.8 | 3090.9 to 7266.7 | 69.5 | 39.3 to 99.7 |
| 2040 | 5179.6 | 2916.8 to 7442.4 | 69.6 | 36.8 to 102.3 |
| 2041 | 5180.3 | 2736.6 to 7624 | 69.6 | 34.2 to 105 |
| 2042 | 5180.7 | 2549.8 to 7811.7 | 69.6 | 31.5 to 107.7 |
| 2043 | 5182.5 | 2357.1 to 8007.9 | 69.6 | 28.7 to 110.6 |
| 2044 | 5188.4 | 2159.6 to 8217.2 | 69.7 | 25.8 to 113.7 |
| 2045 | 5197.9 | 1956.4 to 8439.3 | 69.9 | 22.8 to 116.9 |
| 2046 | 5207.9 | 1745.6 to 8670.2 | 70.0 | 19.7 to 120.3 |
| 2047 | 5218.1 | 1527.3 to 8909.9 | 70.1 | 16.5 to 123.9 |
| 2048 | 5230.5 | 1301.4 to 9162.1 | 70.3 | 13.2 to 127.6 |
| 2049 | 5247.6 | 1069.5 to 9432.1 | 70.6 | 9.8 to 131.6 |
| 2050 | 5269.1 | 831.9 to 9720.1 | 70.9 | 6.3 to 135.9 |

# Table S21. Rankings, ASR of prevalence, and number of cases of total amputation by injury type (1990–2021), with percentage changes in absolute cases (1990–2021).

| **1990** | | | **2000** | | | **2010** | | | **2021** | | | **Percentage change (1990-2021)** |
| --- | --- | --- | --- | --- | --- | --- | --- | --- | --- | --- | --- | --- |
| **Rank** | **ASR** | **Cases** | **Rank** | **ASR** | **Cases** | **Rank** | **ASR** | **Cases** | **Rank** | **ASR** | **Cases** |  |
| Falls | 3976.9 | 194227216.4 | Falls | 3944.7 | 230225077.4 | Falls | 4844.4 | 339302095.6 | Falls | 3682.4 | 309130476.3 | 59.2% |
| Exposure to mechanical forces | 2748.1 | 129654514.2 | Exposure to mechanical forces | 2394.8 | 136703755.5 | Exposure to mechanical forces | 2171.5 | 150632384.4 | Exposure to mechanical forces | 1817.6 | 153031567.4 | 18.0% |
| Other unintentional injuries | 18 37.4 | 89217124.5 | Other unintentional injuries | 1570.5 | 91662813.6 | Other unintentional injuries | 1393.3 | 97778528.5 | Other unintentional injuries | 1127.1 | 94363840.4 | 5.8% |
| Interpersonal violence | 524.9 | 25619812.7 | Road injuries | 449.6 | 25783424.5 | Interpersonal violence | 384.4 | 26815278.4 | Conflict and terrorism | 463.1 | 37486282.6 | 230.1% |
| Road injuries | 504.2 | 24060228.7 | Interpersonal violence | 443.6 | 25768462.2 | Road injuries | 373.8 | 25936951.0 | Road injuries | 307.4 | 25993047.0 | 8.0% |
| Animal contact | 325.6 | 15852103.1 | Conflict and terrorism | 310.6 | 19883491.6 | Conflict and terrorism | 339.6 | 24529938.8 | Interpersonal violence | 302.9 | 25285507.4 | -1.3% |
| Foreign body | 223.4 | 12549752.8 | Animal contact | 294.6 | 17220854.0 | Animal contact | 253.8 | 17743263.6 | Animal contact | 223.2 | 18588321.0 | 17.3% |
| Conflict and terrorism | 217.9 | 11355083.0 | Foreign body | 201.4 | 12071963.5 | Foreign body | 185.2 | 12961502.9 | Foreign body | 176.1 | 14332321.3 | 14.2% |
| Environmental heat and cold exposure | 70.7 | 3407082.3 | Fire, heat, and hot substances | 62.4 | 3631674.9 | Fire, heat, and hot substances | 59.3 | 4152087.5 | Police conflict and executions | 101.8 | 7951921.1 | 791.2% |
| Other transport injuries | 66.7 | 3230337.5 | Other transport injuries | 61.9 | 3576386.3 | Other transport injuries | 54.6 | 3793981.4 | Fire, heat, and hot substances | 55.1 | 4599582.8 | 45.4% |
| Fire, heat, and hot substances | 65.7 | 3163261.3 | Self-harm | 58.8 | 3397079.9 | Environmental heat and cold exposure | 52.4 | 3654370.2 | Other transport injuries | 52.9 | 4423657.3 | 36.9% |
| Self-harm | 60.3 | 2754632.2 | Environmental heat and cold exposure | 58.6 | 3297272.0 | Self-harm | 49.0 | 3396313.2 | Environmental heat and cold exposure | 45.5 | 3790453.2 | 11.3% |
| Drowning | 18.1 | 938580.7 | Police conflict and executions | 27.0 | 1694797.3 | Police conflict and executions | 40.9 | 2903368.8 | Self-harm | 42.6 | 3627168.2 | 31.7% |
| Police conflict and executions | 16.9 | 892307.1 | Exposure to forces of nature | 26.4 | 1694276.2 | Exposure to forces of nature | 38.5 | 2722959.7 | Exposure to forces of nature | 37.9 | 3041920.0 | 352.1% |
| Poisonings | 14.5 | 693024.0 | Drowning | 15.0 | 869789.4 | Poisonings | 11.4 | 791300.9 | Poisonings | 9.0 | 751835.9 | 8.5% |
| Exposure to forces of nature | 11.6 | 672875.3 | Poisonings | 12.1 | 699998.3 | Drowning | 11.0 | 761528.7 | Drowning | 8.4 | 698255.1 | -25.6% |
| Electrocution | 5.3 | 246146.9 | Electrocution | 4.7 | 268187.2 | Electrocution | 4.2 | 290138.7 | Electrocution | 3.5 | 294157.7 | 19.5% |
| Adverse effects of medical treatment | 0.0 | 0.0 | Adverse effects of medical treatment | 0.0 | 0.0 | Adverse effects of medical treatment | 0.0 | 0.0 | Adverse effects of medical treatment | 0.0 | 0.0 | 0.0% |

# Table S22. Rankings, ASR of prevalence, and number of cases of AMP-F by injury type (1990–2021), with percentage changes in absolute cases (1990–2021).

| **1990** | | | **2000** | | | **2010** | | | **2021** | | | **Percentage change (1990-2021)** |
| --- | --- | --- | --- | --- | --- | --- | --- | --- | --- | --- | --- | --- |
| **Rank** | **ASR** | **Cases** | **Rank** | **ASR** | **Cases** | **Rank** | **ASR** | **Cases** | **Rank** | **ASR** | **Cases** |  |
| Falls | 1706.2 | 83985617.1 | Falls | 1692.6 | 99406080.6 | Falls | 2107.1 | 148038574.2 | Falls | 1607.1 | 134799343.6 | 60.5% |
| Exposure to mechanical forces | 1638.7 | 77372129.0 | Exposure to mechanical forces | 1436.0 | 82032301.0 | Exposure to mechanical forces | 1309.8 | 90905244.9 | Exposure to mechanical forces | 1098.8 | 92530282.1 | 19.6% |
| Other unintentional injuries | 1074.0 | 52433418.6 | Other unintentional injuries | 919.0 | 53884634.0 | Other unintentional injuries | 818.9 | 57604683.0 | Other unintentional injuries | 662.2 | 55393630.0 | 5.6% |
| Interpersonal violence | 255.3 | 12523256.6 | Interpersonal violence | 215.8 | 12585494.1 | Interpersonal violence | 187.3 | 13088851.3 | Conflict and terrorism | 213.5 | 17240968.5 | 205.2% |
| Road injuries | 184.8 | 8907395.4 | Road injuries | 166.7 | 9630123.2 | Conflict and terrorism | 155.6 | 11238710.4 | Interpersonal violence | 147.9 | 12328774.6 | -1.6% |
| Animal contact | 165.7 | 8071879.4 | Conflict and terrorism | 150.7 | 8997771.1 | Road injuries | 139.9 | 9748462.0 | Road injuries | 115.5 | 9753776.4 | 9.5% |
| Conflict and terrorism | 97.8 | 5649483.6 | Animal contact | 140.2 | 8814926.2 | Animal contact | 129.6 | 9068765.3 | Animal contact | 113.7 | 9474049.8 | 17.4% |
| Foreign body | 88.6 | 4532113.6 | Foreign body | 80.5 | 4843506.1 | Foreign body | 74.2 | 5203236.0 | Foreign body | 70.5 | 5723116.2 | 26.3% |
| Environmental heat and cold exposure | 37.8 | 1842410.4 | Fire, heat, and hot substances | 33.8 | 1977546.4 | Fire, heat, and hot substances | 32.4 | 2274983.4 | Police conflict and executions | 49.6 | 3864813.2 | 758.1% |
| Fire, heat, and hot substances | 35.4 | 1714128.9 | Environmental heat and cold exposure | 31.2 | 1824462.0 | Environmental heat and cold exposure | 27.7 | 1942778.4 | Fire, heat, and hot substances | 30.3 | 2526869.5 | 47.4% |
| Self-harm | 21.0 | 974530.7 | Self-harm | 20.6 | 1164701.3 | Police conflict and executions | 19.6 | 1389086.3 | Environmental heat and cold exposure | 24.1 | 1998725.5 | 8.5% |
| Other transport injuries | 19.8 | 971457.6 | Other transport injuries | 18.3 | 1070450.5 | Exposure to forces of nature | 19.2 | 1360483.1 | Exposure to forces of nature | 18.9 | 1512123.7 | 342.7% |
| Police conflict and executions | 8.0 | 450399.3 | Exposure to forces of nature | 13.5 | 869910.7 | Self-harm | 17.3 | 1202267.1 | Self-harm | 15.2 | 1283260.7 | 31.7% |
| Drowning | 7.6 | 373529.9 | Police conflict and executions | 12.8 | 808903.0 | Other transport injuries | 15.8 | 1103544.3 | Other transport injuries | 15.1 | 1266250.2 | 30.3% |
| Exposure to forces of nature | 5.8 | 341545.5 | Drowning | 6.3 | 365131.7 | Drowning | 4.6 | 318475.4 | Drowning | 3.5 | 292791.9 | -21.6% |
| Poisonings | 4.4 | 209894.6 | Poisonings | 3.7 | 216471.1 | Poisonings | 3.5 | 246002.2 | Poisonings | 2.8 | 233246.9 | 11.1% |
| Electrocution | 2.5 | 115666.4 | Electrocution | 2.2 | 125450.4 | Electrocution | 1.9 | 134520.2 | Electrocution | 1.6 | 136112.5 | 17.7% |
| Adverse effects of medical treatment | 0.0 | 0.0 | Adverse effects of medical treatment | 0.0 | 0.0 | Adverse effects of medical treatment | 0.0 | 0.0 | Adverse effects of medical treatment | 0.0 | 0.0 | 0.0% |

# Table S23. Rankings, ASR of prevalence, and number of cases of AMP-LL2 by injury type (1990–2021), with percentage changes in absolute cases (1990–2021).

| **1990** | | | **2000** | | | **2010** | | | **2021** | | | **Percentage change**  **(1990-2021)** |
| --- | --- | --- | --- | --- | --- | --- | --- | --- | --- | --- | --- | --- |
| **Rank** | **ASR** | **Cases** | **Rank** | **ASR** | **Cases** | **Rank** | **ASR** | **Cases** | **Rank** | **ASR** | **Cases** |  |
| Falls | 41.2 | 1959079.2 | Falls | 40.7 | 2328606.9 | Falls | 47.1 | 3267995.6 | Falls | 35.9 | 3028190.0 | 54.6% |
| Road injuries | 7.7 | 399051.8 | Conflict and terrorism | 9.8 | 633195.4 | Conflict and terrorism | 10.4 | 751862.4 | Conflict and terrorism | 14.0 | 1136932.3 | 216.3% |
| Conflict and terrorism | 6.8 | 359424.3 | Road injuries | 6.7 | 378800.9 | Road injuries | 5.4 | 372948.1 | Road injuries | 4.4 | 371025.6 | -7.0% |
| Interpersonal violence | 6.8 | 330174.5 | Interpersonal violence | 5.7 | 333812.0 | Interpersonal violence | 4.9 | 344756.9 | Interpersonal violence | 3.9 | 326523.2 | -1.1% |
| Other unintentional injuries | 5.2 | 243049.4 | Other unintentional injuries | 4.4 | 248645.1 | Other unintentional injuries | 3.8 | 258456.3 | Other unintentional injuries | 3.0 | 256220.2 | 5.4% |
| Exposure to mechanical forces | 4.5 | 210232.4 | Exposure to mechanical forces | 3.9 | 220371.0 | Exposure to mechanical forces | 3.5 | 240863.0 | Exposure to mechanical forces | 2.9 | 245630.7 | 16.8% |
| Other transport injuries | 3.1 | 149178.4 | Other transport injuries | 2.9 | 165403.5 | Other transport injuries | 2.6 | 178488.3 | Other transport injuries | 2.5 | 209399.2 | 40.4% |
| Self-harm | 2.8 | 129718.6 | Self-harm | 2.8 | 155002.8 | Self-harm | 2.3 | 160680.1 | Police conflict and executions | 2.5 | 196852.8 | 903.1% |
| Animal contact | 2.6 | 126309.5 | Animal contact | 2.4 | 137646.9 | Animal contact | 2.1 | 143491.4 | Self-harm | 2.0 | 170857.2 | 31.7% |
| Fire, heat, and hot substances | 0.7 | 32762.8 | Police conflict and executions | 0.6 | 39361.9 | Police conflict and executions | 1.0 | 69485.6 | Animal contact | 1.8 | 152761.3 | 20.9% |
| Poisonings | 0.5 | 27099.7 | Fire, heat, and hot substances | 0.6 | 37572.5 | Fire, heat, and hot substances | 0.6 | 41987.7 | Fire, heat, and hot substances | 0.5 | 45677.9 | 39.4% |
| Drowning | 0.5 | 24858.9 | Poisonings | 0.5 | 28130.2 | Poisonings | 0.4 | 30348.2 | Poisonings | 0.3 | 27719.5 | 2.3% |
| Environmental heat and cold exposure | 0.4 | 21835.5 | Drowning | 0.4 | 23081.5 | Environmental heat and cold exposure | 0.3 | 21930.9 | Environmental heat and cold exposure | 0.3 | 23296.9 | 6.7% |
| Police conflict and executions | 0.4 | 19624.4 | Environmental heat and cold exposure | 0.3 | 19736.6 | Drowning | 0.3 | 18922.9 | Drowning | 0.2 | 16327.3 | -34.3% |
| Electrocution | 0.1 | 5000.7 | Electrocution | 0.1 | 5485.2 | Electrocution | 0.1 | 6033.3 | Electrocution | 0.1 | 6161.4 | 23.2% |
| Exposure to forces of nature | 0.0 | 0.0 | Adverse effects of medical treatment | 0.0 | 0.0 | Adverse effects of medical treatment | 0.0 | 0.0 | Adverse effects of medical treatment | 0.0 | 0.0 | 0.0% |
| Adverse effects of medical treatment | 0.0 | 0.0 | Foreign body | 0.0 | 0.0 | Foreign body | 0.0 | 0.0 | Foreign body | 0.0 | 0.0 | 0.0% |
| Foreign body | 0.0 | 0.0 | Exposure to forces of nature | 0.0 | 0.0 | Exposure to forces of nature | 0.0 | 0.0 | Exposure to forces of nature | 0.0 | 0.0 | 0.0% |

# Table S24. Rankings, ASR of prevalence, and number of cases of AMP-LL1 by injury type (1990–2021), with percentage changes in absolute cases (1990–2021).

| **1990** | | | **2000** | | | **2010** | | | **2021** | | | **Percentage change**  **(1990-2021)** |
| --- | --- | --- | --- | --- | --- | --- | --- | --- | --- | --- | --- | --- |
| **Rank** | **ASR** | **Cases** | **Rank** | **ASR** | **Cases** | **Rank** | **ASR** | **Cases** | **Rank** | **ASR** | **Cases** |  |
| Falls | 149.5 | 6602296.3 | Falls | 155.5 | 8402650.8 | Falls | 190.9 | 12899009.1 | Falls | 173.3 | 14799383.9 | 124.2% |
| Road injuries | 54.2 | 2406725.3 | Road injuries | 53.8 | 2931343.3 | Road injuries | 50.2 | 3403969.5 | Road injuries | 44.3 | 3809935.5 | 58.3% |
| Other unintentional injuries | 26.1 | 1148736.5 | Conflict and terrorism | 24.4 | 1512562.4 | Conflict and terrorism | 24.2 | 1737922.2 | Conflict and terrorism | 31.3 | 2587603.0 | 170.7% |
| Interpersonal violence | 21.7 | 997609.7 | Other unintentional injuries | 22.7 | 1227071.2 | Other unintentional injuries | 19.7 | 1334480.5 | Exposure to mechanical forces | 16.2 | 1383043.7 | 43.1% |
| Exposure to mechanical forces | 21.5 | 966692.8 | Exposure to mechanical forces | 19.8 | 1078670.4 | Exposure to mechanical forces | 18.9 | 1281064.1 | Other unintentional injuries | 16.1 | 1376049.7 | 19.8% |
| Conflict and terrorism | 18.1 | 955983.8 | Interpersonal violence | 19.6 | 1067995.2 | Interpersonal violence | 18.1 | 1230189.5 | Interpersonal violence | 13.0 | 1111655.2 | 11.4% |
| Other transport injuries | 11.1 | 509145.7 | Other transport injuries | 10.5 | 585032.4 | Other transport injuries | 10.0 | 683219.4 | Other transport injuries | 9.9 | 835735.8 | 64.1% |
| Self-harm | 8.6 | 380151.8 | Self-harm | 8.3 | 454032.9 | Self-harm | 6.7 | 459564.1 | Self-harm | 5.8 | 497421.2 | 30.8% |
| Environmental heat and cold exposure | 5.7 | 265134.3 | Foreign body | 5.0 | 288190.7 | Foreign body | 4.5 | 309616.9 | Police conflict and executions | 5.1 | 414439.8 | 582.1% |
| Foreign body | 5.5 | 251459.3 | Environmental heat and cold exposure | 4.8 | 260452.3 | Environmental heat and cold exposure | 4.0 | 268729.9 | Foreign body | 4.3 | 361636.5 | 43.8% |
| Animal contact | 2.7 | 122830.3 | Animal contact | 2.4 | 134119.7 | Police conflict and executions | 2.6 | 181422.8 | Environmental heat and cold exposure | 3.2 | 272055.9 | 2.6% |
| Fire, heat, and hot substances | 1.8 | 79913.5 | Police conflict and executions | 1.9 | 113664.7 | Animal contact | 2.0 | 137495.2 | Exposure to forces of nature | 1.9 | 152115.2 | 424.2% |
| Police conflict and executions | 1.2 | 60759.1 | Fire, heat, and hot substances | 1.7 | 93202.3 | Exposure to forces of nature | 2.0 | 137321.1 | Animal contact | 1.7 | 144410.7 | 17.6% |
| Poisonings | 0.9 | 42232.8 | Exposure to forces of nature | 1.1 | 71134.4 | Fire, heat, and hot substances | 1.6 | 110035.4 | Fire, heat, and hot substances | 1.5 | 127307.5 | 59.3% |
| Drowning | 0.7 | 33885.2 | Poisonings | 0.8 | 46771.1 | Poisonings | 0.8 | 52631.8 | Poisonings | 0.6 | 50964.0 | 20.7% |
| Exposure to forces of nature | 0.5 | 29019.4 | Drowning | 0.6 | 33810.3 | Drowning | 0.5 | 30664.7 | Drowning | 0.4 | 30397.1 | -10.3% |
| Electrocution | 0.2 | 8993.4 | Electrocution | 0.2 | 9803.7 | Electrocution | 0.2 | 10636.6 | Electrocution | 0.1 | 10826.1 | 20.4% |
| Adverse effects of medical treatment | 0.0 | 0.0 | Adverse effects of medical treatment | 0.0 | 0.0 | Adverse effects of medical treatment | 0.0 | 0.0 | Adverse effects of medical treatment | 0.0 | 0.0 | 0.0% |

# Table S25. Rankings, ASR of prevalence, and number of cases of AMP-Th by injury type (1990–2021), with percentage changes in absolute cases (1990–2021).

| **1990** | | | **2000** | | | **2010** | | | **2021** | | | **Percentage change (1990-2021)** |
| --- | --- | --- | --- | --- | --- | --- | --- | --- | --- | --- | --- | --- |
| **Rank** | **ASR** | **Cases** | **Rank** | **ASR** | **Cases** | **Rank** | **ASR** | **Cases** | **Rank** | **ASR** | **Cases** |  |
| Falls | 994.2 | 48750309.0 | Falls | 979.7 | 57355517.2 | Falls | 1186.9 | 83206270.1 | Falls | 874.3 | 73230523.2 | 50.2% |
| Exposure to mechanical forces | 675.7 | 31739401.3 | Exposure to mechanical forces | 578.6 | 32897171.7 | Exposure to mechanical forces | 514.7 | 35640752.3 | Exposure to mechanical forces | 427.9 | 36053575.7 | 13.6% |
| Other unintentional injuries | 388.1 | 18857951.5 | Other unintentional injuries | 330.5 | 19279068.5 | Other unintentional injuries | 291.4 | 20431173.1 | Other unintentional injuries | 235.3 | 19677384.8 | 4.3% |
| Interpersonal violence | 114.4 | 5624495.4 | Interpersonal violence | 95.5 | 5575519.3 | Interpersonal violence | 81.5 | 5692551.7 | Conflict and terrorism | 79.3 | 6394412.0 | 205.2% |
| Road injuries | 102.7 | 4937465.9 | Road injuries | 86.9 | 5010624.0 | Road injuries | 68.3 | 4750463.7 | Interpersonal violence | 65.3 | 5431682.7 | -3.4% |
| Animal contact | 78.9 | 3840931.7 | Animal contact | 70.8 | 4139658.9 | Animal contact | 61.1 | 4270232.3 | Road injuries | 54.1 | 4556986.1 | -7.7% |
| Foreign body | 62.1 | 3159449.6 | Foreign body | 55.7 | 3341071.6 | Conflict and terrorism | 57.8 | 4174742.1 | Animal contact | 54.0 | 4491093.6 | 16.9% |
| Conflict and terrorism | 36.0 | 2095041.3 | Conflict and terrorism | 51.8 | 3340710.9 | Foreign body | 51.3 | 3587849.6 | Foreign body | 48.7 | 3959196.4 | 25.3% |
| Fire, heat, and hot substances | 13.4 | 642033.0 | Fire, heat, and hot substances | 12.6 | 731309.4 | Fire, heat, and hot substances | 11.9 | 829058.5 | Police conflict and executions | 18.0 | 1399961.3 | 759.2% |
| Environmental heat and cold exposure | 12.2 | 589138.8 | Other transport injuries | 11.1 | 647084.2 | Other transport injuries | 9.4 | 657641.8 | Fire, heat, and hot substances | 10.9 | 914047.2 | 42.4% |
| Other transport injuries | 12.0 | 587498.6 | Environmental heat and cold exposure | 10.4 | 588426.7 | Environmental heat and cold exposure | 9.3 | 650269.7 | Other transport injuries | 9.1 | 755779.0 | 28.6% |
| Self-harm | 10.6 | 487520.2 | Self-harm | 10.1 | 583426.6 | Self-harm | 8.7 | 602809.5 | Environmental heat and cold exposure | 8.3 | 687572.9 | 16.7% |
| Drowning | 4.1 | 198951.5 | Exposure to forces of nature | 5.5 | 352359.6 | Exposure to forces of nature | 8.0 | 563406.2 | Self-harm | 7.9 | 641971.7 | 31.7% |
| Poisonings | 3.8 | 179378.6 | Police conflict and executions | 4.7 | 294326.1 | Police conflict and executions | 7.2 | 508917.0 | Exposure to forces of nature | 7.5 | 633617.3 | 349.4% |
| Police conflict and executions | 2.9 | 162947.1 | Drowning | 3.4 | 194811.3 | Poisonings | 2.9 | 201458.7 | Poisonings | 2.3 | 192670.2 | 7.4% |
| Exposure to forces of nature | 2.4 | 140982.8 | Poisonings | 3.1 | 176796.8 | Drowning | 2.5 | 172844.5 | Drowning | 1.9 | 158520.7 | -20.3% |
| Electrocution | 1.1 | 49087.5 | Electrocution | 1.0 | 53748.2 | Electrocution | 0.8 | 58612.4 | Electrocution | 0.7 | 59482.2 | 21.2% |
| Adverse effects of medical treatment | 0.0 | 0.0 | Adverse effects of medical treatment | 0.0 | 0.0 | Adverse effects of medical treatment | 0.0 | 0.0 | Adverse effects of medical treatment | 0.0 | 0.0 | 0.0% |

# Table S26. Rankings, ASR of prevalence, and number of cases of AMP-Toe by injury type (1990–2021), with percentage changes in absolute cases (1990–2021).

| **1990** | | | **2000** | | | **2010** | | | **2021** | | | **Percentage change (1990-2021)** |
| --- | --- | --- | --- | --- | --- | --- | --- | --- | --- | --- | --- | --- |
| **Rank** | **ASR** | **Cases** | **Rank** | **ASR** | **Cases** | **Rank** | **ASR** | **Cases** | **Rank** | **ASR** | **Cases** |  |
| Falls | 960.2 | 46921884.6 | Falls | 950.4 | 55513553.4 | Falls | 1160.1 | 81295890.0 | Falls | 872.8 | 73239986.0 | 56.1% |
| Exposure to mechanical forces | 372.9 | 17736444.5 | Exposure to mechanical forces | 325.7 | 18710626.6 | Exposure to mechanical forces | 296.1 | 20583479.7 | Exposure to mechanical forces | 247.9 | 20809022.2 | 17.3% |
| Other unintentional injuries | 318.9 | 15358548.0 | Other unintentional injuries | 272.6 | 15805915.6 | Other unintentional injuries | 240.9 | 16856239.3 | Other unintentional injuries | 195.4 | 16389855.9 | 6.7% |
| Road injuries | 126.6 | 6126622.0 | Road injuries | 109.6 | 6349060.2 | Road injuries | 88.0 | 6135120.8 | Conflict and terrorism | 93.8 | 7591835.9 | 202.0% |
| Interpersonal violence | 97.1 | 4734679.4 | Interpersonal violence | 81.7 | 4743978.6 | Interpersonal violence | 70.4 | 4947929.2 | Road injuries | 70.6 | 5935406.3 | -3.1% |
| Animal contact | 67.6 | 3303057.1 | Conflict and terrorism | 62.3 | 3983327.7 | Conflict and terrorism | 68.6 | 4907267.8 | Interpersonal violence | 55.7 | 4645125.9 | -1.9% |
| Foreign body | 58.8 | 2985459.8 | Animal contact | 60.9 | 3569649.3 | Animal contact | 52.6 | 3680414.8 | Animal contact | 46.4 | 3854619.9 | 16.7% |
| Conflict and terrorism | 43.8 | 2514036.3 | Foreign body | 52.7 | 3155019.1 | Foreign body | 48.4 | 3387093.1 | Foreign body | 46.0 | 3742755.3 | 25.4% |
| Other transport injuries | 14.5 | 717535.5 | Other transport injuries | 13.3 | 780608.0 | Other transport injuries | 11.6 | 812243.5 | Police conflict and executions | 21.2 | 1654132.6 | 828.0% |
| Fire, heat, and hot substances | 12.4 | 593614.2 | Fire, heat, and hot substances | 11.6 | 675295.9 | Fire, heat, and hot substances | 10.9 | 764416.6 | Other transport injuries | 11.2 | 933966.2 | 30.2% |
| Environmental heat and cold exposure | 12.3 | 592092.4 | Environmental heat and cold exposure | 10.2 | 591374.5 | Environmental heat and cold exposure | 9.3 | 649892.2 | Fire, heat, and hot substances | 10.1 | 842096.8 | 41.9% |
| Self-harm | 10.3 | 469795.0 | Self-harm | 10.0 | 561645.1 | Police conflict and executions | 8.5 | 602995.2 | Environmental heat and cold exposure | 8.2 | 683355.6 | 15.4% |
| Drowning | 4.1 | 202198.8 | Police conflict and executions | 5.6 | 351482.4 | Self-harm | 8.4 | 578524.6 | Self-harm | 7.2 | 615060.5 | 30.9% |
| Poisonings | 3.7 | 194500.8 | Exposure to forces of nature | 4.7 | 300168.9 | Exposure to forces of nature | 6.6 | 468170.1 | Exposure to forces of nature | 6.6 | 527733.6 | 339.8% |
| Police conflict and executions | 3.5 | 178250.6 | Drowning | 3.4 | 198319.5 | Poisonings | 2.9 | 197875.2 | Poisonings | 2.3 | 189762.0 | -2.4% |
| Exposure to forces of nature | 2.1 | 120000.7 | Poisonings | 3.0 | 173445.0 | Drowning | 2.6 | 176123.0 | Drowning | 1.9 | 161958.0 | -19.9% |
| Electrocution | 1.1 | 52212.8 | Electrocution | 1.0 | 57126.5 | Electrocution | 0.9 | 62317.4 | Electrocution | 0.8 | 63336.3 | 21.3% |
| Adverse effects of medical treatment | 0.0 | 0.0 | Adverse effects of medical treatment | 0.0 | 0.0 | Adverse effects of medical treatment | 0.0 | 0.0 | Adverse effects of medical treatment | 0.0 | 0.0 | 0.0% |

# Table S27. Rankings, ASR of prevalence, and number of cases of AMP-UL2 by injury type (1990–2021), with percentage changes in absolute cases (1990–2021).

| **1990** | | | **2000** | | | **2010** | | | **2021** | | | **Percentage change (1990-2021)** |
| --- | --- | --- | --- | --- | --- | --- | --- | --- | --- | --- | --- | --- |
| **Rank** | **ASR** | **Cases** | **Rank** | **ASR** | **Cases** | **Rank** | **ASR** | **Cases** | **Rank** | **ASR** | **Cases** |  |
| Falls | 64.9 | 3114391.6 | Falls | 65.0 | 3738663.7 | Falls | 79.0 | 5497187.6 | Falls | 61.4 | 5178479.4 | 66.3% |
| Exposure to mechanical forces | 20.0 | 949348.1 | Exposure to mechanical forces | 17.9 | 1024959.0 | Exposure to mechanical forces | 16.6 | 1155075.2 | Conflict and terrorism | 16.2 | 1317912.5 | 181.6% |
| Interpersonal violence | 17.1 | 833817.8 | Interpersonal violence | 14.7 | 855451.5 | Interpersonal violence | 12.8 | 899342.2 | Exposure to mechanical forces | 13.9 | 1169922.4 | 23.2% |
| Other unintentional injuries | 12.4 | 586881.7 | Conflict and terrorism | 11.5 | 738812.4 | Conflict and terrorism | 12.1 | 875221.1 | Interpersonal violence | 10.0 | 834599.1 | 0.1% |
| Road injuries | 11.4 | 536824.1 | Other unintentional injuries | 10.6 | 604234.9 | Other unintentional injuries | 9.2 | 639602.8 | Other unintentional injuries | 7.4 | 622923.3 | 6.1% |
| Conflict and terrorism | 8.0 | 468061.3 | Road injuries | 10.4 | 591109.6 | Road injuries | 8.8 | 608697.2 | Road injuries | 7.3 | 620422.9 | 15.6% |
| Foreign body | 4.6 | 226037.0 | Foreign body | 4.2 | 244409.9 | Foreign body | 3.7 | 258469.7 | Foreign body | 3.7 | 307281.9 | 35.9% |
| Animal contact | 4.2 | 200471.5 | Animal contact | 3.8 | 219378.9 | Animal contact | 3.3 | 227882.2 | Animal contact | 2.9 | 240725.1 | 20.1% |
| Self-harm | 3.5 | 159820.6 | Self-harm | 3.4 | 191520.3 | Self-harm | 2.9 | 198673.9 | Police conflict and executions | 2.8 | 218713.3 | 769.7% |
| Other transport injuries | 2.3 | 109258.5 | Other transport injuries | 2.1 | 121612.3 | Other transport injuries | 1.9 | 130891.2 | Self-harm | 2.5 | 212001.6 | 32.6% |
| Environmental heat and cold exposure | 1.3 | 64618.4 | Environmental heat and cold exposure | 1.1 | 64614.4 | Exposure to forces of nature | 1.3 | 92587.0 | Other transport injuries | 1.8 | 153666.2 | 40.6% |
| Fire, heat, and hot substances | 1.1 | 54052.3 | Fire, heat, and hot substances | 1.1 | 62691.1 | Police conflict and executions | 1.1 | 78913.8 | Exposure to forces of nature | 1.3 | 103411.5 | 420.8% |
| Drowning | 0.6 | 32771.4 | Exposure to forces of nature | 0.8 | 48483.3 | Fire, heat, and hot substances | 1.0 | 70820.6 | Fire, heat, and hot substances | 0.9 | 77428.1 | 43.2% |
| Poisonings | 0.6 | 27378.8 | Police conflict and executions | 0.7 | 45195.5 | Environmental heat and cold exposure | 1.0 | 69119.3 | Environmental heat and cold exposure | 0.9 | 71785.1 | 11.1% |
| Police conflict and executions | 0.4 | 25147.3 | Drowning | 0.5 | 30402.8 | Poisonings | 0.4 | 30646.9 | Poisonings | 0.3 | 27973.6 | 2.2% |
| Exposure to forces of nature | 0.4 | 19855.7 | Poisonings | 0.5 | 28420.7 | Drowning | 0.4 | 24699.5 | Drowning | 0.3 | 21220.7 | -35.2% |
| Electrocution | 0.1 | 6348.7 | Electrocution | 0.1 | 6947.9 | Electrocution | 0.1 | 7590.7 | Electrocution | 0.1 | 7711.3 | 21.5% |
| Adverse effects of medical treatment | 0.0 | 0.0 | Adverse effects of medical treatment | 0.0 | 0.0 | Adverse effects of medical treatment | 0.0 | 0.0 | Adverse effects of medical treatment | 0.0 | 0.0 | 0.0% |

# Table S28. Rankings, ASR of prevalence, and number of cases of AMP-UL1 by injury type (1990–2021), with percentage changes in absolute cases (1990–2021).

| **1990** | | | **2000** | | | **2010** | | | **2021** | | | **Percentage change (1990-2021)** |
| --- | --- | --- | --- | --- | --- | --- | --- | --- | --- | --- | --- | --- |
| **Rank** | **ASR** | **Cases** | **Rank** | **ASR** | **Cases** | **Rank** | **ASR** | **Cases** | **Rank** | **ASR** | **Cases** |  |
| Falls | 60.7 | 2893638.5 | Falls | 60.7 | 3480004.8 | Falls | 73.4 | 5097169.1 | Falls | 57.5 | 4854570.1 | 67.8% |
| Road injuries | 16.8 | 785771.7 | Road injuries | 15.5 | 877401.2 | Road injuries | 13.3 | 917289.6 | Conflict and terrorism | 15.0 | 1216680.0 | 185.3% |
| Exposure to mechanical forces | 14.7 | 690975.2 | Exposure to mechanical forces | 13.0 | 739655.8 | Exposure to mechanical forces | 11.9 | 825905.2 | Road injuries | 11.1 | 945494.2 | 20.3% |
| Other unintentional injuries | 12.6 | 606696.3 | Conflict and terrorism | 10.8 | 677125.9 | Conflict and terrorism | 11.1 | 803565.7 | Exposure to mechanical forces | 10.0 | 840090.5 | 21.6% |
| Interpersonal violence | 12.5 | 588538.9 | Interpersonal violence | 10.7 | 621173.9 | Other unintentional injuries | 9.4 | 653893.7 | Other unintentional injuries | 7.7 | 647776.4 | 6.8% |
| Conflict and terrorism | 7.3 | 426468.8 | Other unintentional injuries | 10.5 | 613244.4 | Interpersonal violence | 9.3 | 652318.9 | Interpersonal violence | 7.3 | 607146.7 | 3.2% |
| Animal contact | 3.9 | 186888.6 | Other transport injuries | 3.6 | 206195.4 | Other transport injuries | 3.3 | 227953.0 | Other transport injuries | 3.2 | 268860.7 | 44.1% |
| Other transport injuries | 3.9 | 186623.7 | Animal contact | 3.6 | 205474.0 | Animal contact | 3.1 | 215237.6 | Foreign body | 2.9 | 238335.0 | 30.1% |
| Foreign body | 3.8 | 183190.1 | Foreign body | 3.4 | 199766.0 | Foreign body | 3.1 | 215156.7 | Animal contact | 2.7 | 230660.5 | 23.4% |
| Self-harm | 3.4 | 156168.4 | Self-harm | 3.3 | 186943.2 | Self-harm | 2.8 | 193794.1 | Self-harm | 2.6 | 206595.2 | 32.3% |
| Environmental heat and cold exposure | 1.0 | 47738.5 | Fire, heat, and hot substances | 0.9 | 54057.3 | Exposure to forces of nature | 1.4 | 100818.1 | Police conflict and executions | 2.4 | 203008.1 | 783.0% |
| Fire, heat, and hot substances | 1.0 | 46756.5 | Exposure to forces of nature | 0.8 | 52740.4 | Police conflict and executions | 1.0 | 72548.1 | Exposure to forces of nature | 1.4 | 112918.7 | 425.9% |
| Poisonings | 0.6 | 28788.9 | Environmental heat and cold exposure | 0.8 | 48013.5 | Fire, heat, and hot substances | 0.9 | 60785.4 | Fire, heat, and hot substances | 0.8 | 66155.8 | 41.5% |
| Drowning | 0.5 | 26111.4 | Police conflict and executions | 0.7 | 41342.6 | Environmental heat and cold exposure | 0.7 | 51649.8 | Environmental heat and cold exposure | 0.6 | 53661.2 | 12.4% |
| Police conflict and executions | 0.4 | 22991.5 | Poisonings | 0.5 | 29963.4 | Poisonings | 0.5 | 32337.8 | Poisonings | 0.4 | 29499.7 | 2.5% |
| Exposure to forces of nature | 0.4 | 21471.2 | Drowning | 0.4 | 24232.4 | Drowning | 0.3 | 19798.7 | Drowning | 0.2 | 17039.4 | -34.7% |
| Electrocution | 0.2 | 8837.6 | Electrocution | 0.2 | 9625.3 | Electrocution | 0.2 | 10428.0 | Electrocution | 0.1 | 10527.9 | 19.1% |
| Adverse effects of medical treatment | 0.0 | 0.0 | Adverse effects of medical treatment | 0.0 | 0.0 | Adverse effects of medical treatment | 0.0 | 0.0 | Adverse effects of medical treatment | 0.0 | 0.0 | 0.0% |

**Figure S1. The ASR and EAPC of Global distribution of the burden for** **AMP-F from 1990 to 2021.**

(A) Global distribution of the burden for AMP-F from 1990 to 2021

(B) Global distribution of ASR YLDs of AMP-F on from 1990 to 2021.

(C) Global distribution of EAPC of AMP-F from 1990 to 2021.

(D) Global distribution of EAPC of ASR YLDs of AMP-F from 1990 to 2021

**Figure S2. The ASR and EAPC of Global distribution of the burden for AMP-LL2 from 1990 to 2021.**

(A) Global distribution of the burden for AMP-LL2 from 1990 to 2021

(B) Global distribution of ASR YLDs of AMP-LL2 from 1990 to 2021.

(C) Global distribution of EAPC of AMP-LL2 from 1990 to 2021.

(D) Global distribution of EAPC of ASR YLDs of AMP-LL2 from 1990 to 2021

**Figure S3. The ASR and EAPC of Global distribution of the burden for AMP-LL1 from 1990 to 2021.**

(A) Global distribution of the burden for AMP-LL1 from 1990 to 2021

(B) Global distribution of ASR YLDs of AMP-LL1 from 1990 to 2021.

(C) Global distribution of EAPC of AMP-LL1 from 1990 to 2021.

(D) Global distribution of EAPC of ASR YLDs of AMP-LL1 from 1990 to 2021

**Figure S4. The ASR and EAPC of Global distribution of the burden for AMP-Th from 1990 to 2021.**

(A) Global distribution of the burden for AMP-Th from 1990 to 2021

(B) Global distribution of ASR YLDs of AMP-Th from 1990 to 2021.

(C) Global distribution of EAPC of AMP-Th from 1990 to 2021.

(D) Global distribution of EAPC of ASR YLDs of AMP-Th from 1990 to 2021

**Figure S5. The ASR and EAPC of Global distribution of the burden for AMP-Toe from 1990 to 2021.**

(A) Global distribution of the burden for AMP-Toe from 1990 to 2021

(B) Global distribution of ASR YLDs of AMP-Toe from 1990 to 2021.

(C) Global distribution of EAPC of AMP-Toe from 1990 to 2021.

(D) Global distribution of EAPC of ASR YLDs of AMP-Toe from 1990 to 2021

**Figure S6. The ASR and EAPC of Global distribution of the burden for AMP-UL2 from 1990 to 2021.**

The figure represents:

(A) Global distribution of the burden for AMP-UL2 from 1990 to 2021

(B) Global distribution of ASR YLDs of AMP-UL2 from 1990 to 2021.

(C) Global distribution of EAPC of AMP-UL2 from 1990 to 2021.

(D) Global distribution of EAPC of ASR YLDs of AMP-UL2 from 1990 to 2021

**Figure S7. The ASR and EAPC of Global distribution of the burden for AMP-UL1 from 1990 to 2021.**

(A) Global distribution of the burden for AMP-UL1 from 1990 to 2021

(B) Global distribution of ASR YLDs of AMP-UL1 from 1990 to 2021.

(C) Global distribution of EAPC of AMP-UL1 from 1990 to 2021.

(D) Global distribution of EAPC of ASR YLDs of AMP-UL1 from 1990 to 2021

**Figure S8. Gender-specific burden and YLDs of AMP-F across 21 GBD regions in 2021.**

The number of cases of prevalence (A) and YLDs (B) for AMP-F across 21 GBD regions.

**Figure S9. Gender-specific burden and YLDs of AMP-LL2 across 21 GBD regions in 2021.**

The number of cases of prevalence (A) and YLDs (B) for AMP-LL2 across 21 GBD regions.

**Figure S10. Gender-specific burden and YLDs of AMP-LL1 across 21 GBD regions in 2021.**

The number of cases of prevalence (A) and YLDs (B) for AMP-LL1 across 21 GBD regions.

**Figure S11. Gender-specific burden and YLDs of AMP-Th across 21 GBD regions in 2021.**

The number of cases of prevalence (A) and YLDs (B) for AMP-Th across 21 GBD regions.

**Figure S12. Gender-specific burden and YLDs of AMP-Toe across 21 GBD regions in 2021.**

The number of cases of prevalence (A) and YLDs (B) for AMP-Toe across 21 GBD regions.

**Figure S13. Gender-specific burden and YLDs of AMP-UL2 across 21 GBD regions in 2021.**

The number of cases of prevalence (A) and YLDs (B) for AMP-UL2 across 21 GBD regions.

**Figure S14. Gender-specific burden and YLDs of AMP-UL1 across 21 GBD regions in 2021.**

The number of cases of prevalence (A) and YLDs (B) for AMP-UL1 across 21 GBD regions.

**Figure S15.** **Curves of the correlation between the prevalence of AMP-F and SDI, and YLDs with SDI in 2021.**

The figure represents:(A) Prevalence and SDI; (B) YLDs and SDI

**Figure S16.** **Curves of the correlation between the prevalence of AMP-LL2 and SDI, and YLDs with SDI in 2021.**

The figure represents:(A) Prevalence and SDI; (B) YLDs and SDI

**Figure S17. Curves of the correlation between the prevalence of AMP-LL1 and SDI, and YLDs with SDI in 2021.**

The figure represents:(A) Prevalence and SDI; (B) YLDs and SDI

**Figure S18. Curves of the correlation between the prevalence of AMP-Th and SDI, and YLDs with SDI in 2021.**

The figure represents:(A) Prevalence and SDI; (B) YLDs and SDI

**Figure S19. Curves of the correlation between the prevalence of AMP-Toe and SDI, and YLDs with SDI in 2021.**

The figure represents:(A) Prevalence and SDI; (B) YLDs and SDI

**Figure S20. Curves of the correlation between the prevalence of AMP-UL2 and SDI, and YLDs with SDI in 2021.**

The figure represents:(A) Prevalence and SDI; (B) YLDs and SDI

**Figure S21. Curves of the correlation between the prevalence of AMP-UL1 and SDI, and YLDs with SDI in 2021.**

The figure represents:(A) Prevalence and SDI; (B) YLDs and SDI

**Figure S22. Temporal validation of the Bayesian age–period–cohort (BAPC) prediction model.**

(A) Observed and predicted ASR of prevalence and YLDs for amputations during 2016–2021. Predictions were generated by fitting the BAPC model to data from 1990–2015. Shaded areas represent the predicted 95% credible intervals.

(B) Mean absolute percentage error (MAPE) of the predicted ASRs for each amputation subtype.

(C) Coverage of observed ASRs within the predicted 95% credible intervals for each amputation subtype.

# Figure S23. Ranking of Causes of Injury Leading to Amputation Worldwide, 1990–2021, and Trends in Prevalence.

Data dimensions: Covers rankings for 1990, 2000, 2010 and 2021, as well as the prevalence rate for 2021.

Colour coding:

Orange (Transport injuries): Transport-related injuries (e.g., road traffic injuries, other transport-related injuries).

Purple (Unintentional injuries): Unintentional injuries (e.g., falls, exposure to mechanical forces, etc.).

Yellow (Self-harm and interpersonal violence): Self-harm and interpersonal violence (e.g., conflict and terrorism, interpersonal violence, etc.).

# Figure S24. Ranking of Causes of Injury Leading to AMP-F Worldwide, 1990–2021, and Trends in Prevalence.

Data dimensions: Covers rankings for 1990, 2000, 2010 and 2021, as well as the prevalence rate for 2021.

Colour coding:

Orange (Transport injuries): Transport-related injuries (e.g., road traffic injuries, other transport-related injuries).

Purple (Unintentional injuries): Unintentional injuries (e.g., falls, exposure to mechanical forces, etc.).

Yellow (Self-harm and interpersonal violence): Self-harm and interpersonal violence (e.g., conflict and terrorism, interpersonal violence, etc.).

# Figure S25. Ranking of Causes of Injury Leading to AMP-LL2 Worldwide, 1990–2021, and Trends in Prevalence.

Data dimensions: Covers rankings for 1990, 2000, 2010 and 2021, as well as the prevalence rate for 2021.

Colour coding:

Orange (Transport injuries): Transport-related injuries (e.g., road traffic injuries, other transport-related injuries).

Purple (Unintentional injuries): Unintentional injuries (e.g., falls, exposure to mechanical forces, etc.).

Yellow (Self-harm and interpersonal violence): Self-harm and interpersonal violence (e.g., conflict and terrorism, interpersonal violence, etc.).

# Figure S26. Ranking of Causes of Injury Leading to AMP-LL1 Worldwide, 1990–2021, and Trends in Prevalence.

Data dimensions: Covers rankings for 1990, 2000, 2010 and 2021, as well as the prevalence rate for 2021.

Colour coding:

Orange (Transport injuries): Transport-related injuries (e.g., road traffic injuries, other transport-related injuries).

Purple (Unintentional injuries): Unintentional injuries (e.g., falls, exposure to mechanical forces, etc.).

Yellow (Self-harm and interpersonal violence): Self-harm and interpersonal violence (e.g., conflict and terrorism, interpersonal violence, etc.).

# Figure S27. Ranking of Causes of Injury Leading to AMP-Th Worldwide, 1990–2021, and Trends in Prevalence.

Data dimensions: Covers rankings for 1990, 2000, 2010 and 2021, as well as the prevalence rate for 2021.

Colour coding:

Orange (Transport injuries): Transport-related injuries (e.g., road traffic injuries, other transport-related injuries).

Purple (Unintentional injuries): Unintentional injuries (e.g., falls, exposure to mechanical forces, etc.).

Yellow (Self-harm and interpersonal violence): Self-harm and interpersonal violence (e.g., conflict and terrorism, interpersonal violence, etc.).

# Figure S28. Ranking of Causes of Injury Leading to AMP-Toe Worldwide, 1990–2021, and Trends in Prevalence.

Data dimensions: Covers rankings for 1990, 2000, 2010 and 2021, as well as the prevalence rate for 2021.

Colour coding:

Orange (Transport injuries): Transport-related injuries (e.g., road traffic injuries, other transport-related injuries).

Purple (Unintentional injuries): Unintentional injuries (e.g., falls, exposure to mechanical forces, etc.).

Yellow (Self-harm and interpersonal violence): Self-harm and interpersonal violence (e.g., conflict and terrorism, interpersonal violence, etc.).

# Figure S29. Ranking of Causes of Injury Leading to AMP-UL2 Worldwide, 1990–2021, and Trends in Prevalence.

Data dimensions: Covers rankings for 1990, 2000, 2010 and 2021, as well as the prevalence rate for 2021.

Colour coding:

Orange (Transport injuries): Transport-related injuries (e.g., road traffic injuries, other transport-related injuries).

Purple (Unintentional injuries): Unintentional injuries (e.g., falls, exposure to mechanical forces, etc.).

Yellow (Self-harm and interpersonal violence): Self-harm and interpersonal violence (e.g., conflict and terrorism, interpersonal violence, etc.).

# Figure S30. Ranking of Causes of Injury Leading to AMP-UL1 Worldwide, 1990–2021, and Trends in Prevalence.

Data dimensions: Covers rankings for 1990, 2000, 2010 and 2021, as well as the prevalence rate for 2021.

Colour coding:

Orange (Transport injuries): Transport-related injuries (e.g., road traffic injuries, other transport-related injuries).

Purple (Unintentional injuries): Unintentional injuries (e.g., falls, exposure to mechanical forces, etc.).

Yellow (Self-harm and interpersonal violence): Self-harm and interpersonal violence (e.g., conflict and terrorism, interpersonal violence, etc.).
